# Iridium nitrenoid-catalyzed atroposelective C2 arylation of indoles

**DOI:** 10.1039/d6sc04377a

**Published:** 2026-07-02

**Authors:** Fen Mi, Wenrui Zheng, Yuhan Cao, Liang-Wen Qi, Yixin Lu

**Affiliations:** a Department of Chemistry, National University of Singapore 3 Science Drive 3 Singapore 117543 Singapore chmlyx@nus.edu.sg; b Faculty of Pharmaceutical Sciences, Shenzhen University of Advanced Technology Gongchang Road 1, Guangming District 518107 Shenzhen China qiliangwen@suat-sz.edu.cn; c Department of Chemistry, State Key Laboratory of Synthetic Biology, Tianjin University Tianjin 300072 China

## Abstract

Axially chiral indole derivatives are valuable in synthetic and medicinal chemistry, yet the catalytic synthesis of C2-aryl indole atropisomers remains challenging. Herein, we report a highly atroposelective C2-arylation of indoles with β-azidonaphthalenes *via* iridium nitrenoid-promoted arene C–H functionalization. Employing a chiral oxazoline *N*,*C*-chelating Cp*Ir(iii) complex as the catalyst, the reaction proceeds under mild conditions, delivering configurationally stable C2-aryl indoles in good yields with high enantioselectivities. The strategy provides a straightforward method for atroposelective synthesis of C2-aryl indoles, a class of compounds that hold great potential in asymmetric catalysis and medicinal chemistry.

## Abstract 2

A highly atroposelective C2-arylation of indoles with β-azidonaphthalenes has been developed, allowing facile access to C2-arylindoles through iridium nitrenoid-promoted arene C–H functionalization.

## Graphical abstract

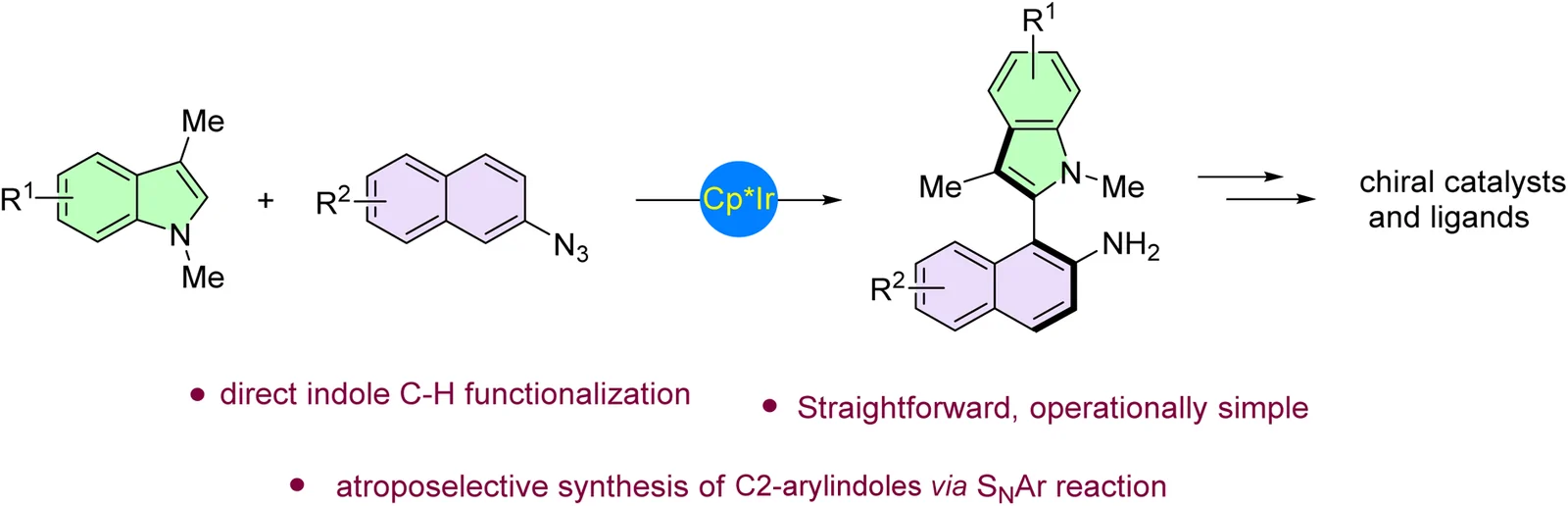

## Introduction

**Fig. 1:**
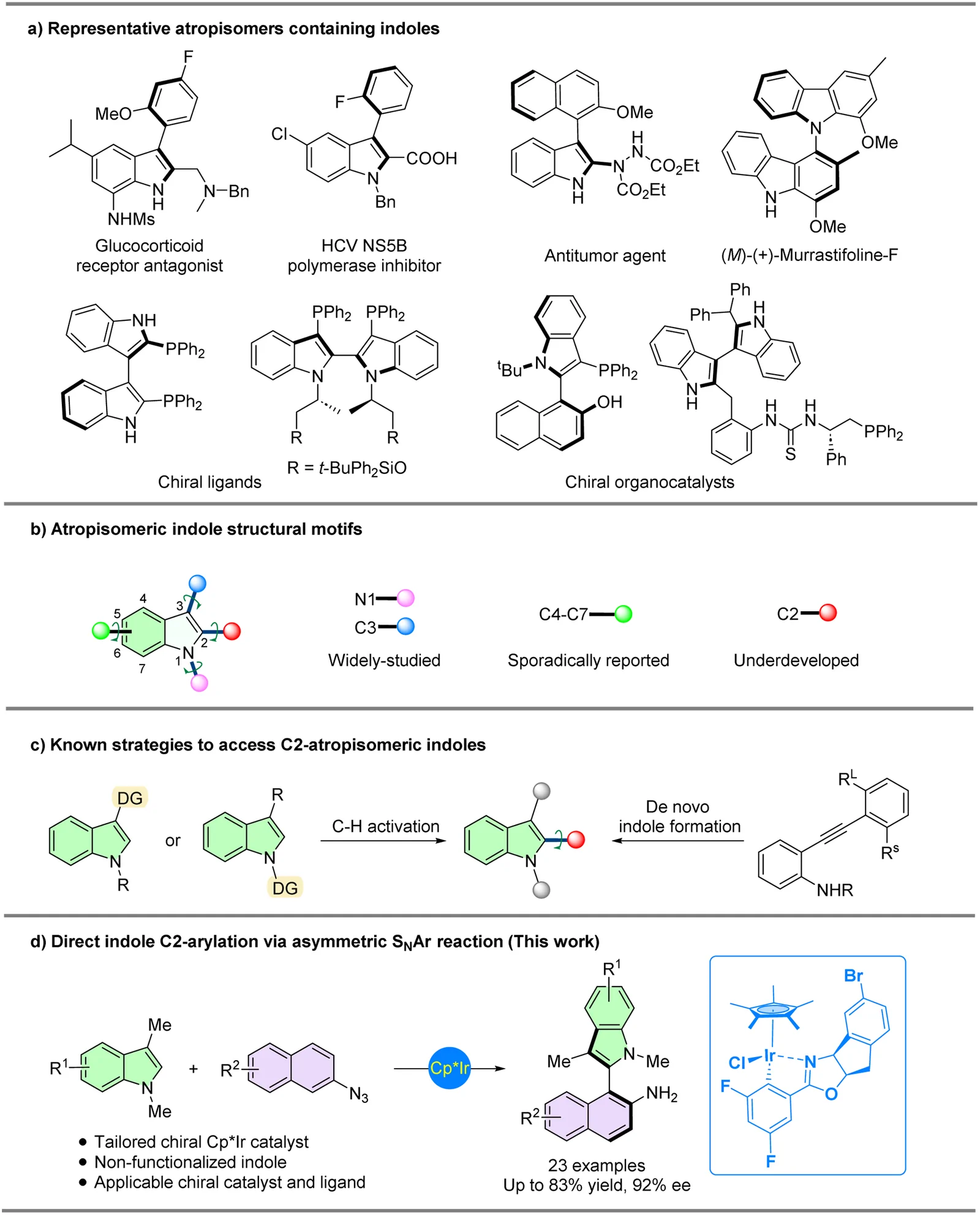
Background and our working hypothesis. (a) representative atropisomers containing indoles; (b) atropisomeric indole structural motifs; (c) known strategies to access C2-atropisomeric indoles; (d) direct indole C2-arylation *via* asymmetric S_N_Ar reaction (this work).

Axially chiral molecules are tremendously useful in synthetic organic chemistry, drug discovery, and materials science, providing tunable three-dimensional architectures that afford precise steric and electronic control and enable high-fidelity in molecular recognition.^1^ In asymmetric catalysis, axially chiral frameworks are regarded as privileged scaffolds because their inherent rigidity and tunability furnish highly selective chiral environments. In drug discovery, unlike the more common central chirality, axial chirality imposes a notably rigid stereochemical arrangement that can enhance binding affinity, metabolic stability, and target selectivity. Consequently, the past decade has seen a growing number of powerful ligands and catalysts, bioactive small molecules and approved pharmaceuticals whose performances and activities rely on atropisomerism. In this context, indole-based axially chiral scaffolds have garnered considerable interest, as indole is among the most ubiquitous heteroaromatic motifs in natural products and bioactive small molecules,^2–5^ chiral ligands,^6,7^ and organocatalysts (Fig. 1a).^8–10^

In recent years, numerous catalytic strategies for constructing indole-based axially chiral scaffolds have emerged and can be broadly classified according to the substitution site and the structural nature of the stereogenic axis (Fig. 1b). Among these, C3- and N1-(hetero)aryl atropisomeric indoles have received the most attention.^11^ To date, C3-indole atropisomers have been synthesized primarily through three established strategies: (i) exploiting the intrinsic reactivity of pre-assembled biaryls;^12–15^ (ii) directing-group-assisted catalytic C3 functionalization of C2-substituted indoles;^16–26^ and (iii) *de novo* construction of C3-(hetero)aryl indole scaffolds.^27–31^ For example, leveraging chiral phosphoric acid (CPA) catalysis, Tan and co-workers developed C3-nucleophilic additions of indoles to diverse electrophiles, including azobenzenes, nitrosonaphthalenes, and quinones. In parallel, N1-(hetero)aryl indoles have been prepared either by indole N–H nucleophilic attack on electrophilic azonaphthalenes under CPA catalysis^32^ or *via de novo* annulation with alkyne partners under Rh- or Pd-catalysis.^33–39^ By contrast, atroposelective functionalization at the peripheral C4–C7 positions of indoles remains relatively underexplored, primarily because distal activation typically necessitates an appropriately positioned directing group on either the aryl ring or the indole nitrogen to achieve site- and stereoselectivity.^40,41^ Regarding C2-atropisomeric indoles, only a limited number of examples has been documented in the literature (Fig. 1c). In 2019, Li and co-workers disclosed a Rh(iii)-catalyzed asymmetric synthesis of axially chiral biindolyls through integration of C–H activation and alkyne cyclization.^42^ Around the same time, Yan *et al.* reported a quinine-derived thiourea-catalyzed annulation of *ortho*-alkynylanilines for atroposelective synthesis of axially chiral naphthyl-C2-indoles.^43^ Shortly thereafter, Ackermann and Wencel-Delord^44^ disclosed a cobalt-catalyzed enantioselective C2-arylation of indoles, while You independently reported the rhodium-catalyzed variant.^45^ By developing a palladium-catalyzed Cacchi reaction, Zhu and co-workers accomplished asymmetric synthesis of axially chiral 2,3-disubstituted indoles.^46^ Nevertheless, these strategies depend on particular directing groups or on specific structural features of the indole substrate, thus being less general.

Recently, we disclosed an iridium nitrenoid-enabled arene C–H functionalization of aryl azides. Using tailored chiral oxazoline-ligated iridium catalysts with readily available β-naphthalene azides and β-naphthols, we achieved the enantioselective synthesis of structurally diverse chiral NOBINs.^47^ Building on the blueprint of the metal-nitrenoid platform for arene activation and our ongoing interest in axially chiral motifs and atroposelective synthesis,^48–54^ we asked whether C2-aryl indole atropisomers could be accessed directly *via* asymmetric S_N_Ar C2-arylation of indoles. In our hypothesis, we bore in mind the following questions: (1) whether the metal-nitrenoid activation would be sufficient to activate the indole C2 position for nucleophilic reactivity; (2) the requirement of substituents at the N1 and C3 positions of indoles to ensure configurational stability of the targeted atropisomers; (3) whether simultaneous activation and stereocontrol of both the indole and the electrophile could deliver high enantioselectivity for the projected S_N_Ar reaction. Here, we document an iridium nitrenoid-triggered activation of β-azidonaphthalenes, allowing for efficient atroposelective C2-arylation of indoles. With the employment of a chiral oxazoline-bearing iridium complex, configurationally stable C2-aryl indole atropisomers were prepared with excellent enantioselectivities (Fig. 1d).

## Results and discussion

We initiated our study by examining the coupling of β-azidonaphthalene 1 with 1,3-dimethylindole 2 using a series of chiral oxazoline-chelated iridium catalysts, and the results are summarized in Table 1. In HFIP at 40 °C, Ir1 and Ir2 delivered the 2-arylindole adduct 3, but with poor enantioselectivity (entries 1 and 2). Switching to Ir3, which bears an indane-derived oxazoline, led to enhanced reaction efficiency (entry 3). Modifying the Cp ligand by replacing one methyl group with i-Pr (Ir4) or Bn (Ir5) did not lead to any improvement (entries 4 and 5). We then prepared catalysts featuring arenes with varied substituents in combination with a bromo-substituted indane moiety. It is noteworthy that 3,5-dihalogen substitution on the phenyl ring significantly enhanced enantioselectivity; fluorine proved most effective, giving 3 in 89% ee (entries 6–9). However, introducing more fluorine atoms to the aryl ring did not improve the reaction further (entries 10 and 11). Notably, Ir12, previously identified as the optimal catalyst for the synthesis of chiral NOBINs from β-naphthol, afforded only 28% yield and moderate 50% ee in this system (entry 12). Screening fluorinated solvents revealed that TFE gave poor reactivity, whereas perfluoro-*tert*-butyl alcohol [(CF_3_)_3_COH] was effective (entries 13 and 14). Finally, the order of addition proved crucial. Precomplexation of azide 1 with Ir7 in HFIP for 2 h generated a uniform chiral nitrenoid intermediate, enabling efficient asymmetric induction and affording the product with 92% ee (entry 15). In contrast, premixing indole 2 with Ir7 (entry 16) or combining all reactants simultaneously (entry 7) did not form a uniform chiral nitrenoid intermediate, thus resulting in substantially reduced enantioselectivity.

**Table 1:** Optimizing reaction conditions^a^

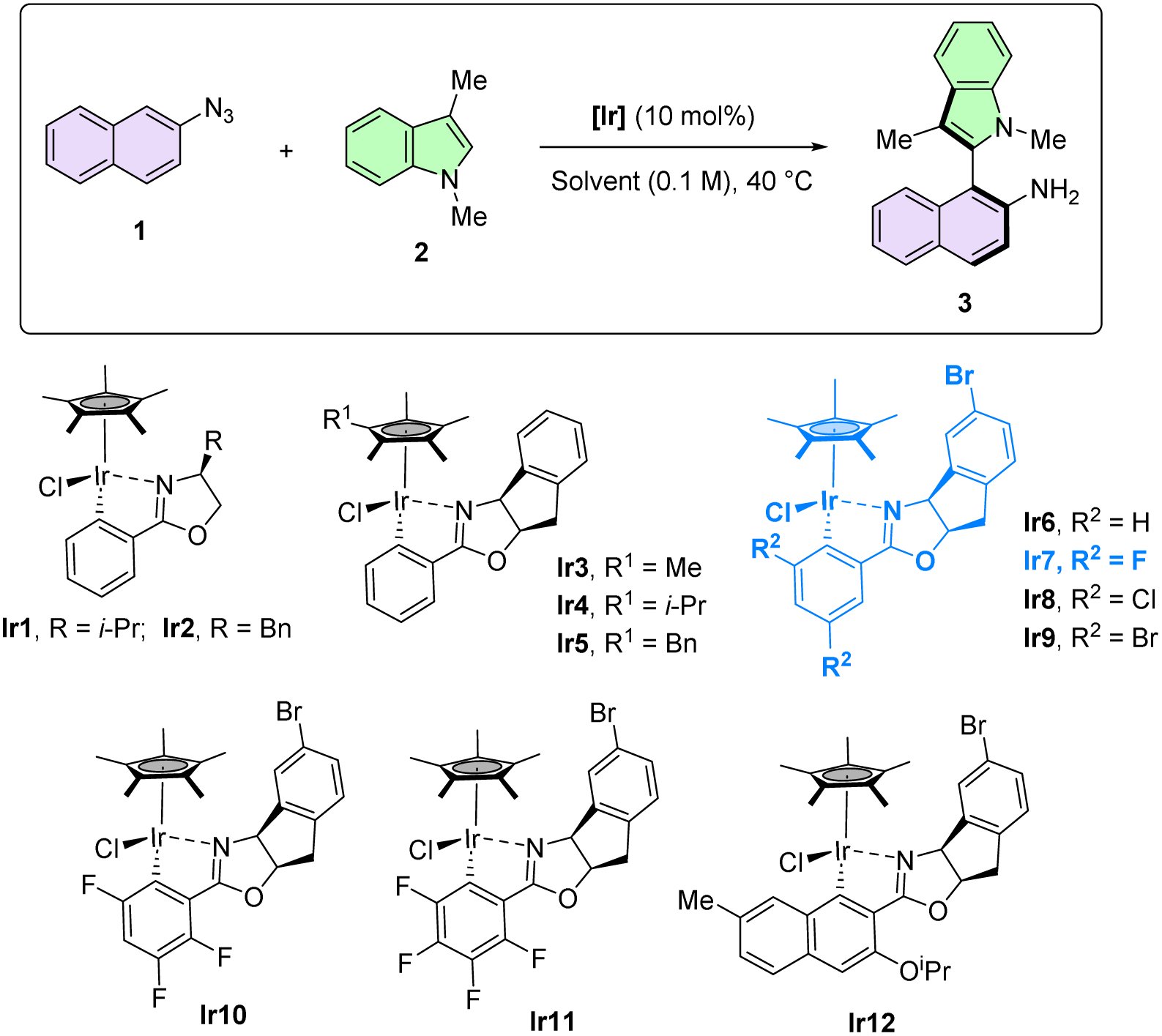				
Entry	Cat.	Solvent	Yield (%)^b^	ee (%)^c^
1	Ir1	HFIP	21	9
2	Ir2	HFIP	28	29
3	Ir3	HFIP	39	27
4	Ir4	HFIP	38	10
5	Ir5	HFIP	32	39
6	Ir6	HFIP	37	37
7	Ir7	HFIP	45	89
8	Ir8	HFIP	25	71
9	Ir9	HFIP	17	55
10	Ir10	HFIP	28	84
11	Ir11	HFIP	17	74
12	Ir12	HFIP	28	50
13	Ir7	TFE	10	70
14	Ir7	(CF_3_)_3_COH	56	87
15^d^	Ir7	**HFIP**	**53**	**92**
16^e^	Ir7	HFIP	48	76

a Unless otherwise stated, the reaction was performed with 0.05 mmol 1, 0.1 mmol 2, and 10 mol% Ir in 0.5 mL of solvent.

b Yield was determined by analysis of the crude ^1^H NMR spectrum using 1,3,5-trimethoxybenzene as the internal standard.

c Determined by chiral HPLC analysis using a chiral stationary phase.

d 1 and Ir7 were pre-mixed in HFIP for 2 h before adding 2.

e 2 and Ir7 were pre-mixed in HFIP for 2 h before adding 1.

**Fig. 2:**
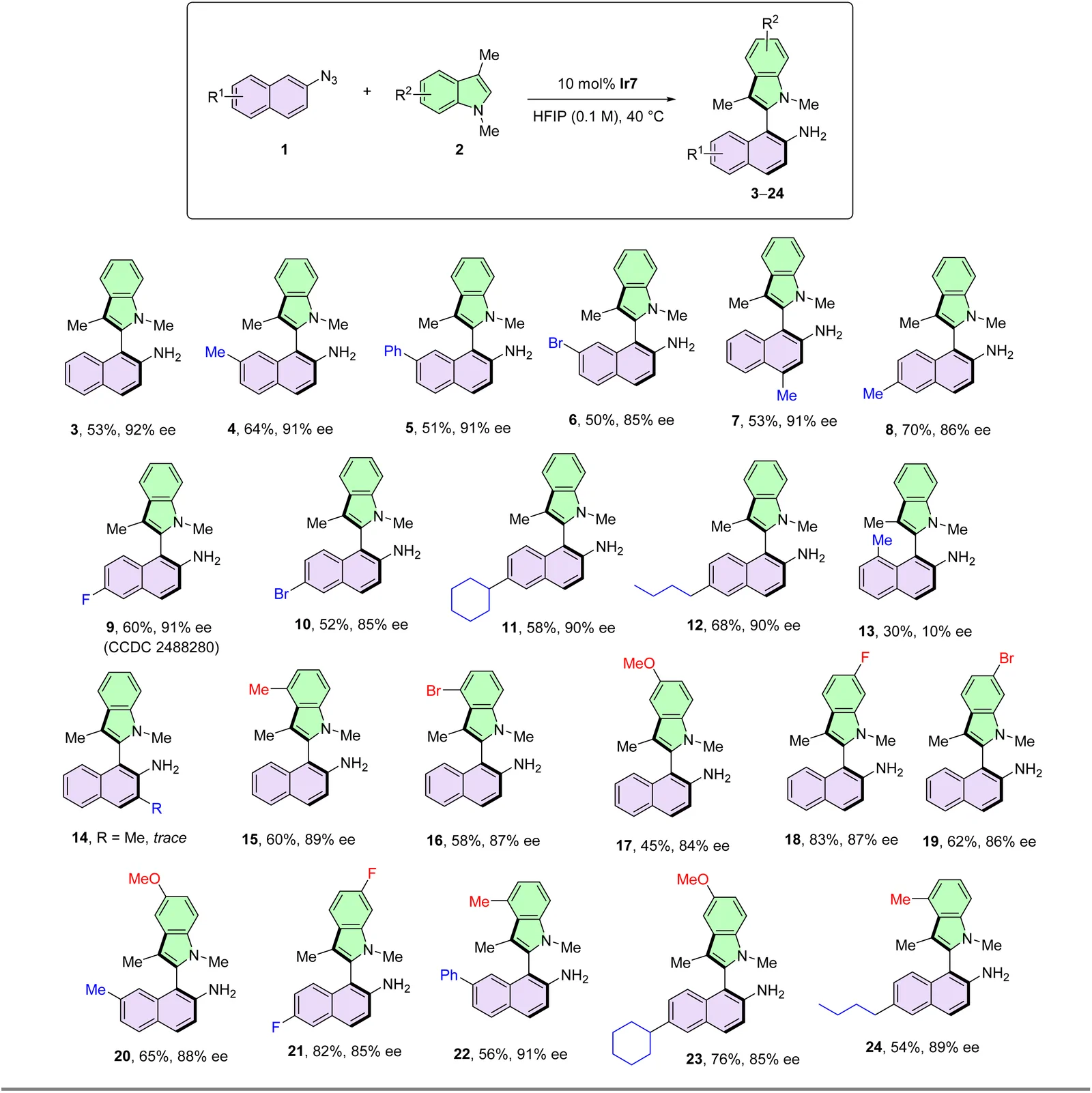
Substrate scope.

**Fig. 3:**
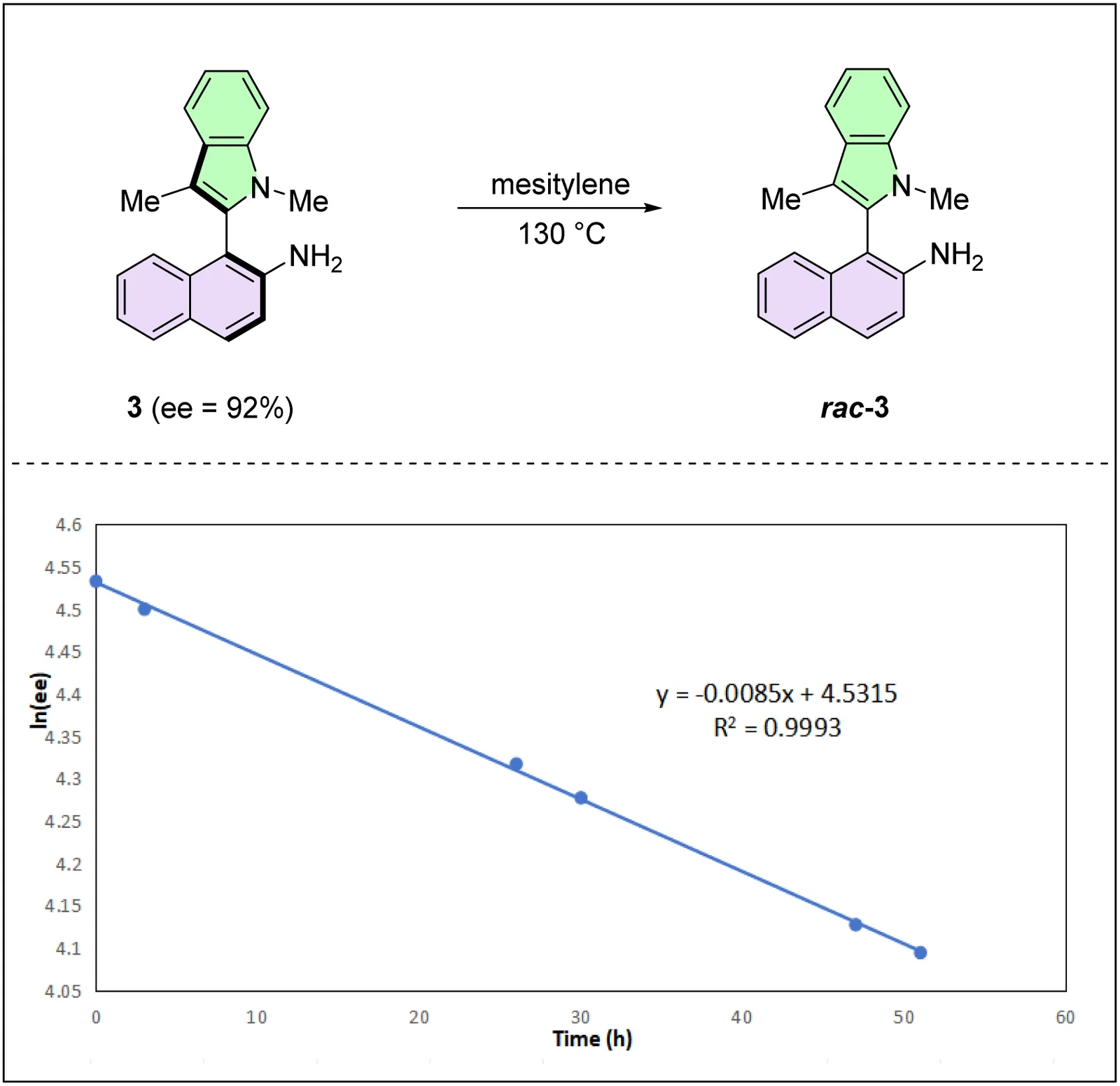
First-order kinetic plot for the racemization of 3 in mesitylene at 130 °C.

With the optimized conditions established (entry 15, Table 1), the substrate scope was next investigated (Fig. 2). A diverse group of substituted β-naphthyl azides was examined. Both electron-donating and electron-withdrawing groups at the C4, C6, or C7 positions were well tolerated, delivering the corresponding C2-arylindoles 4–12 in good isolated yields with high enantioselectivities. In contrast, the C8-methyl β-naphthyl azide furnished product 13 in only 30% yield with a markedly reduced ee of 10%, and the C3-methyl substrate failed to afford any desired product (14). The low yield of 13 is likely attributable to steric hindrance imposed by the C8-methyl group, which may interfere with the key axial bond-forming event. The failure of the reaction to produce 14 is consistent with our previous computational studies of the reaction mechanism. Specifically, the substitution pattern of the azidonaphthalene substrate disfavours the rearomatization step *via* proton transfer from the dearomatized intermediate, thereby suppressing formation of the desired product 14. The reaction also displayed good compatibility with various indole partners: indoles bearing substituents at C5, C6, or C7 generally provided coupling products 16–20 in moderate to good yields with high ee. Varying both the β-naphthyl azide and N–Me indole substrates consistently delivered C2-arylindoles 21–24 with excellent enantioselectivity. The moderate isolated yields of the axially chiral indole products are likely due to side-product formation. In particular, partial decomposition of azidonaphthalene to naphthylamine was observed, along with the formation of BINAMs. The absolute configuration of 9 was assigned by X-ray crystallographic analysis, and those of the remaining products were determined by analogy. To evaluate configurational stability, racemization studies were conducted by heating a solution of 3 in mesitylene at 130 °C and monitoring the ee over time by chiral HPLC (see the SI for details). The natural logarithm of the ee values decreased linearly with time, consistent with first-order racemization kinetics (Fig. 3). From the slope of the linear fit (*k*_rac_ = 0.0085 h^−1^ at 130 °C), the rotational barrier (Δ*G*^‡^) was calculated to be 34.8 kcal mol^−1^ using the Eyring equation, corresponding to a racemization half-life of 5.48 × 10^4^ years at 25 °C, indicating that the C2-arylindoles possess sufficient configurational stability for downstream synthetic manipulations and potential applications. Accordingly, the axially chiral 2-arylindole products were further transformed into thiourea and phosphine ligands, and their utility in asymmetric catalysis was demonstrated (see the SI for details).

**Fig. 4:**
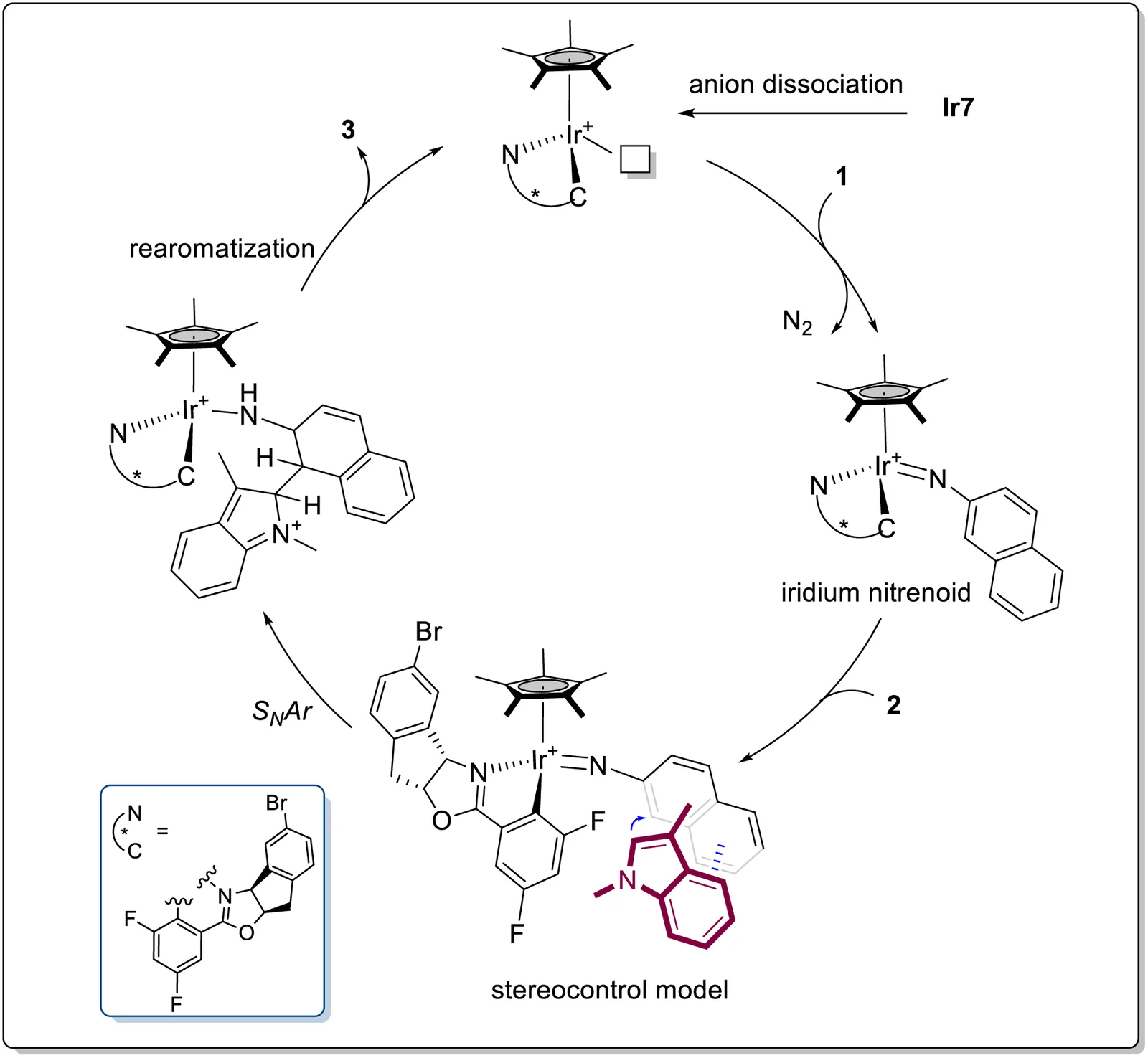
Proposed catalytic cycle.

On the basis of our previous theoretical studies,^47^ we propose the catalytic cycle depicted in Fig. 4. The chiral precatalyst Ir7 undergoes HFIP-promoted chloride dissociation to generate the unsaturated 16-electron active species. Coordination of the azidonaphthalene substrate then triggers irreversible N_2_ extrusion, affording the key iridium nitrenoid intermediate. Subsequent front-side coordination of 2 to the naphthyl moiety of the nitrene, with the nitrogen atom oriented toward the chiral ligand, gives a dearomatized intermediate. Finally, intramolecular proton transfer enables rearomatization to furnish 3 and regenerate the active catalyst.

## Conclusions

In conclusion, we have developed a straightforward atroposelective C2-arylation of indoles with β-azidonaphthalenes through an iridium nitrenoid-promoted arene C–H functionalization. This protocol furnishes configurationally stable C2-arylindole atropisomers in generally good yields and with excellent enantioselectivities under mild reaction conditions. Product derivatization allows for rapid access to axially chiral thiourea and phosphine scaffolds that function as useful organocatalysts and ligands in asymmetric catalysis. Mechanistically, the C2 arylation of indoles proceeds through a novel nucleophilic aromatic substitution (S_N_Ar). By expanding the range of nucleophiles and aryl azides that engage through metal-nitrenoid activation, together with the development of tunable and efficient chiral oxazoline-chelating metal complexes, additional S_N_Ar reactions are forthcoming, which will open new opportunities in asymmetric catalysis and medicinal chemistry.

## Author contributions

F. M. designed and carried out the experiments and prepared the SI. W. Z. and Y. C. participated in the synthesis of partial substrates. F. M., L. Q. and Y. L. initiated the project and wrote the paper. Y. L. supervised the project overall. All authors discussed the results and commented on the manuscript.

## Conflicts of interest

There are no conflicts to declare.

## Supplementary Material

SC-017-D6SC04377A-s001

SC-017-D6SC04377A-s002

## Data Availability

CCDC 2488280 contains the supplementary crystallographic data for this paper.^55^ Supplementary information (SI): general comments, general procedure, analytic data, and NMR spectra. See DOI: https://doi.org/10.1039/d6sc04377a.
