# Supplementary material for: Iridium nitrenoid-catalyzed atroposelective C2 arylation of indoles
Source: Chem Sci. 2026 Jul 2. Online ahead of print. doi: 10.1039/d6sc04377a (PMC13356844; doi:10.1039/d6sc04377a)
Supplement: SC-OLF-D6SC04377A-s001 [file SC-OLF-D6SC04377A-s001.pdf]

Supplementary Information (SI) for Chemical Science.
This journal is © The Royal Society of Chemistry 2026

Electronic Supplementary Information

Iridium-Nitrenoid Catalyzed Atroposelective C2 Arylation of Indoles

Fen Mi, Wenrui Zheng, Yuhan Cao, Liang-Wen Qi,* and Yixin Lu*

Table of Contents

1. General information S1
2. General procedure for the preparation of Iridium catalysts S2
3. General procedure for the preparation of substrates...S9
4. General procedure for the atroposelective C2 arylation of indoles.......................S12
5. The transformation and application...S41
6. Racemization Experiment and Calculation of Half-life of **3**................................S52
7. X-ray data...S54
8. Copies of NMR spectra..S56
9. Supplementary references..S103

1. General information

Chemicals were purchased from commercial suppliers and used without further purification unless otherwise stated. Analytical thin layer chromatography (TLC) was performed on silica gel plates (60F -254) using UV light (254 nm). Flash column chromatography was performed using silica gel (300-400 mesh). ^{1}H and ^{13}C NMR spectra were recorded on a Bruker 400 MHz, 500 MHz or 600 MHz spectrometer in $CDCl_3$, CD_2Cl_2 or DMSO-d_6. The chemical shifts are expressed in ppm and coupling constants are given in Hz. Data for ^{1}H NMR are recorded as follows: chemical shift (δ, ppm), multiplicity (s = singlet; d = doublet; t = triplet; q = quartet; m = multiplet or overlap of non equivalent resonances; br = broad), coupling constant (Hz), integration. Data for ^{13}C NMR are reported in terms of chemical shift (δ, ppm). Optical rotations were measured using an Anton Paar MCP-100 polarimeter. The enantiomeric excess values were determined by chiral HPLC analysis on a chiral stationary phase using Daicel CHIRALCEL and CHIRALPAK columns eluting with *n*-hexane/isopropanol. High resolution mass spectroscopy (HRMS) analyses were performed by the MS service at the chemistry department, National University of Singapore on a Thermo Scientific LCQ Fleet (APCI) and a Finnigan/MAT 95XL-T spectrometer.

2. General procedure for the preparation of Iridium catalysts

Ir1-Ir3, **Ir6** and **Ir12** were prepared according to the literature and the NMR spectra are in agreement with the published data[1, 2]. $[Cp^{R}IrCl_2]_2$ (R = Bn, or ^{i}Pr) were prepared as described in the literature[3]. Iridium(III) catalysts **Ir4**, **Ir5** and **Ir7-Ir11** were synthesized and characterized according to the procedures detailed below.

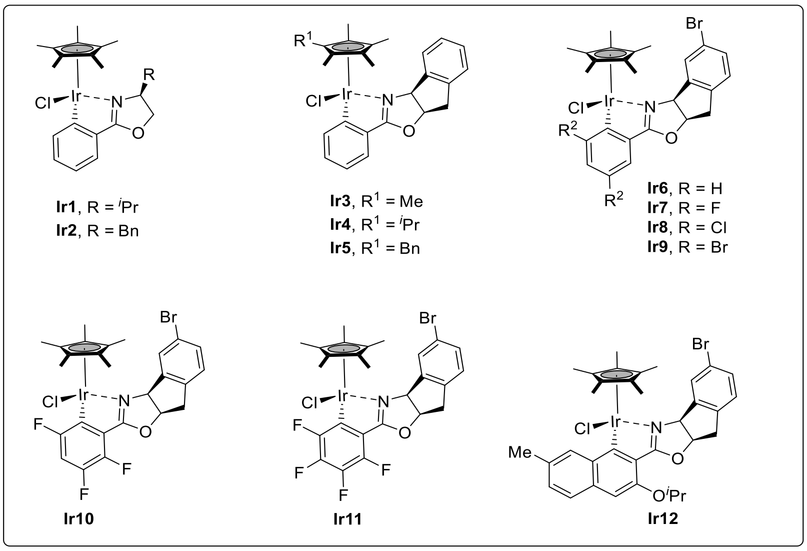

2.1 Synthesis of chiral oxazoline ligands

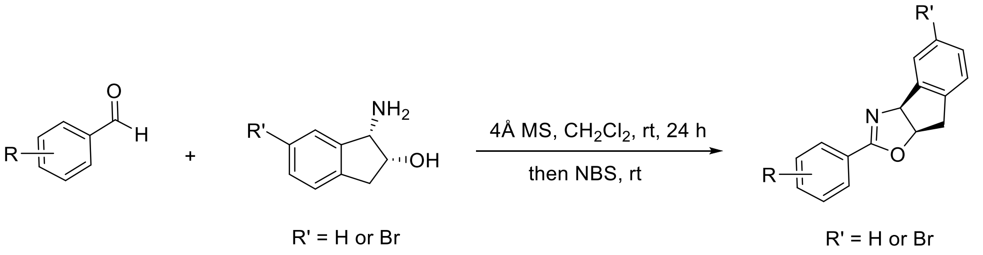

According to the literature[2], 4 Å molecular sieves (1.0 g for 3 mmol substrate) was added to a flame-dried 25 mL Schlenk tube, and then activated under reduced pressure with a heat gun for 15 min. After cooling, (1*S*,2*R*)-1-amino-6-bromo-2,3-dihydro-1*H*-inden-2-ol (1.0 equiv.), substituted aryl aldehyde (1.0 equiv.) and CH_2Cl_2 (10 mL for 3 mmol substrate) were added under N_2 atmosphere. The resulting mixture was stirred for 24 hours at room temperature, and then NBS (1.5 equiv.) was added in one portion. The resulting orange solution was further

stirred at room temperature until the above intermediate was completely consumed (monitored by TLC). The reaction mixture was filtered and washed with 10% $Na_2S_2O_3$ aqueous solution, brine, and dried over Na_2SO_4. After evaporation of the solvent, the residue was subjected to column chromatography on silica gel eluting with *n*-hexane/EA (20:1) to yield corresponding chiral oxazoline ligands.

(3a*S*,8a*R*)-5-bromo-2-(3,5-difluorophenyl)-3a,8a-dihydro-8*H*-indeno[1,2-d]oxazole

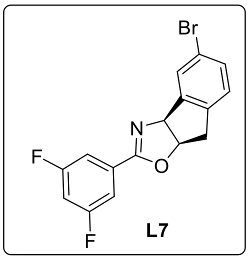

^{1}H NMR (600 MHz, $CDCl_3$) δ 7.65 (s, 1H), 7.52 – 7.31 (m, 3H), 7.12 (d, *J* = 8.1 Hz, 1H), 6.97 – 6.80 (m, 1H), 5.69 (d, *J* = 7.9 Hz, 1H), 5.48 (t, *J* = 7.3 Hz, 1H), 3.43 (dd, *J* = 18.0, 6.9 Hz, 1H), 3.29 (d, *J* = 18.1 Hz, 1H). ^{13}C NMR (126 MHz, $CDCl_3$) δ 162.7 (d, *J* = 249.1 Hz), 162.6 (d, *J* = 249.2 Hz), 162.4, 143.7, 138.5, 131.8, 130.6, 128.7, 126.8, 121.2, 111.5 (d, *J* = 27.4 Hz), 111.5 (d, *J* = 14.1 Hz), 106.8, 83.9, 76.7, 39.3. ^{19}F NMR (471 MHz, $CDCl_3$) δ -108.9. HRMS (ESI) calcd for $[M+H]^+$ $C_{16}H_{11}BrF_2NO$, m/z: 349.9987, found: 349.9988. $[\alpha]_D^{25}$ = -87.037 (*c* = 0.540, $CHCl_3$).

(3a*S*,8a*R*)-5-bromo-2-(2,3,5-trifluorophenyl)-3a,8a-dihydro-8*H*-indeno[1,2-d]oxazole

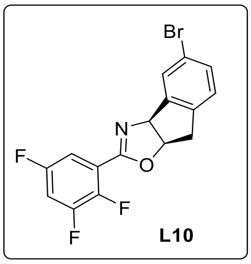

^{1}H NMR (500 MHz, $CDCl_3$) δ 7.70 (d, *J* = 1.8 Hz, 1H), 7.42 – 7.34 (m, 2H), 7.14 (d, *J* = 8.1 Hz, 1H), 7.08 – 6.99 (m, 1H), 5.75 (d, *J* = 7.9 Hz, 1H), 5.55 – 5.46 (m, 1H), 3.51 – 3.40 (m, 1H), 3.36 – 3.23 (m, 1H). ^{13}C NMR (126 MHz, $CDCl_3$) δ 159.7, 157.9, 155.9, 151.9, 149.9, 143.5, 138.5, 131.9, 128.8, 126.8, 121.3, 112.2, 108.3, 83.6, 76.6, 39.3. ^{19}F NMR (565 MHz, $CDCl_3$) δ -114.6, -131.7, -139.2. HRMS (ESI) calcd for $[M+H]^+$ $C_{16}H_{10}BrF_3NO$, m/z: 367.9892, found: 367.9891. $[\alpha]_D^{25}$ = -194.000 (*c* = 0.450, $CHCl_3$).

(3a*S*,8a*R*)-5-bromo-2-(2,3,4,5-tetrafluorophenyl)-3a,8a-dihydro-8*H*-indeno[1,2-d]oxazole

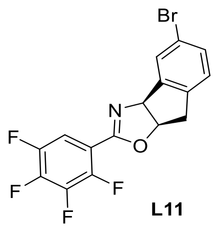

L11

^{1}H NMR (500 MHz, $CDCl_3$) δ 7.68 (d, *J* = 1.9 Hz, 1H), 7.56 – 7.49 (m, 1H), 7.39 (dd, *J* = 8.1, 1.9 Hz, 1H), 7.14 (d, *J* = 8.1 Hz, 1H), 5.73 (d, *J* = 7.9 Hz, 1H), 5.52 – 5.46 (m, 1H), 3.45 (dd, *J* = 18.2, 6.8 Hz, 1H), 3.34 – 3.28 (m, 1H). ^{13}C NMR (126 MHz, $CDCl_3$) δ 159.1, 147.4, 145.4, 143.4, 142.3, 140.2, 138.4, 131.9, 128.8, 126.9, 121.3, 112.3, 112.1, 83.7, 76.7, 39.2. ^{19}F NMR (471 MHz, $CDCl_3$) δ -134.2, -138.3, -149.8, -154.0. HRMS (ESI) calcd for $[M+H]^+$ $C_{16}H_9BrF_4NO$, m/z: 385.9798, found: 385.9796. $[\alpha]_D^{25}$ = -198.000 (*c* = 0.100, $CHCl_3$).

2.2 Synthesis of chiral iridium(III) catalysts

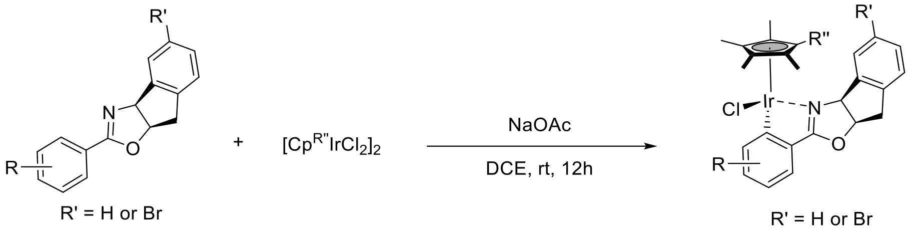

According to the literature[2], a glass vial was charged with $[Cp^RIrCl_2]_2$ (1 equiv.), ligand (2.1 equiv.), NaOAc (5 equiv.) and DCE (5 mL for 1 mmol Ir catalyst). The solution was allowed to stir at room temperature for 12 h. The reaction mixture was filtered through a pad of Celite and the filtrate was concentrated *in vacuo*. The resulting residue was purified by washing with Et_2O (or *n*-hexane/CH_2Cl_2) to yield iridium catalysts **Ir4**, **Ir5 and Ir7-Ir11**.

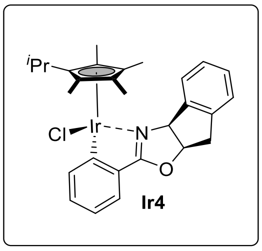

^{1}H NMR (600 MHz, $CDCl_3$) δ 7.70 (dt, *J* = 7.6, 0.8 Hz, 1H), 7.65 – 7.61 (m, 1H), 7.38 – 7.34 (m, 1H), 7.29 – 7.24 (m, 2H), 7.23 – 7.20 (m, 1H), 7.16 (td, *J* = 7.4, 1.5 Hz, 1H), 6.92 (td, *J* = 7.4, 1.1 Hz, 1H), 5.80 (dt, *J* = 7.7, 3.9 Hz, 1H), 5.65 (d, *J* = 7.6 Hz, 1H), 3.53 – 3.48 (m, 2H), 2.53 – 2.47 (m, 1H), 2.08 (s, 3H), 1.95 (s, 3H), 1.84 (d, *J* = 12.3 Hz, 6H), 1.16 (d, *J* = 7.1 Hz, 3H), 1.08 (d, *J* = 7.2 Hz, 3H). ^{13}C NMR (151 MHz, $CDCl_3$) δ 179.7, 164.6, 138.9, 137.9, 135.7 (2C), 135.6, 132.5, 130.0, 129.1, 127.3, 126.3, 124.6, 96.8, 93.4, 90.8, 88.3, 85.3, 80.9, 71.3, 38.5, 26.1 (2C), 21.3, 10.3, 10.1 (2C), 10.0. HRMS (ESI) calcd for [M-Cl] $C_{28}H_{31}IrNO$, m/z: 590.2031, found: 590.2032. $[\alpha]_D^{25}$ = -25.714 (*c* = 0.070, $CHCl_3$).

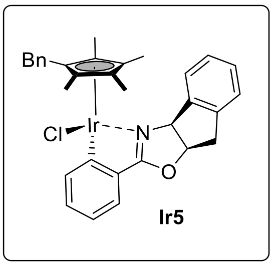

^{1}H NMR (600 MHz, $CDCl_3$) δ 7.77 (d, *J* = 7.6 Hz, 1H), 7.67 – 7.64 (m, 1H), 7.39 (dd, *J* = 7.6, 1.4 Hz, 1H), 7.33 – 7.27 (m, 4H), 7.25 – 7.18 (m, 3H), 7.18 – 7.14 (m, 2H), 6.96 (td, *J* = 7.4, 1.1 Hz, 1H), 5.84 – 5.77 (m, 1H), 5.63 (d, *J* = 7.6 Hz, 1H), 3.64 – 3.54 (m, 2H), 3.52 (d, *J* = 3.9 Hz, 2H), 1.90 (d, *J* = 16.8 Hz, 6H), 1.86 (d, *J* = 2.0 Hz, 6H). ^{13}C NMR (151 MHz, $CDCl_3$) δ 179.7, 164.0, 139.0, 138.8, 137.9, 135.6 (2C), 132.5, 130.1, 128.6, 128.1, 127.2 (3C), 126.5, 126.4, 90.2, 89.2, 88.6, 88.4, 88.0, 85.6, 71.2, 38.5, 30.6, 9.8, 9.6, 9.5 (2C). HRMS (ESI) calcd for [M-Cl] $C_{32}H_{31}IrNO$, m/z: 638.2031, found: 638.2029. $[\alpha]_D^{25}$ = -135.714 (*c* = 0.070, $CHCl_3$).

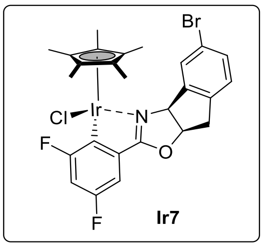

^{1}H NMR (600 MHz, $CDCl_3$) δ 7.71 (d, *J* = 1.9 Hz, 1H), 7.39 – 7.34 (m, 1H), 7.06 (d, *J* = 8.1 Hz, 1H), 6.95 (dd, *J* = 8.0, 2.4 Hz, 1H), 6.74 – 6.68 (m, 1H), 5.83 – 5.78 (m, 1H), 5.63 (dd, *J* = 7.7, 0.8 Hz, 1H), 3.48 – 3.42 (m, 1H), 3.38 (dt, *J* = 18.2, 1.2 Hz, 1H), 1.86 (s, 15H). ^{13}C NMR (151 MHz, $CDCl_3$) δ 178.9, 165.4 (d, *J* = 238.8 Hz), 159.4 (d, *J* = 240.5 Hz) , 141.3 (d, *J* = 3.0 Hz), 139.3, 137.7, 132.2 (d, *J* = 19.8), 131.9 (dd, *J* = 18.6, 9.9 Hz), 130.7, 126.1 (dd, *J* = 24.5, 4.8 Hz), 120.9, 109.4, 107.0, 88.6, 88.3 (5C), 70.6, 38.3, 9.6 (5C). ^{19}F NMR (565

MHz, $CDCl_3$) δ -92.2, -119.9. HRMS (ESI) calcd for [M-Cl] $C_{26}H_{24}BrF_2IrNO$, m/z: 676.0618, found: 676.0616. $[\alpha]_D^{25}$ = 223.333 (*c* = 0.030, $CHCl_3$).

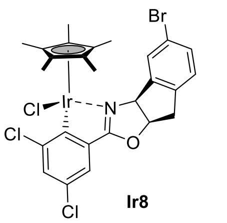

Ir8

^1H NMR (600 MHz, $CDCl_3$) δ 7.73 (d, *J* = 1.9 Hz, 1H), 7.37 (dd, *J* = 8.0, 1.9 Hz, 1H), 7.31 (d, *J* = 2.1 Hz, 1H), 7.23 (d, *J* = 2.1 Hz, 1H), 7.05 (d, *J* = 8.1 Hz, 1H), 5.83 – 5.76 (m, 1H), 5.70 (d, *J* = 7.7 Hz, 1H), 3.47 (dd, *J* = 18.4, 6.6 Hz, 1H), 3.38 (d, *J* = 18.3 Hz, 1H), 1.84 (s, 15H). ^{13}C NMR (151 MHz, $CDCl_3$) δ 178.4, 160.0, 143.1, 139.1, 137.5, 132.8, 132.5, 132.4, 130.8, 128.1, 126.0, 125.0, 121.1, 88.7 (5C), 87.9, 70.5, 38.5, 9.7 (5C). HRMS (ESI) calcd for [M-Cl] $C_{26}H_{24}BrCl_2IrNO$, m/z: 708.0026, found: 708.0020. $[\alpha]_D^{25}$ = 18.889 (*c* = 0.090, $CHCl_3$).

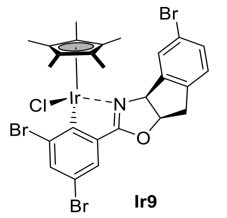

Ir9

^1H NMR (600 MHz, $CDCl_3$) δ 7.74 (d, *J* = 1.9 Hz, 1H), 7.63 (d, *J* = 2.0 Hz, 1H), 7.40 (d, *J* = 2.1 Hz, 1H), 7.36 (dd, *J* = 8.1, 1.8 Hz, 1H), 7.03 (d, *J* = 8.1 Hz, 1H), 5.82 – 5.76 (m, 1H), 5.71 (d, *J* = 7.7 Hz, 1H), 3.45 (dd, *J* = 18.3, 6.7 Hz, 1H), 3.35 (d, *J* = 18.3 Hz, 1H), 1.83 (s, 15H). ^{13}C NMR (151 MHz, $CDCl_3$) δ 178.0, 163.6, 139.1, 138.4, 137.4, 134.6, 133.4, 132.5, 130.9, 128.1, 126.1, 121.1, 115.4, 88.7 (5C), 87.8, 70.4, 38.6, 9.8 (5C). HRMS (ESI) calcd for [M-Cl] $C_{26}H_{24}Br_3IrNO$, m/z: 795.9005, found: 795.9008. $[\alpha]_D^{25}$ = 54.000 (*c* = 0.050, $CHCl_3$).

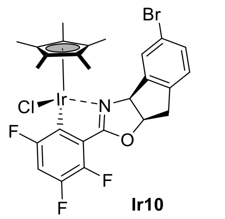

^{1}H NMR (600 MHz, $CDCl_3$) δ 7.71 (d, *J* = 1.9 Hz, 1H), 7.41 – 7.37 (m, 1H), 7.08 (d, *J* = 8.2 Hz, 1H), 6.85 (dt, *J* = 10.8, 6.2 Hz, 1H), 5.91 – 5.84 (m, 1H), 5.60 (d, *J* = 7.8 Hz, 1H), 3.49 – 3.46 (m, 2H), 1.87 (d, *J* = 0.5 Hz, 15H). ^{13}C NMR (151 MHz, $CDCl_3$) δ 177.8, 160.4, 147.1, 145.4, 142.5, 139.0, 137.8, 132.4, 130.7, 126.3, 121.0, 119.7, 108.6, 89.0, 88.8 (5C), 70.0, 38.4, 9.6 (5C). ^{19}F NMR (565 MHz, $CDCl_3$) δ -97.0, -143.8, -145.5. HRMS (ESI) calcd for [M-Cl] $C_{26}H_{23}BrF_3IrNO$, m/z: 694.0524, found: 694.0523. $[\alpha]_D^{25}$ = 7.414 (*c* = 0.580, $CHCl_3$).

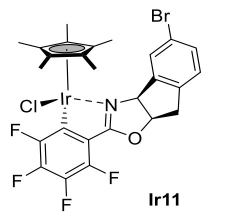

^{1}H NMR (600 MHz, $CDCl_3$) δ 7.68 (d, *J* = 1.9 Hz, 1H), 7.42 – 7.37 (m, 1H), 7.12 – 7.07 (m, 1H), 5.87 (dt, *J* = 7.8, 3.9 Hz, 1H), 5.59 (d, *J* = 7.7 Hz, 1H), 3.47 (d, *J* = 3.7 Hz, 2H), 1.88 (s, 15H). ^{13}C NMR (151 MHz, $CDCl_3$) δ 177.5, 148.1, 147.6, 142.3, 142.0, 138.9, 137.7, 132.5, 130.7, 126.3, 121.0, 113.2, 89.0, 89.0 (5C), 69.9, 38.4, 9.6 (5C). ^{19}F NMR (565 MHz, $CDCl_3$) δ -120.1, -140.3, -148.3, -163.9. HRMS (ESI) calcd for [M-Cl] $C_{26}H_{22}BrF_4IrNO$, m/z: 712.0430, found: 712.0432. $[\alpha]_D^{25}$ = 11.667 (*c* = 0.180, $CHCl_3$).

3. General procedure for the preparation of substrates

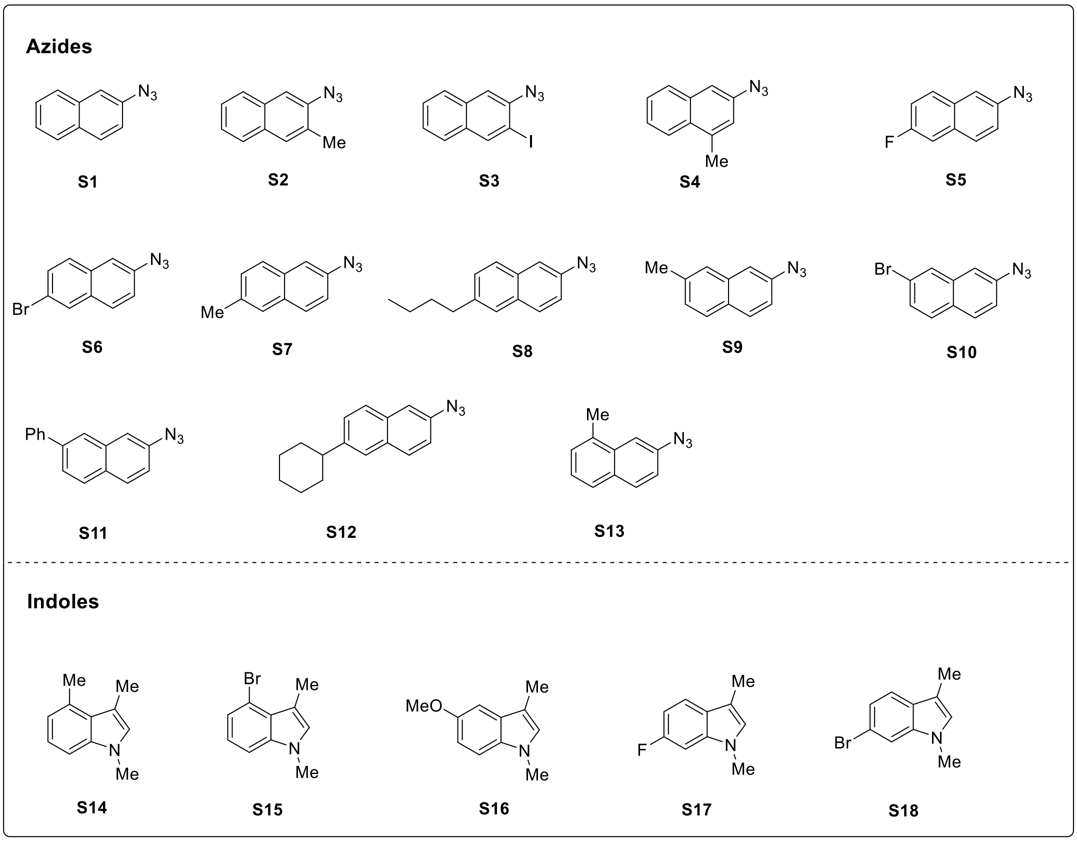

Azides **S1–S10** were prepared according to the reported procedures[2]. Indoles **S14–S16** were synthesized following literature methods[4], while **S17** and **S18** were obtained according to references[5,6]. Azides **S11–S13** were synthesized and characterized as described below.

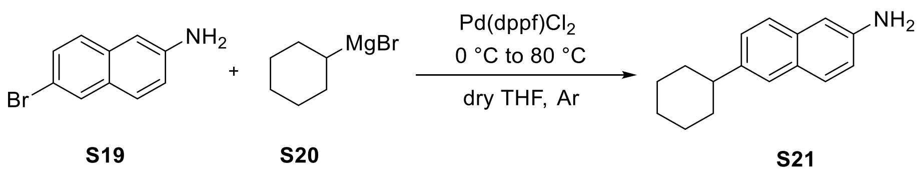

The 6-bromonaphthylamine **S19** (1.0 equiv) and Pd(dppf)Cl_2 (5 mol%) were dissolved in anhydrous THF. Cyclohexylmagnesium bromide **S20** (1.0 M in THF, 4.0 equiv) was added dropwise at 0 °C. The mixture was then heated at 80 °C and stirred overnight. After completion of the reaction (TLC), the reaction mixture was cooled to room temperature and

carefully quenched with saturated NH_4Cl solution. The resulting mixture was diluted with EtOAc, and the layers were separated. The aqueous phase was extracted with EtOAc (×2). The combined organic extracts were dried over anhydrous Na_2SO_4, filtered, and concentrated under reduced pressure. The crude residue was purified by column chromatography to afford the desired coupling product.

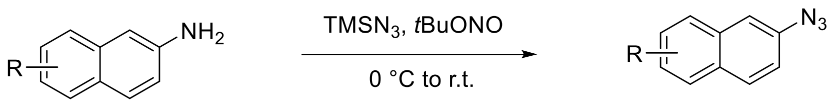

S11 or S12

The corresponding amine was dissolved in anhydrous MeCN and the solution was cooled to 0 °C. *t*-BuONO (1.5 equiv) was added dropwise, followed by the addition of $TMSN_3$ (1.0 equiv). The reaction mixture was then allowed to warm to room temperature and stirred until completion, as monitored by TLC. The solvent was removed under reduced pressure, and the crude residue was purified by column chromatography, eluting with hexane, to afford the desired product.

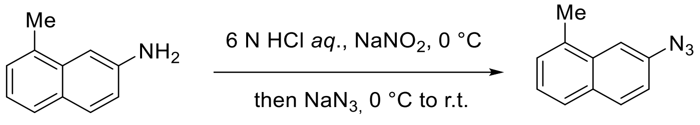

S22 **S13**

According to the literature[2], to a stirred solution of the substituted arylamine (3.0 mmol) in aqueous HCl (6 N, 5 mL, 10 equiv.) at 0 °C was added a solution of $NaNO_2$ (0.31 g, 4.5 mmol, 1.5 equiv.) in water (1.0 mL) dropwise. After stirring for 30 min at 0 °C, a solution of NaN_3 (0.29 g, 4.5 mmol, 1.5 equiv.) in water (1.0 mL) was added dropwise at the same temperature. The resulting reaction mixture was stirred at 0 °C for 2 h and then extracted with EtOAc (×2). The combined organic layers were washed with water and brine, dried over Na_2SO_4, and concentrated in vacuo (keeping the bath temperature below 35 °C). The residue was briefly

purified by flash chromatography on silica gel, eluting with *n*-hexane, to afford the corresponding azide products.

6-cyclohexylnaphthalen-2-amine (S21)

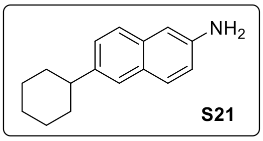

^{1}H NMR (400 MHz, $CDCl_3$) δ 7.64 (d, J = 8.4 Hz, 1H), 7.56 (d, J = 8.3 Hz, 1H), 7.52 (s, 1H), 7.34 – 7.27 (m, 1H), 6.99 (d, J = 2.3 Hz, 1H), 6.95 (dd, J = 8.4, 2.3 Hz, 1H), 3.77 (s, 2H), 2.70 – 2.54 (m, 1H), 2.04 – 1.77 (m, 5H), 1.58 – 1.28 (m, 5H). ^{13}C NMR (101 MHz, $CDCl_3$) δ 143.4, 142.2, 133.4, 128.8, 128.2, 126.6, 125.7, 124.4, 118.2, 108.6, 44.4, 34.5 (2C), 27.0 (2C), 26.2. HRMS (ESI) calcd for $[M+H]^+$ $C_{16}H_{20}N$, m/z: 226.159, found: 226.1589.

2-azido-7-phenylnaphthalene (S11)

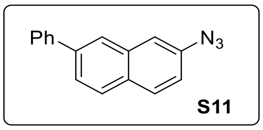

^{1}H NMR (400 MHz, $CDCl_3$) δ 7.96 (s, 1H), 7.87 (dd, J = 12.0, 8.7 Hz, 2H), 7.75 – 7.67 (m, 3H), 7.50 (t, J = 7.5 Hz, 3H), 7.40 (t, J = 7.3 Hz, 1H), 7.17 (dd, J = 8.7, 2.2 Hz, 1H). ^{13}C NMR (101 MHz, $CDCl_3$) δ 140.7, 139.7, 137.9, 134.3, 130.2, 129.6, 128.9 (2C), 128.3, 127.6, 127.4 (2C), 125.2, 124.8, 118.8, 116.0.

2-azido-6-cyclohexylnaphthalene (S12)

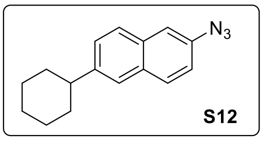

^{1}H NMR (600 MHz, $CDCl_3$) δ 7.77 (d, *J* = 8.8 Hz, 1H), 7.69 (d, *J* = 8.5 Hz, 1H), 7.60 (d, *J* = 1.7 Hz, 1H), 7.43 – 7.38 (m, 2H), 7.13 (dd, *J* = 8.7, 2.3 Hz, 1H), 2.69 – 2.63 (m, 1H), 2.00 – 1.95 (m, 2H), 1.93 – 1.87 (m, 2H), 1.82 – 1.78 (m, 1H), 1.56 – 1.50 (m, 2H), 1.49 – 1.41 (m, 2H), 1.36 – 1.31 (m, 1H). ^{13}C NMR (151 MHz, $CDCl_3$) δ 145.2, 136.6, 132.6, 131.3, 129.6, 127.4, 126.8, 124.5, 118.6, 115.5, 44.5, 34.4 (2C), 26.9 (2C), 26.2.

7-azido-1-methylnaphthalene (S13)

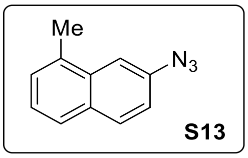

^{1}H NMR (400 MHz, $CDCl_3$) δ 7.84 (d, *J* = 8.7 Hz, 1H), 7.68 (dd, *J* = 5.8, 3.6 Hz, 1H), 7.56 (d, *J* = 2.3 Hz, 1H), 7.36 – 7.31 (m, 2H), 7.18 (dd, *J* = 8.7, 2.2 Hz, 1H), 2.66 (s, 3H). ^{13}C NMR (101 MHz, $CDCl_3$) δ 137.3, 133.4, 133.2, 131.1, 130.5, 127.6, 126.2, 125.1, 118.2, 112.5, 19.3.

4. General procedure for the atroposelective C2 arylation of indoles

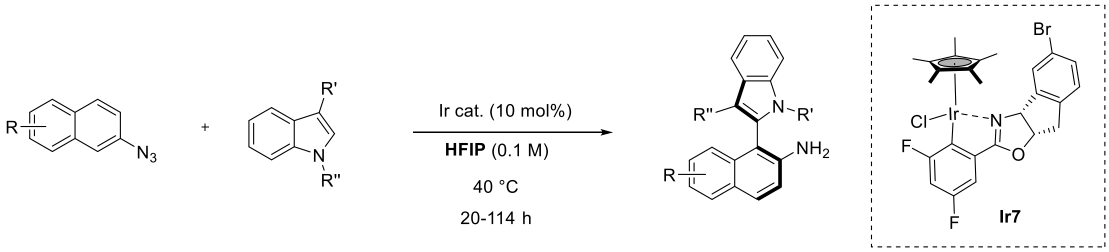

Without protection precaution from air and moisture, a mixture of the azide compound (1.0 equiv) and iridium catalyst **Ir7** (10 mol%) was dissolved in HFIP (0.1 M) and stirred at 40 °C for 2 h. Subsequently, indole (2.0 equiv) was added to the reaction mixture, and the stirring was continued until the complete consumption of azide (monitored by TLC). The mixture was then quenched by the addition of saturated aqueous $NaHCO_3$ solution, extracted with EtOAc. The combined organic phases were washed with brine and dried over anhydrous Na_2SO_4.

After evaporation of the solvent, the residue was subjected to column chromatography on silica gel eluting with *n*-hexane/EtOAc to afford corresponding product.

1-(1,3-dimethyl-1*H*-indol-2-yl)naphthalen-2-amine (3)

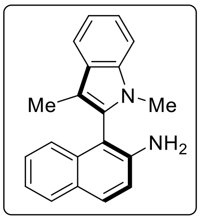

3 was obtained in 53% yield with 92% ee as an off-white solid. ^{1}H NMR (400 MHz, DMSO-d_6) δ 7.79 – 7.72 (m, 2H), 7.63 – 7.56 (m, 1H), 7.49 – 7.43 (m, 1H), 7.25 – 7.06 (m, 5H), 6.95 – 6.88 (m, 1H), 5.09 (s, 2H), 3.35 (s, 3H), 2.01 (s, 3H). ^{13}C NMR (101 MHz, DMSO-d_6) δ 145.9, 137.2, 134.4, 132.6, 129.8, 128.2, 128.0, 126.7, 126.4, 122.6, 121.1, 120.9, 118.3 (2C), 118.2, 109.6, 108.6, 105.2, 29.7, 9.1. HRMS (ESI) calcd for $[M+H]^+$ $C_{20}H_{19}N_2$, m/z: 287.1543, found: 287.1542. HPLC analysis: HPLC DAICEL CHIRALCEL OD-H, hexane/isopropanol = 99/1, 1.0 mL/min, λ = 229 nm, t_R (minor) = 15.288 min, t_R (major) = 17.429 min, er = 96:4. $[\alpha]_D^{25}$ = -12.500 (*c* = 0.200, $CHCl_3$).

Chiral HPLC spectrum of (*rac*)-**3**

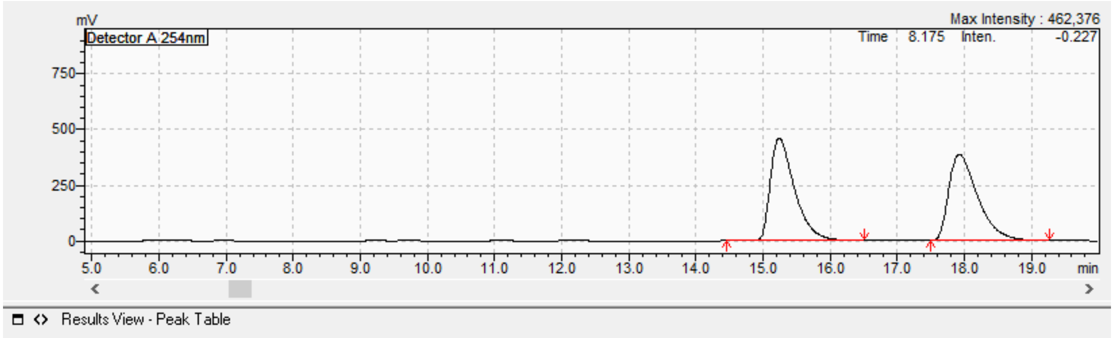

Peak#	Ret. Time	Area	Height	Mark	Conc.	Area%
1	15.241	11571694	460909	M	49.876	49.876
2	17.925	11629338	387833	M	50.124	50.124
Total		23201032	848742		100.000	100.000

Chiral HPLC spectrum of (*S*)-**3**

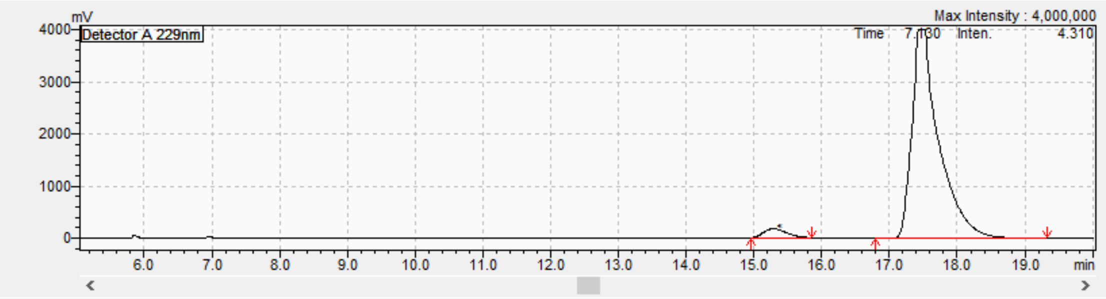

Results View - Peak Table

Peak Table | Compound | Group | Calibration Curve

Peak#	Ret. Time	Area	Height	Mark	Conc.	Area%
1	15.288	4128359	174113	M	3.601	3.601
2	17.429	110528349	3998109	M	96.399	96.399
Total		114656708	4172223		100.000	100.000

1-(1,3-dimethyl-1*H*-indol-2-yl)-7-methylnaphthalen-2-amine (4)

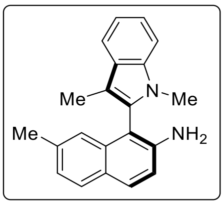

4 was obtained in 64% yield with 91% ee as an off-white solid. ^{1}H NMR (400 MHz, DMSO-d_6) δ 7.70 (d, J = 8.7 Hz, 1H), 7.64 (d, J = 8.2 Hz, 1H), 7.62 – 7.57 (m, 1H), 7.49 – 7.43 (m, 1H), 7.21 – 7.16 (m, 1H), 7.12 – 7.06 (m, 2H), 6.98 (dd, J = 8.3, 1.7 Hz, 1H), 6.76 – 6.66 (m, 1H), 5.00 (s, 2H), 3.34 (s, 3H), 2.23 (s, 3H), 2.01 (s, 3H). ^{13}C NMR (101 MHz, DMSO-d_6) δ 146.0, 137.2, 135.8, 134.6, 132.8, 129.5, 128.2, 128.0, 124.7, 123.3, 121.7, 120.8, 118.3 (2C), 117.2, 109.7, 108.5, 104.8, 29.8, 21.6, 9.2. HRMS (ESI) calcd for $[M+H]^+$ $C_{21}H_{21}N_2$, m/z: 301.1699, found: 301.1698. HPLC analysis: HPLC DAICEL CHIRALCEL OD-H, hexane/isopropanol = 99/1, 1.0 mL/min, λ = 229 nm, t_R (minor) = 14.175 min, t_R (major) = 16.667 min, er = 95.5:4.5. $[\alpha]_D^{25}$ = -10.500 (c = 0.580, $CHCl_3$).

Chiral HPLC spectrum of (*rac*)-**4**

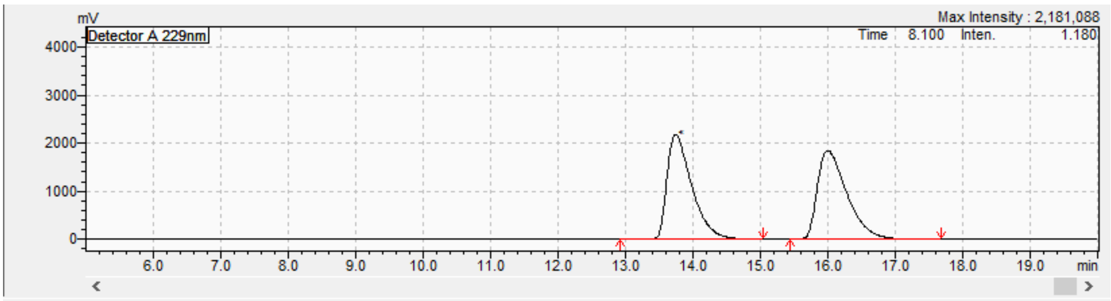

Results View - Peak Table

Peak Table | Compound | Group | Calibration Curve

Peak#	Ret. Time	Area	Height	Mark	Conc.	Area%
1	13.744	55306784	2178782	M	49.782	49.782
2	16.000	55791152	1842387	M	50.218	50.218
Total		111097936	4021169		100.000	100.000

Chiral HPLC spectrum of (*S*)-**4**

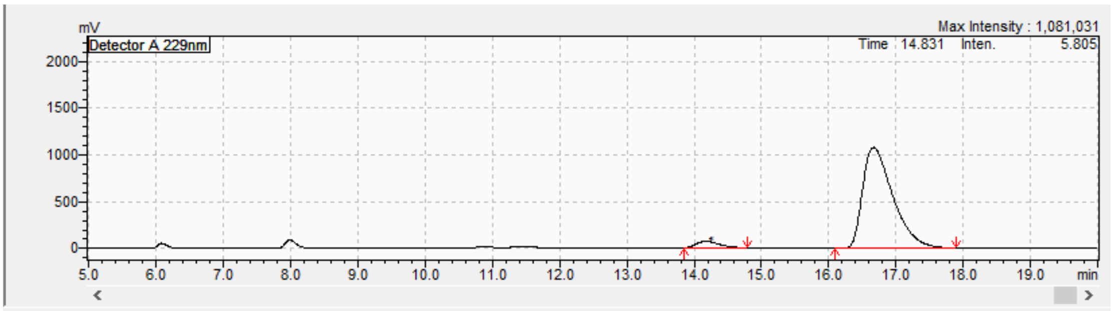

Results View - Peak Table

Peak Table | Compound | Group | Calibration Curve

Peak#	Ret. Time	Area	Height	Mark	Conc.	Area%
1	14.175	1682534	70468	M	4.761	4.761
2	16.667	33655428	1077670	M	95.239	95.239
Total		35337962	1148138		100.000	100.000

1-(1,3-dimethyl-1*H*-indol-2-yl)-7-phenylnaphthalen-2-amine (5)

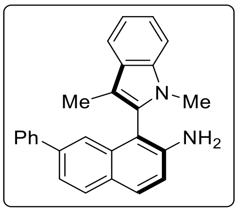

5 was obtained in 51% yield with 91% ee as a yellow solid. ^{1}H NMR (400 MHz, DMSO-d_6) δ 7.87 – 7.79 (m, 2H), 7.63 – 7.59 (m, 1H), 7.49 – 7.42 (m, 4H), 7.37 (dd, *J* = 8.5, 6.9 Hz, 2H), 7.30 – 7.25 (m, 1H), 7.21 – 7.16 (m, 2H), 7.13 – 7.06 (m, 2H), 5.14 (s, 2H), 3.40 (s, 3H), 2.05

(s, 3H). ^{13}C NMR (101 MHz, DMSO-d_6) δ 146.4, 140.9, 138.7, 137.3, 134.7, 132.4, 129.6, 128.9 (3C), 128.2, 127.3, 126.7 (2C) 125.7, 120.9, 120.5, 120.3, 118.4 (3C), 109.72, 108.7, 105.4, 29.8, 9.2. HRMS (ESI) calcd for $[M+H]^+$ $C_{26}H_{23}N_2$, m/z: 363.1856, found: 363.1854. HPLC analysis: HPLC DAICEL CHIRALCEL OD-H, hexane/isopropanol = 99/1, 1.0 mL/min, λ = 229 nm, t_R (minor) = 23.644 min, t_R (major) = 29.018 min, er = 95.5:4.5. $[\alpha]_D^{25}$ = -5.869 (c = 0.130, CH_2Cl_2).

Chiral HPLC spectrum of (*rac*)-**5**

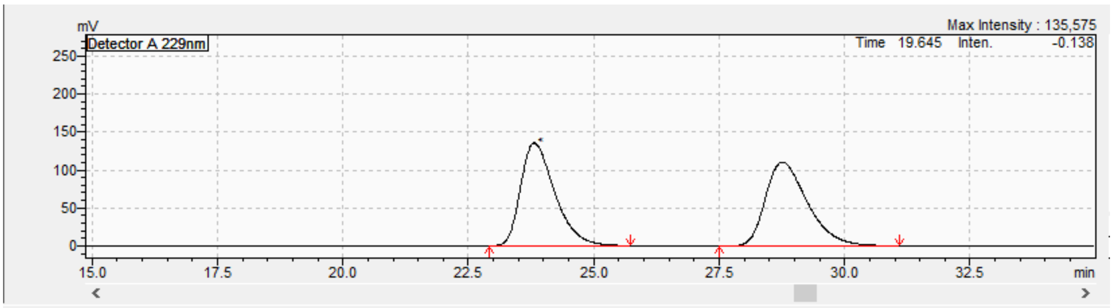

Results View - Peak Table

Peak Table | Compound | Group | Calibration Curve

Peak#	Ret. Time	Area	Height	Mark	Conc.	Area%
1	23.814	6415804	135467	M	49.909	49.909
2	28.758	6439090	110792	M	50.091	50.091
Total		12854893	246259		100.000	100.000

Chiral HPLC spectrum of (*S*)-**5**

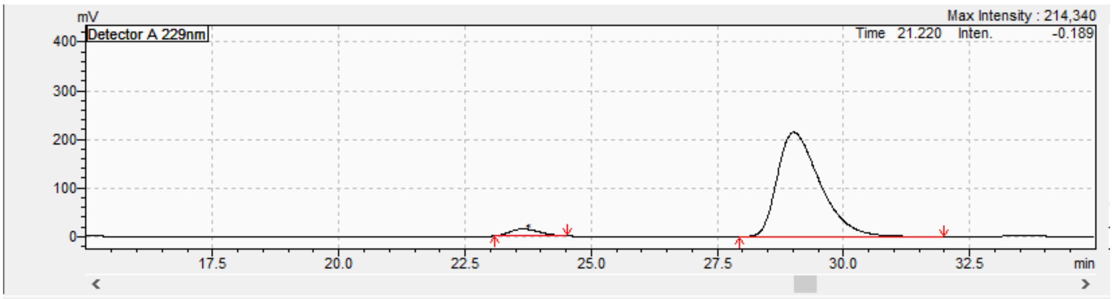

Results View - Peak Table

Peak Table | Compound | Group | Calibration Curve

Peak#	Ret. Time	Area	Height	Mark	Conc.	Area%
1	23.644	589825	14322	M	4.468	4.468
2	29.018	12611411	214568	M	95.532	95.532
Total		13201235	228890		100.000	100.000

7-bromo-1-(1,3-dimethyl-1*H*-indol-2-yl)naphthalen-2-amine (6)

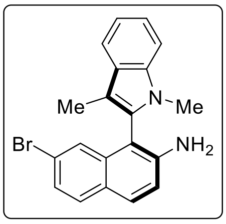

6 was obtained in 50% yield with 85% ee as a yellow-brown oil. ^{1}H NMR (400 MHz, CD_2Cl_2) δ 7.77 (dd, J = 8.8, 0.8 Hz, 1H), 7.69 – 7.61 (m, 2H), 7.43 – 7.38 (m, 1H), 7.35 – 7.23 (m, 3H), 7.21 – 7.14 (m, 1H), 7.11 (d, J = 8.8 Hz, 1H), 3.99 (s, 2H), 3.41 (s, 3H), 2.12 (s, 3H). ^{13}C NMR (101 MHz, CD_2Cl_2) δ 145.8, 138.2, 136.5, 131.7, 130.6, 130.2, 129.0, 126.5, 126.1, 125.8, 121.9 (2C), 119.2 (2C), 118.4, 110.8, 109.8, 107.6, 30.3, 9.2. HRMS (ESI) calcd for [M+H] $C_{20}H_{18}BrN_2$, m/z: 365.0648, found: 365.0649. HPLC analysis: HPLC DAICEL CHIRALCEL OD-H, hexane/isopropanol = 99/1, 1.0 mL/min, λ = 229 nm, t_R (minor) = 17.735 min, t_R (major) = 20.894 min, er = 92.5:7.5. $[\alpha]_D^{25}$ = -5.600 (c = 0.500, $CHCl_3$).

Chiral HPLC spectrum of (*rac*)-**6**

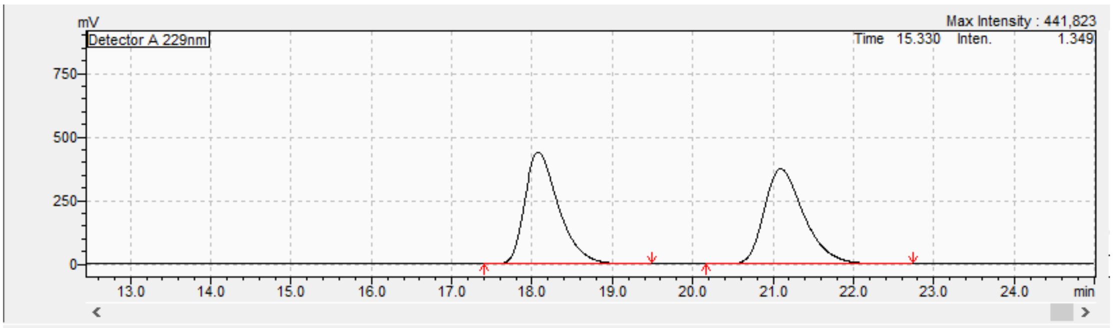

Results View - Peak Table

Peak Table | Compound | Group | Calibration Curve

Peak#	Ret. Time	Area	Height	Mark	Conc.	Area%
1	18.079	12910524	440217	M	49.982	49.982
2	21.094	12919728	374812	M	50.018	50.018
Total		25830252	815029		100.000	100.000

Chiral HPLC spectrum of (*S*)-**6**

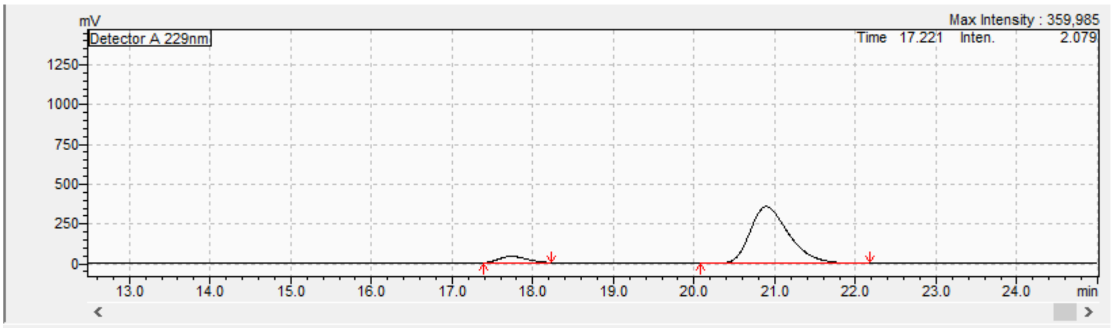

Results View - Peak Table

Peak Table | Compound | Group | Calibration Curve

Peak#	Ret. Time	Area	Height	Mark	Conc.	Area%
1	17.735	982360	40309	M	7.539	7.539
2	20.894	12048180	358458	M	92.461	92.461
Total		13030539	398768		100.000	100.000

1-(1,3-dimethyl-1*H*-indol-2-yl)-4-methylnaphthalen-2-amine (7)

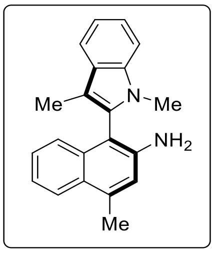

7 was obtained in 53% yield with 91% ee as an off-white solid. ^{1}H NMR (400 MHz, CD_2Cl_2) δ 7.96 – 7.90 (m, 1H), 7.67 – 7.62 (m, 1H), 7.41 – 7.36 (m, 1H), 7.30 – 7.22 (m, 3H), 7.18 – 7.12 (m, 2H), 6.98 (d, *J* = 1.1 Hz, 1H), 3.39 (s, 3H), 2.71 (s, 3H), 2.11 (s, 3H). ^{13}C NMR (101 MHz, CD_2Cl_2) δ 144.4, 138.1, 137.4, 135.4, 132.9, 129.0, 127.5, 127.0, 124.7, 124.7, 122.4, 121.6, 119.0 (2C), 118.9, 110.4, 109.6, 106.8, 30.2, 19.7, 9.2. HRMS (ESI) calcd for $[M+H]^+$ $C_{21}H_{21}N_2$, m/z: 301.1699, found: 301.1698. HPLC analysis: HPLC DAICEL CHIRALCEL OD-H, hexane/isopropanol = 99/1, 1.0 mL/min, λ = 229 nm, t_R (minor) = 13.172min, t_R (major) = 17.534 min, er = 95.5:4.5. $[\alpha]_D^{25}$ = -18.100 (*c* = 1.000, CH_2Cl_2).

Chiral HPLC spectrum of (*rac*)-**7**

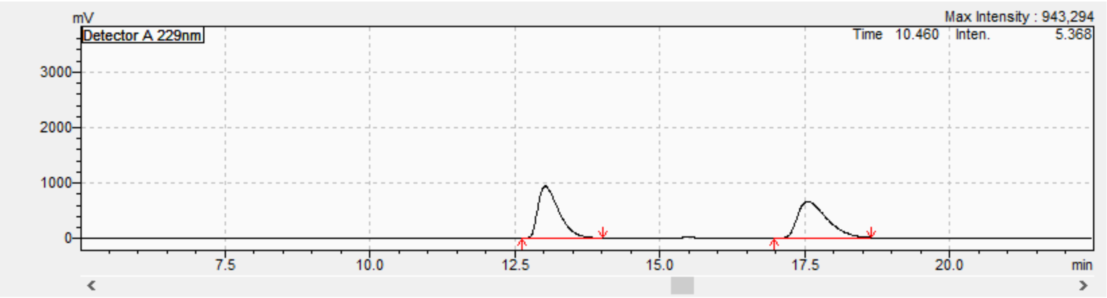

Results View - Peak Table

Peak Table | Compound | Group | Calibration Curve

Peak#	Ret. Time	Area	Height	Mark	Conc.	Area%
1	13.027	23371356	936536	M	50.113	50.113
2	17.555	23266205	659510	M	49.887	49.887
Total		46637560	1596047		100.000	100.000

Chiral HPLC spectrum of (*S*)-**7**

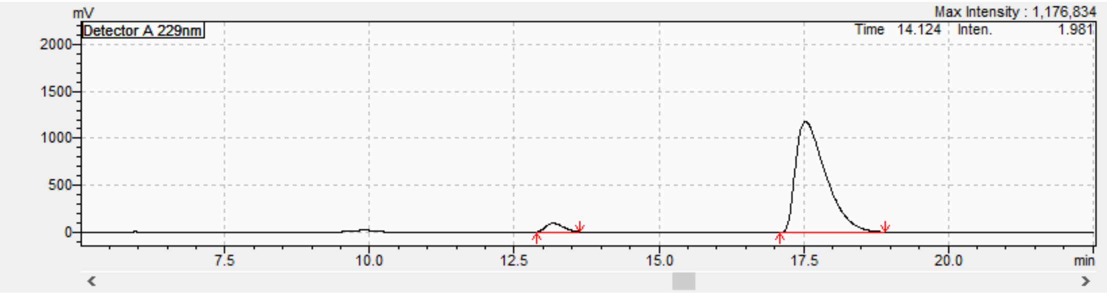

Results View - Peak Table

Peak Table | Compound | Group | Calibration Curve

Peak#	Ret. Time	Area	Height	Mark	Conc.	Area%
1	13.172	1983215	92672	M	4.447	4.447
2	17.534	42615869	1173621	M	95.553	95.553
Total		44599083	1266293		100.000	100.000

1-(1,3-dimethyl-1*H*-indol-2-yl)-6-methylnaphthalen-2-amine (8)

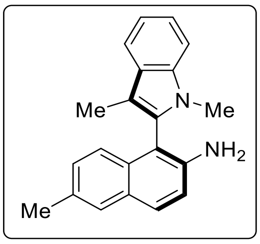

8 was obtained in 70% yield with 86% ee as a yellow-brown oil. ^{1}H NMR (400 MHz, CD_2Cl_2) δ 7.72 (dd, *J* = 8.8, 0.8 Hz, 1H), 7.68 – 7.63 (m, 1H), 7.56 (dt, *J* = 1.9, 1.0 Hz, 1H), 7.41 – 7.37 (m, 1H), 7.28 – 7.23 (m, 1H), 7.18 – 7.12 (m, 2H), 7.09 – 7.03 (m, 2H), 3.39 (s, 3H),

2.44 (s, 3H), 2.11 (s, 3H). ^{13}C NMR (126 MHz, CD_2Cl_2) δ 144.1, 138.1, 133.2, 132.8, 132.1, 129.9, 129.5, 129.0, 128.3, 127.5, 124.1, 121.7, 119.0 (2C), 118.2, 110.3, 109.6, 108.6, 30.2, 21.3, 9.2. HRMS (ESI) calcd for [M+H] $C_{21}H_{21}N_2$, m/z: 301.1699, found: 301.1701. HPLC analysis: HPLC DAICEL CHIRALCEL OD-H, hexane/isopropanol = 99/1, 1.0 mL/min, λ = 229 nm, t_R (minor) = 15.271 min, t_R (major) = 16.767 min, er = 93:7. $[\alpha]_D^{25}$ = -7.778 (c = 0.090, CH_2Cl_2).

Chiral HPLC spectrum of (*rac*)-**8**

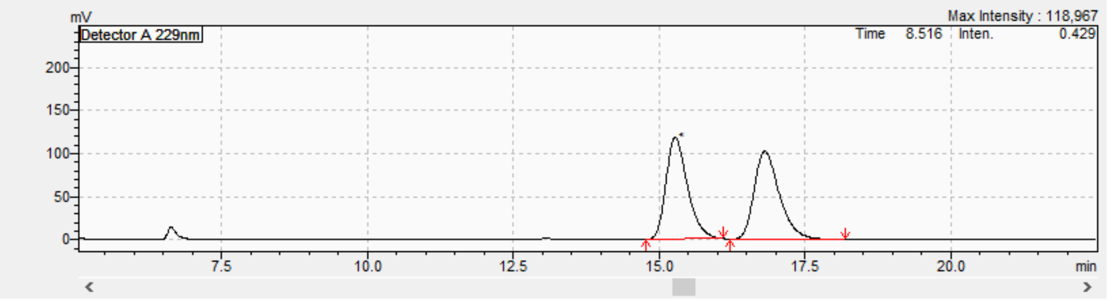

Results View - Peak Table

Peak Table | Compound | Group | Calibration Curve

Peak#	Ret. Time	Area	Height	Mark	Conc.	Area%
1	15.277	3040707	118051	M	49.894	49.894
2	16.813	3053637	101808	M	50.106	50.106
Total		6094344	219859		100.000	100.000

Chiral HPLC spectrum of (*S*)-**8**

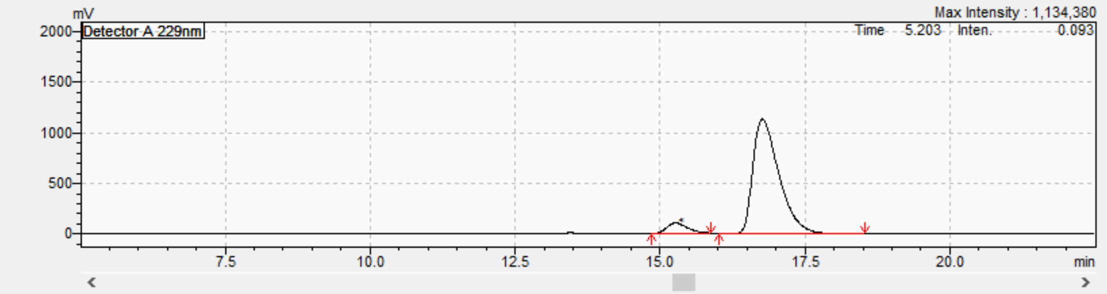

Results View - Peak Table

Peak Table | Compound | Group | Calibration Curve

Peak#	Ret. Time	Area	Height	Mark	Conc.	Area%
1	15.271	2562671	103950	M	6.803	6.803
2	16.767	35108784	1126992	M	93.197	93.197
Total		37671455	1230943		100.000	100.000

1-(1,3-dimethyl-1*H*-indol-2-yl)-6-fluoronaphthalen-2-amine (9)

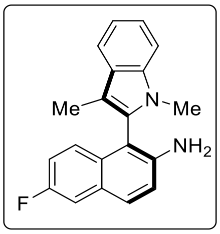

9 was obtained in 60% yield with 91% ee as a yellow-brown oil. ^{1}H NMR (400 MHz, DMSO-d_6) δ 7.76 (d, J = 8.7 Hz, 1H), 7.63 – 7.51 (m, 2H), 7.46 (dt, J = 8.2, 0.9 Hz, 1H), 7.27 – 7.05 (m, 4H), 6.98 – 6.89 (m, 1H), 5.08 (s, 2H), 3.34 (s, 3H), 2.00 (s, 3H). ^{13}C NMR (101 MHz, DMSO-d_6) δ 157.4 (d, J = 238.4 Hz), 145.5 (d, J = 2.0 Hz), 137.3, 132.3, 131.5, 129.0 (d, J = 4.7 Hz), 128.1, 126.6 (d, J = 8.7 Hz), 125.1 (d, J = 8.4 Hz), 121.0, 119.6, 118.4 (2C), 116.3 (d, J = 24.6 Hz), 111.1 (d, J = 20.1 Hz), 109.7, 108.8, 105.5, 29.8, 9.1. ^{19}F NMR (377 MHz, DMSO-d_6) δ -122.09. HRMS (ESI) calcd for [M+H] $C_{20}H_{18}FN_2$, m/z: 305.1449, found: 305.1447. HPLC analysis: HPLC DAICEL CHIRALCEL OD-H, hexane/isopropanol = 99/1, 1.0 mL/min, λ = 229 nm, t_R (minor) = 21.150 min, t_R (major) = 22.563 min, er = 95.5:4.5. $[\alpha]^{25}_{D}$ = -15.500 (c = 0.600, CH_2Cl_2).

Chiral HPLC spectrum of (*rac*)-**9**

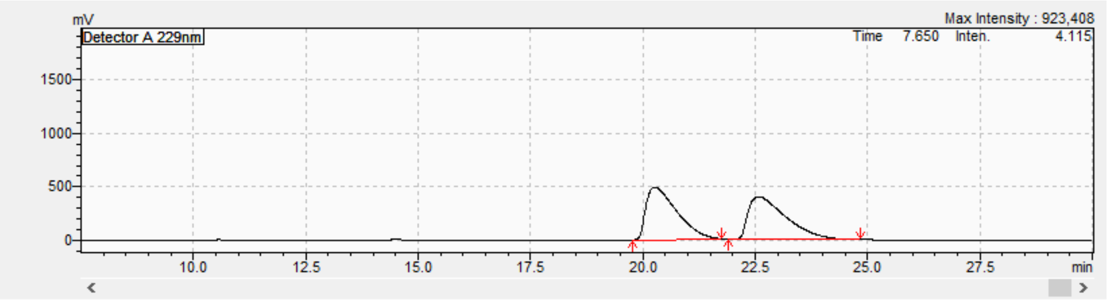

Results View - Peak Table

Peak Table | Compound | Group | Calibration Curve

Peak#	Ret. Time	Area	Height	Mark	Conc.	Area%
1	20.268	23289600	489866	M	49.850	49.850
2	22.574	23429554	400244	M	50.150	50.150
Total		46719154	890110		100.000	100.000

Chiral HPLC spectrum of (*S*)-**9**

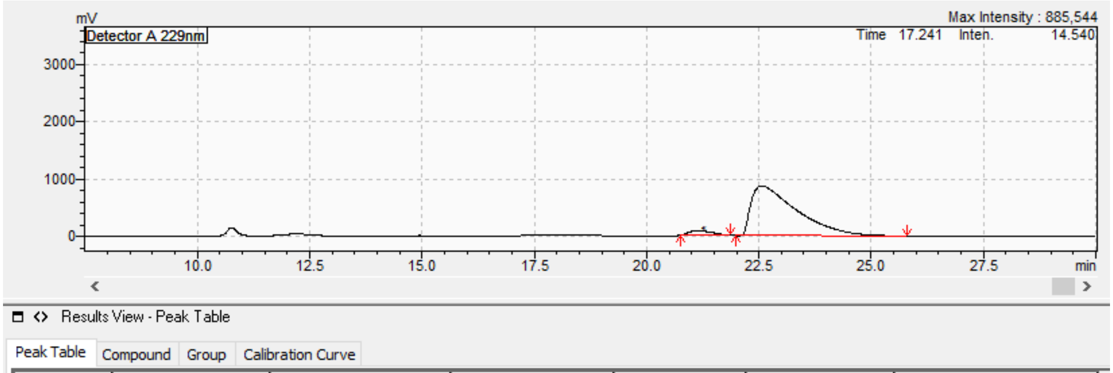

Peak#	Ret. Time	Area	Height	Mark	Conc.	Area%
1	21.150	2793007	77761	M	4.331	4.331
2	22.563	61691184	872371	M	95.669	95.669
Total		64484191	950131		100.000	100.000

6-bromo-1-(1,3-dimethyl-1*H*-indol-2-yl)naphthalen-2-amine (10)

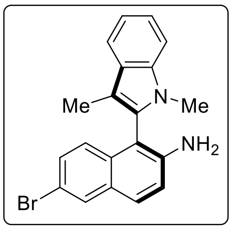

10 was obtained in 52% yield with 85% ee as a yellow-brown oil. ^{1}H NMR (500 MHz, Methylene Chloride-d_2) δ 7.92 (d, J = 2.1 Hz, 1H), 7.71 (dd, J = 8.9, 0.8 Hz, 1H), 7.65 (dt, J = 7.8, 1.0 Hz, 1H), 7.39 (dt, J = 8.1, 0.9 Hz, 1H), 7.34 (dd, J = 9.0, 2.1 Hz, 1H), 7.28 – 7.24 (m, 1H), 7.17 – 7.11 (m, 2H), 7.04 (dt, J = 8.7, 0.6 Hz, 1H), 3.39 (s, 3H), 2.10 (s, 3H). ^{13}C NMR (126 MHz, Methylene Chloride-d_2) δ 145.3, 138.1, 133.8, 131.9, 130.3 (2C), 129.7, 129.1, 128.9, 126.2, 121.9, 119.2 (2C), 119.1, 115.9, 110.7, 109.7, 108.5, 30.3, 9.1. HRMS (ESI) calcd for [M+H] $C_{20}H_{18}BrN_2$, m/z: 365.0648, found: 365.0646. HPLC analysis: HPLC DAICEL CHIRALCEL IA, hexane/isopropanol = 99/1, 1.0 mL/min, λ = 229 nm, t_R (minor) = 18.282 min, t_R (major) = 22.704 min, er = 92.5:7.5. $[\alpha]_D^{25}$ = -6.316 (c = 0.190, CH_2Cl_2).

Chiral HPLC spectrum of (*rac*)-**10**

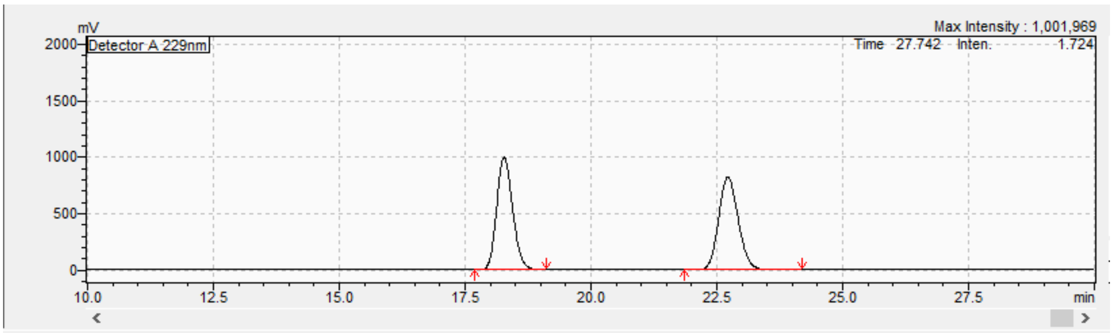

Results View - Peak Table

Peak Table | Compound | Group | Calibration Curve

Peak#	Ret. Time	Area	Height	Mark	Conc.	Area%
1	18.273	22101488	999673	M	49.870	49.870
2	22.718	22216637	821034	M	50.130	50.130
Total		44318125	1820707		100.000	100.000

Chiral HPLC spectrum of (*S*)-**10**

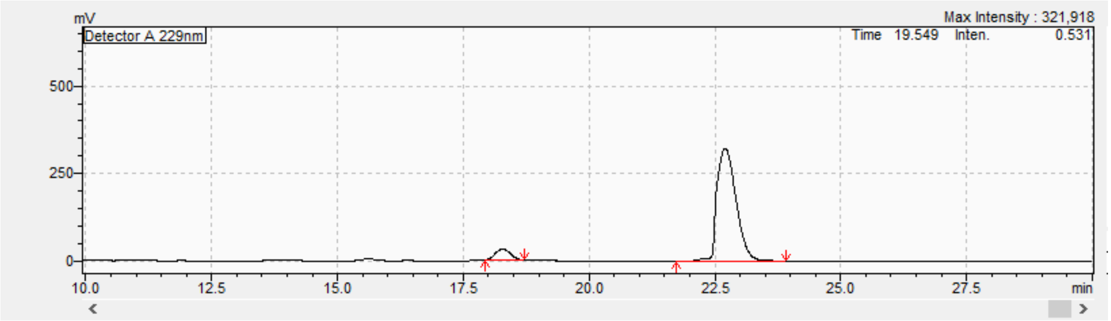

Results View - Peak Table

Peak Table | Compound | Group | Calibration Curve

Peak#	Ret. Time	Area	Height	Mark	Conc.	Area%
1	18.282	691496	33083	M	7.590	7.590
2	22.704	8419325	321769	M	92.410	92.410
Total		9110821	354852		100.000	100.000

6-cyclohexyl-1-(1,3-dimethyl-1*H*-indol-2-yl)naphthalen-2-amine (11)

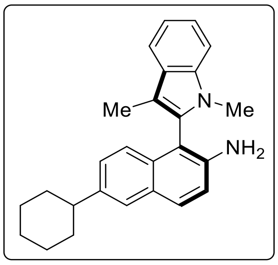

11 was obtained in 58% yield with 90% ee as a yellow-brown oil. ^{1}H NMR (400 MHz, DMSO-d_6) δ 7.70 (d, *J* = 8.8 Hz, 1H), 7.58 (dt, *J* = 7.8, 1.0 Hz, 1H), 7.53 (d, *J* = 1.8 Hz, 1H), 7.44 (dt, *J* = 8.2, 0.9 Hz, 1H), 7.20 – 7.06 (m, 4H), 6.84 (d, *J* = 8.6 Hz, 1H), 4.95 (s, 2H), 3.33

(s, 3H), 2.59 – 2.51 (m, 1H), 2.00 (s, 3H), 1.86 – 1.67 (m, 5H), 1.50 – 1.25 (m, 5H).^{13}C NMR (101 MHz, DMSO-d_6) δ 145.2, 140.3, 137.2, 133.0, 132.8, 129.5, 128.2, 126.7, 126.6, 124.7, 122.7, 120.8, 118.3 (2C), 118.1, 109.6, 108.5, 105.3, 43.4, 34.1(2C), 29.8, 26.4 (2C), 25.7, 9.1. HRMS (ESI) calcd for [M+H] $C_{26}H_{29}N_2$, m/z: 369.2325, found: 369.2324. HPLC analysis: HPLC DAICEL CHIRALCEL OD-H, hexane/isopropanol = 99/1, 1.0 mL/min, λ = 229 nm, t_R (minor) = 11.844 min, t_R (major) = 15.653 min, er = 95:5. $[\alpha]_D^{25}$ = -4.000 (*c* = 0.200, Acetone).

Chiral HPLC spectrum of (*rac*)-**11**

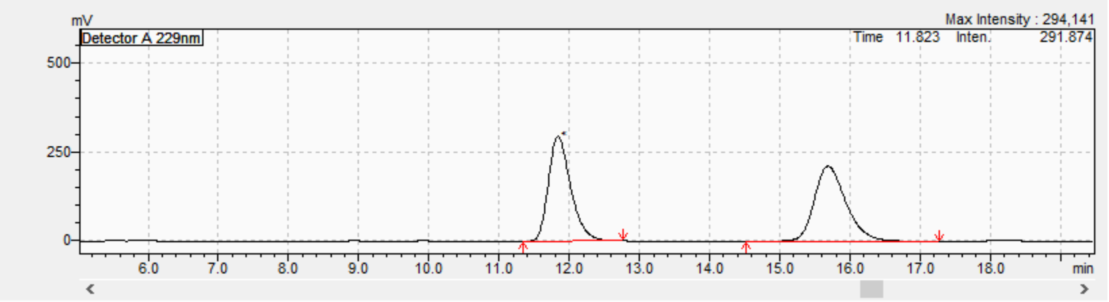

Results View - Peak Table

Peak Table | Compound | Group | Calibration Curve

Peak#	Ret. Time	Area	Height	Mark	Conc.	Area%
1	11.840	6533284	294761	M	49.586	49.586
2	15.687	6642362	211709	M	50.414	50.414
Total		13175646	506469		100.000	100.000

Chiral HPLC spectrum of (*S*)-**11**

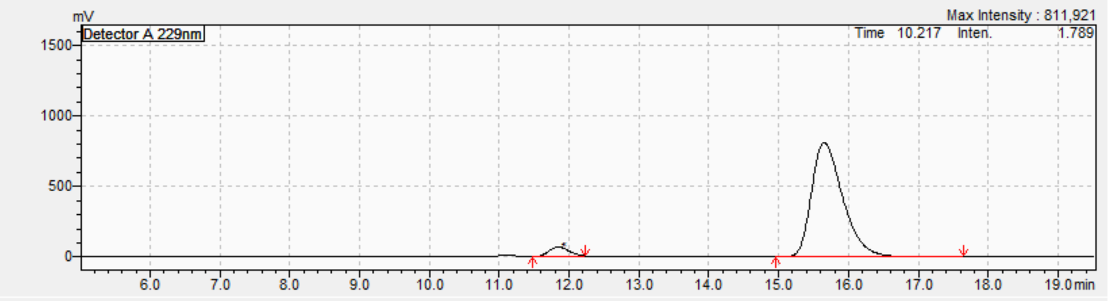

Results View - Peak Table

Peak Table | Compound | Group | Calibration Curve

Peak#	Ret. Time	Area	Height	Mark	Conc.	Area%
1	11.844	1309536	65672	M	4.942	4.942
2	15.653	25189675	809846	M	95.058	95.058
Total		26499211	875518		100.000	100.000

6-butyl-1-(1,3-dimethyl-1*H*-indol-2-yl)naphthalen-2-amine (12)

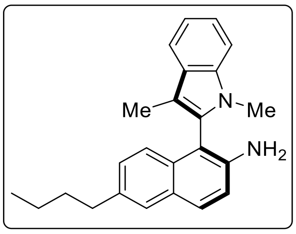

12 was obtained in 68% yield with 90% ee as a yellow-brown oil. ^{1}H NMR (400 MHz, Methylene Chloride-d_2) δ 7.73 (dd, J = 9.0, 0.8 Hz, 1H), 7.65 (dt, J = 7.8, 1.1 Hz, 1H), 7.56 (d, J = 1.8 Hz, 1H), 7.39 (dt, J = 8.2, 1.0 Hz, 1H), 7.29 – 7.23 (m, 1H), 7.19 – 7.13 (m, 2H), 7.10 – 7.05 (m, 2H), 3.40 (s, 3H), 2.74 – 2.69 (m, 2H), 2.12 (s, 3H), 1.70 – 1.63 (m, 2H), 1.45 – 1.37 (m, 2H), 0.96 (t, J = 7.3 Hz, 3H). ^{13}C NMR (101 MHz, Methylene Chloride-d_2) δ 144.1, 138.1, 137.2, 133.5, 132.8, 130.1, 129.1, 128.9, 128.3, 127.0, 124.1, 121.7, 119.1, 119.0, 118.1, 110.3, 109.6, 108.6, 35.8, 34.1, 30.3, 22.9, 14.2, 9.2. HRMS (ESI) calcd for [M+H] $C_{24}H_{27}N_2$, m/z: 343.2169, found: 343.2168. HPLC analysis: HPLC DAICEL CHIRALCEL OD-H, hexane/isopropanol = 99/1, 1.0 mL/min, λ = 229 nm, t_R (minor) = 12.469 min, t_R (major) = 15.900 min, er = 95:5. $[\alpha]_D^{25}$ = -6.667 (c = 0.420, CH_2Cl_2).

Chiral HPLC spectrum of (*rac*)-**12**

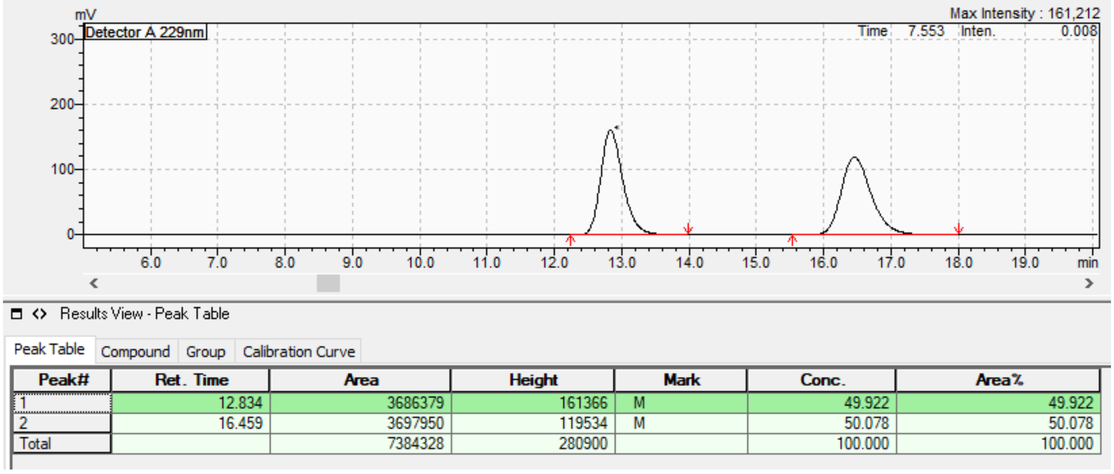

Peak#	Ret. Time	Area	Height	Mark	Conc.	Area%
1	12.834	3686379	161366	M	49.922	49.922
2	16.459	3697950	119534	M	50.078	50.078
Total		7384328	280900		100.000	100.000

Chiral HPLC spectrum of (*S*)-**12**

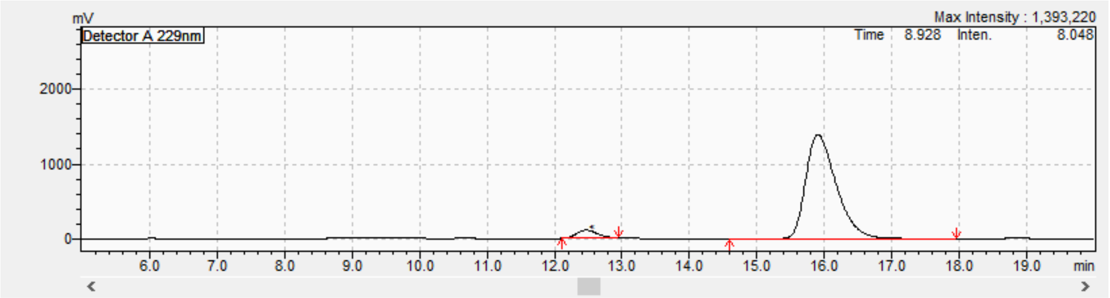

Results View - Peak Table

Peak Table | Compound | Group | Calibration Curve

Peak#	Ret. Time	Area	Height	Mark	Conc.	Area%
1	12.469	2352917	108436	M	5.118	5.118
2	15.900	43620116	1392822	M	94.882	94.882
Total		45973034	1501258		100.000	100.000

1-(1,3-dimethyl-1*H*-indol-2-yl)-8-methylnaphthalen-2-amine (13)

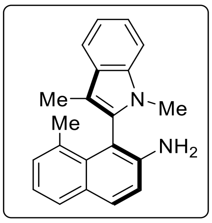

13 was obtained in 30% yield with 10% ee as an off-white solid. ^{1}H NMR (400 MHz, Methylene Chloride-d_2) δ 7.78 (d, *J* = 8.8 Hz, 1H), 7.66 – 7.58 (m, 2H), 7.40 – 7.36 (m, 1H), 7.26 – 7.22 (m, 1H), 7.17 – 7.10 (m, 3H), 7.07 (d, *J* = 8.7 Hz, 1H), 3.83 (s, 2H), 3.38 (s, 3H), 2.12 (s, 3H), 1.79 (s, 3H). ^{13}C NMR (101 MHz, Methylene Chloride-d_2) δ 145.9, 137.3, 135.4, 134.3, 133.3, 132.0, 130.8, 129.3, 128.9, 127.9, 122.4, 121.6, 119.2, 119.1, 117.4, 110.1, 109.7, 107.6, 30.1, 21.3, 9.2. HRMS (ESI) calcd for $[M+H]^+$ $C_{21}H_{21}N_2$, m/z: 301.1699, found: 301.1697. HPLC analysis: HPLC DAICEL CHIRALCEL OD-H, hexane/isopropanol = 99/1, 1.0 mL/min, λ = 229 nm, t_R (major) = 13.176 min, t_R (minor) = 20.745 min, er = 55:45. $[\alpha]_D^{25}$ = -2.500 (*c* = 0.200, CH_2Cl_2).

Chiral HPLC spectrum of (*rac*)-**13**

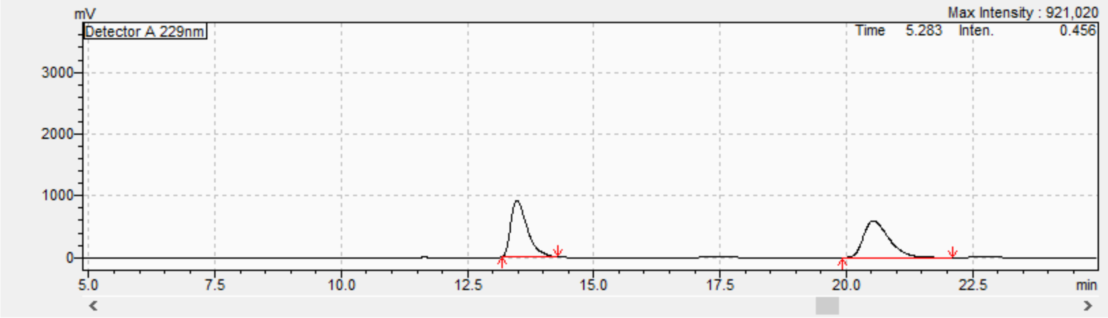

Results View - Peak Table

Peak Table | Compound | Group | Calibration Curve

Peak#	Ret. Time	Area	Height	Mark	Conc.	Area%
1	13.479	20734464	912066	M	49.939	49.939
2	20.539	20784953	596275	M	50.061	50.061
Total		41519416	1508341		100.000	100.000

Chiral HPLC spectrum of (*S*)-**13**

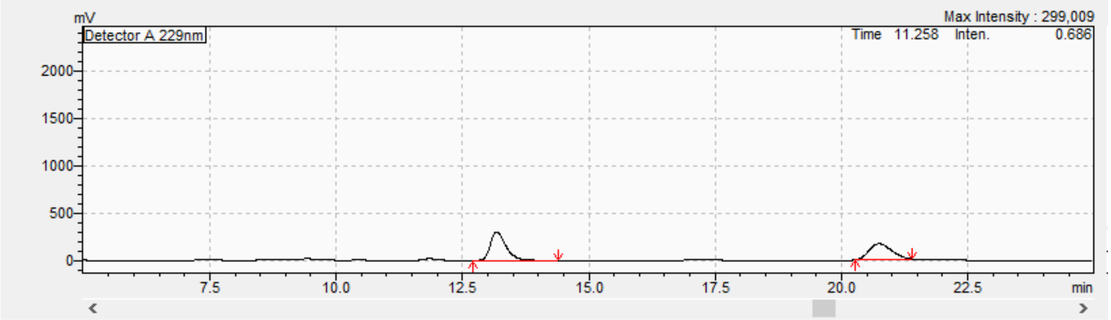

Results View - Peak Table

Peak Table | Compound | Group | Calibration Curve

Peak#	Ret. Time	Area	Height	Mark	Conc.	Area%
1	13.176	6383880	298399	M	55.096	55.096
2	20.745	5202946	167766	M	44.904	44.904
Total		11586826	466165		100.000	100.000

1-(1,3,4-trimethyl-1*H*-indol-2-yl)naphthalen-2-amine (15)

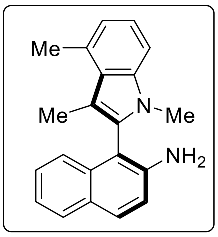

15 was obtained in 60% yield with 89% ee as a yellow-brown oil. ^{1}H NMR (400 MHz, Methylene Chloride-*d*$_2$) δ 7.83 – 7.74 (m, 2H), 7.32 – 7.20 (m, 3H), 7.18 – 7.08 (m, 3H), 6.91 – 6.84 (m, 1H), 3.93 (s, 2H), 3.36 (s, 3H), 2.80 (s, 3H), 2.29 (s, 3H). ^{13}C NMR (101 MHz, Methylene Chloride-*d*$_2$) δ 145.0, 138.5, 135.3, 132.4, 131.6, 130.5, 128.4, 128.1, 127.5, 127.3,

124.2, 122.6, 121.7, 120.7, 118.1, 111.1, 108.6, 107.7, 30.3, 20.4, 12.2. HRMS (ESI) calcd for [M+H] $C_{21}H_{21}N_2$, m/z: 301.1699, found: 301.1700. HPLC analysis: HPLC DAICEL CHIRALCEL IA, hexane/isopropanol = 99/1, 1.0 mL/min, λ = 229 nm, t_R (minor) = 13.882 min, t_R (major) = 17.748 min, er = 94.5:5.5. $[\alpha]_D^{25}$ = -5.000 (*c* = 0.240, Acetone).

Chiral HPLC spectrum of (*rac*)-**15**

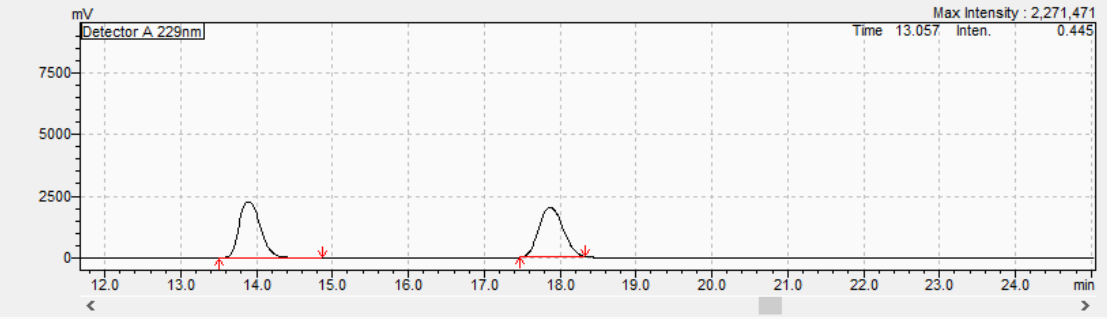

Results View - Peak Table

Peak Table | Compound | Group | Calibration Curve

Peak#	Ret. Time	Area	Height	Mark	Conc.	Area%
1	13.892	44032013	2269450	M	49.687	49.687
2	17.870	44585910	2008534	M	50.313	50.313
Total		88617923	4277984		100.000	100.000

Chiral HPLC spectrum of (*S*)-**15**

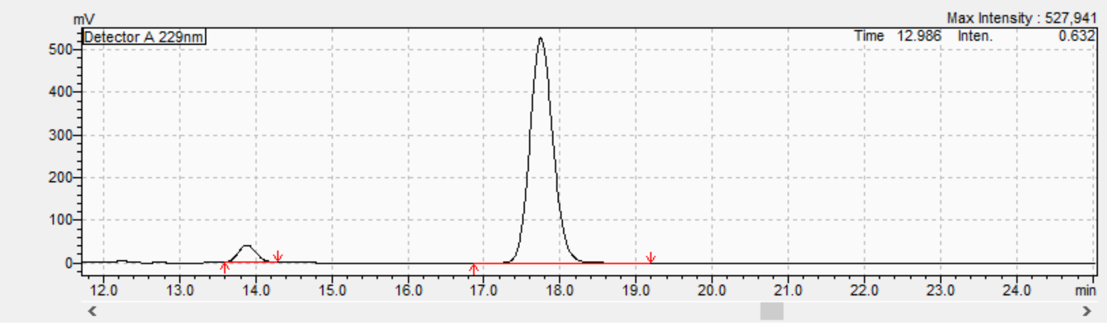

Results View - Peak Table

Peak Table | Compound | Group | Calibration Curve

Peak#	Ret. Time	Area	Height	Mark	Conc.	Area%
1	13.882	661785	40563	M	5.612	5.612
2	17.748	11130531	527672	M	94.388	94.388
Total		11792315	568235		100.000	100.000

1-(4-bromo-1,3-dimethyl-1*H*-indol-2-yl)naphthalen-2-amine (16)

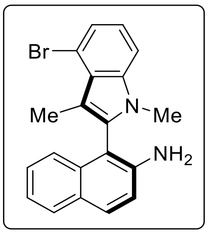

16 was obtained in 58% yield with 87% ee as a yellow-brown oil. ^{1}H NMR (400 MHz, Methylene Chloride-d_2) δ 7.85 – 7.72 (m, 2H), 7.37 – 7.19 (m, 4H), 7.14 – 7.03 (m, 3H), 4.00 (s, 2H), 3.37 (s, 3H), 2.32 (s, 3H). ^{13}C NMR (101 MHz, Methylene Chloride-d_2) δ 144.8, 139.4, 135.0, 134.5, 130.9, 128.5, 128.1, 127.5, 126.9, 124.0, 123.5, 122.7, 122.4, 118.1, 114.9, 111.3, 109.2, 107.8, 30.5, 11.6. HRMS (ESI) calcd for [M+H] $C_{20}H_{18}BrN_2$, m/z: 365.0648, found: 365.0648. HPLC analysis: HPLC DAICEL CHIRALCEL OD-H, hexane/isopropanol = 99/1, 1.0 mL/min, λ = 229 nm, t_R (minor) = 20.020 min, t_R (major) = 24.527 min, er = 93.5:6.5. $[\alpha]_D^{25}$ = -7.000 (c = 0.100, CH_2Cl_2).

Chiral HPLC spectrum of (*rac*)-**16**

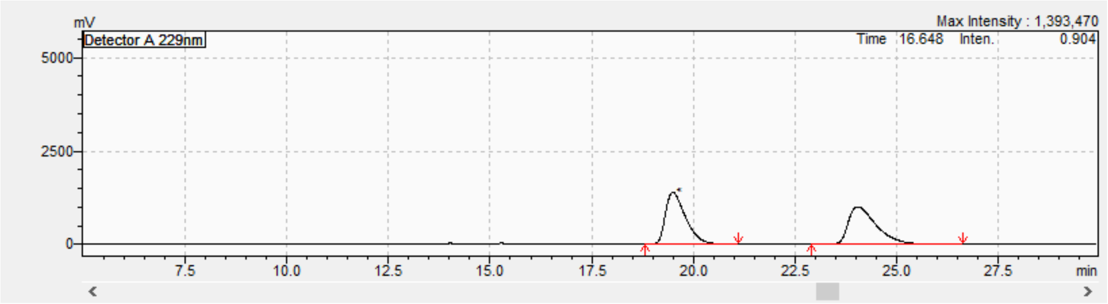

Results View - Peak Table

Peak Table | Compound | Group | Calibration Curve

Peak#	Ret. Time	Area	Height	Mark	Conc.	Area%
1	19.498	47652736	1392092	M	49.688	49.688
2	24.037	48250360	1005131	M	50.312	50.312
Total		95903096	2397223		100.000	100.000

Chiral HPLC spectrum of (*S*)-**16**

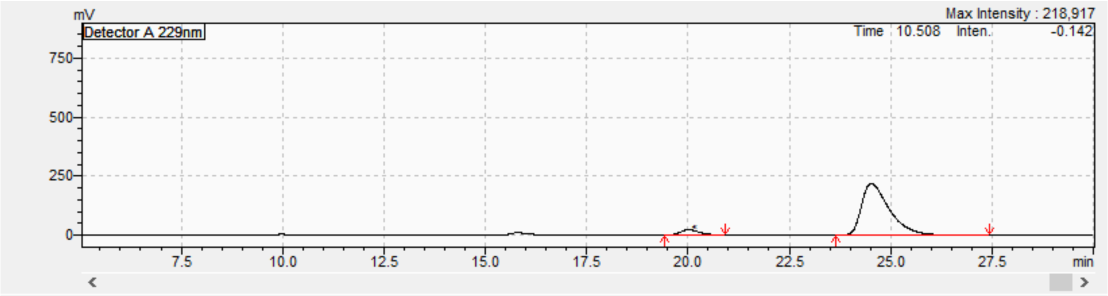

Results View - Peak Table

Peak Table | Compound | Group | Calibration Curve

Peak#	Ret. Time	Area	Height	Mark	Conc.	Area%
1	20.020	752460	22923	M	6.710	6.710
2	24.527	10461610	218726	M	93.290	93.290
Total		11214069	241649		100.000	100.000

1-(5-methoxy-1,3-dimethyl-1*H*-indol-2-yl)naphthalen-2-amine (17)

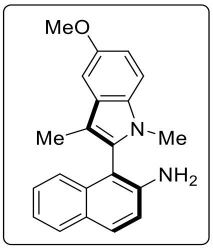

17 was obtained in 45% yield with 84% ee as a yellow-brown oil. ^{1}H NMR (400 MHz, Methylene Chloride-d_2) δ 7.82 – 7.75 (m, 2H), 7.31 – 7.22 (m, 3H), 7.17 – 7.08 (m, 3H), 6.92 (dd, *J* = 8.8, 2.5 Hz, 1H), 3.91 (s, 3H), 3.37 (s, 3H), 2.10 (s, 3H). ^{13}C NMR (101 MHz, Methylene Chloride-d_2) δ 154.3, 144.8, 135.1, 133.5, 133.3, 130.6, 129.2, 128.4, 128.1, 127.3, 124.2, 122.6, 118.1, 111.7, 110.4, 109.9, 108.7, 101.1, 56.3, 30.4, 9.3. HRMS (ESI) calcd for [M+H] $C_{21}H_{21}N_2O$, m/z: 317.1648, found: 317.1650. HPLC analysis: HPLC DAICEL CHIRALCEL OD-H, hexane/isopropanol = 99/1, 1.0 mL/min, λ = 229 nm, t_R (minor) = 26.585 min, t_R (major) = 29.410 min, er = 92:8. $[\alpha]_D^{25}$ = -6.154 (*c* = 0.130, CH_2Cl_2).

Chiral HPLC spectrum of (*rac*)-**17**

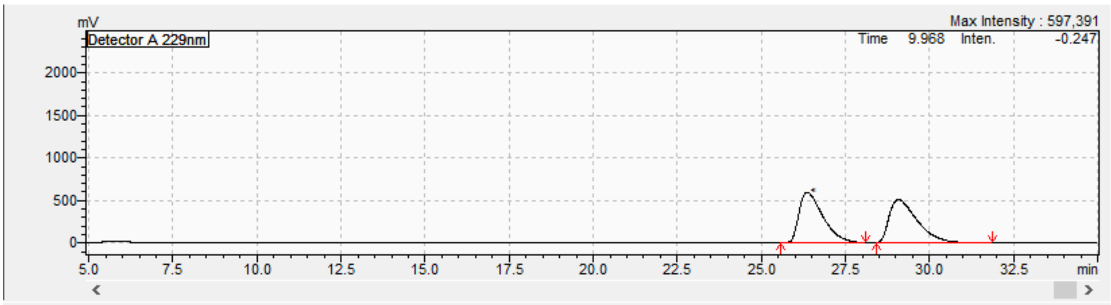

Results View - Peak Table

Peak Table | Compound | Group | Calibration Curve

Peak#	Ret. Time	Area	Height	Mark	Conc.	Area%
1	26.363	29543774	596560	M	49.995	49.995
2	29.075	29550182	509725	M	50.005	50.005
Total		59093956	1106285		100.000	100.000

Chiral HPLC spectrum of (*S*)-**17**

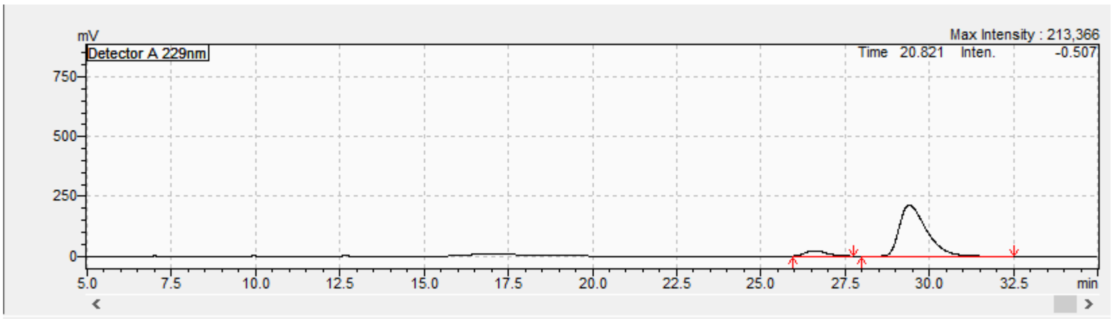

Results View - Peak Table

Peak Table | Compound | Group | Calibration Curve

Peak#	Ret. Time	Area	Height	Mark	Conc.	Area%
1	26.585	1012352	22858	M	7.866	7.866
2	29.410	11858294	213886	M	92.134	92.134
Total		12870646	236744		100.000	100.000

1-(6-fluoro-1,3-dimethyl-1*H*-indol-2-yl)naphthalen-2-amine (18)

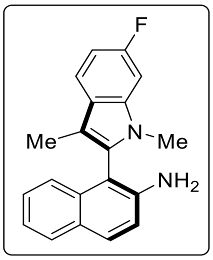

18 was obtained in 83% yield with 87% ee as a yellow-brown oil. ^{1}H NMR (400 MHz, Methylene Chloride-d_2) δ 7.83 – 7.76 (m, 2H), 7.61 – 7.54 (m, 1H), 7.34 – 7.21 (m, 2H), 7.17 – 7.06 (m, 3H), 6.98 – 6.90 (m, 1H), 3.93 (s, 2H), 3.35 (s, 3H), 2.11 (s, 3H). ^{13}C NMR (101

MHz, Methylene Chloride-d_2) δ 160.3 (d, J = 236.2 Hz), 144.9, 138.1 (d, J = 12.1 Hz), 135.1, 133.0 (d, J = 3.9 Hz), 130.7, 128.5, 128.1, 127.4, 125.7, 124.1, 122.6, 119.8 (d, J = 10.1 Hz), 118.1, 110.7, 108.2, 107.4 (d, J = 24.5 Hz), 96.0 (d, J = 26.1 Hz), 30.4, 9.2. ^{19}F NMR (377 MHz, Methylene Chloride-d_2) δ -122.20. HRMS (ESI) calcd for [M+H] $C_{20}H_{18}FN_2$, m/z: 305.1449, found: 305.1452. HPLC analysis: HPLC DAICEL CHIRALCEL OD-H, hexane/isopropanol = 99/1, 1.0 mL/min, λ = 229 nm, t_R (minor) = 14.469 min, t_R (major) = 18.553 min, er = 93.5:6.5. $[\alpha]_D^{25}$ = -12.121 (c = 0.660, CH_2Cl_2).

Chiral HPLC spectrum of (*rac*)-**18**

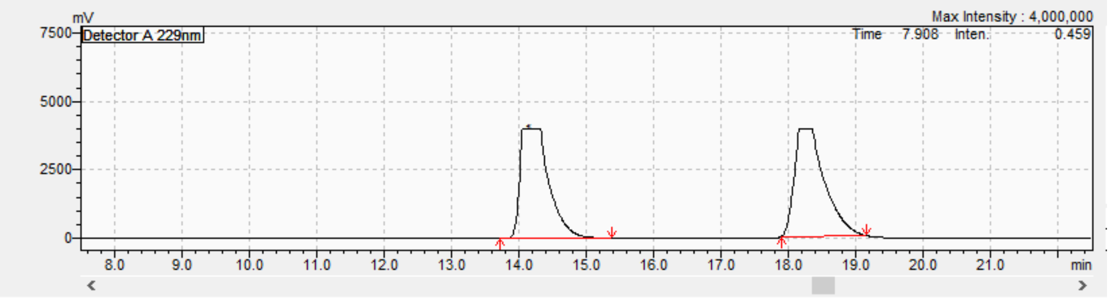

Results View - Peak Table

Peak Table | Compound | Group | Calibration Curve

Peak#	Ret. Time	Area	Height	Mark	Conc.	Area%
1	14.063	118774669	3996523	M	49.131	49.131
2	18.171	122974575	3943551	M	50.869	50.869
Total		241749244	7940074		100.000	100.000

Chiral HPLC spectrum of (*S*)-**18**

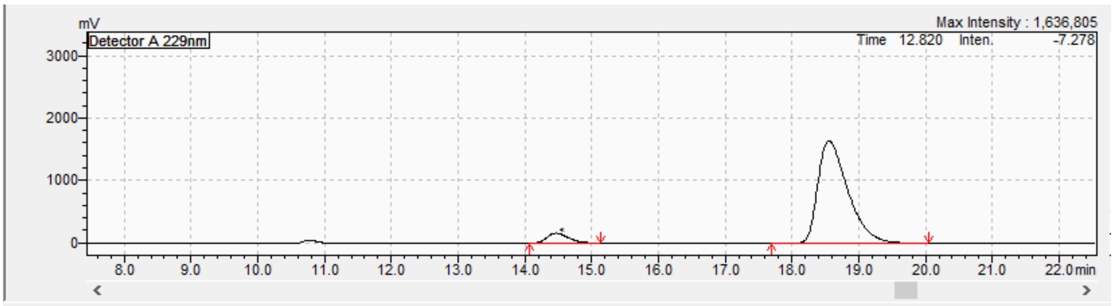

Results View - Peak Table

Peak Table | Compound | Group | Calibration Curve

Peak#	Ret. Time	Area	Height	Mark	Conc.	Area%
1	14.469	3613396	160044	M	6.606	6.606
2	18.553	51086608	1642873	M	93.394	93.394
Total		54700004	1802917		100.000	100.000

1-(6-bromo-1,3-dimethyl-1*H*-indol-2-yl)naphthalen-2-amine (19)

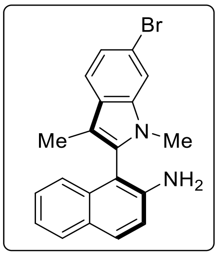

19 was obtained in 62% yield with 86% ee as a yellow-brown oil. ^{1}H NMR (400 MHz, Methylene Chloride-d_2) δ 7.84 – 7.74 (m, 2H), 7.63 – 7.47 (m, 2H), 7.32 – 7.22 (m, 3H), 7.10 (dd, J = 8.5, 1.5 Hz, 2H), 4.02 (s, 2H), 3.36 (s, 3H), 2.09 (s, 3H). ^{13}C NMR (101 MHz, Methylene Chloride-d_2) δ 144.7, 138.9, 135.0, 133.5, 130.9, 128.5, 128.2, 127.9, 127.5, 124.0, 122.7, 122.2, 120.4, 118.1, 115.2, 112.7, 110.9, 108.0, 30.4, 9.1. HRMS (ESI) calcd for [M+H] $C_{20}H_{18}BrN_2$, m/z: 365.0648, found: 365.0648. HPLC analysis: HPLC DAICEL CHIRALCEL OD-H, hexane/isopropanol = 99/1, 1.0 mL/min, λ = 229 nm, t_R (minor) = 16.386 min, t_R (major) = 25.168 min, er = 93:7. $[\alpha]_D^{25}$ = -16.842 (c = 0.190, CH_2Cl_2).

Chiral HPLC spectrum of (*rac*)-**19**

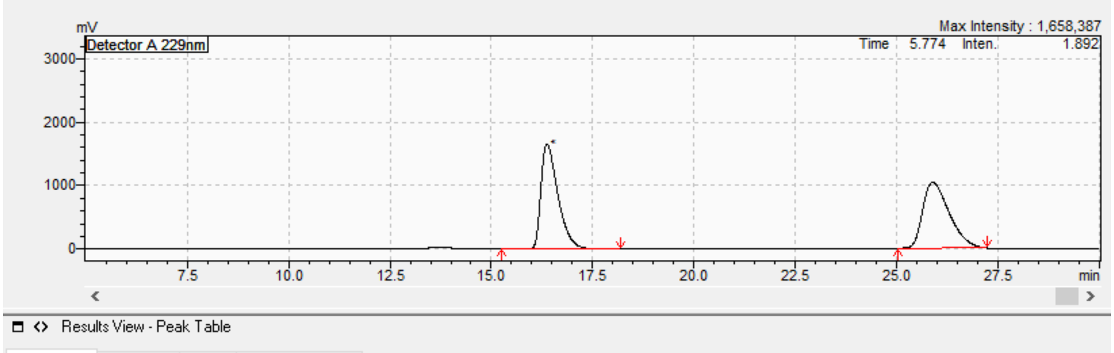

Peak#	Ret. Time	Area	Height	Mark	Conc.	Area%
1	16.375	47386765	1650681	M	50.078	50.078
2	25.886	47239694	1038237	M	49.922	49.922
Total		94626459	2688918		100.000	100.000

Chiral HPLC spectrum of (*S*)-**19**

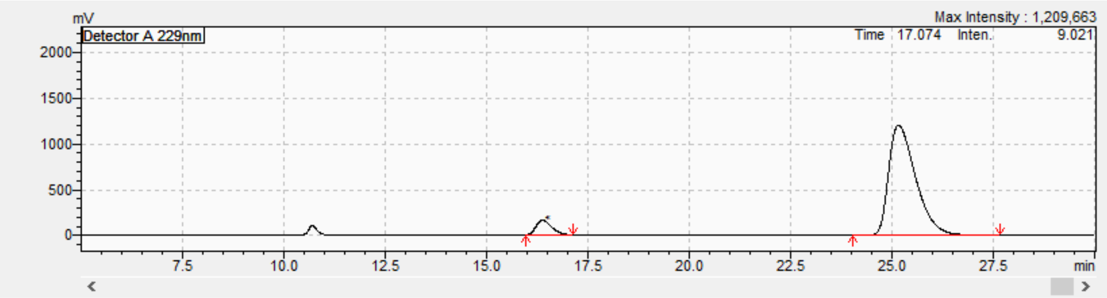

Results View - Peak Table

Peak Table | Compound | Group | Calibration Curve

Peak#	Ret. Time	Area	Height	Mark	Conc.	Area%
1	16.386	4350563	162541	M	7.277	7.277
2	25.168	55433379	1205258	M	92.723	92.723
Total		59783943	1367799		100.000	100.000

1-(5-methoxy-1,3-dimethyl-1*H*-indol-2-yl)-7-methylnaphthalen-2-amine (20)

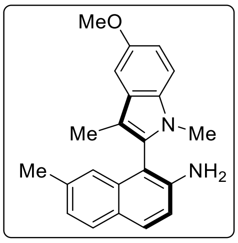

20 was obtained in 65% yield with 88% ee as a yellow-brown oil. ^{1}H NMR (400 MHz, Methylene Chloride-d_2) δ 7.73 (dd, *J* = 8.8, 0.8 Hz, 1H), 7.66 (d, *J* = 8.2 Hz, 1H), 7.29 (dd, *J* = 8.7, 0.6 Hz, 1H), 7.14 – 7.05 (m, 2H), 7.02 (d, *J* = 8.8 Hz, 1H), 6.96 – 6.85 (m, 2H), 3.90 (s, 3H), 3.89 (s, 2H), 3.37 (s, 3H), 2.32 (s, 3H), 2.08 (s, 3H). ^{13}C NMR (101 MHz, Methylene Chloride-d_2) δ 154.3, 144.9, 137.3, 135.3, 133.6, 133.5, 130.3, 129.3, 128.3, 126.3, 124.8, 123.2, 117.1, 111.6, 110.4, 109.8, 108.2, 101.1, 56.3, 30.4, 22.0, 9.3. HRMS (ESI) calcd for [M+H] $C_{22}H_{23}N_2O$, m/z: 331.1805, found: 331.1806. HPLC analysis: HPLC DAICEL CHIRALCEL OD-H, hexane/isopropanol = 99/1, 1.0 mL/min, λ = 229 nm, t_R (minor) = 24.833 min, t_R (major) = 28.717 min, er = 94:6. $[\alpha]_D^{25}$ = -6.316 (*c* = 0.190, CH_2Cl_2).

Chiral HPLC spectrum of (*rac*)-**20**

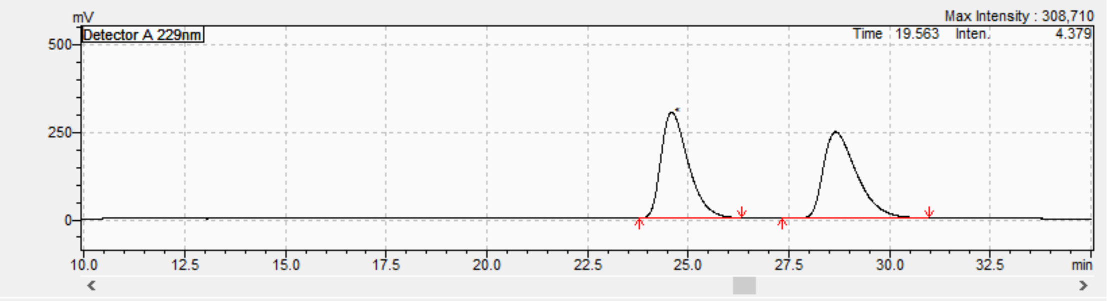

Results View - Peak Table

Peak Table | Compound | Group | Calibration Curve

Peak#	Ret. Time	Area	Height	Mark	Conc.	Area%
1	24.589	14210327	303891	M	49.976	49.976
2	28.659	14223727	247135	M	50.024	50.024
Total		28434054	551026		100.000	100.000

Chiral HPLC spectrum of (*S*)-**20**

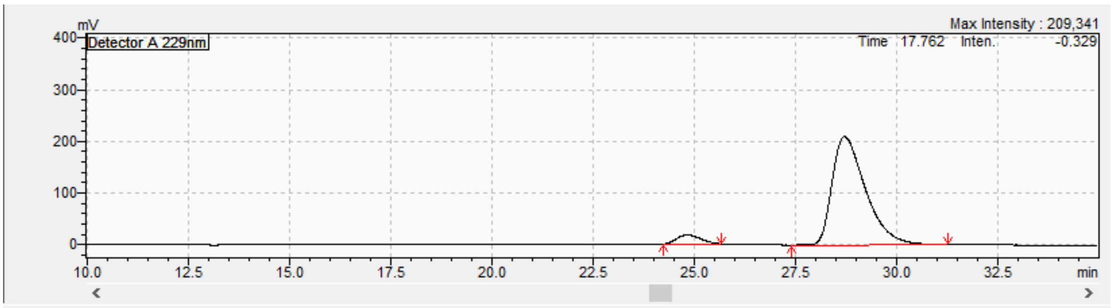

Results View - Peak Table

Peak Table | Compound | Group | Calibration Curve

Peak#	Ret. Time	Area	Height	Mark	Conc.	Area%
1	24.833	744339	17931	M	5.831	5.831
2	28.717	12021349	209608	M	94.169	94.169
Total		12765688	227539		100.000	100.000

6-fluoro-1-(6-fluoro-1,3-dimethyl-1*H*-indol-2-yl)naphthalen-2-amine (21)

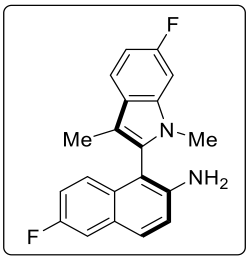

21 was obtained in 82% yield with 85% ee as a yellow-brown oil. ^{1}H NMR (400 MHz, Methylene Chloride-d_2) δ 7.74 (d, *J* = 8.8 Hz, 1H), 7.57 (dd, *J* = 8.7, 5.4 Hz, 1H), 7.42 (dd, *J* = 9.7, 2.6 Hz, 1H), 7.16 – 7.06 (m, 4H), 6.97 – 6.90 (m, 1H), 3.94 (s, 2H), 3.34 (s, 3H), 2.09 (s, 3H). ^{13}C NMR (101 MHz, Methylene Chloride-d_2) δ 160.3 (d, *J* = 236.5 Hz), 159.1 (d, *J* = 241.2 Hz), 144.3 (d, *J* = 2.3 Hz), 138.2 (d, *J* = 12.1 Hz), 132.7 (d, *J* = 3.8 Hz), 132.1, 129.9 (d, *J* = 4.9 Hz), 128.4 (d, *J* = 8.8 Hz), 126.4 (d, *J* = 8.3 Hz), 125.6, 119.9 (d, *J* = 10.2 Hz), 119.4, 117.1 (d, *J* = 24.8 Hz), 111.5 (d, *J* = 20.5 Hz), 110.9, 108.6, 107.5 (d, *J* = 24.4 Hz), 96.1 (d, *J* = 26.1 Hz), 30.4, 9.2. ^{19}F NMR (377 MHz, Methylene Chloride-d_2) δ -121.88. HRMS (ESI) calcd for [M+H] $C_{20}H_{17}F_2N_2$, m/z: 323.1354, found: 323.1357. HPLC analysis: HPLC DAICEL CHIRALCEL IA, hexane/isopropanol = 99/1, 1.0 mL/min, λ = 229 nm, t_R (minor) = 15.872 min, t_R (major) = 28.311 min, er = 92.5:7.5. $[\alpha]_D^{25}$ = -7.288 (*c* = 0.590, CH_2Cl_2).

Chiral HPLC spectrum of (*rac*)-**21**

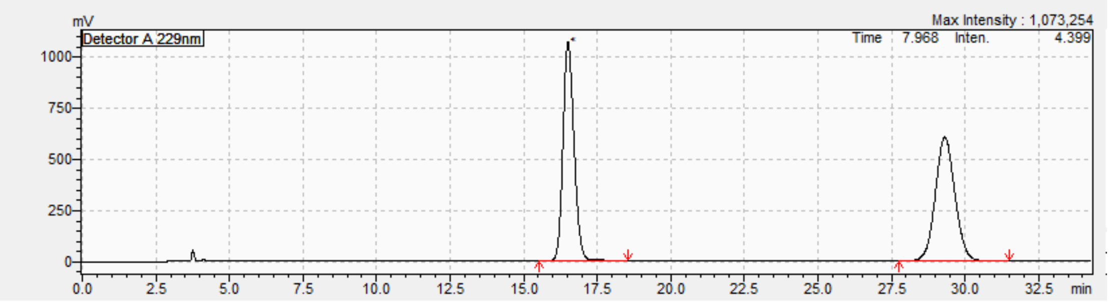

Results View - Peak Table

Peak Table | Compound | Group | Calibration Curve

Peak#	Ret. Time	Area	Height	Mark	Conc.	Area%
1	16.508	28061706	1068406	M	50.043	50.043
2	29.313	28013008	605653	M	49.957	49.957
Total		56074713	1674059		100.000	100.000

Chiral HPLC spectrum of (*S*)-**21**

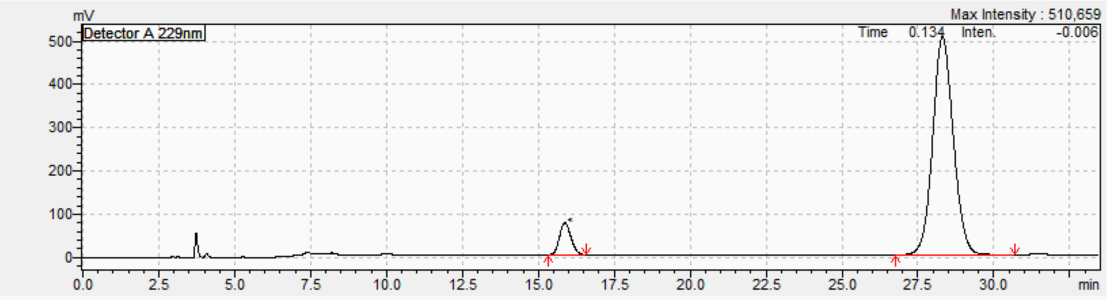

Results View - Peak Table

Peak Table | Compound | Group | Calibration Curve

Peak#	Ret. Time	Area	Height	Mark	Conc.	Area%
1	15.872	1970959	74511	M	7.647	7.647
2	28.311	23803874	504226	M	92.353	92.353
Total		25774832	578737		100.000	100.000

7-phenyl-1-(1,3,4-trimethyl-1*H*-indol-2-yl)naphthalen-2-amine (22)

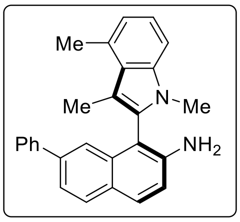

22 was obtained in 56% yield with 91% ee as a yellow-brown oil. ^{1}H NMR (400 MHz, Methylene Chloride-d_2) δ 7.89 – 7.79 (m, 2H), 7.58 – 7.49 (m, 3H), 7.44 – 7.34 (m, 3H), 7.33 – 7.25 (m, 1H), 7.22 (d, *J* = 8.2 Hz, 1H), 7.16 – 7.07 (m, 2H), 6.91 – 6.83 (m, 1H), 3.92 (s, 2H), 3.40 (s, 3H), 2.80 (s, 3H), 2.32 (s, 3H). ^{13}C NMR (101 MHz, Methylene Chloride-d_2) δ 145.4, 141.8, 140.1, 138.5, 135.6, 132.3, 131.7, 130.3, 129.1 (3C), 127.7 (2C), 127.6 (2C), 127.4, 122.3, 122.1, 121.7, 120.7, 118.2, 111.2, 108.9, 107.8, 30.4, 20.5, 12.3. HRMS (ESI) calcd for [M+H] $C_{27}H_{25}N_2$, m/z: 377.2012, found: 377.2011. HPLC analysis: HPLC DAICEL CHIRALCEL IA, hexane/isopropanol = 99/1, 0.5 mL/min, λ = 229 nm, t_R (minor) = 39.823 min, t_R (major) = 41.549 min, er = 95.5:4.5. $[\alpha]_D^{25}$ = 9.231 (*c* = 0.070, CH_2Cl_2).

Chiral HPLC spectrum of (*rac*)-**22**

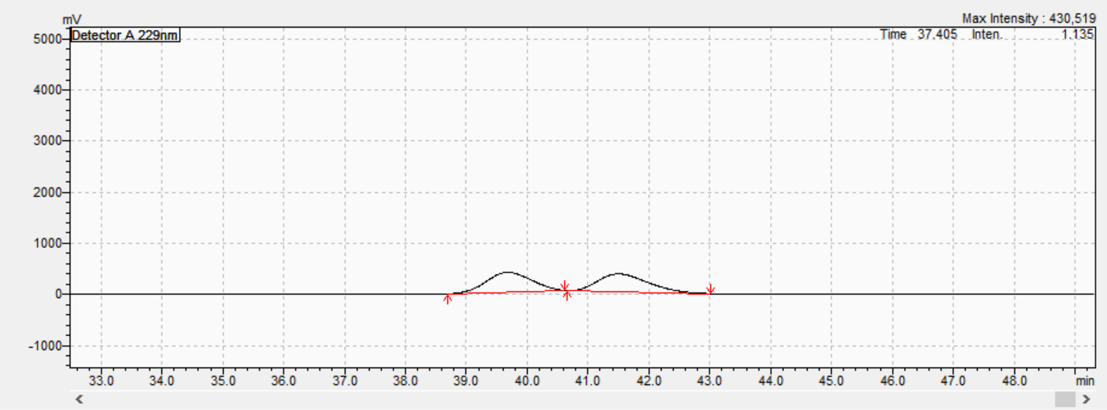

Results View - Peak Table

Peak Table | Compound | Group | Calibration Curve

Peak#	Ret. Time	Area	Height	Mark	Conc.	Area%
1	39.682	19835834	384444	M	50.084	50.084
2	41.498	19768954	346174	M	49.916	49.916
Total		39604788	730618		100.000	100.000

Chiral HPLC spectrum of (*S*)-**22**

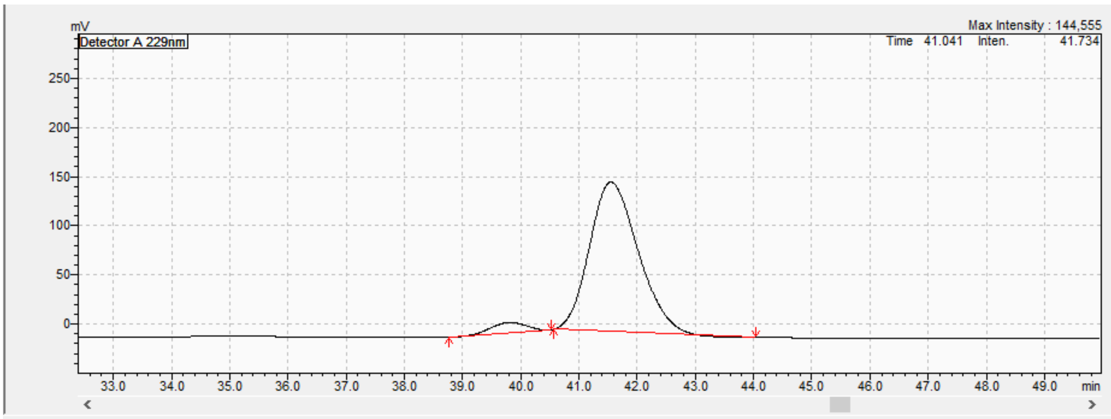

Results View - Peak Table

Peak Table | Compound | Group | Calibration Curve

Peak#	Ret. Time	Area	Height	Mark	Conc.	Area%
1	39.823	440387	10431	M	4.754	4.754
2	41.549	8822766	152180	M	95.246	95.246
Total		9263153	162611		100.000	100.000

6-cyclohexyl-1-(5-methoxy-1,3-dimethyl-1*H*-indol-2-yl)naphthalen-2-amine (23)

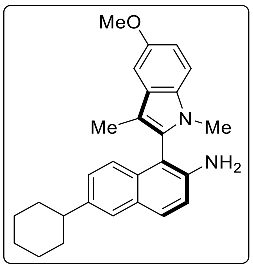

23 was obtained in 76% yield with 85% ee as a yellow-brown oil. ^{1}H NMR (400 MHz, Methylene Chloride-d_2) δ 7.73 (dd, J = 8.9, 0.8 Hz, 1H), 7.56 (d, J = 1.9 Hz, 1H), 7.27 (dd, J = 8.8, 0.6 Hz, 1H), 7.19 (dd, J = 8.7, 1.9 Hz, 1H), 7.14 – 7.01 (m, 3H), 6.90 (dd, J = 8.8, 2.5 Hz, 1H), 3.90 (s, 3H), 3.86 (s, 2H), 3.36 (s, 3H), 2.67 – 2.53 (m, 1H), 2.08 (s, 3H), 1.95 – 1.75 (m, 5H), 1.53 – 1.29 (m, 5H). ^{13}C NMR (101 MHz, Methylene Chloride-d_2) δ 154.3, 144.2, 142.4, 133.6 (2C), 133.4, 130.3, 129.2, 128.3, 127.6, 125.2, 124.1, 118.0, 111.6, 110.3, 109.8, 108.7, 101.0, 56.3, 44.6, 34.9 (2C), 30.4, 27.4 (2C), 26.7, 9.3. HRMS (ESI) calcd for [M+H] $C_{27}H_{31}N_2O$, m/z: 399.2431, found: 399.2432. HPLC analysis: HPLC DAICEL CHIRALCEL OD-H, hexane/isopropanol = 99/1, 1.0 mL/min, λ = 229 nm, t_R (minor) = 21.033 min, t_R (major) = 25.422 min, er = 92.5:7.5. $[\alpha]_D^{25}$ = -6.875 (c = 0.160, CH_2Cl_2).

Chiral HPLC spectrum of (*rac*)-**23**

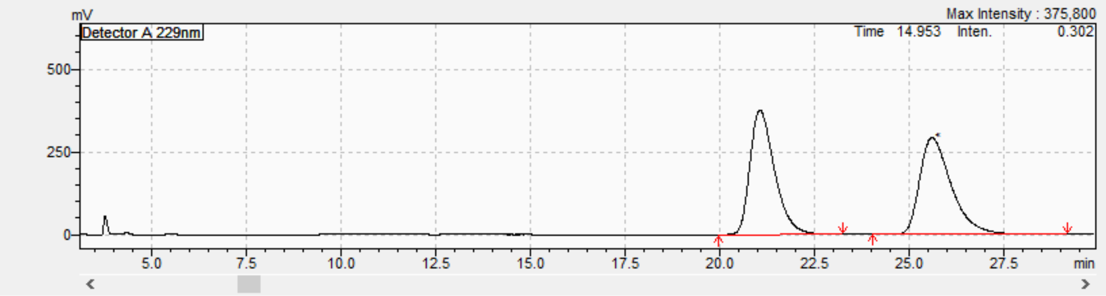

Results View - Peak Table

Peak Table | Compound | Group | Calibration Curve

Peak#	Ret. Time	Area	Height	Mark	Conc.	Area%
1	21.051	16919896	375324	M	49.799	49.799
2	25.596	17056433	291532	M	50.201	50.201
Total		33976330	666856		100.000	100.000

Chiral HPLC spectrum of (*S*)-**23**

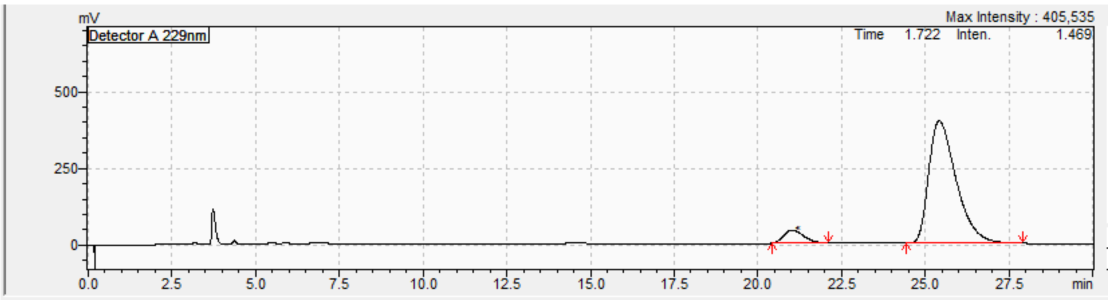

Results View - Peak Table

Peak Table | Compound | Group | Calibration Curve

Peak#	Ret. Time	Area	Height	Mark	Conc.	Area%
1	21.033	1799998	42861	M	7.312	7.312
2	25.422	22815682	399251	M	92.688	92.688
Total		24615680	442112		100.000	100.000

6-butyl-1-(1,3,4-trimethyl-1*H*-indol-2-yl)naphthalen-2-amine (24)

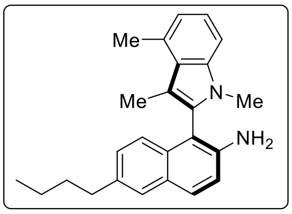

24 was obtained in 54% yield with 89% ee as a yellow-brown oil. ^{1}H NMR (400 MHz, Methylene Chloride-d_2) δ 7.73 (dd, *J* = 8.9, 0.8 Hz, 1H), 7.56 (d, *J* = 1.7 Hz, 1H), 7.23 – 7.15 (m, 2H), 7.13 – 7.05 (m, 3H), 6.89 – 6.83 (m, 1H), 3.87 (s, 2H), 3.35 (s, 3H), 2.79 (s, 3H), 2.74 – 2.69 (m, 2H), 2.28 (s, 3H), 1.71 – 1.63 (m, 2H), 1.45 – 1.37 (m, 2H), 0.96 (t, *J* = 7.3 Hz, 3H). ^{13}C NMR (101 MHz, Methylene Chloride-d_2) δ 144.2, 138.4, 137.2, 133.6, 132.6, 131.5, 130.1, 128.9, 128.3, 127.5, 126.9, 124.2, 121.7, 120.6, 118.1, 110.9, 108.8, 107.6, 35.8, 34.1, 30.4, 22.9, 20.4, 14.2, 12.2. HRMS (ESI) calcd for [M+H] $C_{25}H_{29}N_2$, m/z: 357.2325, found: 357.2324. HPLC analysis: HPLC DAICEL CHIRALCEL OD-H, hexane/isopropanol = 99/1, 1.0 mL/min, λ = 229 nm, t_R (minor) = 13.491 min, t_R (major) = 15.169 min, er = 94.5:5.5. $[\alpha]_D^{25}$ = -12.273 (*c* = 0.220, CH_2Cl_2).

Chiral HPLC spectrum of (*rac*)-**24**

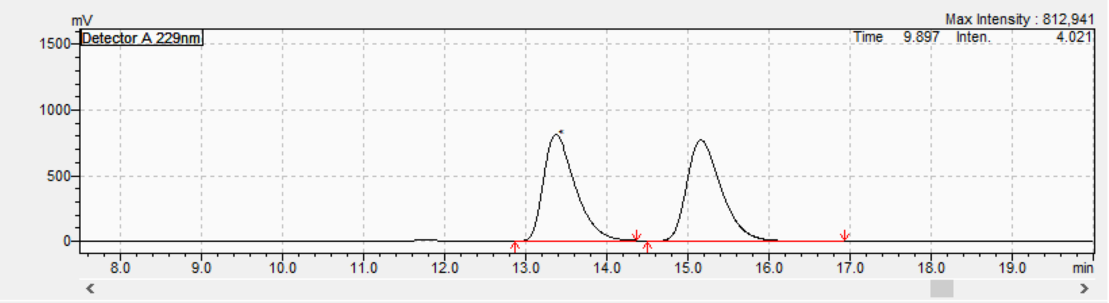

Results View - Peak Table

Peak Table | Compound | Group | Calibration Curve

Peak#	Ret. Time	Area	Height	Mark	Conc.	Area%
1	13.369	22426842	810782	M	49.820	49.820
2	15.165	22589002	767890	M	50.180	50.180
Total		45015844	1578672		100.000	100.000

Chiral HPLC spectrum of (*S*)-**24**

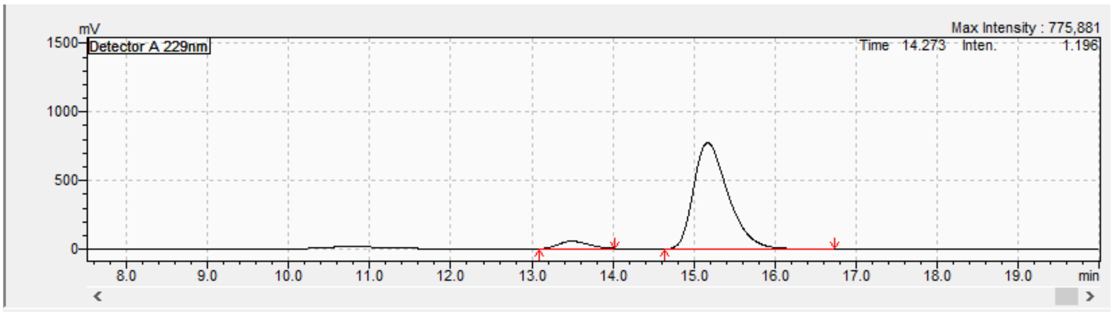

Results View - Peak Table

Peak Table | Compound | Group | Calibration Curve

Peak#	Ret. Time	Area	Height	Mark	Conc.	Area%
1	13.491	1397262	54583	M	5.727	5.727
2	15.169	23000838	774835	M	94.273	94.273
Total		24398100	829418		100.000	100.000

5. The transformation and application

The 2-arylindole products were synthetically manipulated. With the employment of 1.2 mmol azide, the axially chiral indole **3** was obtained in 55% yield. The anilide amino group in **3** provides a versatile handle for downstream derivatization. For example, thiourea catalyst **25** was prepared in high yield and with excellent ee. Alternatively, **3** was converted to iodide **26**, followed by a palladium-catalyzed phosphorylation and reduction, to furnish the axially chiral phosphine ligand **28**. In our preliminary evaluations, thiourea **25** and phosphine **28** promoted an asymmetric Mannich-type reaction of a phosphorus ylide with an N-Boc aldimine and a

palladium-catalyzed asymmetric allylic substitution of diethyl malonate respectively, each delivering products with certain level of asymmetry. These results highlight the potential of 2-arylindoles to serve as ligands or organocatalysts in asymmetric catalysis.

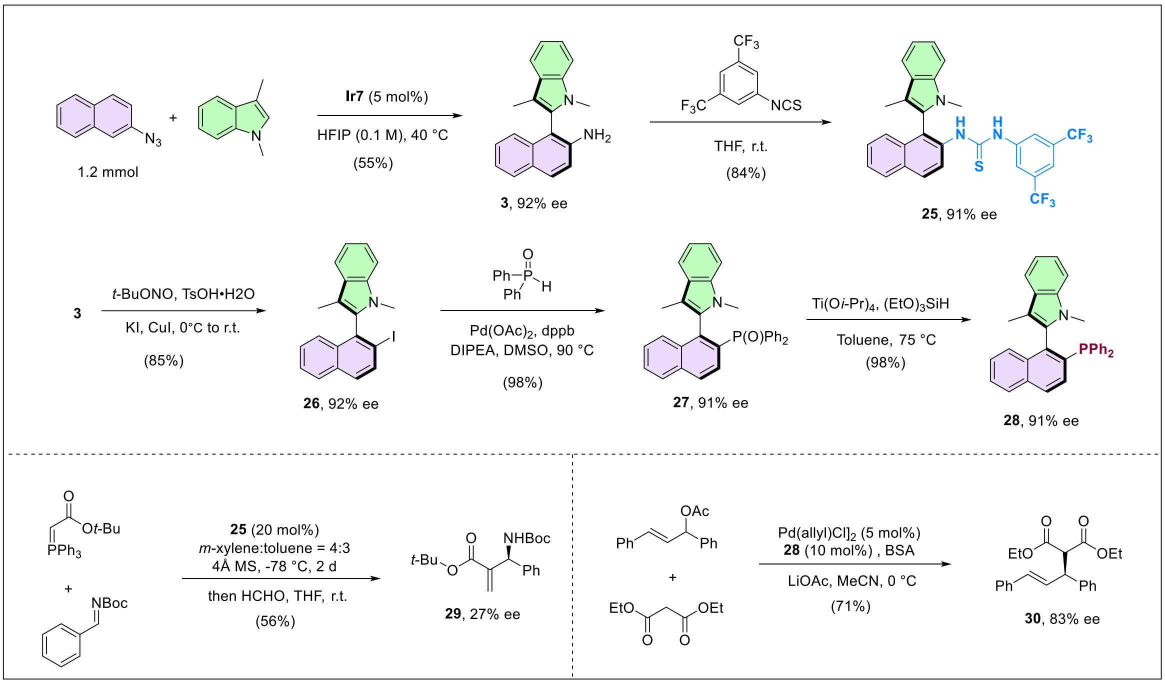

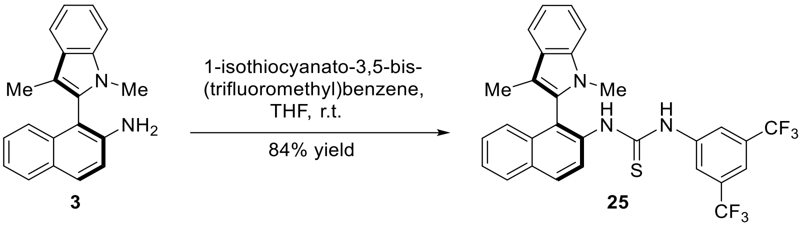

According to the literature[7], a 50 mL Schlenk tube was charged with naphthylamine **3** (0.20 mmol, 1.0 equiv), 1-isothiocyanato-3,5-bis(trifluoromethyl)benzene (0.22 mmol, 1.1 equiv), and THF (2 mL). The reaction mixture was stirred at room temperature for 24 h. The resulting mixture was diluted with DCM and filtered through a Celite pad. The filtrate was concentrated in vacuo, and the crude residue was purified by preparative TLC (hexane/EtOAc) to afford the desired product **25** (84% yield, 91% ee, yellow oil). ^{1}H NMR (400 MHz, Methylene Chloride-d_2) δ 8.19 (d, J = 8.9 Hz, 1H), 8.15 – 8.04 (m, 2H), 7.97 (dt, J = 8.2, 1.0

Hz, 1H), 7.89 (s, 1H), 7.83 – 7.76 (m, 2H), 7.61 – 7.51 (m, 3H), 7.48 – 7.41 (m, 1H), 7.39 – 7.31 (m, 2H), 7.27 (ddd, J = 8.2, 6.9, 1.2 Hz, 1H), 7.14 (ddd, J = 7.9, 6.9, 1.1 Hz, 1H), 3.36 (s, 3H), 2.03 (s, 3H). ^{13}C NMR (101 MHz, Methylene Chloride-d_2) δ 179.8, 139.7, 138.3, 135.7, 134.3, 132.7, 132.3, 130.6, 130.2, 128.8, 128.6, 128.2, 127.0, 126.5, 124.9, 123.6, 123.5 (q, J = 272.9 Hz), 123.0, 122.8, 119.8, 112.0, 110.0, 31.0, 9.5. ^{19}F NMR (377 MHz, Methylene Chloride-d_2) δ -63.22. HRMS (ESI) calcd for $[M+H]^+$ $C_{29}H_{22}F_6N_3S$, m/z: 558.1433, found: 558.1434. HPLC analysis: HPLC DAICEL CHIRALCEL OD-H, hexane/isopropanol = 95/5, 1.0 mL/min, λ = 254 nm, t_R (minor) = 5.872 min, t_R (major) = 6.495 min, er = 4.5:95.5. $[\alpha]^{25}_{D}$ = -30.000 (c = 0.170, CH_2Cl_2).

Chiral HPLC spectrum of (*rac*)-**25**

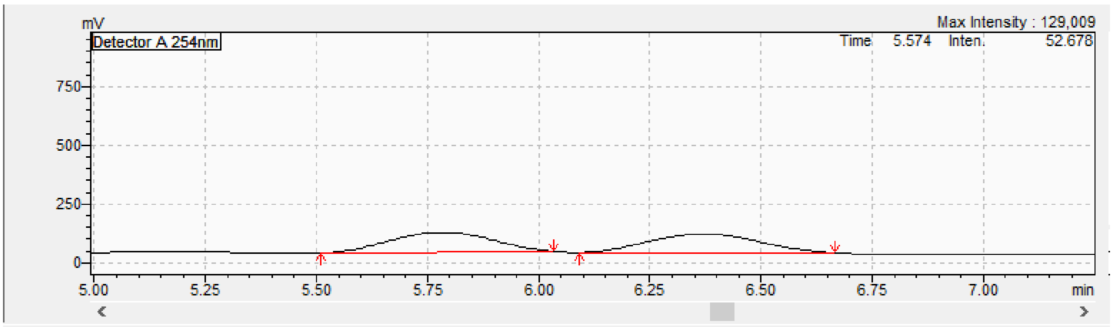

Results View - Peak Table

Peak Table | Compound | Group | Calibration Curve

Peak#	Ret. Time	Area	Height	Mark	Conc.	Area%
1	5.785	1348334	84082	M	49.755	49.755
2	6.369	1361595	79214	M	50.245	50.245
Total		2709929	163296		100.000	100.000

Chiral HPLC spectrum of (*S*)-**25**

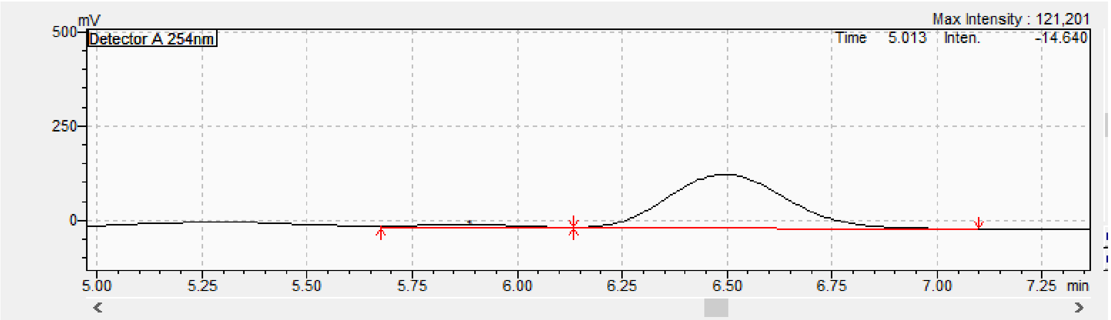

Results View - Peak Table

Peak Table | Compound | Group | Calibration Curve

Peak#	Ret. Time	Area	Height	Mark	Conc.	Area%
1	5.872	125112	6940	M	4.357	4.357
2	6.495	2746614	141697	M	95.643	95.643
Total		2871725	148637		100.000	100.000

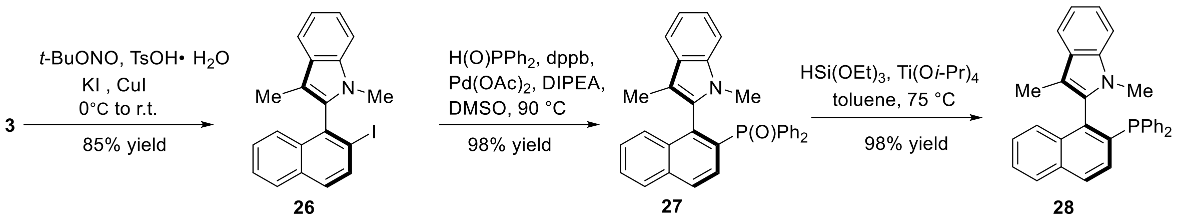

To a solution of the naphthylamine substrate **3** in MeCN was added PTSA·H_2O (1.0 equiv). The mixture was cooled to 0 °C, and *t*BuONO (3.0 equiv) was added dropwise. After stirring for 10 min at 0 °C, KI (3.0 equiv) and CuI (0.1 equiv) were added. The reaction mixture was stirred at 0 °C for an additional 10 min, then warmed to room temperature and stirred for 2 h. Upon completion of the reaction (monitored by TLC), the mixture was quenched with aqueous Na_2SO_3 followed by saturated $NaHCO_3$. The resulting mixture was extracted with EtOAc, and the combined organic layers were dried, filtered, and concentrated in vacuo. The crude residue was purified by column chromatography on silica gel (hexane/EtOAc = 10:1) to afford the desired product **26** (85% yield, 92% ee, yellow oil). ^{1}H NMR (500 MHz, CD_2Cl_2) δ 8.04 (d, *J* = 8.7 Hz, 1H), 7.93 (d, *J* = 8.2 Hz, 1H), 7.72 (d, *J* = 8.7 Hz, 1H), 7.67 (d, *J* = 7.9 Hz, 1H), 7.56 – 7.52 (m, 1H), 7.39 (dd, *J* = 13.8, 7.4 Hz, 2H), 7.33 (d, *J* = 8.5 Hz, 1H), 7.31 – 7.26 (m, 1H), 7.18 (t, *J* = 7.5 Hz, 1H), 3.38 (s, 3H), 2.06 (s, 3H). ^{13}C NMR (126 MHz, CD_2Cl_2)

δ 138.1, 137.3, 135.8, 135.6, 134.9, 133.1, 130.8, 128.5, 127.9, 127.0, 126.8, 126.4, 122.1, 119.3 (2C), 109.9, 109.6, 102.1, 30.5, 9.1. HRMS (ESI) calcd for $[M+H]^+$ $C_{20}H_{17}IN$, m/z: 398.04, found: 398.0397. HPLC analysis: HPLC DAICEL CHIRALCEL OJ-H, hexane/isopropanol = 99/1, 0.5 mL/min, λ = 229 nm, t_R (major) = 16.997 min, t_R (minor) = 21.268 min, er = 96:4. $[\alpha]_D^{25}$ = 5.660 (c = 0.530, CH_2Cl_2).

Chiral HPLC spectrum of (*rac*)-**26**

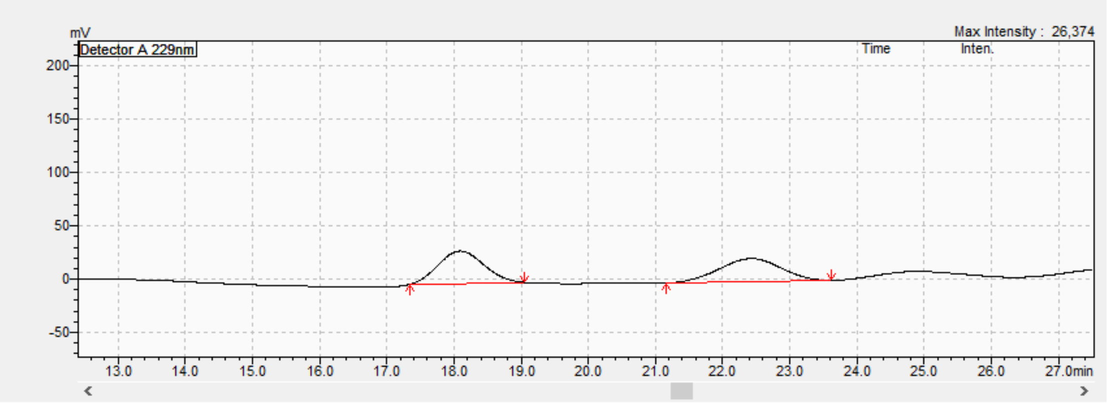

Results View - Peak Table

Peak Table | Compound | Group | Calibration Curve

Peak#	Ret. Time	Area	Height	Mark	Conc.	Area%
1	18.092	1425825	30325	M	50.328	50.328
2	22.425	1407226	21770	M	49.672	49.672
Total		2833051	52094		100.000	100.000

Chiral HPLC spectrum of (*S*)-**26**

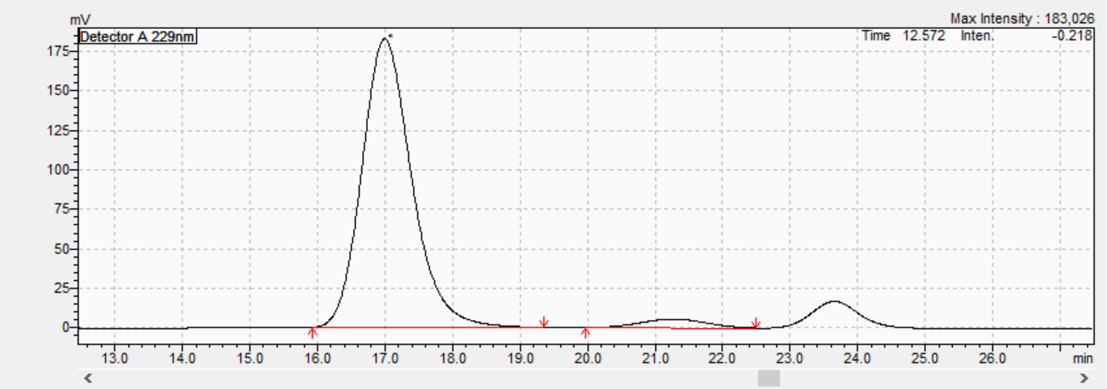

Results View - Peak Table

Peak Table | Compound | Group | Calibration Curve

Peak#	Ret. Time	Area	Height	Mark	Conc.	Area%
1	16.997	9405363	182912	M	96.050	96.050
2	21.268	386783	5657	M	3.950	3.950
Total		9792146	188569		100.000	100.000

According to the literature[8], a flame-dried Schlenk flask was charged with compound **26**, $H(O)PPh_2$ (2.0 equiv), $Pd(OAc)_2$ (20 mol%), dppb (20 mol%), and DIPEA (4.0 equiv), and the mixture was dissolved in dry DMSO under an argon atmosphere. The reaction mixture was stirred at 90 °C for 24 h. After cooling to room temperature, water was added, and the mixture was extracted with EtOAc. The combined organic layers were dried over anhydrous Na_2SO_4, filtered, concentrated in vacuo, and purified by flash chromatography on silica gel to afford the desired product **27** (98% yield, 91% ee, yellow oil). ^{1}H NMR (600 MHz, CD_2Cl_2) δ 8.04 – 8.00 (m, 1H), 7.97 (dt, *J* = 8.3, 1.0 Hz, 1H), 7.66 (dd, *J* = 11.4, 8.6 Hz, 1H), 7.62 – 7.58 (m, 1H), 7.58 – 7.54 (m, 2H), 7.47 – 7.32 (m, 8H), 7.25 – 7.16 (m, 5H), 7.08 – 7.05 (m, 1H), 3.21 (s, 3H), 1.53 (s, 3H). ^{13}C NMR (151 MHz, CD_2Cl_2) δ 137.3, 136.5 (two signals overlap), 135.0 (two signals overlap), 134.6, 134.5 (two signals overlap), 133.8, 133.6, 132.6 (two signals overlap), 132.2, 132.0 (two signals overlap), 131.7 (two signals overlap), 131.6, 131.5, 129.1, 129.0 (two signals overlap), 128.9, 128.7, 128.6, 128.5, 128.4, 128.3, 128.2 (two signals overlap), 128.0, 127.0, 121.8, 119.0, 118.9, 111.6, 109.5, 30.8, 9.1. ^{31}P NMR (243 MHz, CD_2Cl_2) δ 26.64. HRMS (ESI) calcd for $[M+H]^+$ $C_{32}H_{27}NOP$, m/z: 472.1825, found: 472.1824. HPLC analysis: HPLC DAICEL CHIRALCEL IA, hexane/isopropanol = 90/10, 1.0 mL/min, λ = 229 nm, t_R (minor) = 14.131 min, t_R (major) = 21.823 min, er = 4.5:95.5. $[\alpha]_D^{25}$ = 5.000 (*c* = 0.100, CH_2Cl_2).

Chiral HPLC spectrum of (*rac*)-**27**

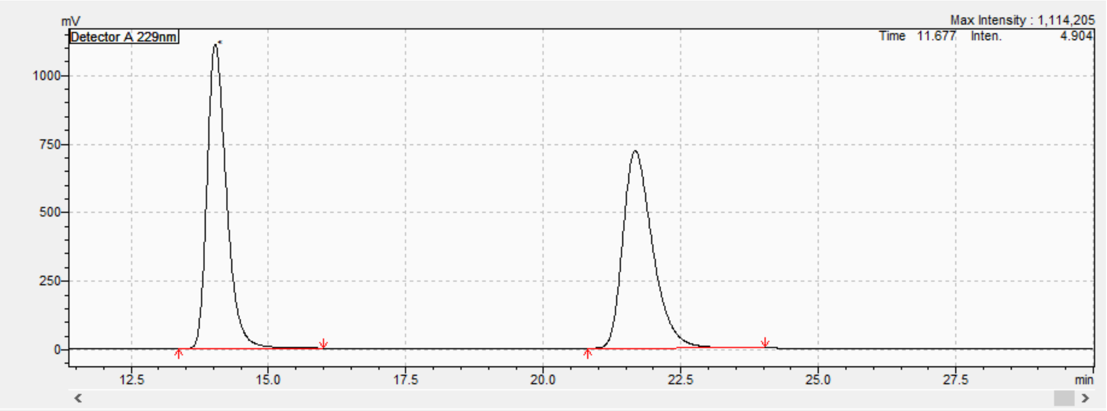

Results View - Peak Table

Peak Table | Compound | Group | Calibration Curve

Peak#	Ret. Time	Area	Height	Mark	Conc.	Area%
1	14.033	27132233	1108784	M	50.023	50.023
2	21.670	27106758	719113	M	49.977	49.977
Total		54238991	1827897		100.000	100.000

Chiral HPLC spectrum of (*S*)-**27**

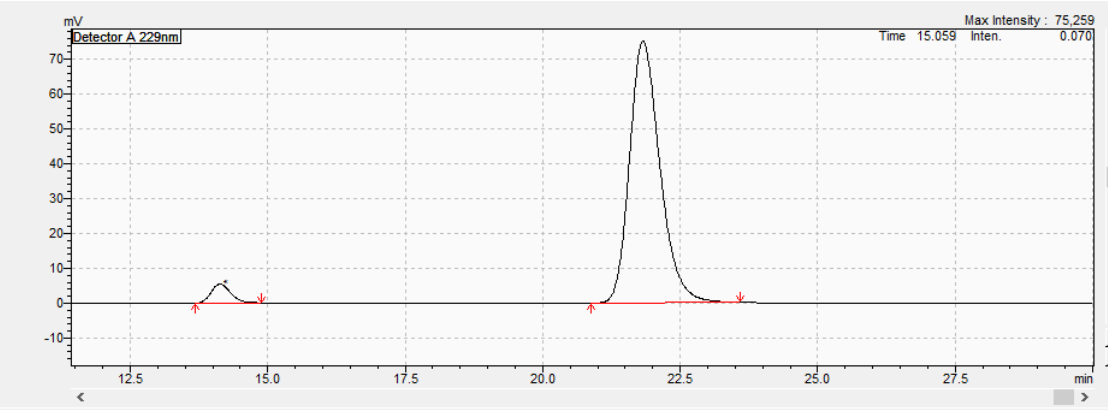

Results View - Peak Table

Peak Table | Compound | Group | Calibration Curve

Peak#	Ret. Time	Area	Height	Mark	Conc.	Area%
1	14.131	135833	5476	M	4.489	4.489
2	21.823	2890334	75161	M	95.511	95.511
Total		3026167	80637		100.000	100.000

According to the literature[9], a 10 mL Schlenk tube was charged with compound **27**, Ti(Oi-Pr)$_4$ (0.3 equiv), SiH(OEt)$_3$ (3.0 equiv), and toluene under an Ar atmosphere at 0 °C. The reaction mixture was then heated to 75 °C and stirred until complete consumption of the starting material (monitored by TLC). After cooling to room temperature, the reaction mixture was diluted with Et$_2$O and quenched with saturated NaHCO$_3$. The resulting suspension was filtered, and the filtrate was dried over MgSO$_4$, filtered again, and concentrated under reduced pressure. The crude residue was purified by column

chromatography on silica gel to afford compound **28** (98% yield, 91% ee, colorless oil). ^{1}H NMR (500 MHz, CD_2Cl_2) δ 7.94 – 7.90 (m, 2H), 7.58 – 7.50 (m, 2H), 7.40 – 7.27 (m, 10H), 7.26 – 7.18 (m, 5H), 7.16 – 7.12 (m, 1H), 3.20 (s, 3H), 1.76 (s, 3H). ^{13}C NMR (126 MHz, CD_2Cl_2) δ 139.0, 138.9, 138.2, 138.1, 137.6, 137.5, 137.3, 137.2, 134.9, 134.8, 134.2 (two signals overlap), 134.1, 134.0 (two signals overlap), 133.9, 130.0, 129.4, 128.9 (two signals overlap), 128.8 (four signals overlap), 128.6, 128.4, 127.4 (two signals overlap), 126.6 (two signals overlap), 121.7, 119.1, 119.0, 111.3 (two signals overlap), 109.5, 30.5 (two signals overlap), 9.2 (two signals overlap) ^{31}P NMR (202 MHz, CD_2Cl_2) δ -12.93. HPLC analysis: HPLC DAICEL CHIRALCEL IA, hexane/isopropanol = 85/15, 1.0 mL/min, λ = 229 nm, t_R (minor) = 9.676 min, t_R (major) = 14.104 min, er = 4.5:95.5. $[\alpha]_D^{25}$ = 20.750 (*c* = 0.400, CH_2Cl_2).

Chiral HPLC spectrum of (*rac*)-**28**

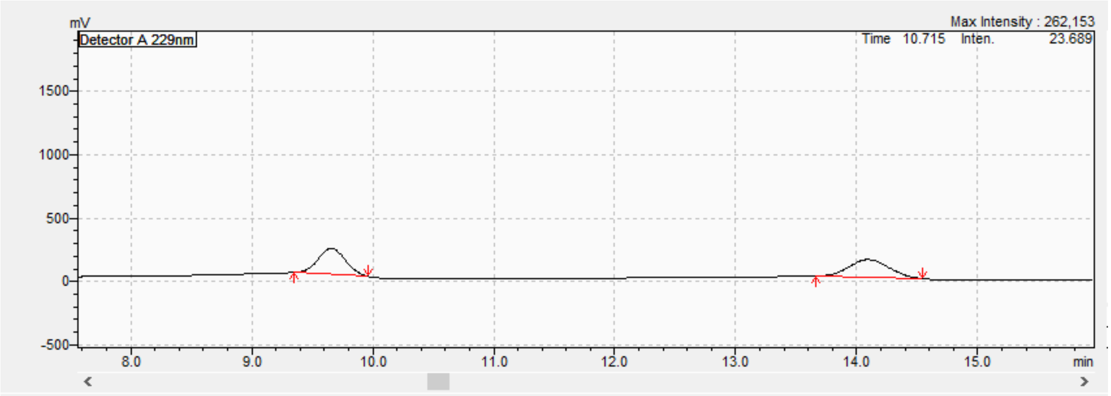

Results View - Peak Table

Peak Table | Compound | Group | Calibration Curve

Peak#	Ret. Time	Area	Height	Mark	Conc.	Area%
1	9.656	3235060	205878	M	49.784	49.784
2	14.094	3263162	140654	M	50.216	50.216
Total		6498222	346532		100.000	100.000

Chiral HPLC spectrum of (*S*)-**28**

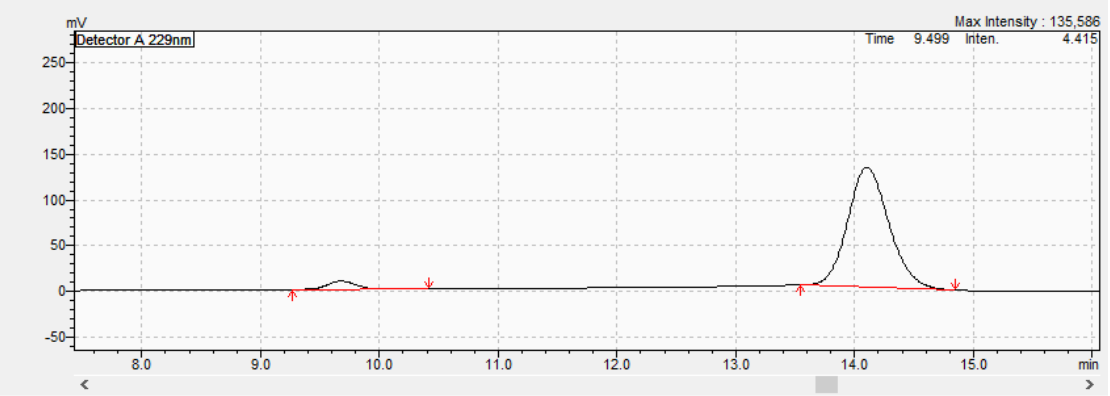

Results View - Peak Table

Peak Table | Compound | Group | Calibration Curve

Peak#	Ret. Time	Area	Height	Mark	Conc.	Area%
1	9.676	143611	8927	M	4.327	4.327
2	14.104	3175156	130804	M	95.673	95.673
Total		3318767	139732		100.000	100.000

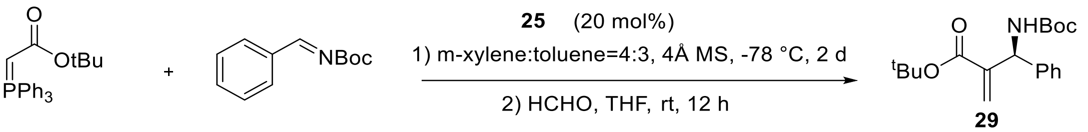

29

56% yield, 27% ee

According to a modified literature procedure[8], catalyst **25** (20 mol%), ylide (1.0 equiv), and 4 Å molecular sieves (400 mg per 1 mmol of ylide) were stirred in a mixture of m-xylene and toluene (4:3) at –78 °C. *N*-Boc imine (2.0 equiv) was then added, and the reaction mixture was stirred at –78 °C for 2 days. The crude product from the first step was rapidly isolated by flash column chromatography. The isolated intermediate was dissolved in THF, followed by the addition of formalin (35%, 2 mL per 1 mmol of intermediate). The solution was stirred at room temperature for 12 h. The reaction mixture was poured into water and extracted with EtOAc. The combined organic extracts were dried over Na_2SO_4, concentrated under reduced pressure, and purified by flash column chromatography to afford product **29** (56% yield, 27% ee, yellow oil). ^{1}H NMR (500 MHz, $CDCl_3$) δ 7.36 – 7.23 (m, 5H), 6.31 (s, 1H), 5.80 (s, 1H), 5.66 (d, J = 8.9 Hz, 1H), 5.34 (d, J = 8.9 Hz, 1H), 1.48 (s, 9H), 1.33 (s, 9H). ^{13}C NMR (126 MHz, $CDCl_3$) δ 165.0, 154.9, 128.5 (3C), 127.4, 126.7, 125.1, 81.4, 79.7, 56.1, 28.4 (3C),

27.8 (3C). HPLC analysis: HPLC DAICEL CHIRALCEL IC, hexane/isopropanol = 99/1, 1.0 mL/min, λ = 210 nm, t_R (minor) = 13.251 min, t_R (major) = 15.510 min, er = 36.5:63.5. $[\alpha]_D^{25}$ = 5.333 (c = 2.250, $CHCl_3$).

Chiral HPLC spectrum of (*rac*)-**29**

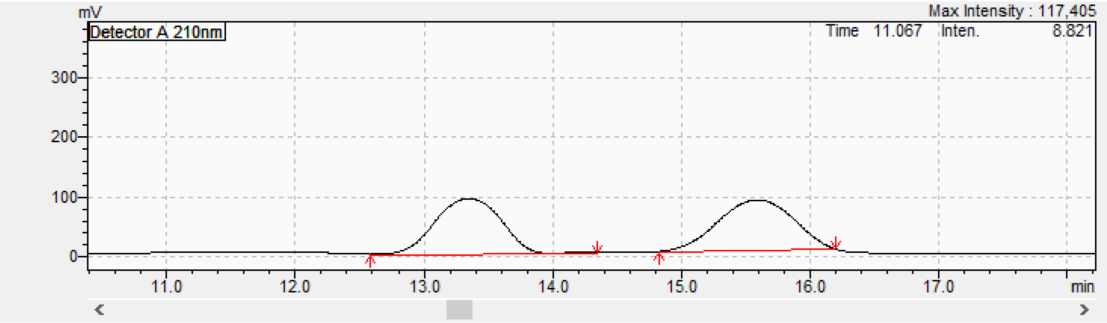

Results View - Peak Table

Peak Table | Compound | Group | Calibration Curve

Peak#	Ret. Time	Area	Height	Mark	Conc.	Area%
1	13.344	3413414	93444	M	50.084	50.084
2	15.590	3401950	83815	M	49.916	49.916
Total		6815364	177260		100.000	100.000

Chiral HPLC spectrum of (*S*)-**29**

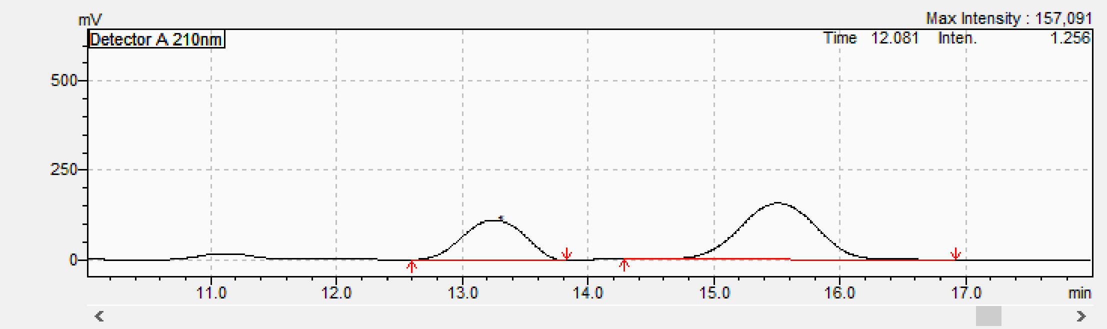

Results View - Peak Table

Peak Table | Compound | Group | Calibration Curve

Peak#	Ret. Time	Area	Height	Mark	Conc.	Area%
1	13.251	3855693	111643	M	36.703	36.703
2	15.510	6649476	157058	M	63.297	63.297
Total		10505169	268701		100.000	100.000

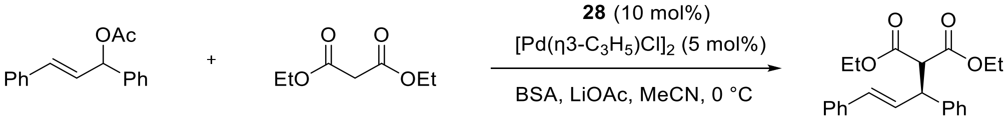

30

71% yield, 83% ee

According to a modified literature procedure[8], a mixture of chiral ligand **28** (10 mol%), $[Pd(\eta^3\text{-}C_3H_5)Cl]_2$ (5 mol%), and LiOAc (8 mol%) in MeCN was prepared under an argon atmosphere at 0 °C. BSA (3.0 equiv) and allylic ester (1.0 equiv) were added to the mixture and stirred for 30 min. Malonate (3.0 equiv) was then added, and the reaction was allowed to proceed for 12 h at 0 °C. The reaction mixture was diluted with EtOAc and water. The organic layer was separated, washed with brine, and dried over Na_2SO_4. Filtration and concentration under reduced pressure followed by purification by flash column chromatography afforded the desired product **30** (71% yield, 83% ee, yellow oil). ^{1}H NMR (500 MHz, $CDCl_3$) δ 7.35 – 7.20 (m, 10H), 6.50 (d, *J* = 15.8 Hz, 1H), 6.36 (dd, *J* = 15.7, 8.6 Hz, 1H), 4.29 (ddd, *J* = 11.0, 8.6, 0.9 Hz, 1H), 4.20 (q, *J* = 7.1 Hz, 2H), 4.06 – 3.96 (m, 2H), 3.94 (d, *J* = 11.0 Hz, 1H), 1.23 (t, *J* = 7.1 Hz, 3H), 1.04 (t, *J* = 7.1 Hz, 3H). ^{13}C NMR (126 MHz, $CDCl_3$) δ 167.8, 167.4, 140.3, 136.8, 131.7, 129.3, 128.6, 128.4, 128.0, 127.5, 127.1, 126.3, 61.6, 61.4, 57.8, 49.2, 14.1, 13.8. HPLC analysis: HPLC DAICEL CHIRALCEL AD-H, hexane/isopropanol = 93/7, 1.0 mL/min, λ = 254 nm, t_R (minor) = 9.283 min, t_R (major) = 11.901 min, er = 8.5:91.5. $[\alpha]_D^{25}$ = -8.689 (*c* = 1.220, $CHCl_3$).

Chiral HPLC spectrum of (*rac*)-**30**

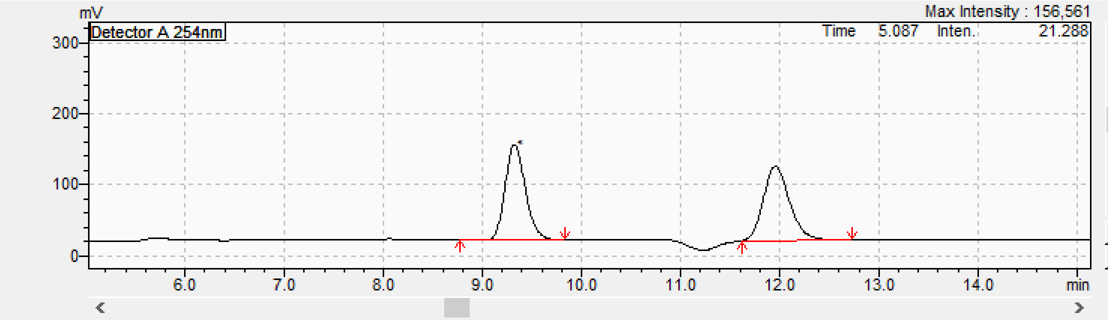

Results View - Peak Table

Peak Table | Compound | Group | Calibration Curve

Peak#	Ret. Time	Area	Height	Mark	Conc.	Area%
1	9.322	1908535	134539	M	49.938	49.938
2	11.961	1913249	104553	M	50.062	50.062
Total		3821784	239092		100.000	100.000

Chiral HPLC spectrum of (*S*)-**30**

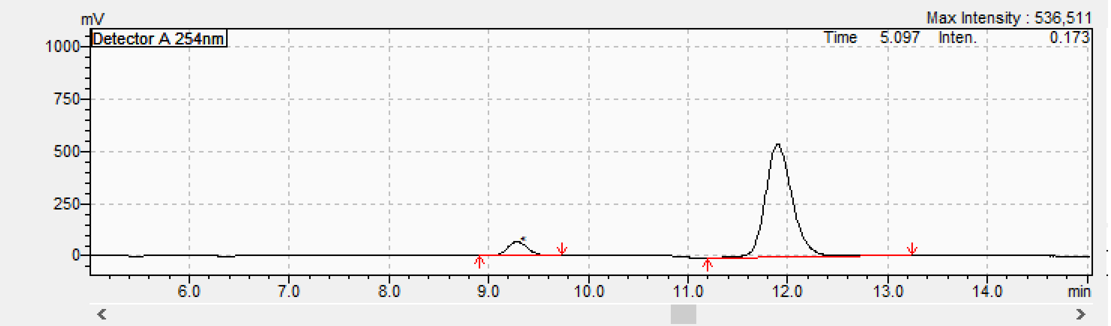

Results View - Peak Table

Peak Table | Compound | Group | Calibration Curve

Peak#	Ret. Time	Area	Height	Mark	Conc.	Area%
1	9.283	967928	68628	M	8.452	8.452
2	11.901	10483485	545599	S	91.548	91.548
Total		11451412	614227		100.000	100.000

6. Racemization experiment and calculation of half-life of 3

The enantiomerisation barrier was obtained by kinetic of racemisation of an enantiomer. The slope of the first order kinetic line gives the racemisation constant ($k_{racemisation}$ = 2 * $k_{enantiomerisation}$). Eyring equation gives the enantiomerisation barrier ($\Delta G‡_{enaniomerization}$) from enantiomerisation constant ($k_{enantiomerisation}$), R = 8.31451 J.K^{-1}mol^{-1}, h = 6.62608 * 10^{-34} Js and k_B = 1.38066 *10^{-23} J/K. Enantiomeric excess values were determined by HPLC.

Table S1. Racemization of **3** in mesitylene at 130 °C.

t (h)	ee (%)	ln (ee)
0	93	4.532599493
3	90	4.49980967
26	75	4.317488114
30	72	4.276666119
47	62	4.127134385
51	60	4.094344562

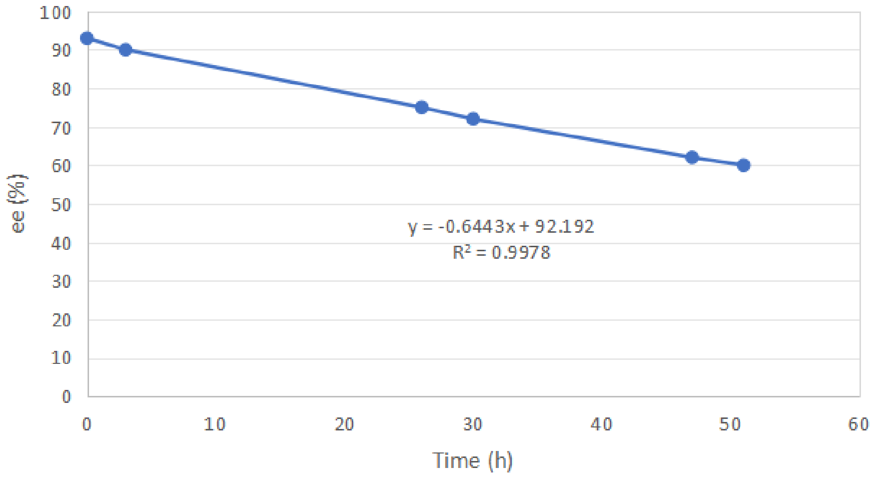

Figure S1. Graph of ee against time in the racemization of **3** in mesitylene at 130 °C.

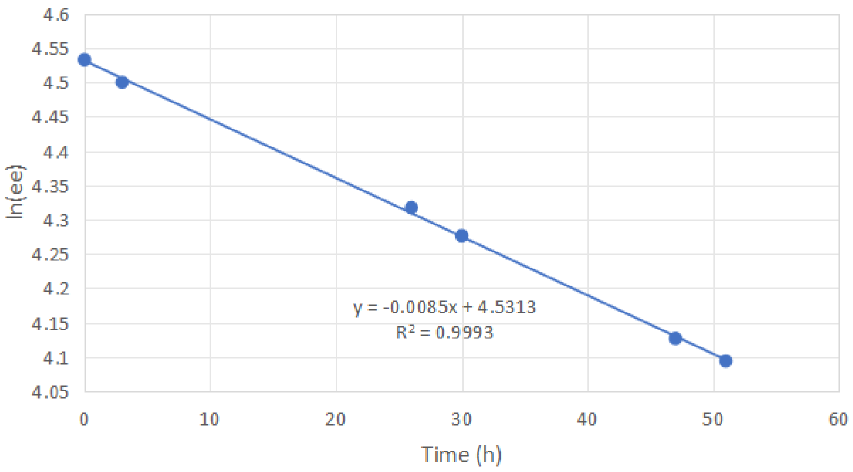

Figure S2. Graph of ln(ee) against time in the racemization of **3** in mesitylene at 130 °C.

At 130 °C, k_{rac} = 0.0085 h^{-1} , and the calculated racemization half-life was $t_{1/2}$ = ln2/K_{rac} = 81.5 h. Using the Eyring equation, the corresponding enantiomerization barrier was calculated to be $\Delta G^{\ddagger}$ = 34.8 kcal mol^{-1}. The estimated racemization half-life at 25 °C was

calculated to be 5.48 × 10^{4} years.

7. X-ray data

The crystal structure of compound **9** (CCDC 2488280) has been deposited at the Cambridge Crystallographic Data Centre.

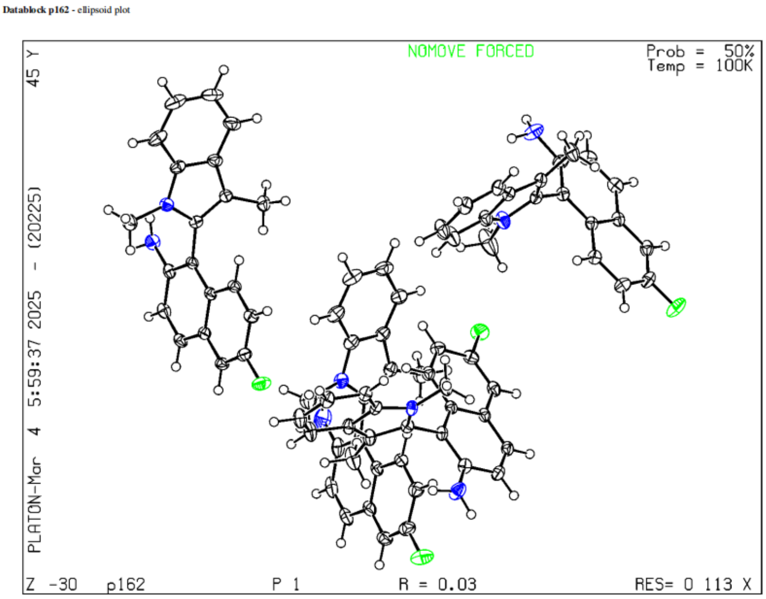

Table S2. Crystal data and structure refinement for compound **9**

Identification code	p162	
Empirical formula	C20 H17 F N2	
Formula weight	304.35	
Temperature	100.00 K	
Wavelength	1.54178 Å	
Crystal system	Triclinic	
Space group	P1	
Unit cell dimensions	a = 11.4214(8) Å	a= 89.199(3)°.
	b = 11.5908(9) Å	b= 73.066(3)°.
	c = 13.0500(10) Å	g = 75.686(3)°.
Volume	1598.2(2) Å^3	
Z	4	
Density (calculated)	1.265 Mg/m^3	
Absorption coefficient	0.664 mm^{-1}	

F(000)	640
Crystal size	0.173 x 0.134 x 0.089 mm^3
Theta range for data collection	3.547 to 66.825°.
Index ranges	-13<=h<=13, -13<=k<=13, -15<=l<=15
Reflections collected	140756
Independent reflections	10966 [R(int) = 0.0278]
Completeness to theta = 66.825°	99.4 %
Absorption correction	Semi-empirical from equivalents
Max. and min. transmission	0.7528 and 0.7110
Refinement method	Full-matrix least-squares on F^2
Data / restraints / parameters	10966 / 31 / 861
Goodness-of-fit on F^2	1.035
Final R indices [I>2sigma(I)]	R1 = 0.0275, wR2 = 0.0740
R indices (all data)	R1 = 0.0275, wR2 = 0.0741
Absolute structure parameter	-0.03(2)
Extinction coefficient	n/a
Largest diff. peak and hole	0.164 and -0.164 e.$Å^{-3}$

8. Copies of NMR spectra

^{1}H NMR spectra of **L7** (600 MHz, $CDCl_3$)

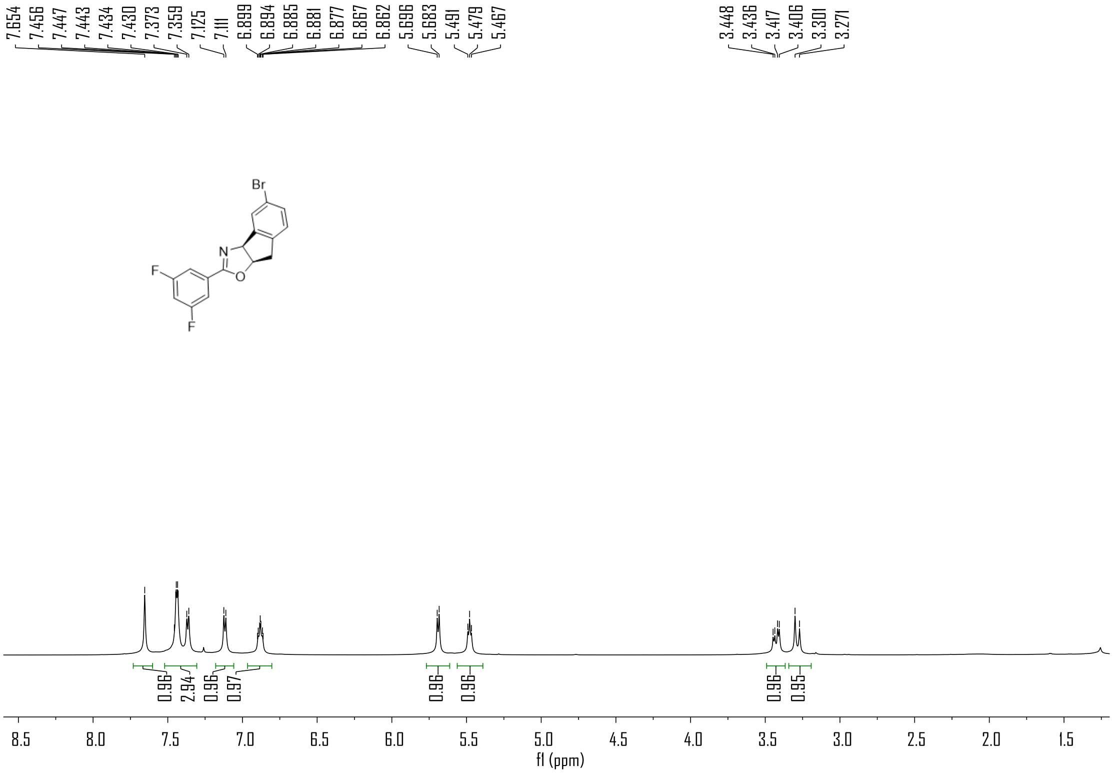

^{13}C NMR spectra of **L7** (126 MHz, $CDCl_3$)

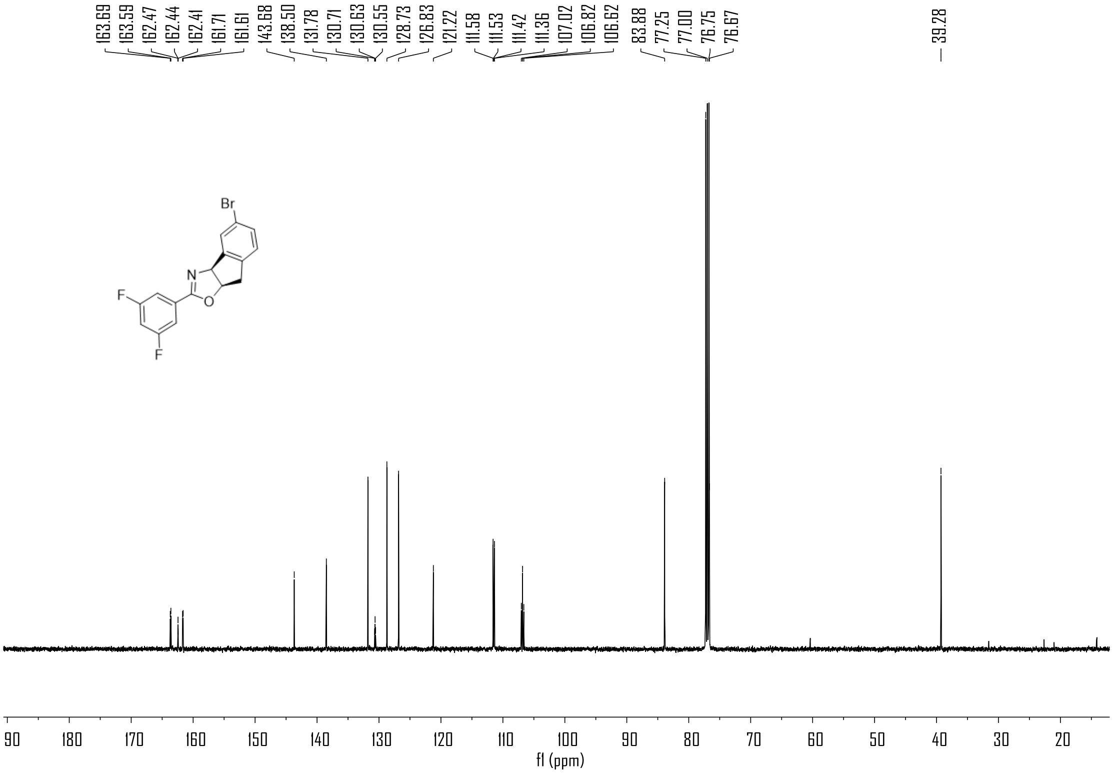

^{19}F NMR spectra of **L7** (471 MHz, $CDCl_3$)

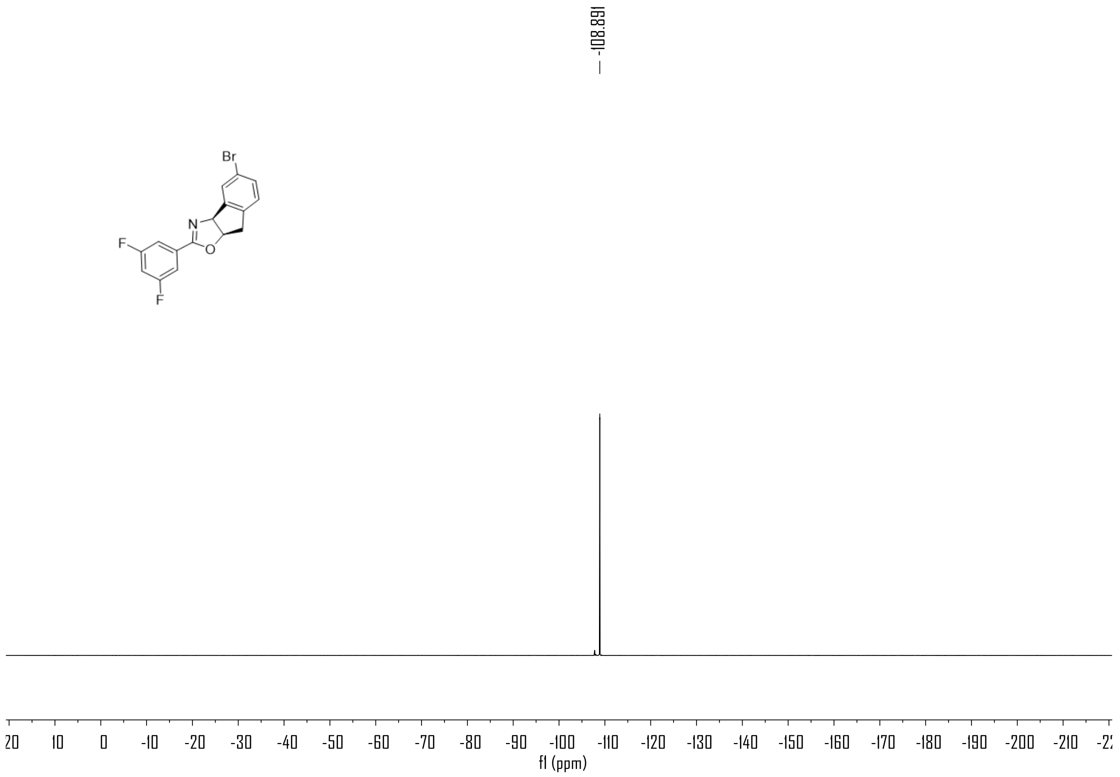

^{1}H NMR spectra of **L10** (500 MHz, $CDCl_3$)

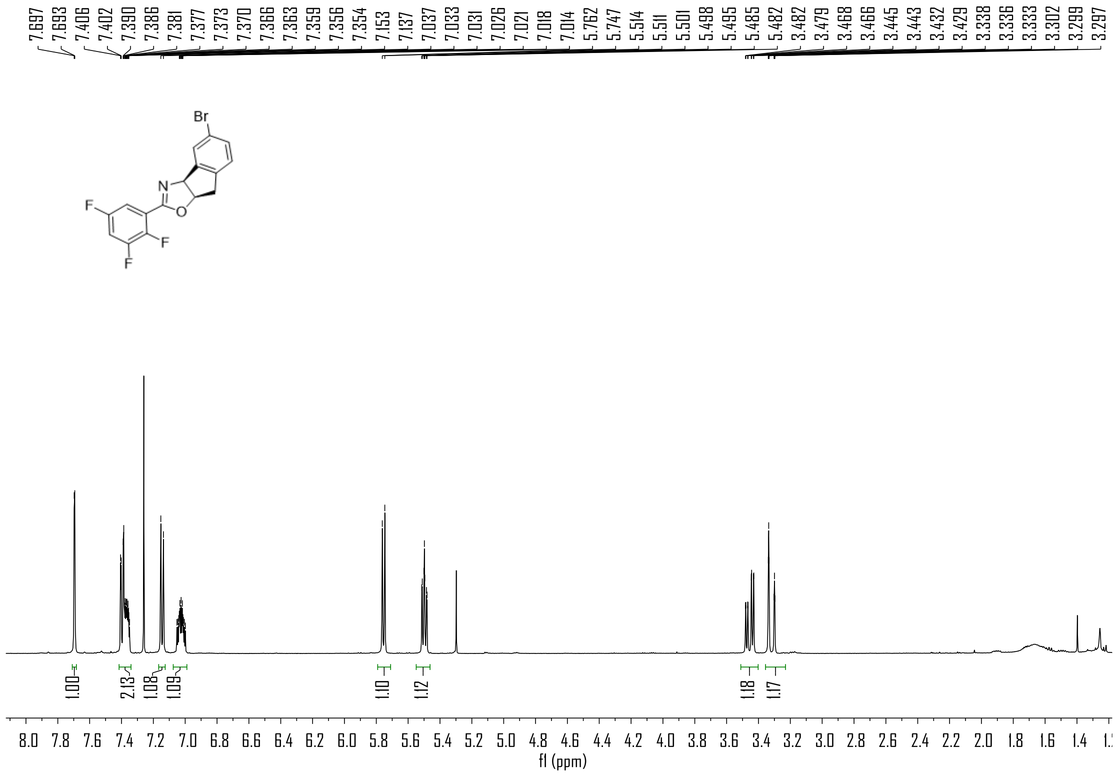

^{13}C NMR spectra of **L10** (126 MHz, $CDCl_3$)

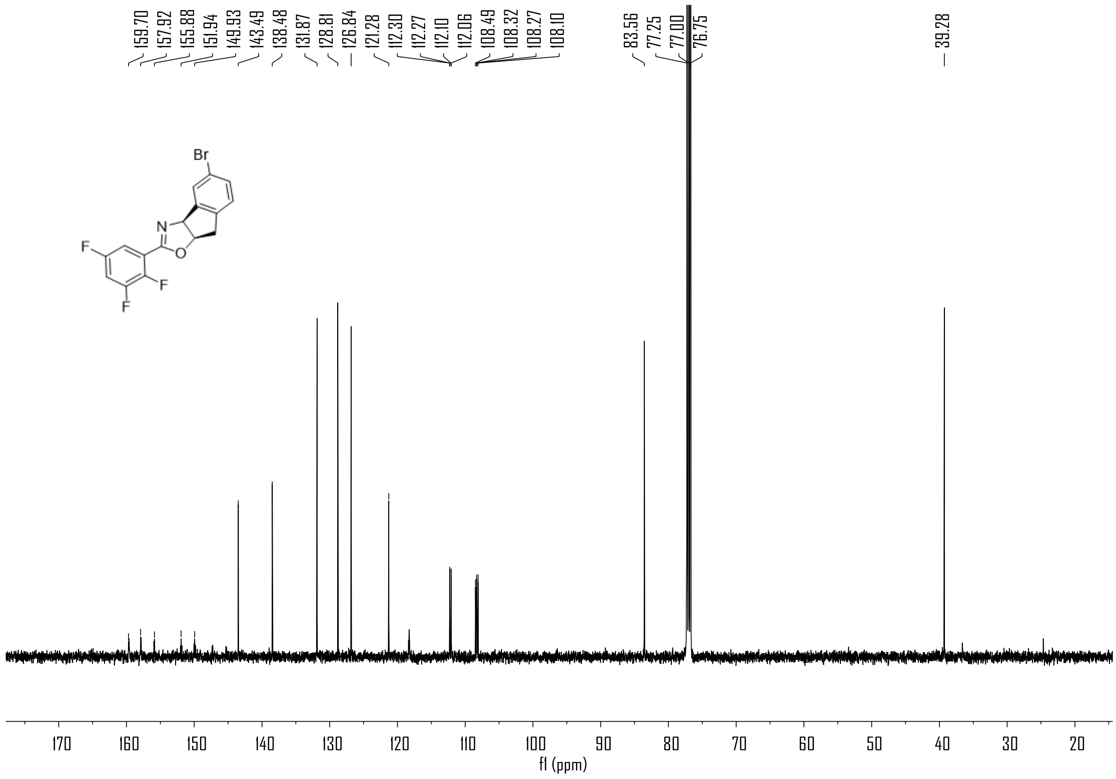

^{19}F NMR spectra of **L10** (565 MHz, $CDCl_3$)

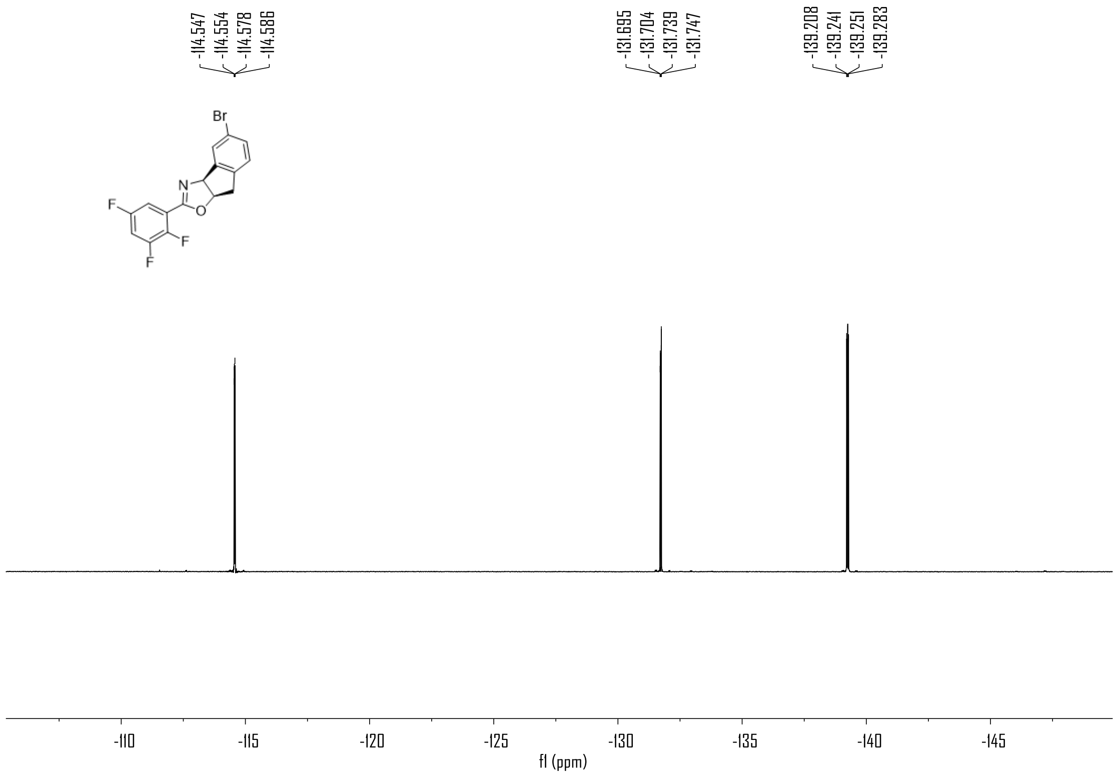

^{1}H NMR spectra of **L11** (500 MHz, $CDCl_3$)

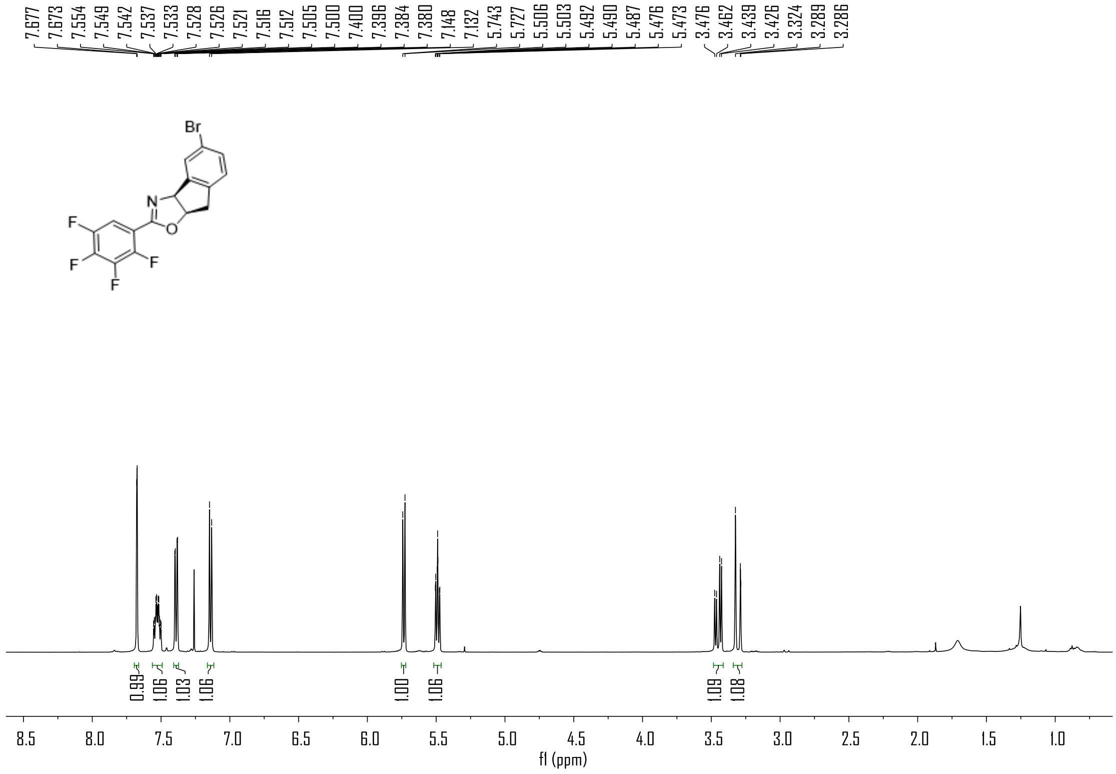

^{13}C NMR spectra of **L11** (126 MHz, $CDCl_3$)

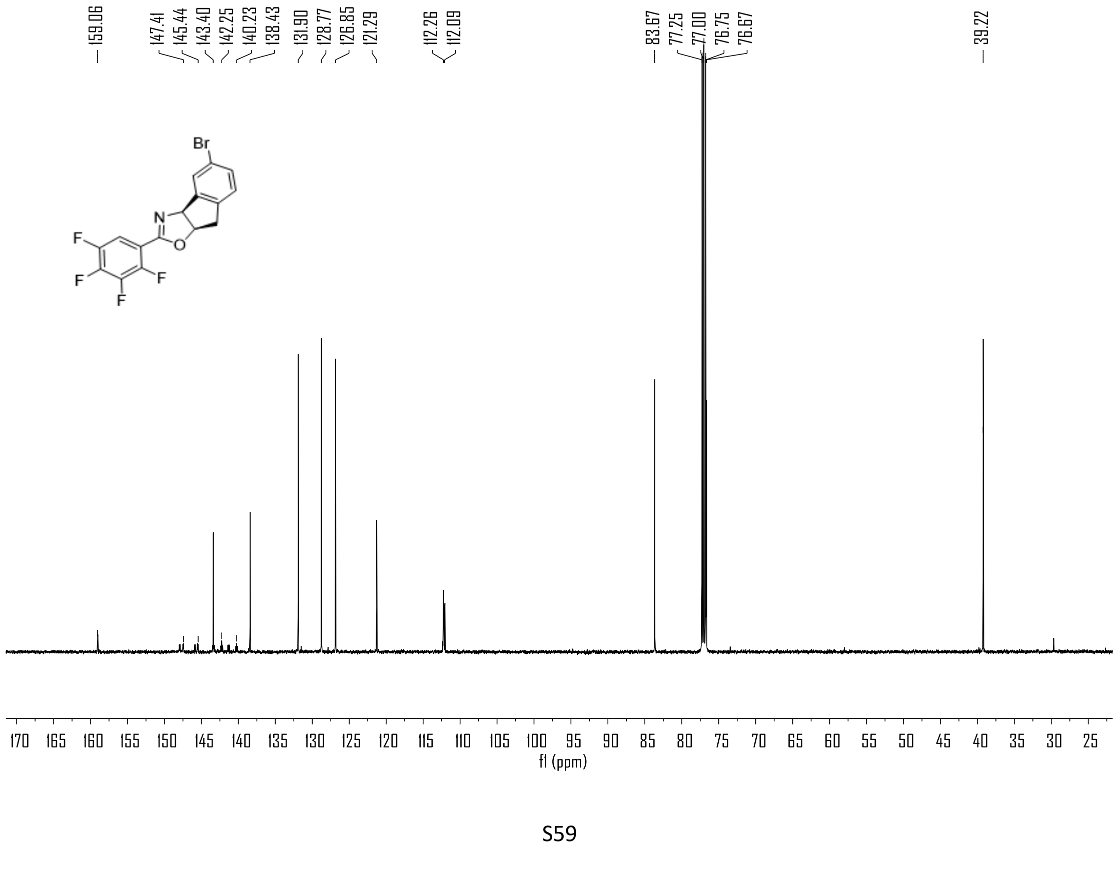

^{19}F NMR spectra of **L11** (471 MHz, $CDCl_3$)

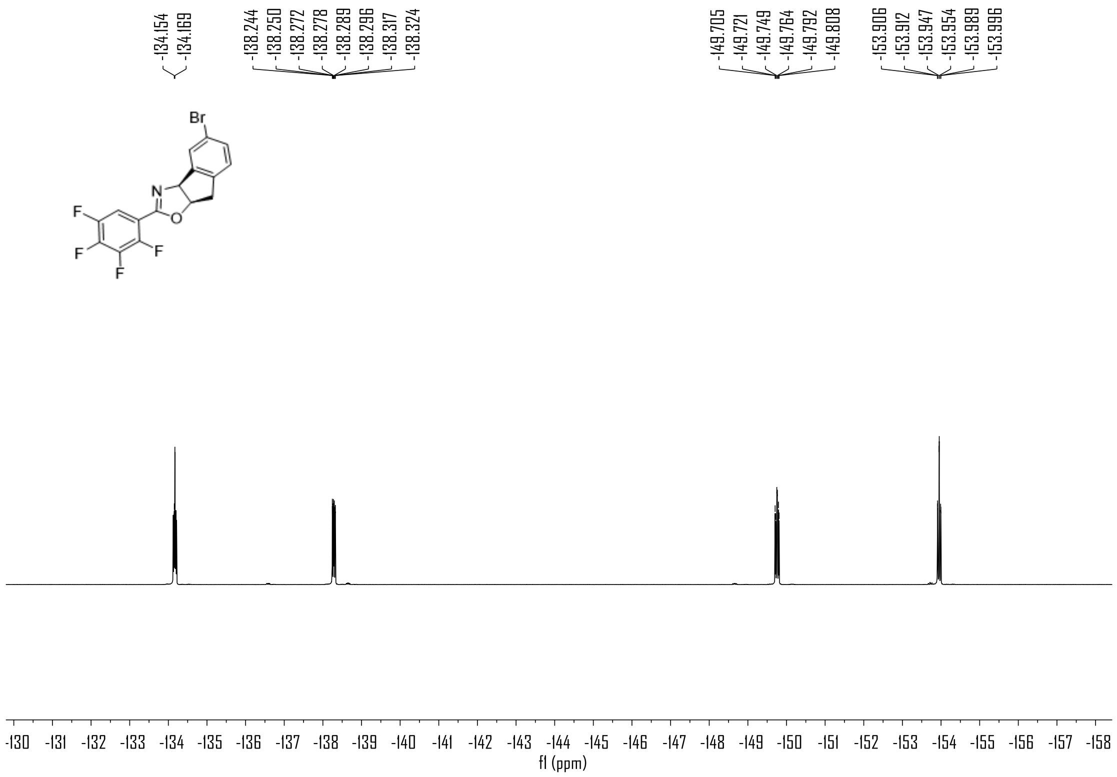

^{1}H NMR spectra of **Ir4** (600 MHz, $CDCl_3$)

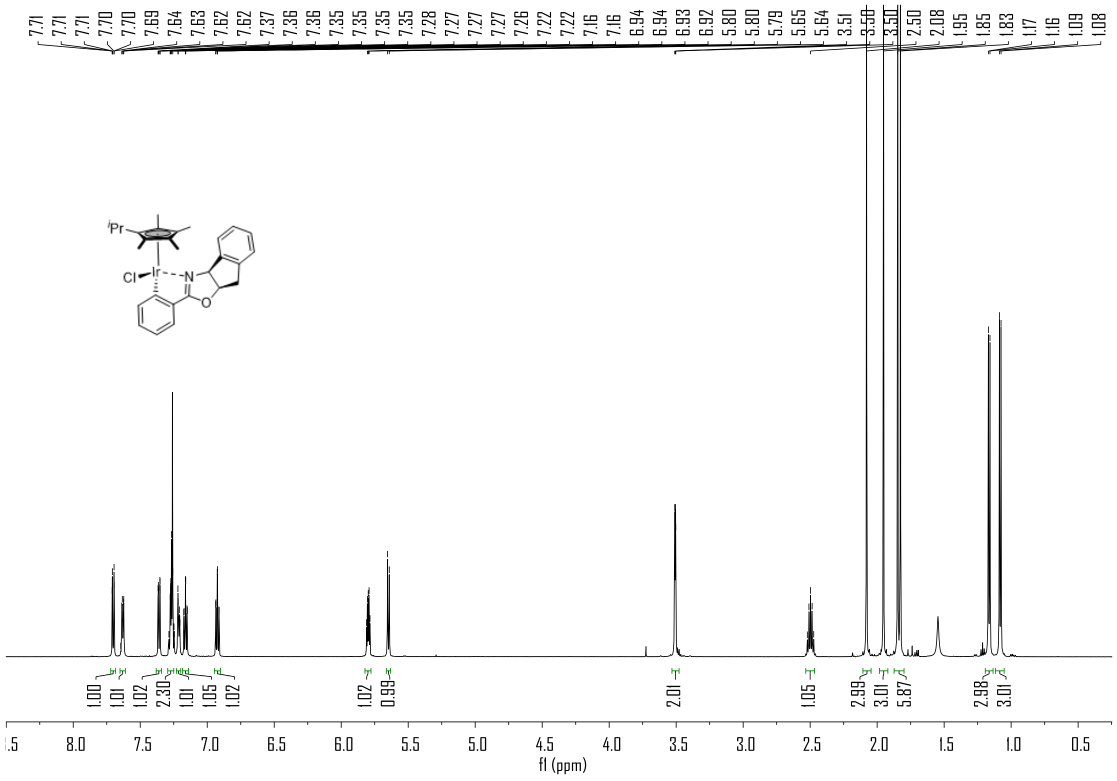

^{13}C NMR spectra of **Ir4** (151 MHz, $CDCl_3$)

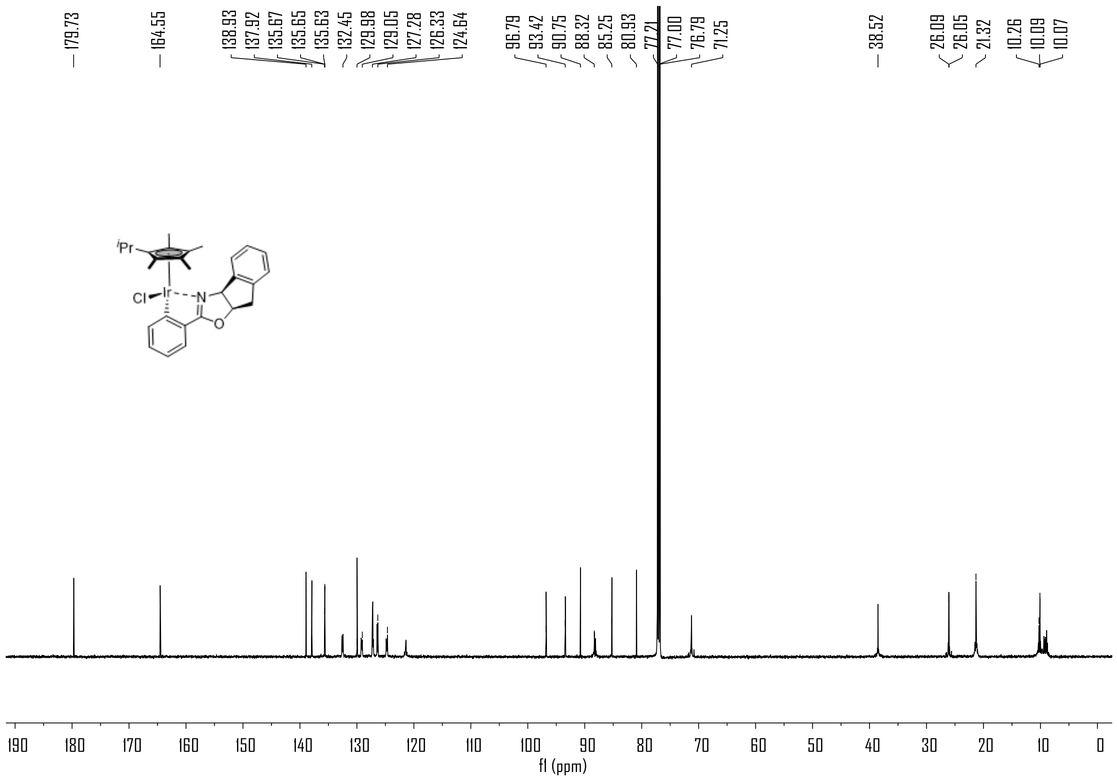

^{1}H NMR spectra of **Ir5** (600 MHz, $CDCl_3$)

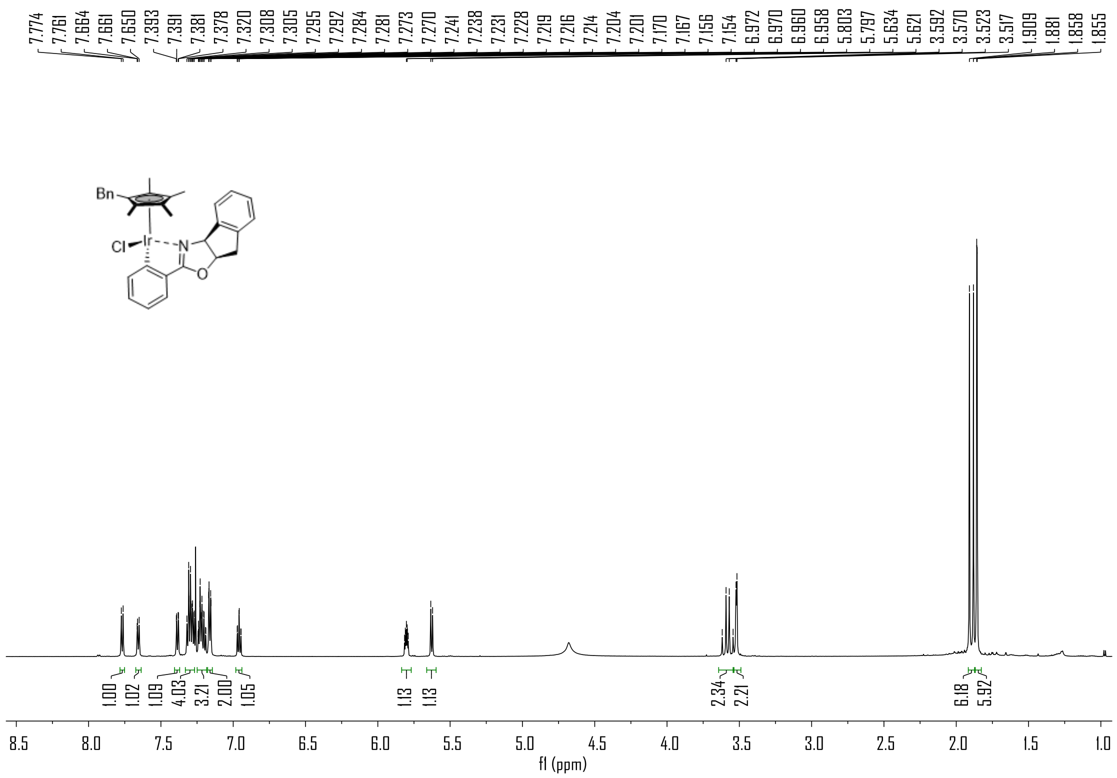

^{13}C NMR spectra of **Ir5** (151 MHz, $CDCl_3$)

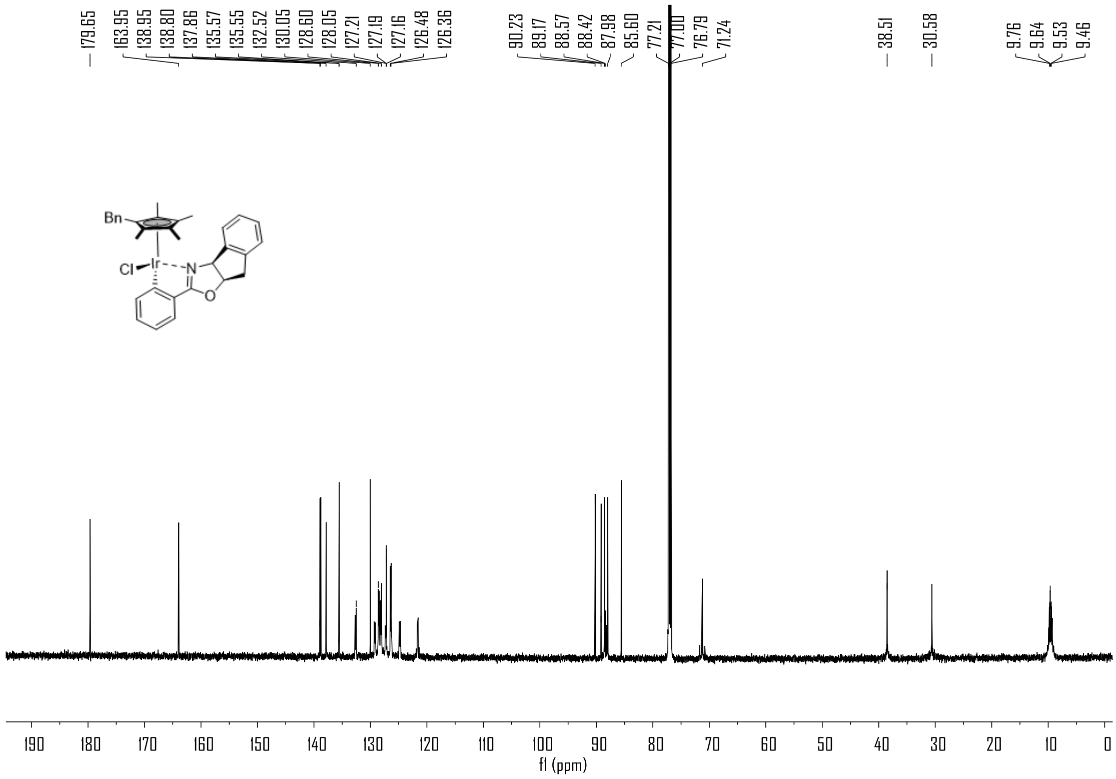

^{1}H NMR spectra of **Ir7** (600 MHz, $CDCl_3$)

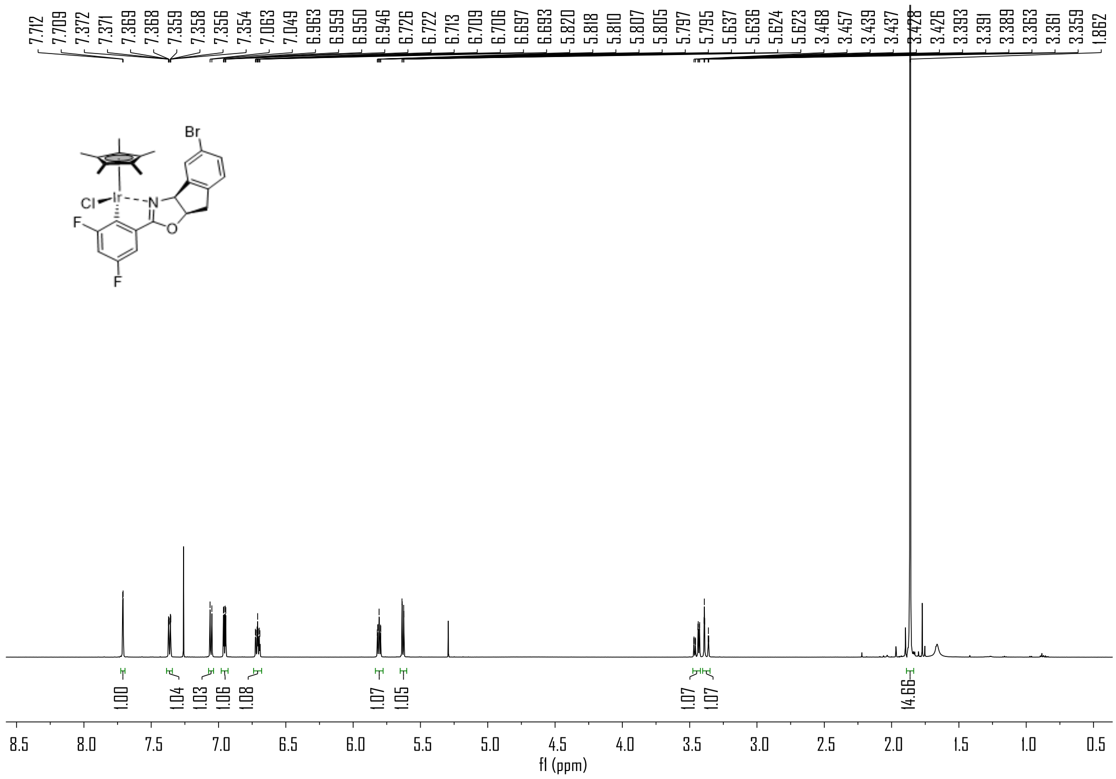

^{13}C NMR spectra of **Ir7** (151 MHz, $CDCl_3$)

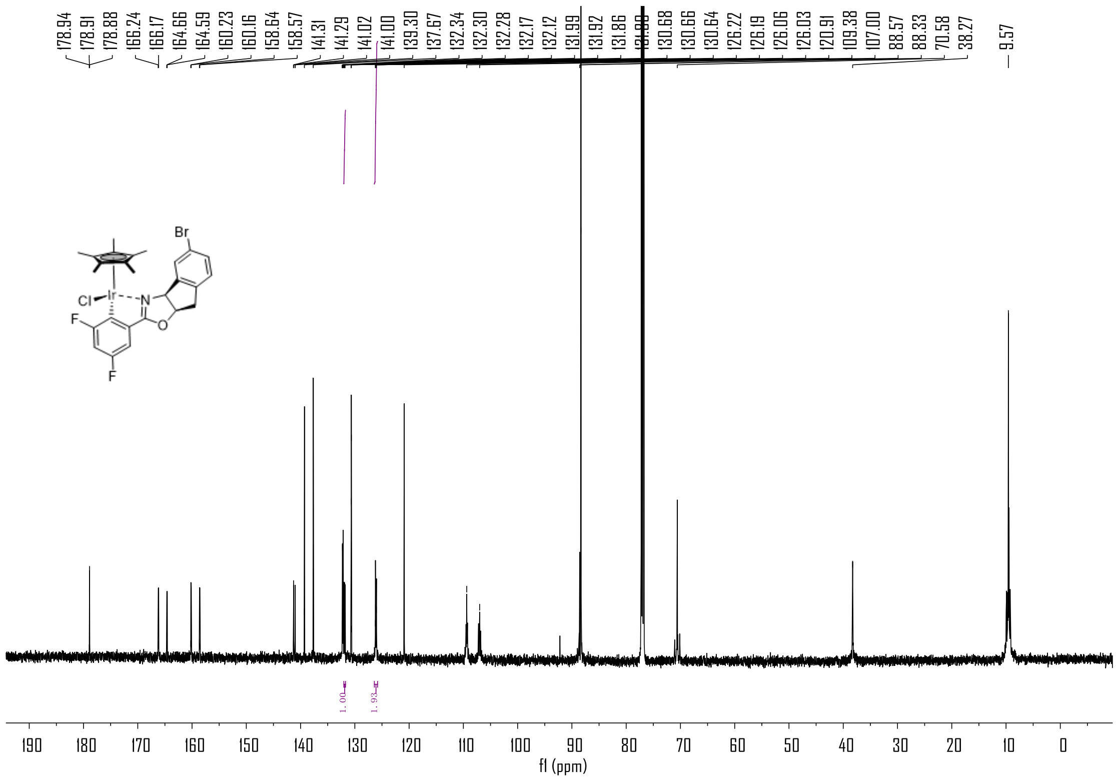

^{19}F NMR spectra of **Ir7** (565 MHz, $CDCl_3$)

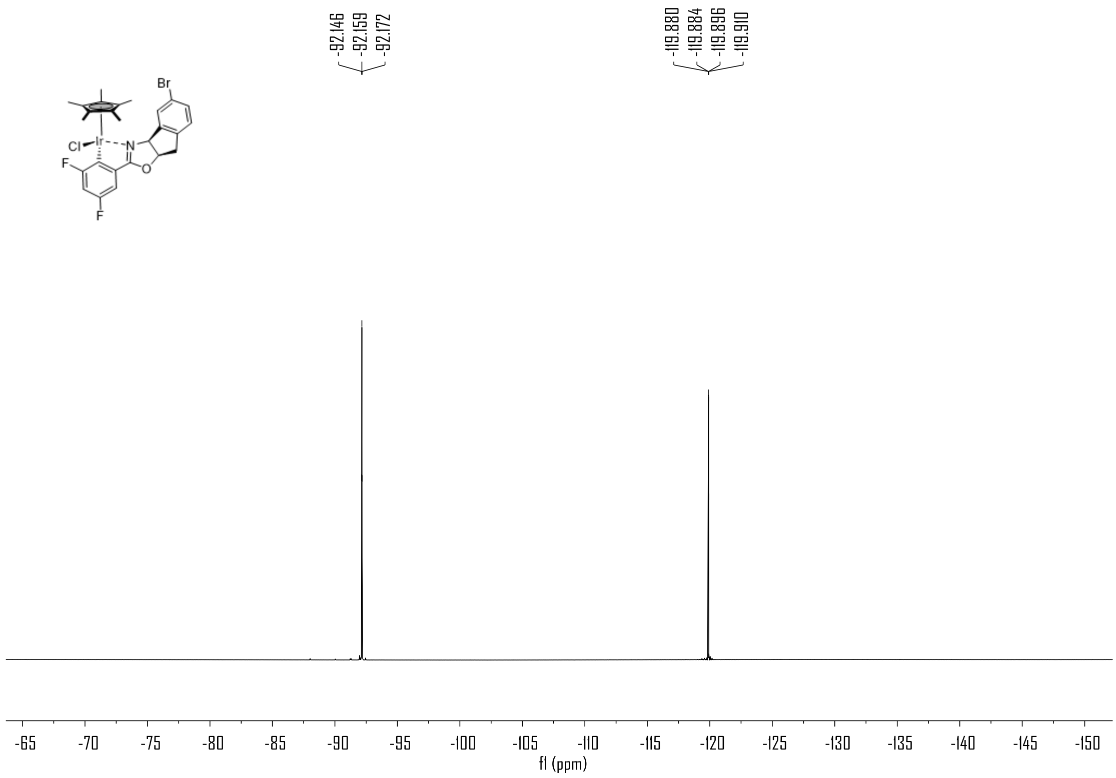

^{1}H NMR spectra of **Ir8** (600 MHz, $CDCl_3$)

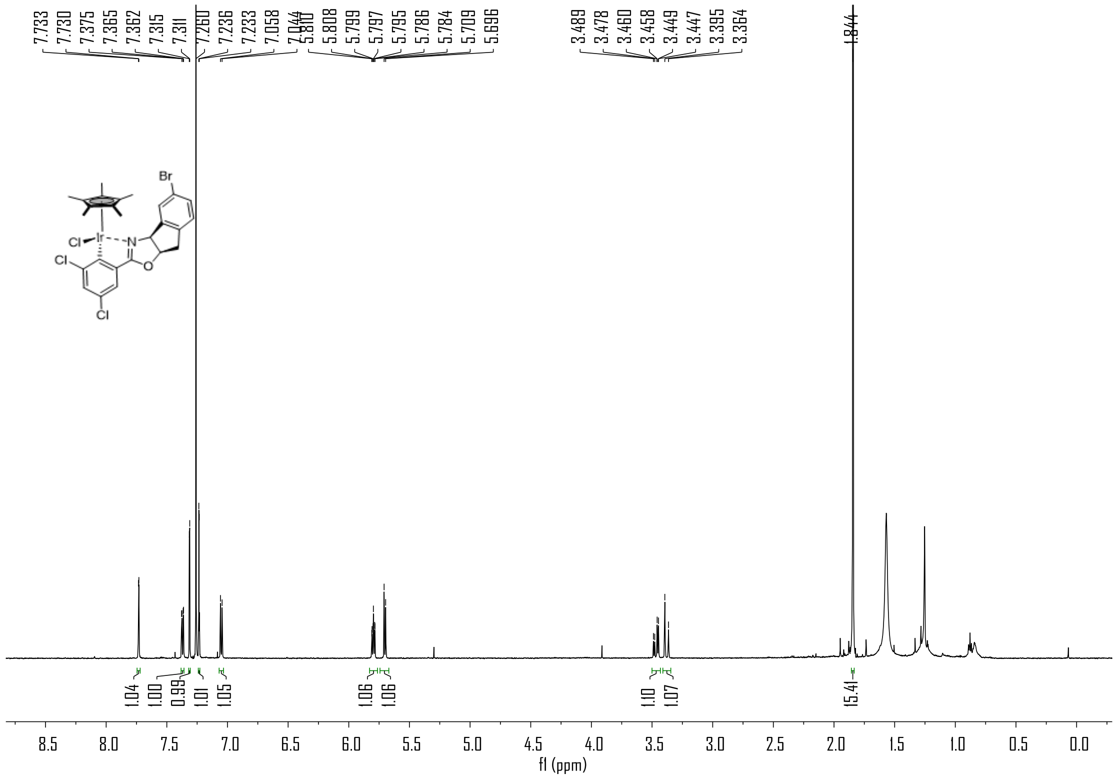

^{13}C NMR spectra of **Ir8** (151 MHz, $CDCl_3$)

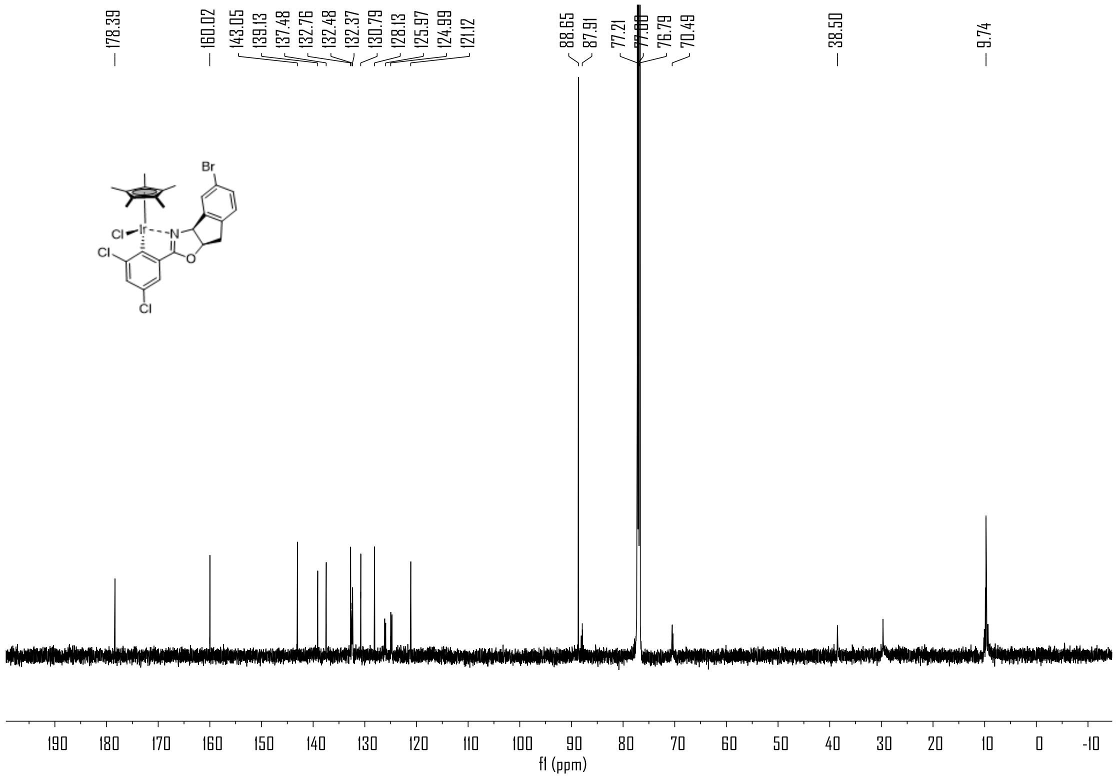

^{1}H NMR spectra of **Ir9** (600 MHz, $CDCl_3$)

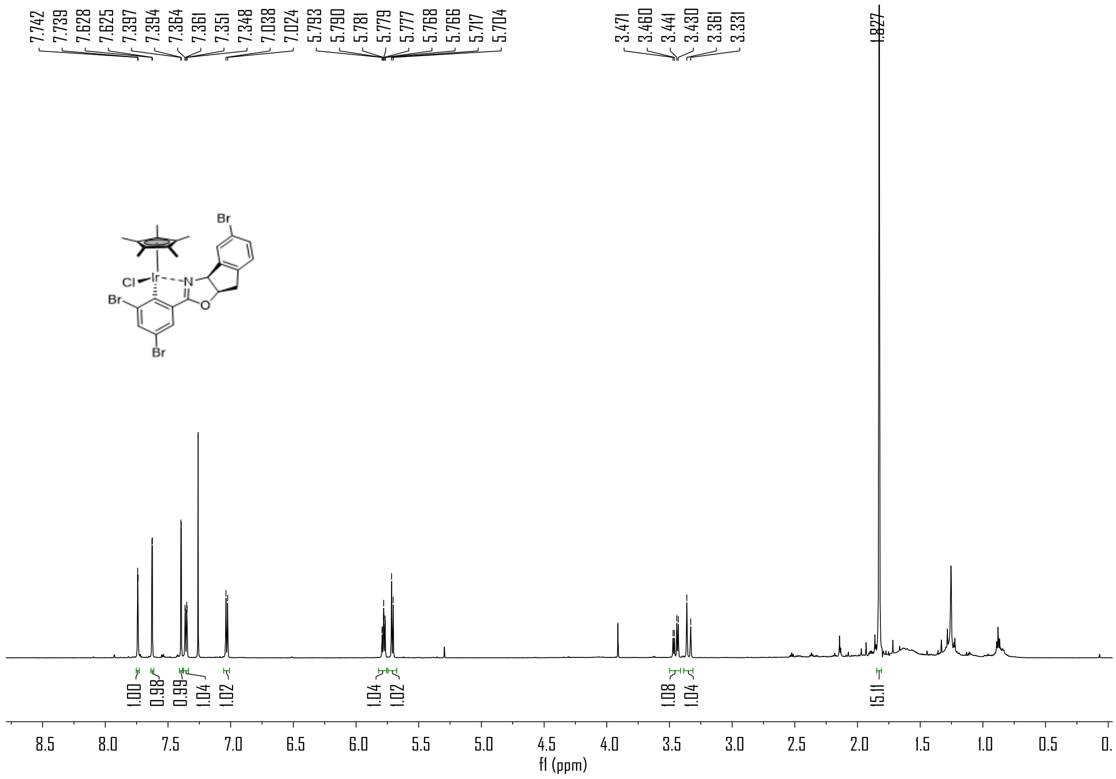

^{13}C NMR spectra of **Ir9** (151 MHz, $CDCl_3$)

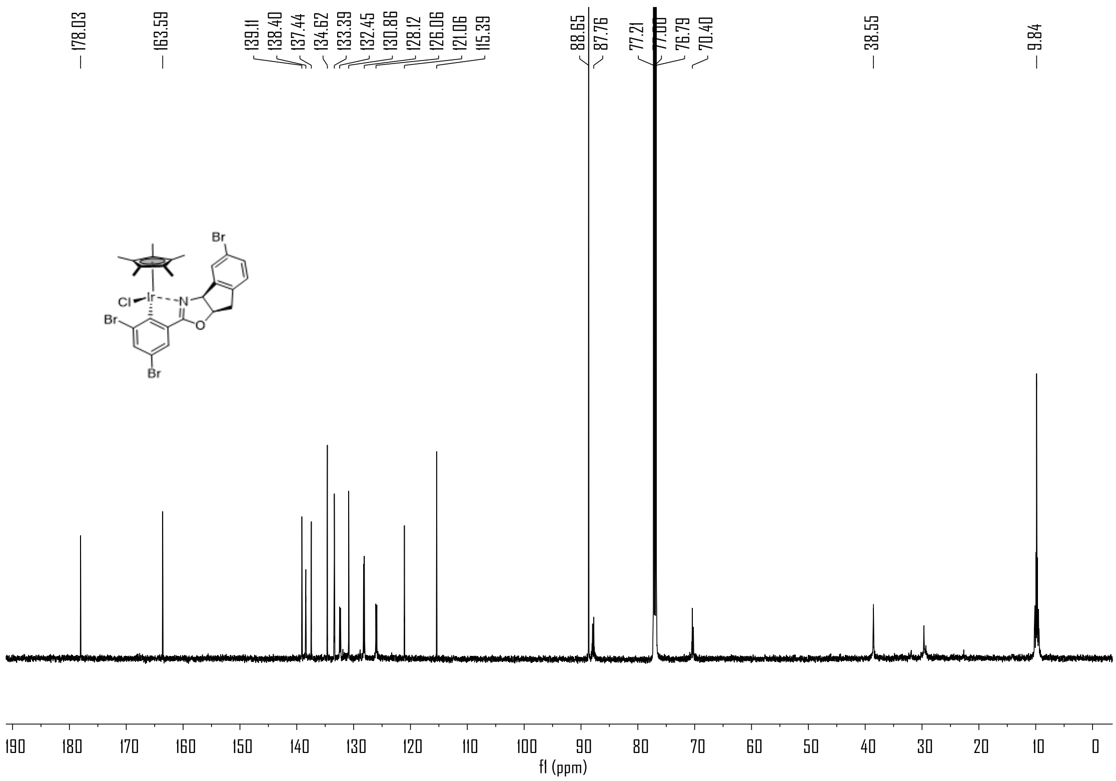

^{1}H NMR spectra of **Ir10** (600 MHz, $CDCl_3$)

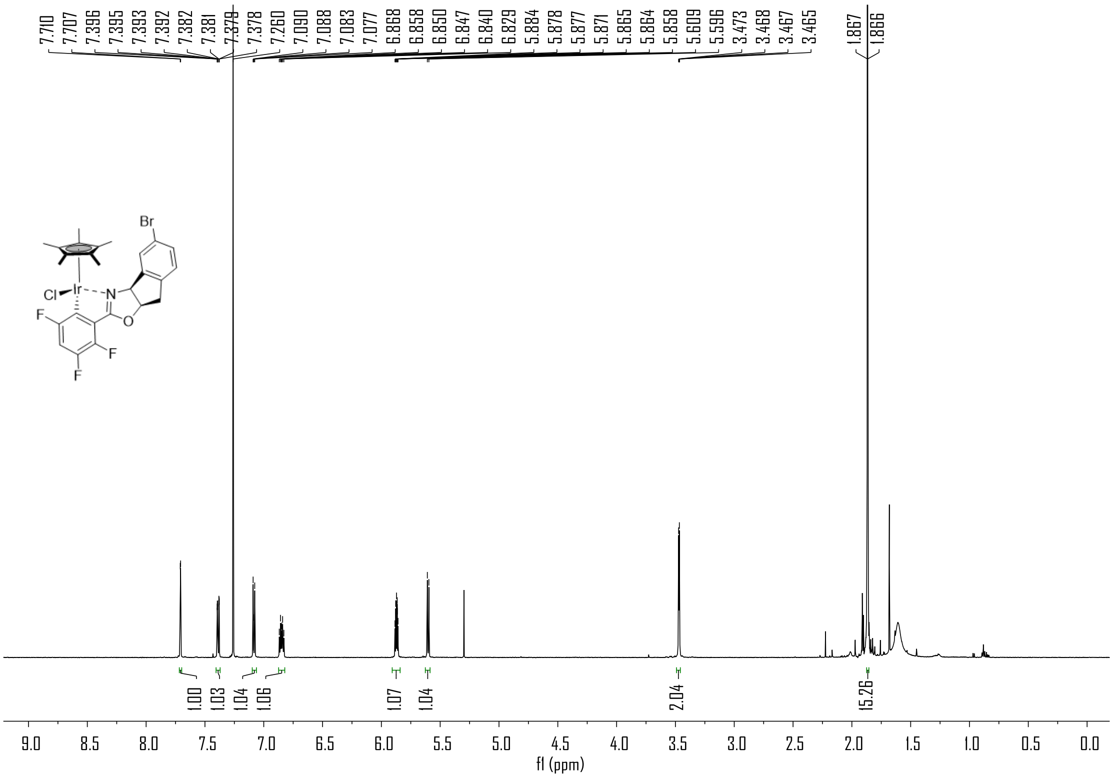

^{13}C NMR spectra of **Ir10** (151 MHz, $CDCl_3$)

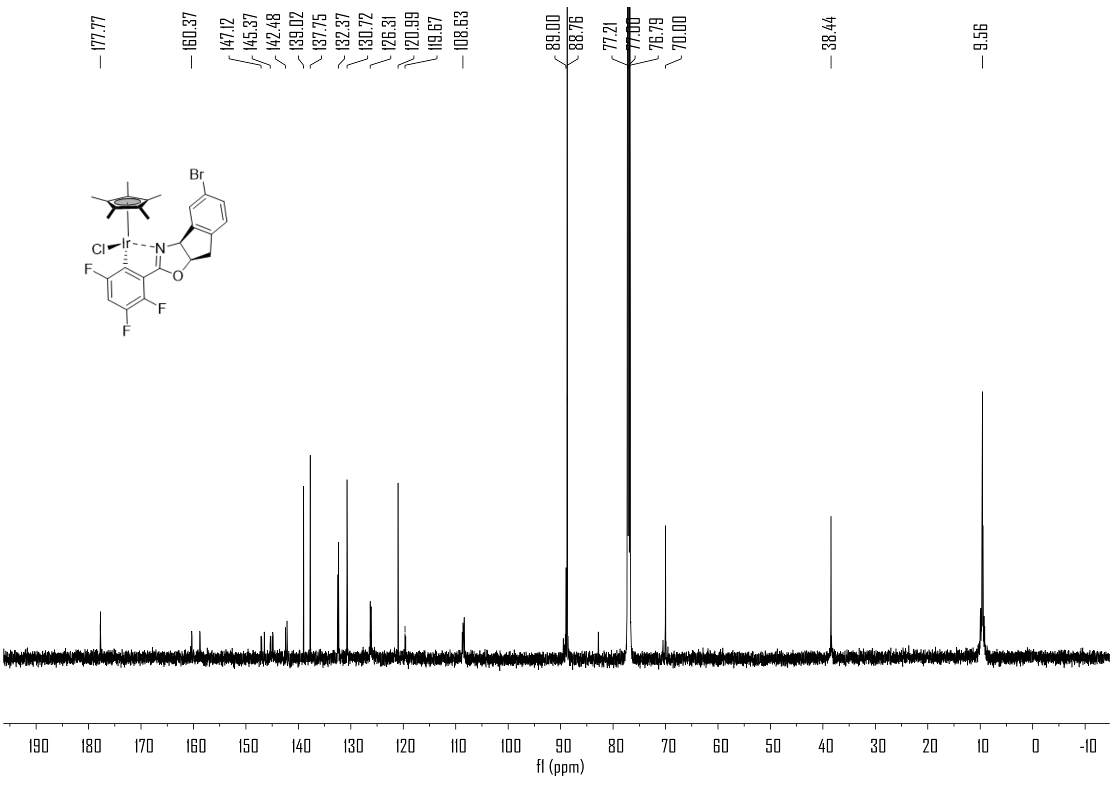

^{19}F NMR spectra of **Ir10** (565 MHz, $CDCl_3$)

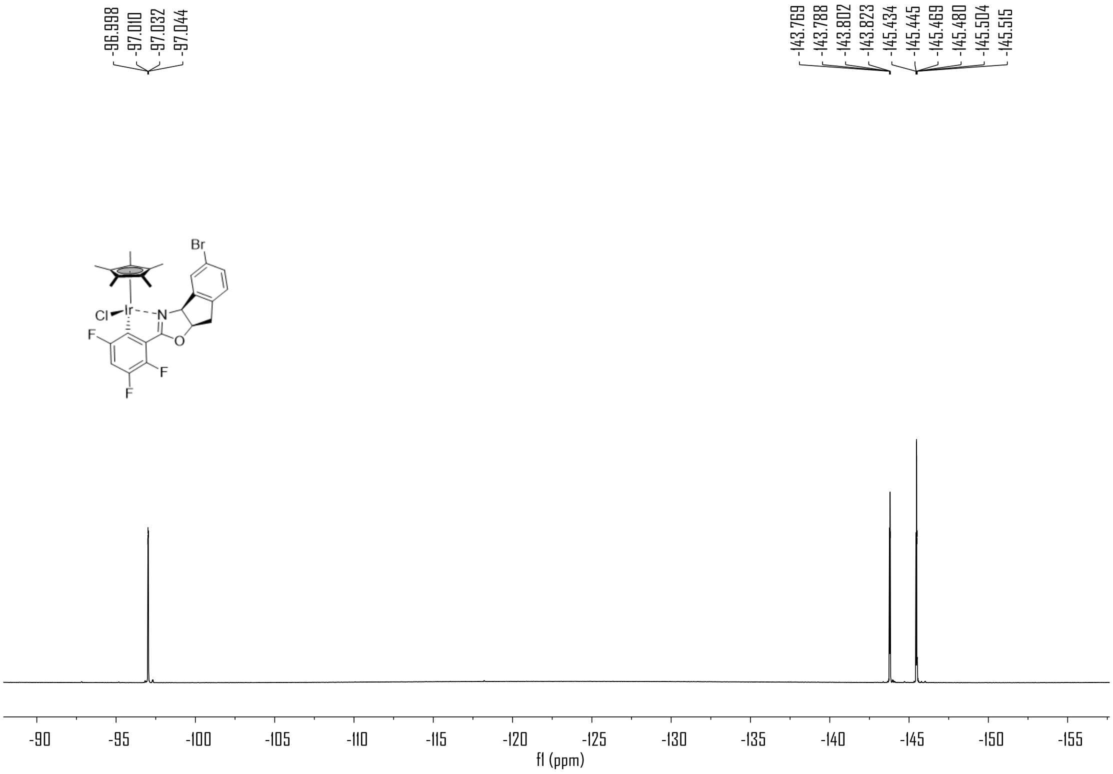

^{1}H NMR spectra of **Ir11** (600 MHz, $CDCl_3$)

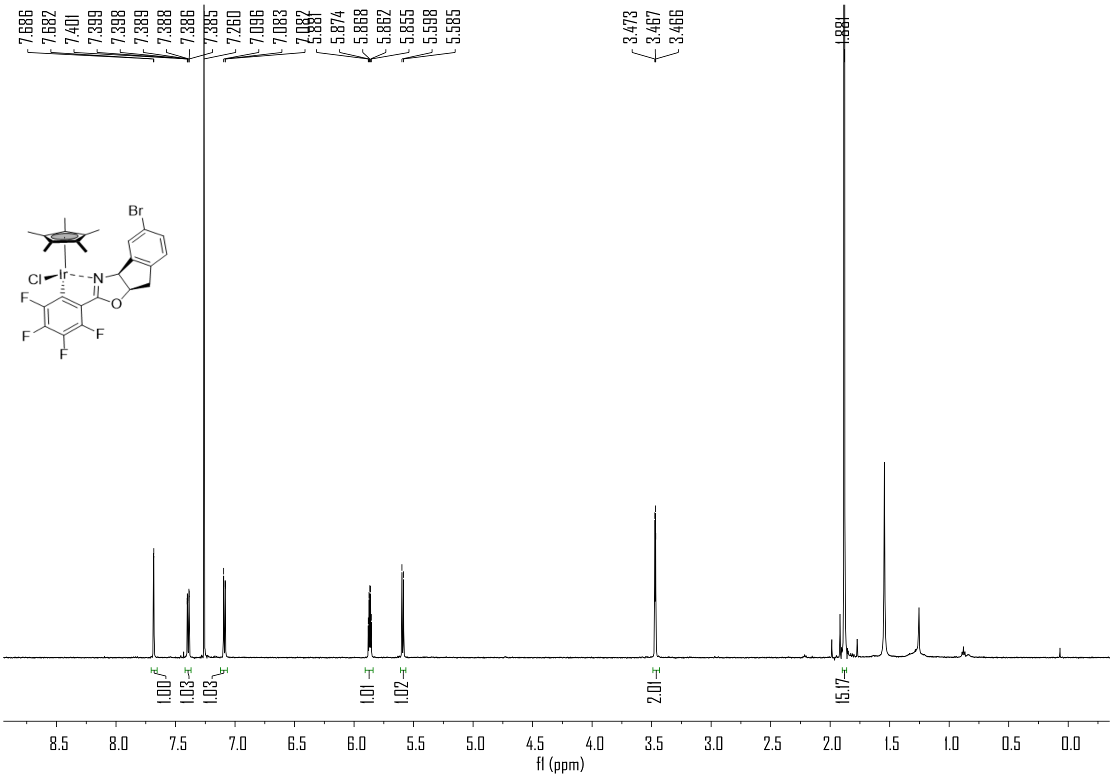

^{13}C NMR spectra of **Ir11** (151 MHz, $CDCl_3$)

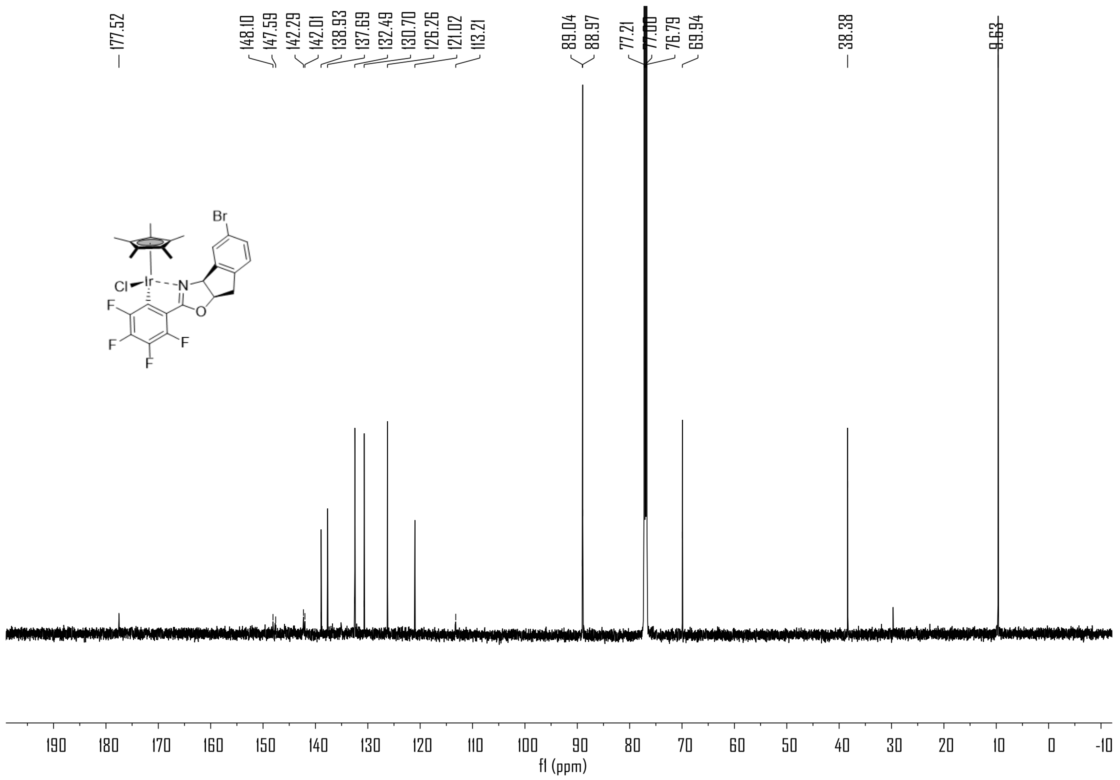

^{19}F NMR spectra of **Ir11** (565 MHz, $CDCl_3$)

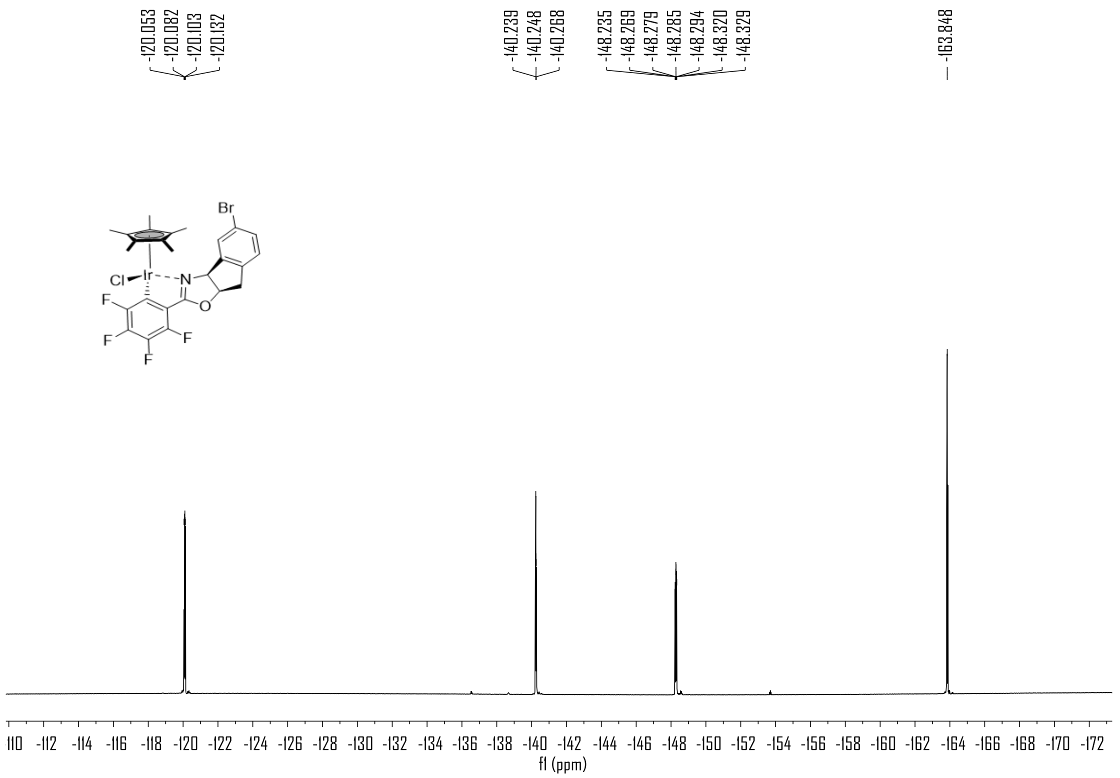

^{1}H NMR spectra of **S21** (400 MHz, $CDCl_3$)

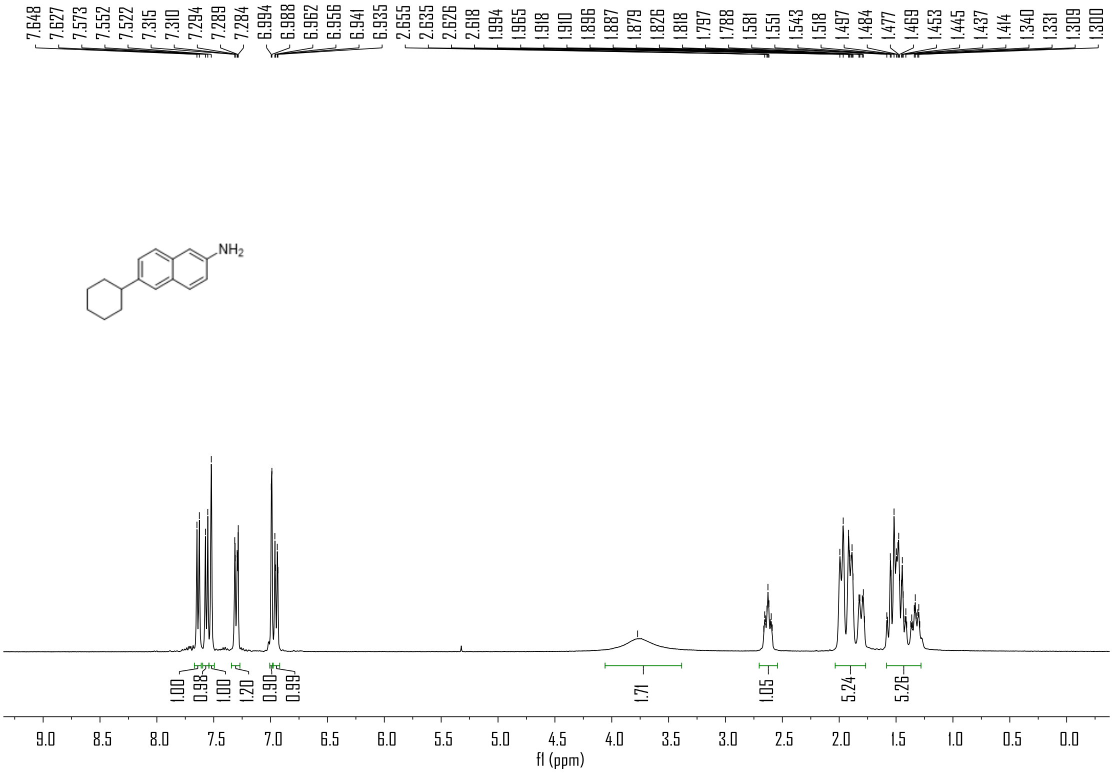

^{13}C NMR spectra of **S21** (101 MHz, $CDCl_3$)

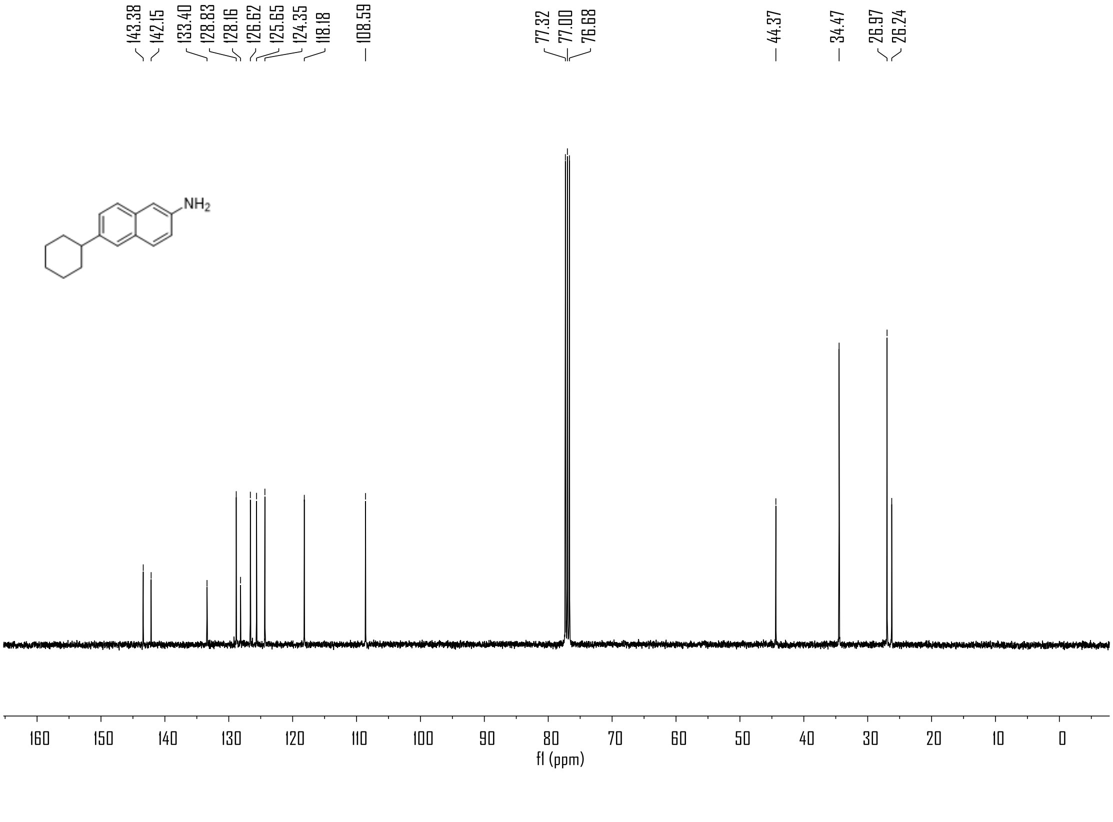

^{1}H NMR spectra of **S11** (400 MHz, $CDCl_3$)

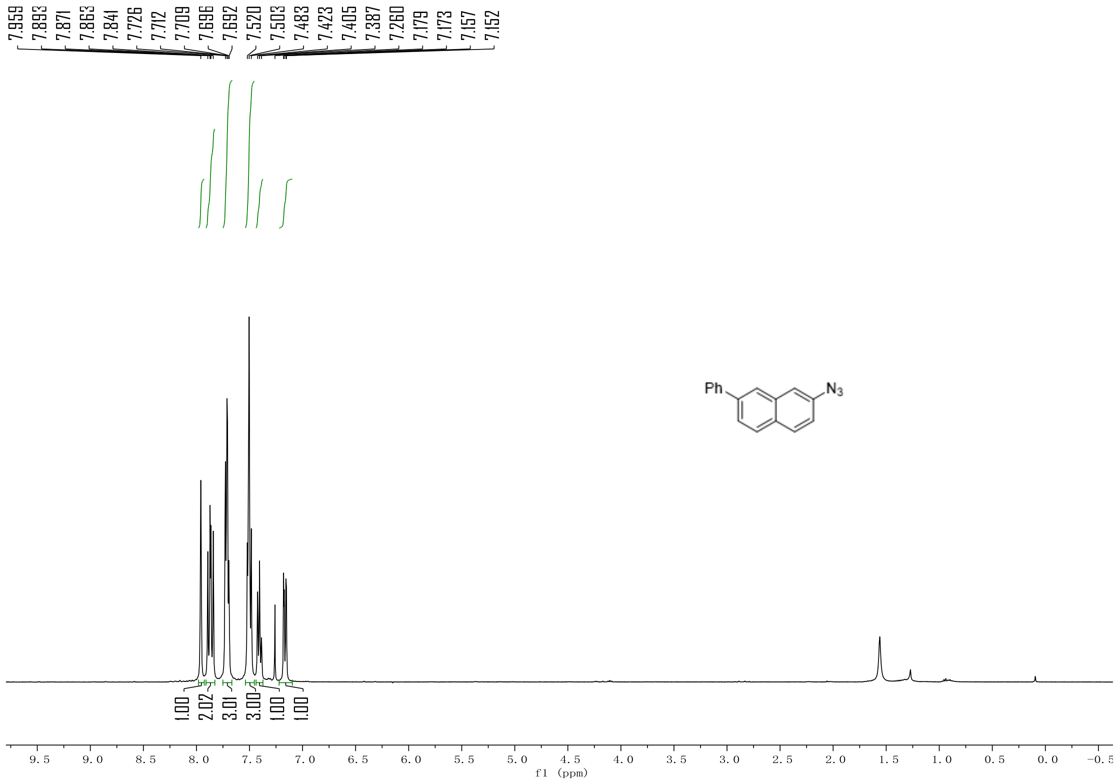

^{13}C NMR spectra of **S11** (101 MHz, $CDCl_3$)

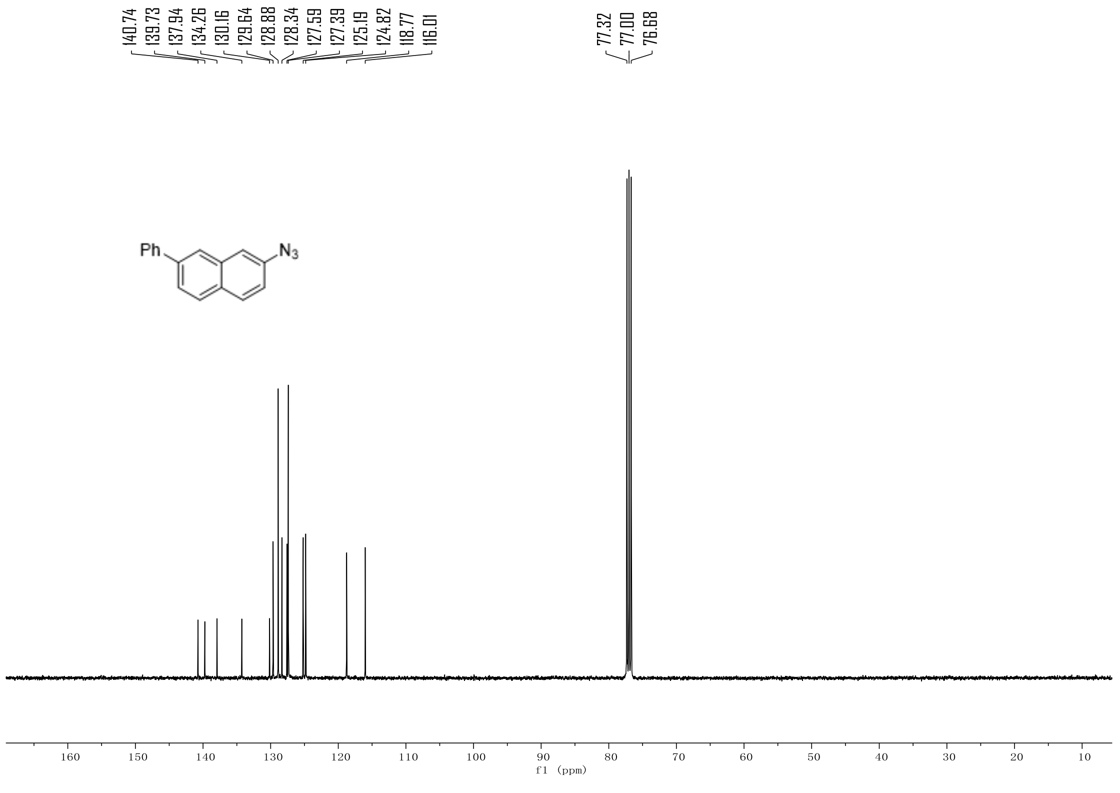

^{1}H NMR spectra of **S12** (600 MHz, $CDCl_3$)

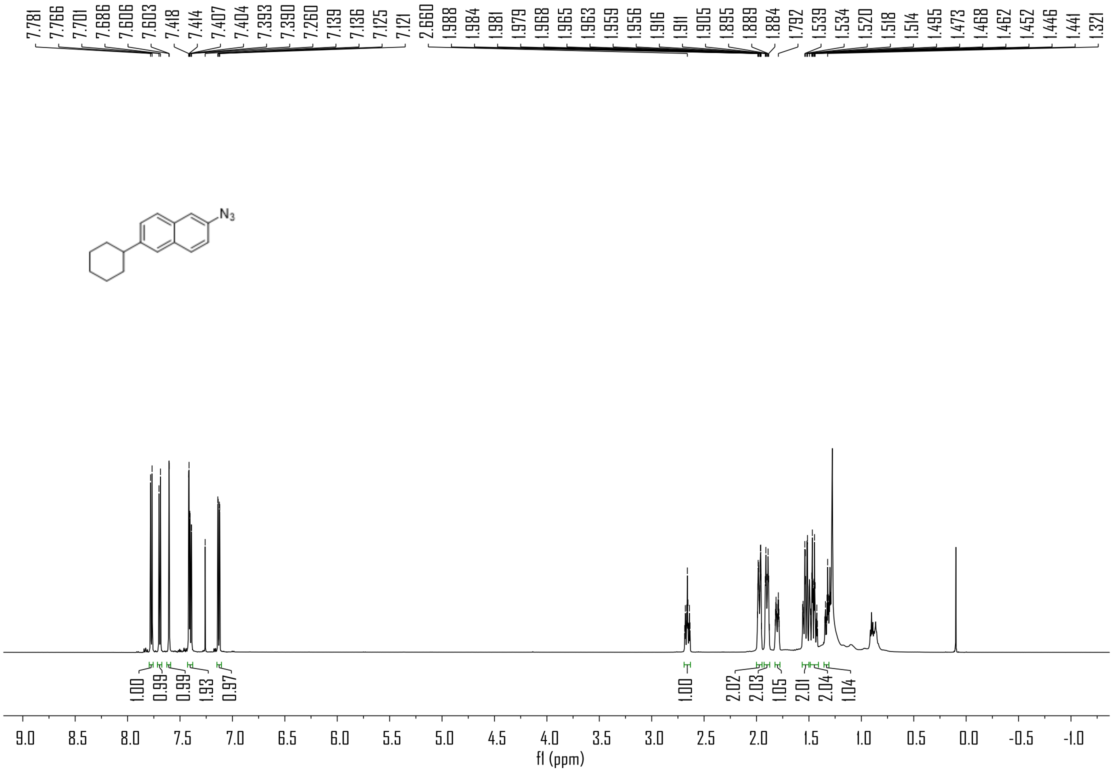

^{13}C NMR spectra of **S12** (151 MHz, $CDCl_3$)

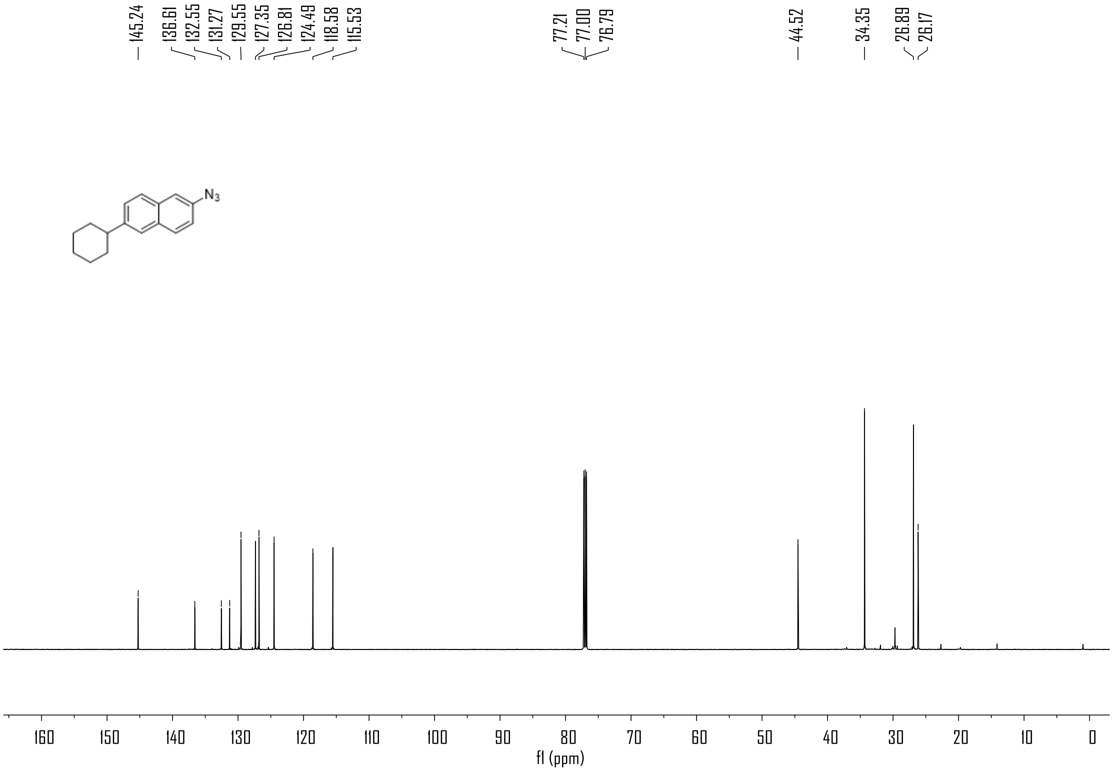

^{1}H NMR spectra of **S13** (400 MHz, $CDCl_3$)

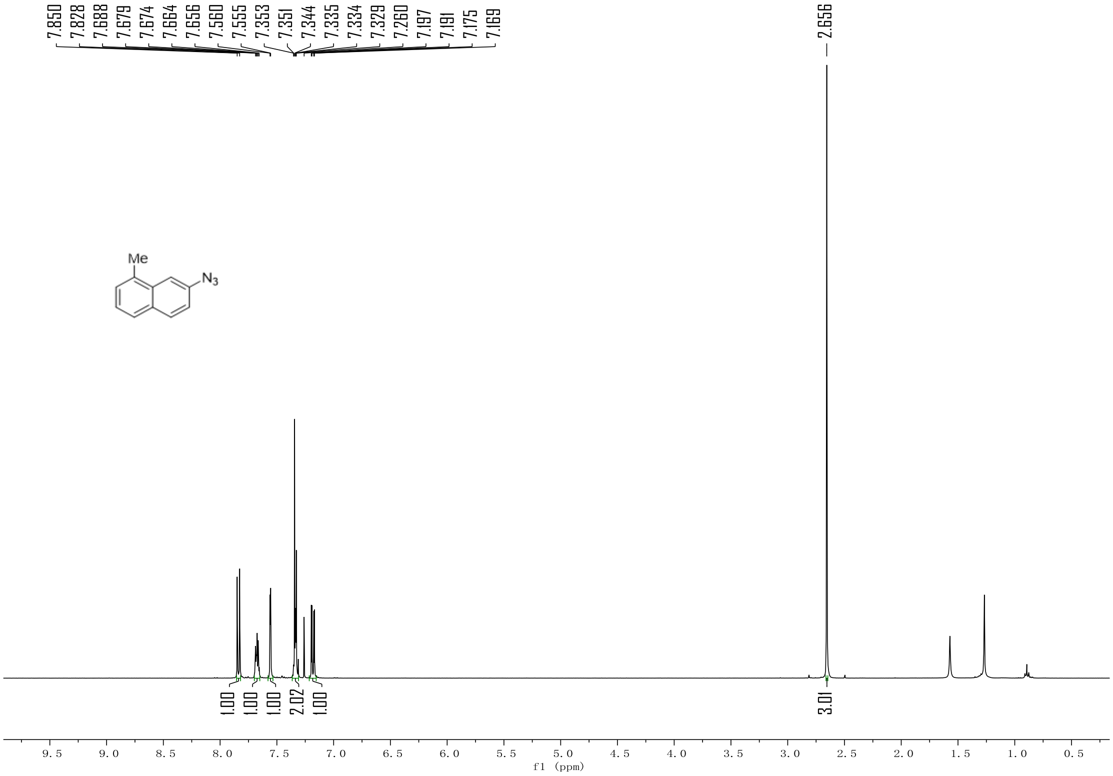

^{13}C NMR spectra of **S13** (101 MHz, $CDCl_3$)

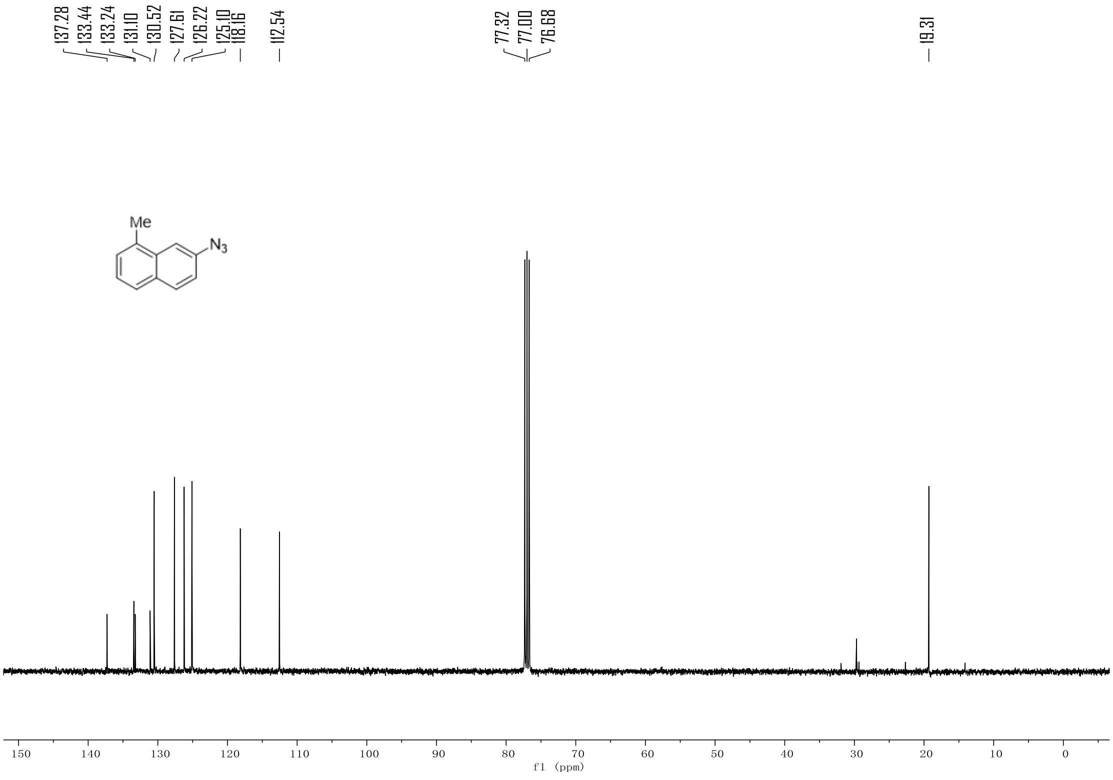

^{1}H NMR spectra of **3** (400 MHz, DMSO-d_6)

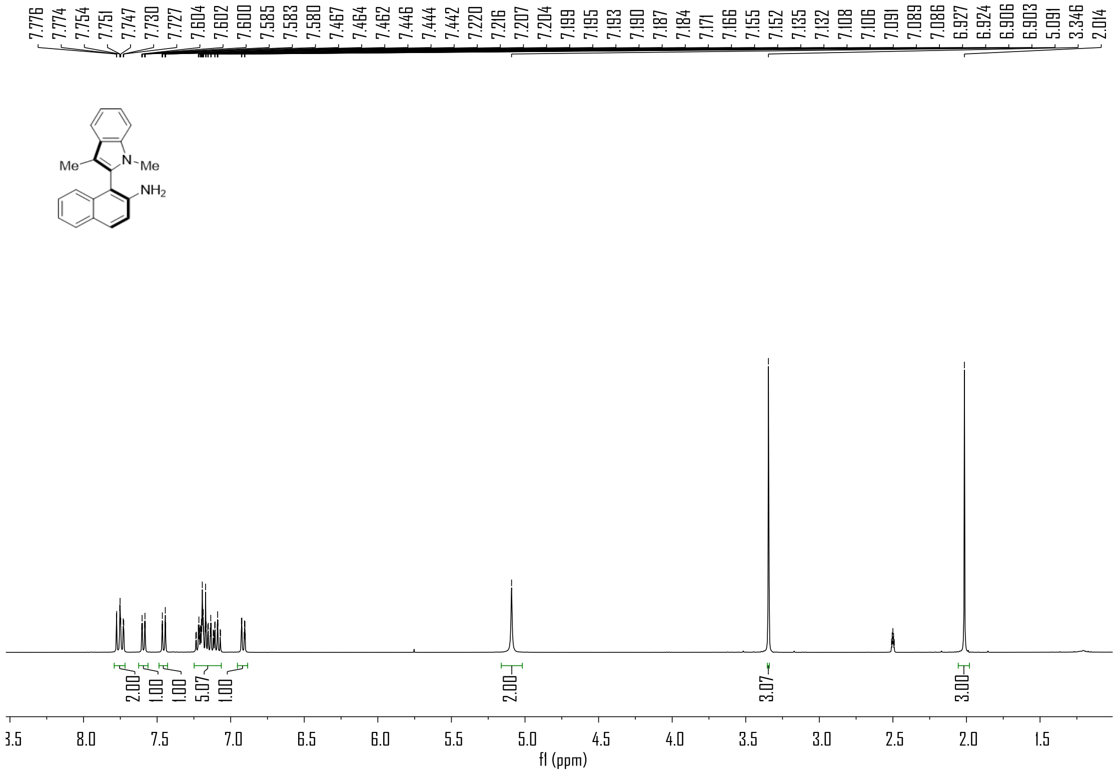

^{13}C NMR spectra of **3** (101 MHz, DMSO-d_6)

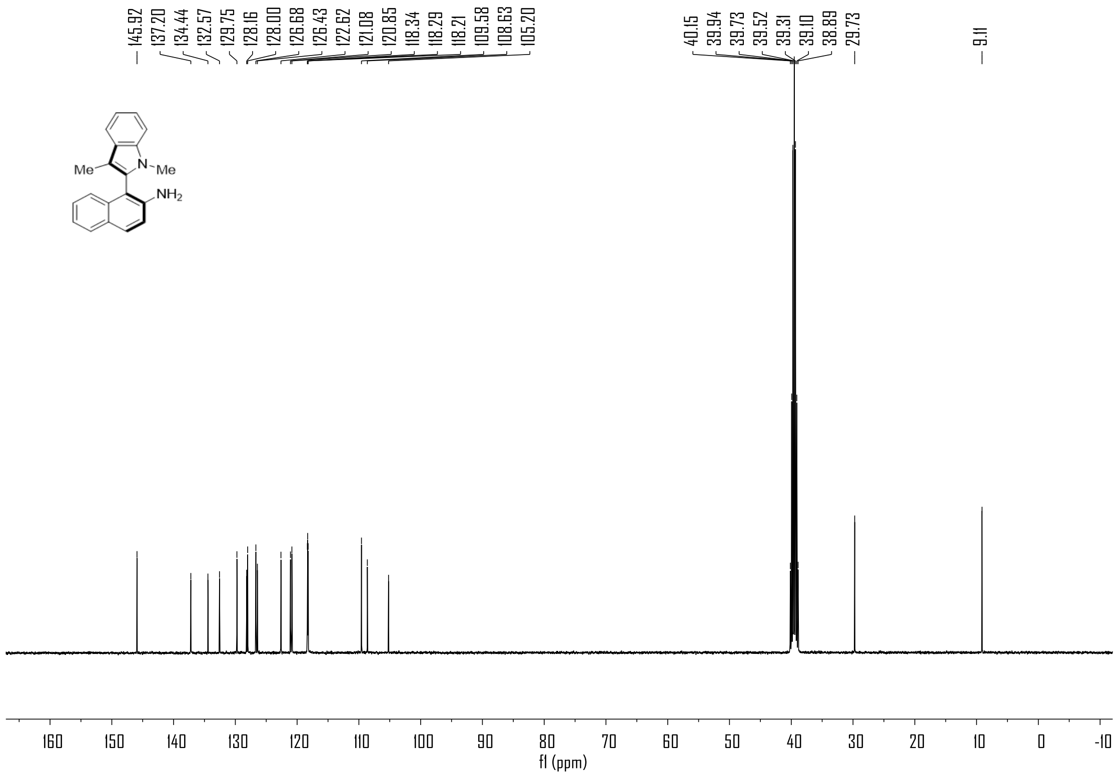

^{1}H NMR spectra of **4** (400 MHz, DMSO-d_6)

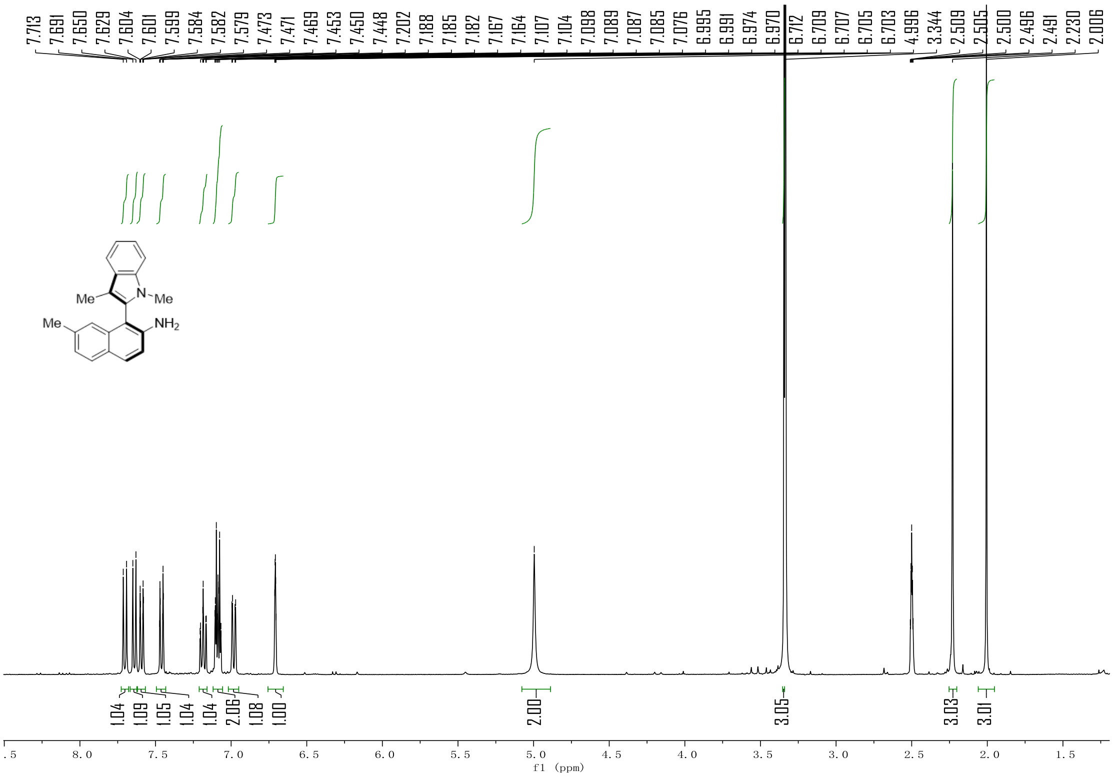

^{13}C NMR spectra of **4** (101 MHz, DMSO-d_6)

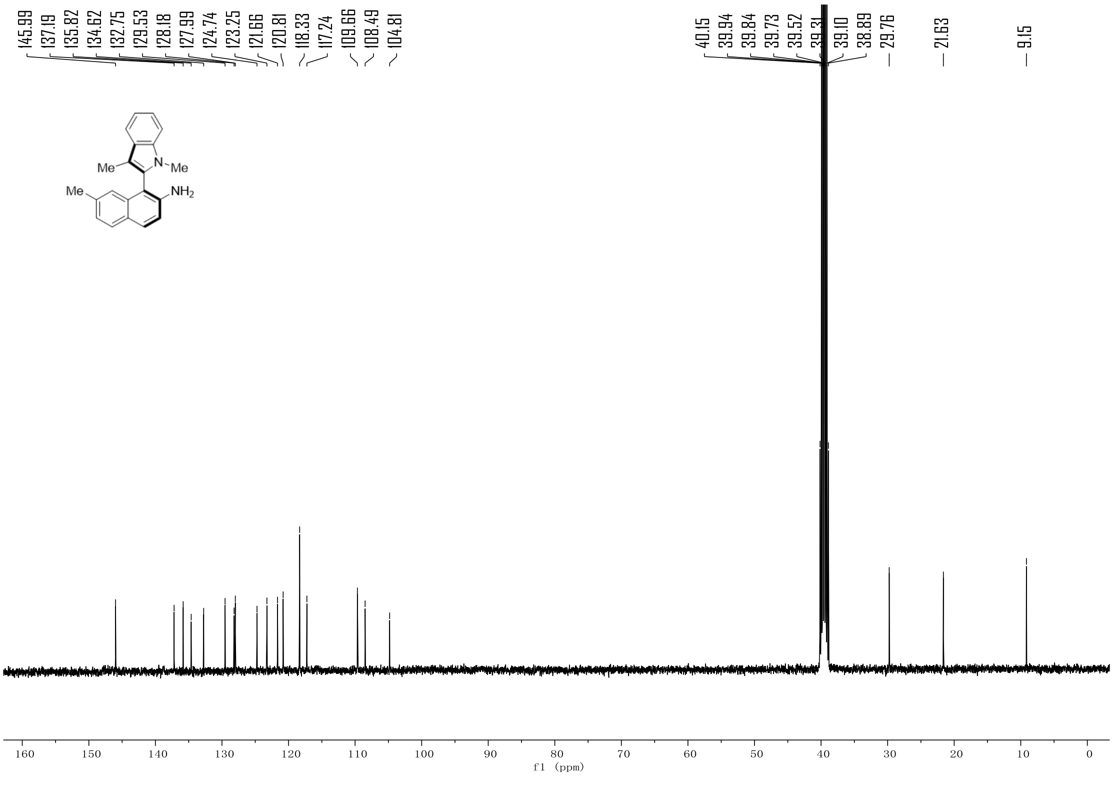

^{1}H NMR spectra of **5** (400 MHz, DMSO-d_6)

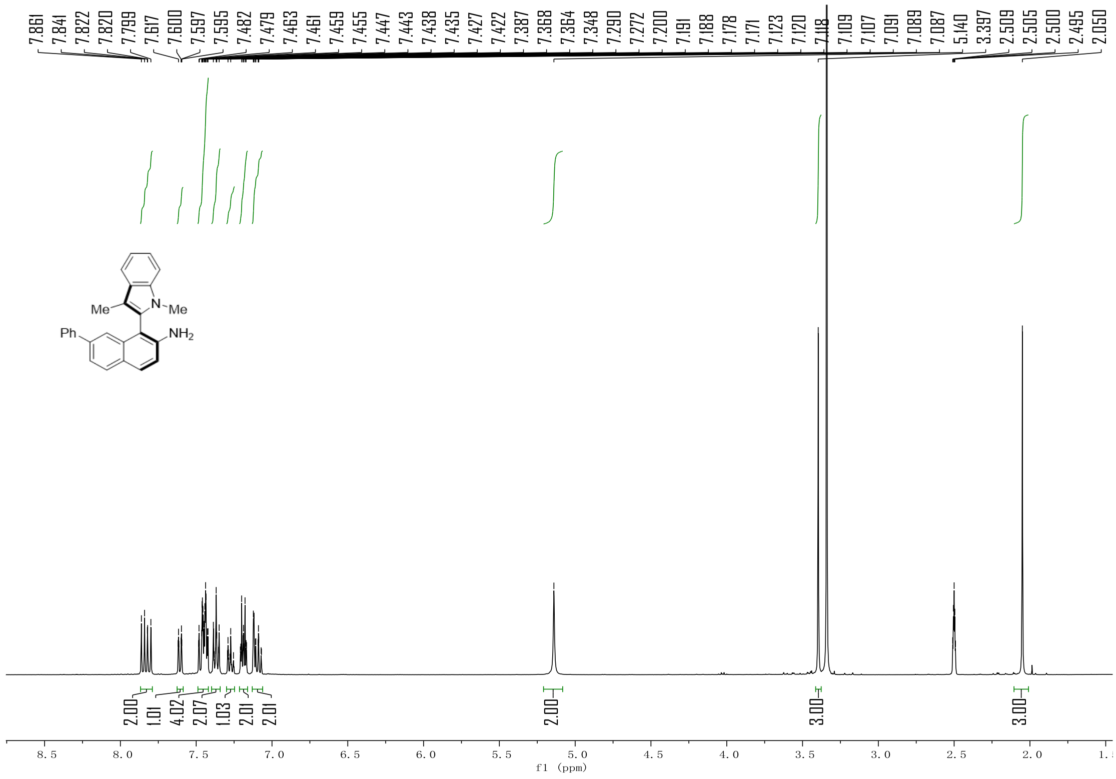

^{13}C NMR spectra of **5** (101 MHz, DMSO-d_6)

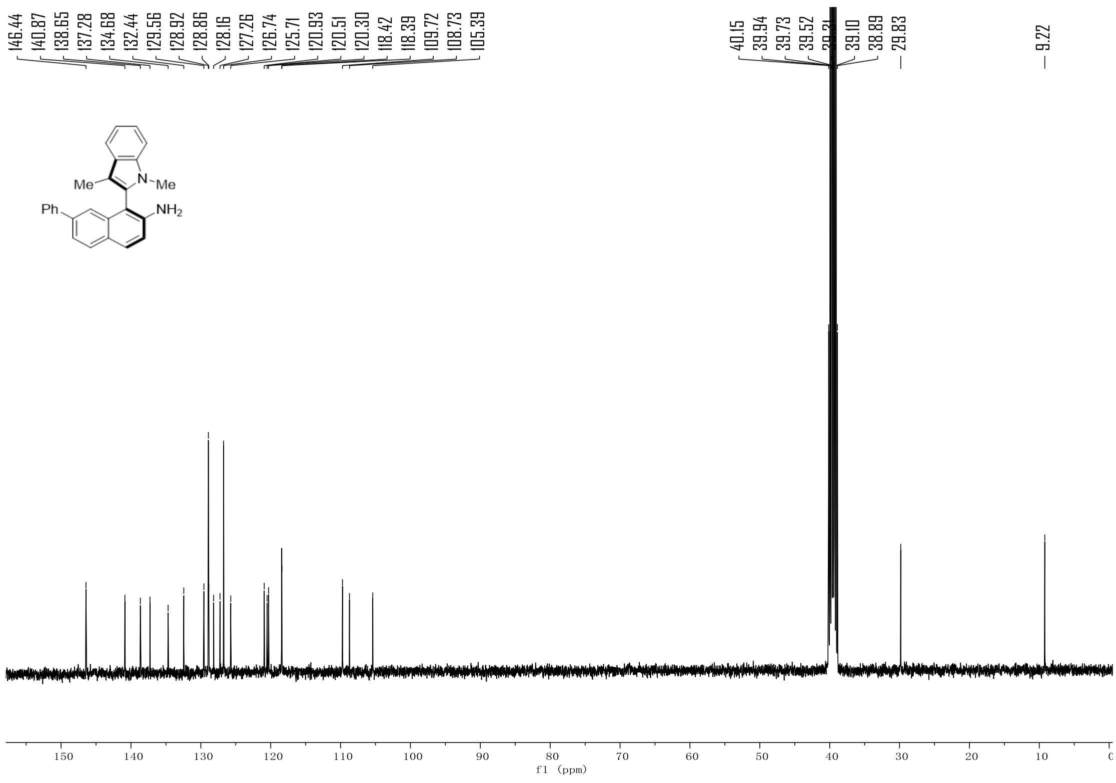

^{1}H NMR spectra of **6** (400 MHz, Methylene Chloride-d_2)

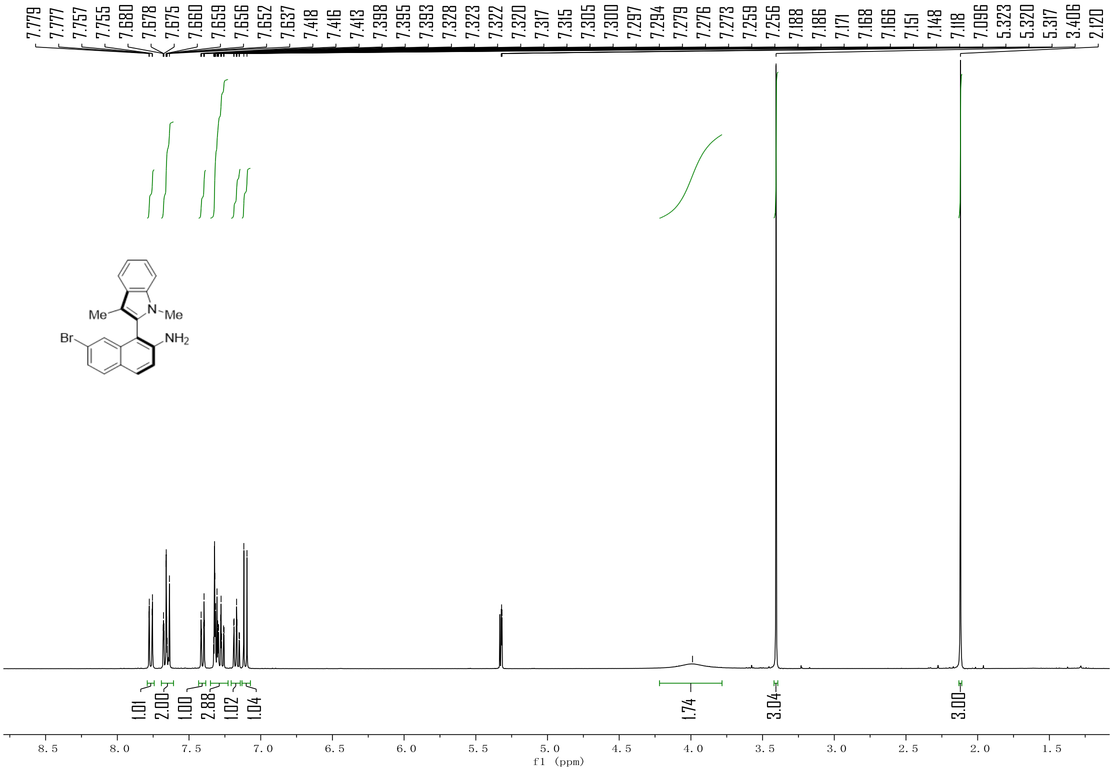

^{13}C NMR spectra of **6** (101 MHz, Methylene Chloride-d_2)

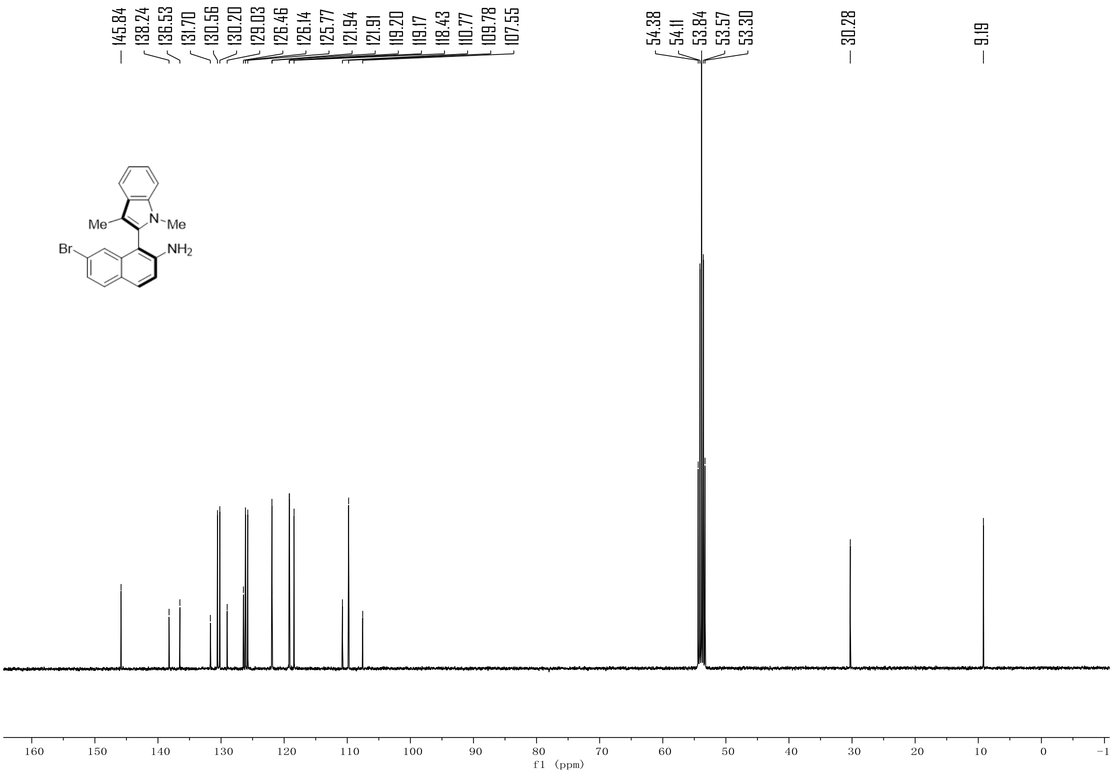

^{1}H NMR spectra of **7** (400 MHz, Methylene Chloride-d_2)

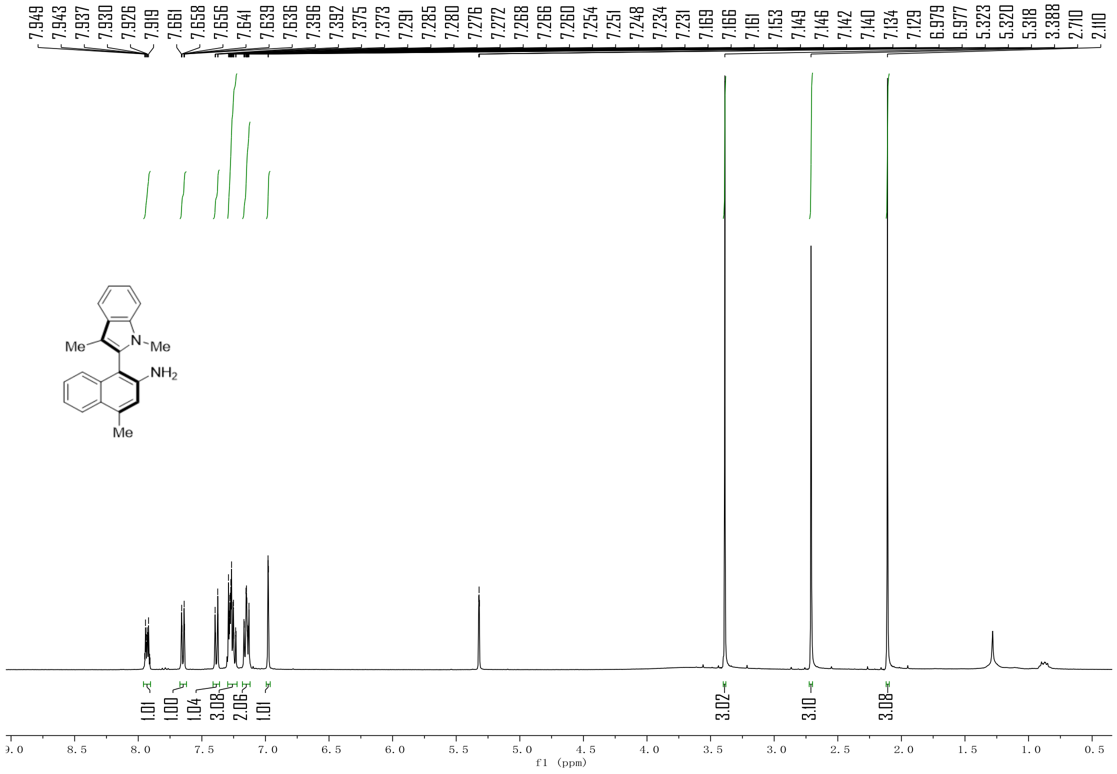

^{13}C NMR spectra of **7** (101 MHz, Methylene Chloride-d_2)

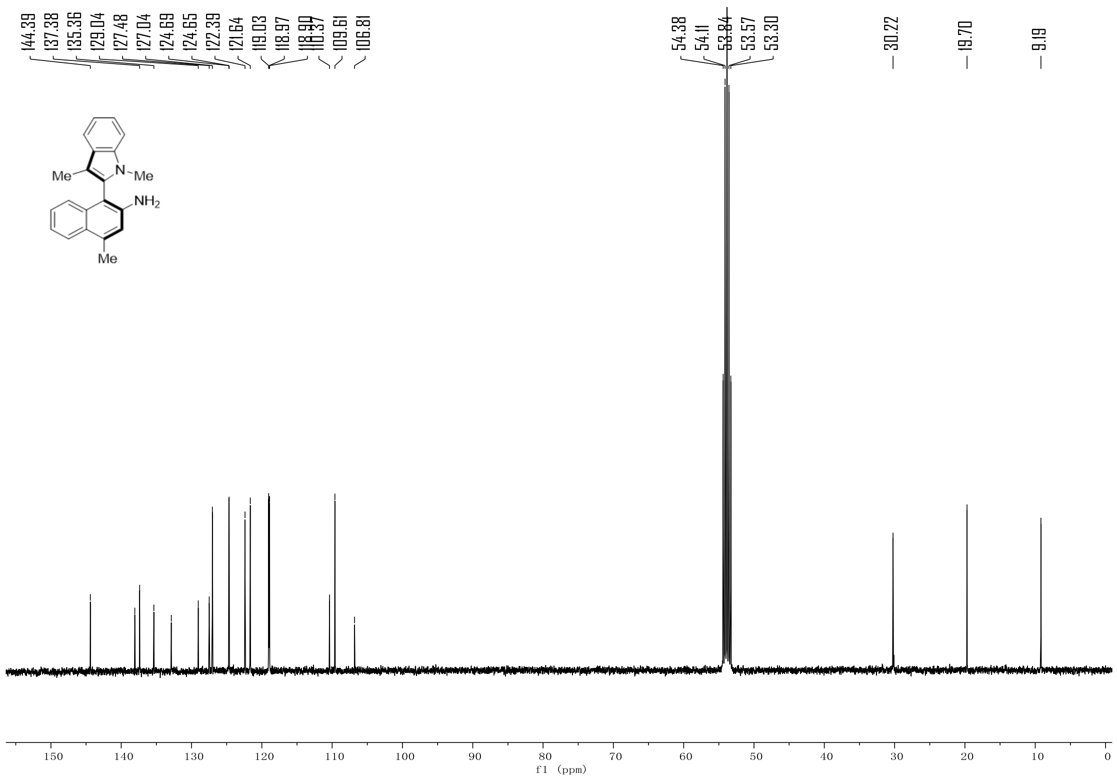

^{1}H NMR spectra of **8** (400 MHz, Methylene Chloride-d_2)

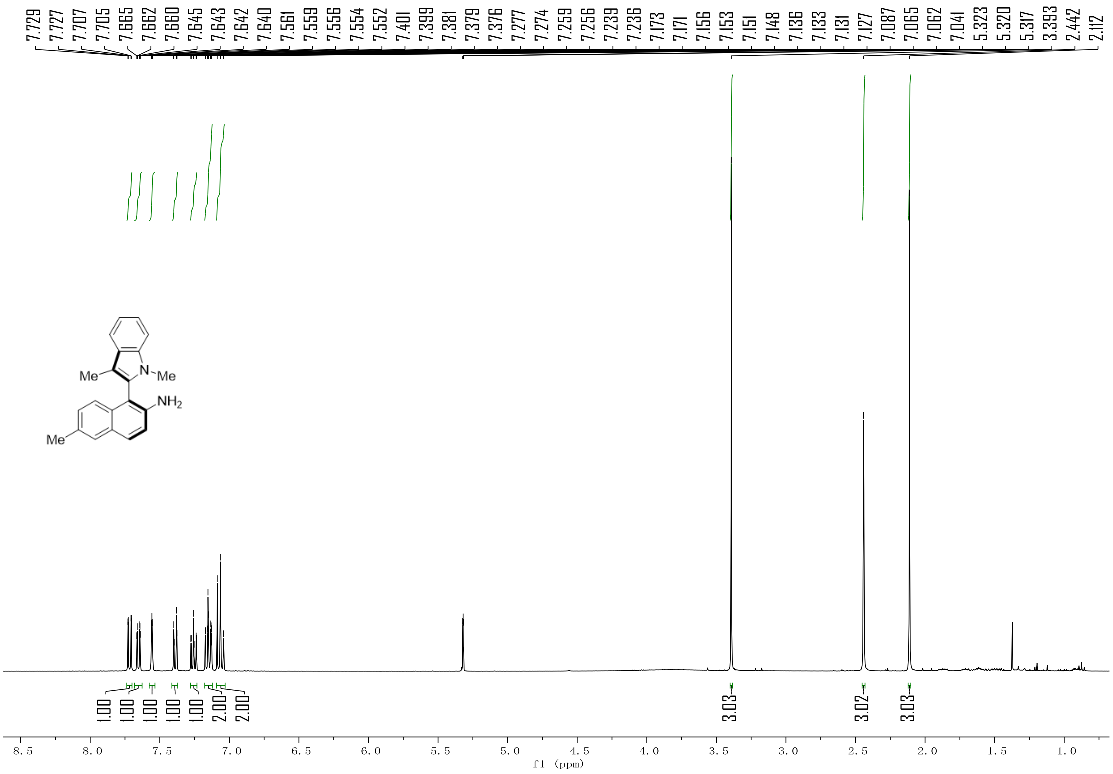

^{13}C NMR spectra of **8** (126 MHz, Methylene Chloride-d_2)

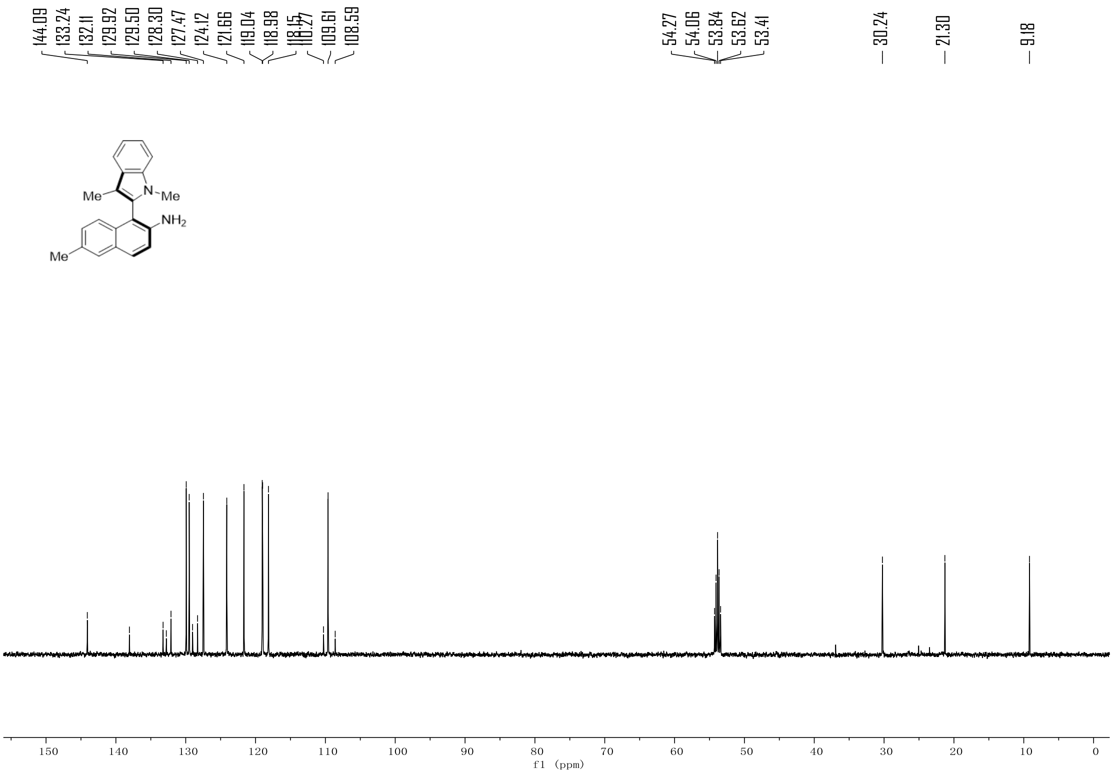

^1H NMR spectra of **9** (400 MHz, DMSO-d_6)

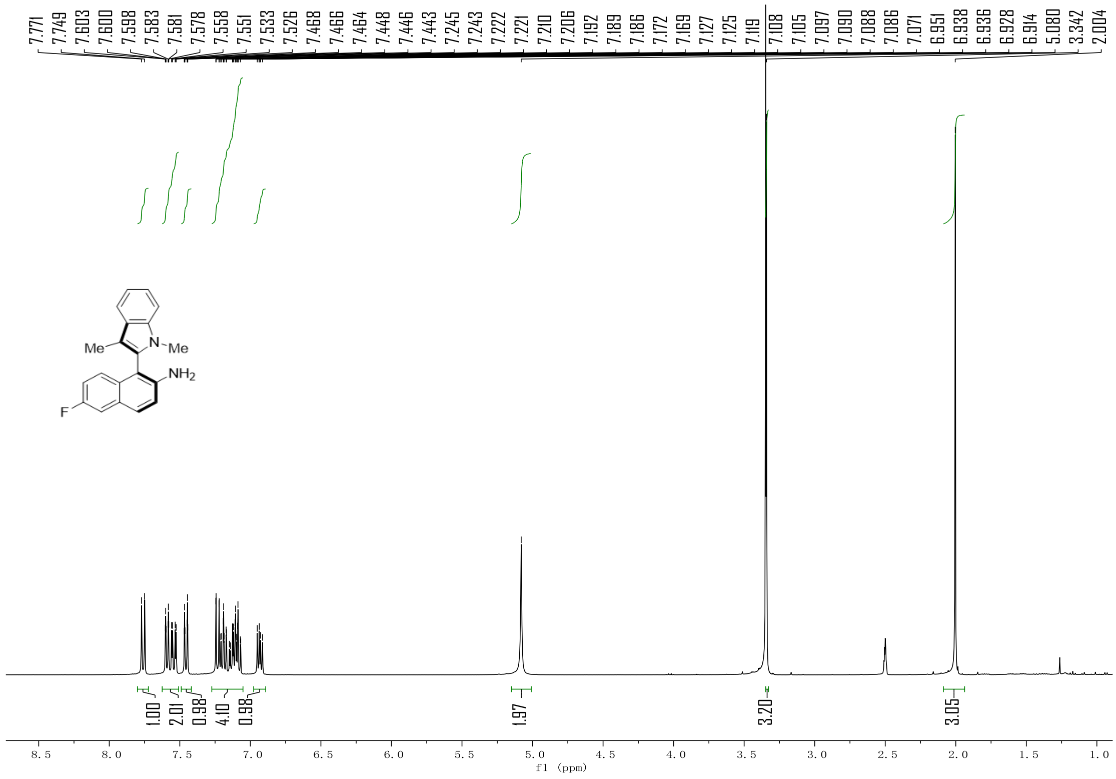

^{13}C NMR spectra of **9** (101 MHz, DMSO-d_6)

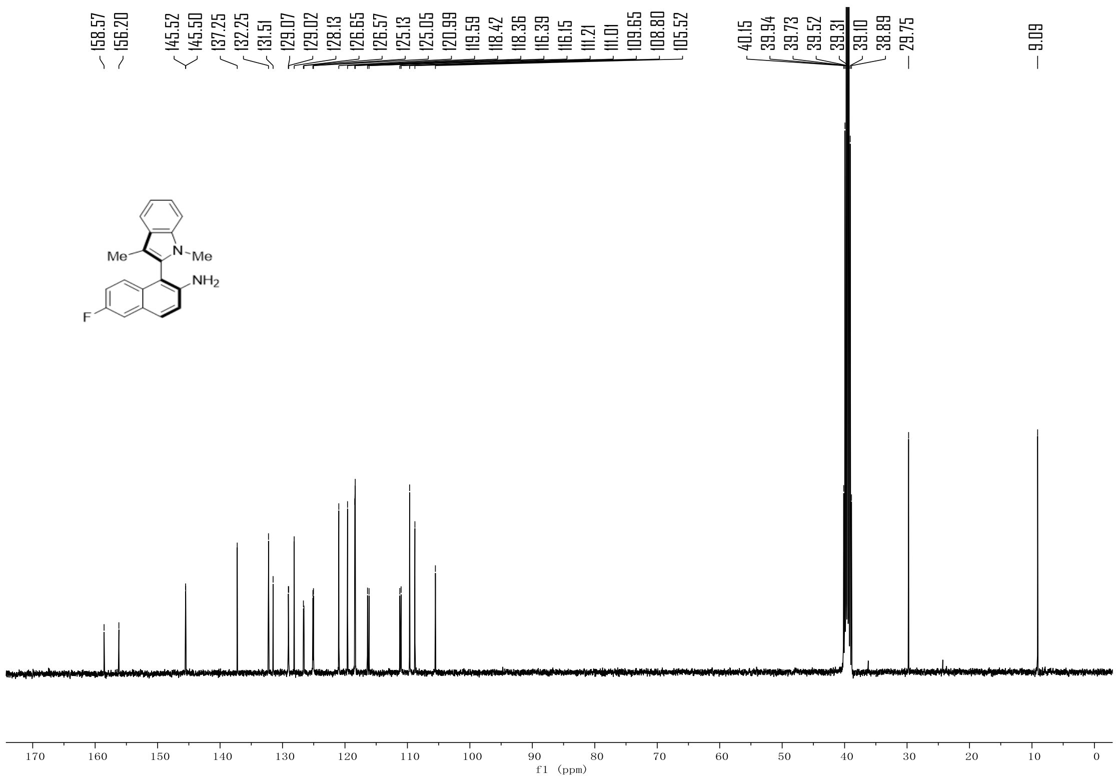

^{19}F NMR spectra of **9** (377 MHz, DMSO-d_6)

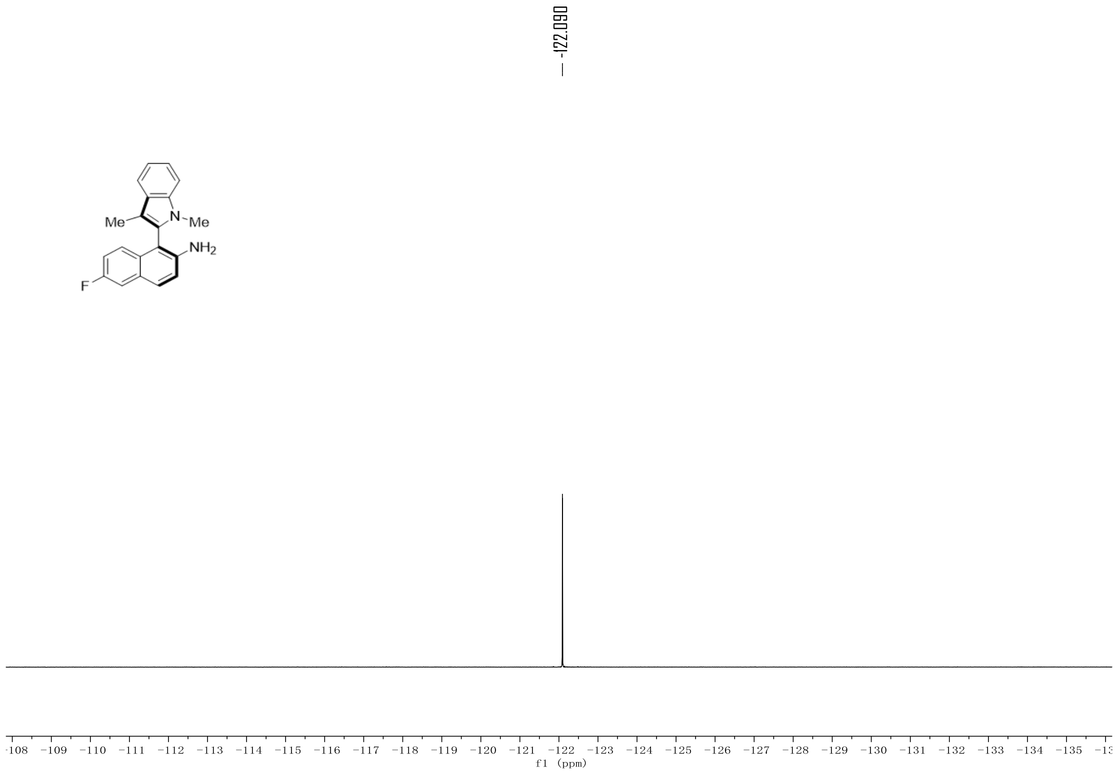

^{1}H NMR spectra of **10** (500 MHz, Methylene Chloride-d_2)

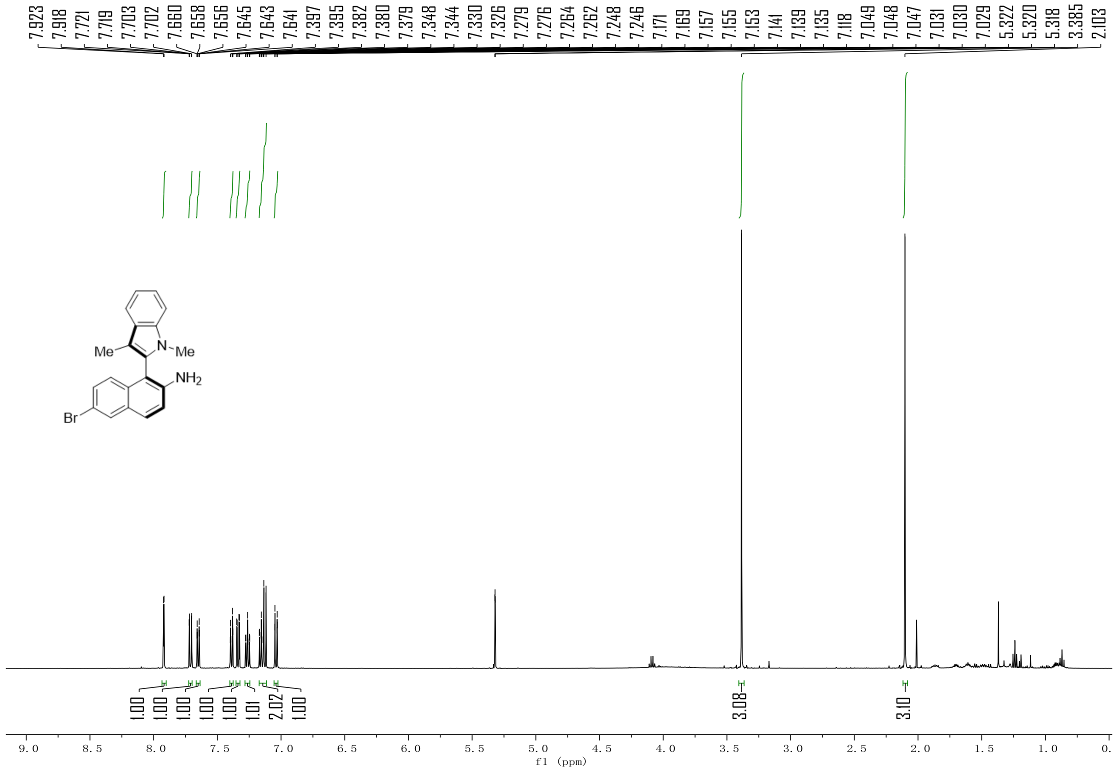

^{13}C NMR spectra of **10** (126 MHz, Methylene Chloride-d_2)

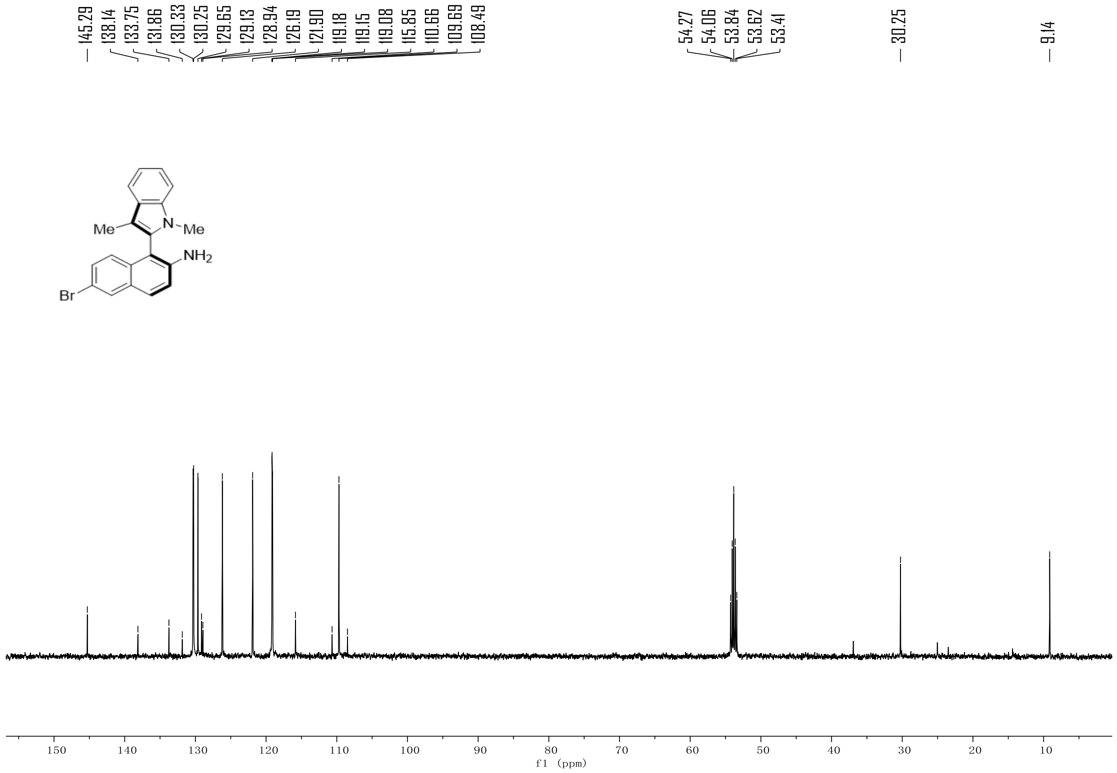

^{1}H NMR spectra of **11** (400 MHz, DMSO-d_6)

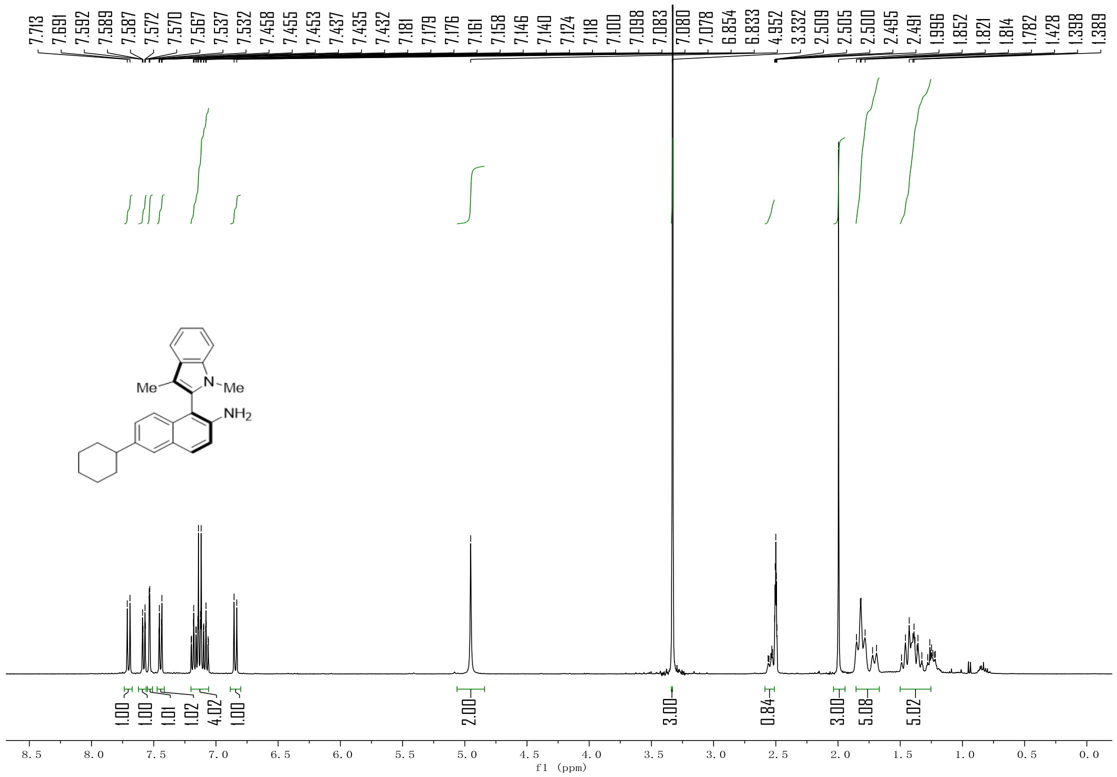

^{13}C NMR spectra of **11** (101 MHz, DMSO-d_6)

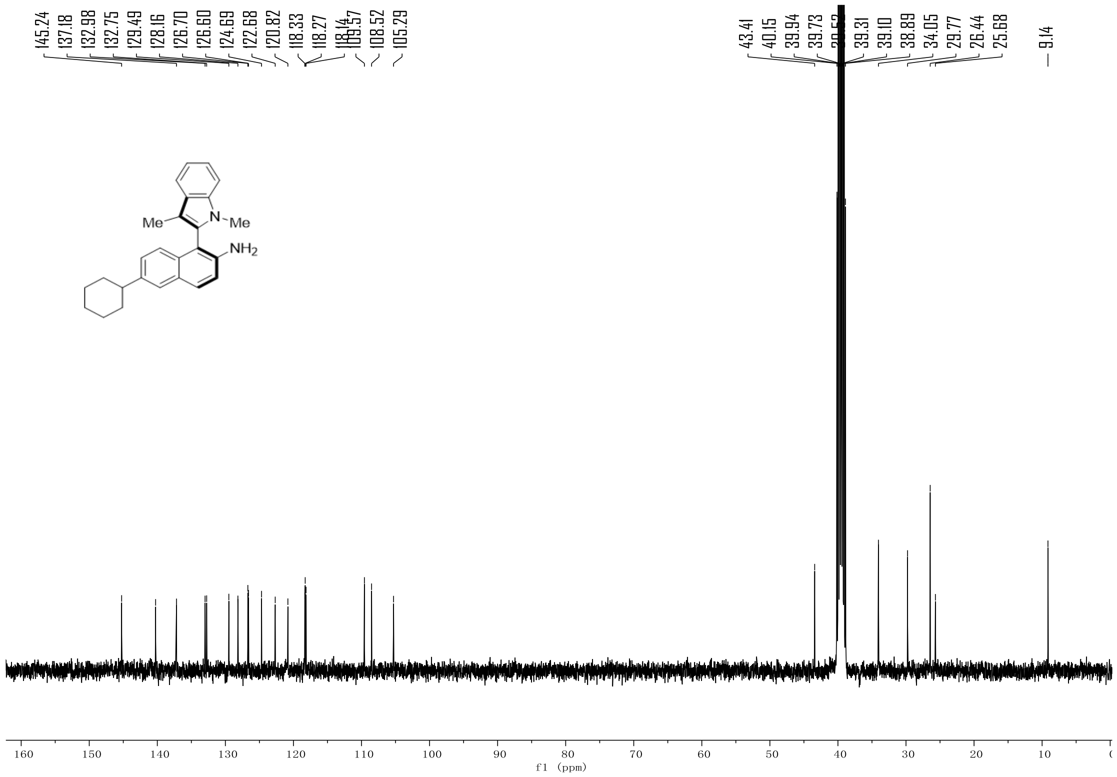

^{1}H NMR spectra of **12** (400 MHz, Methylene Chloride-d_2)

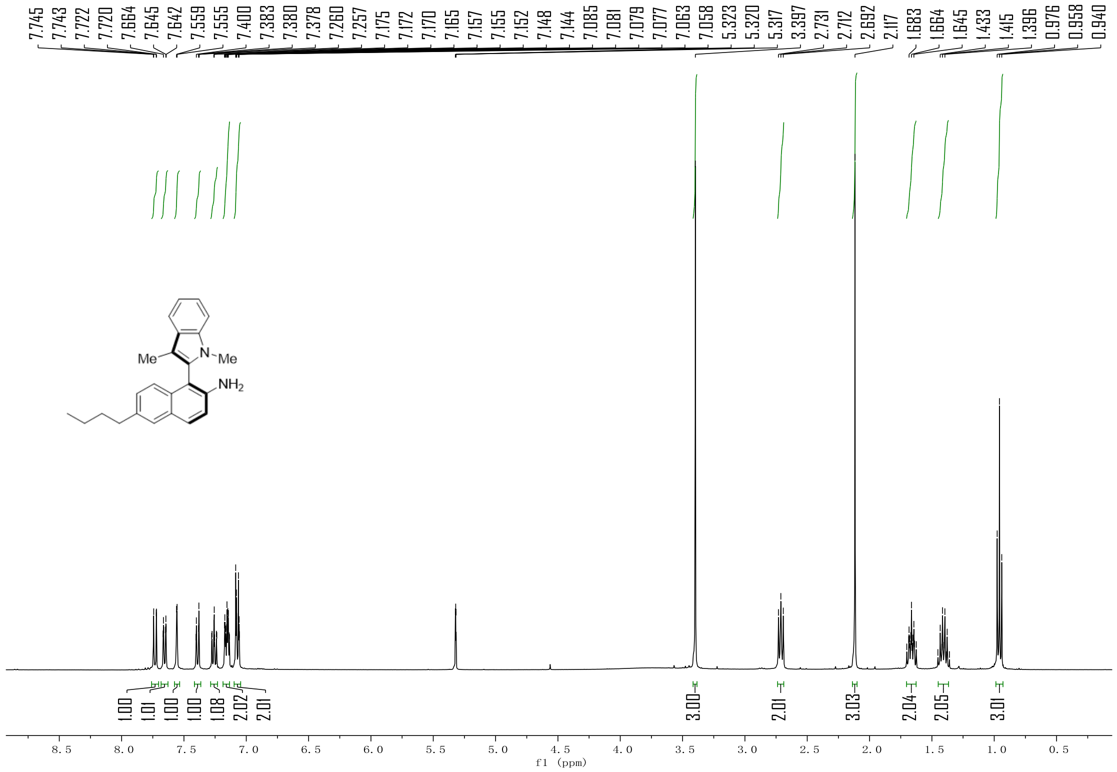

^{13}C NMR spectra of **12** (101 MHz, Methylene Chloride-d_2)

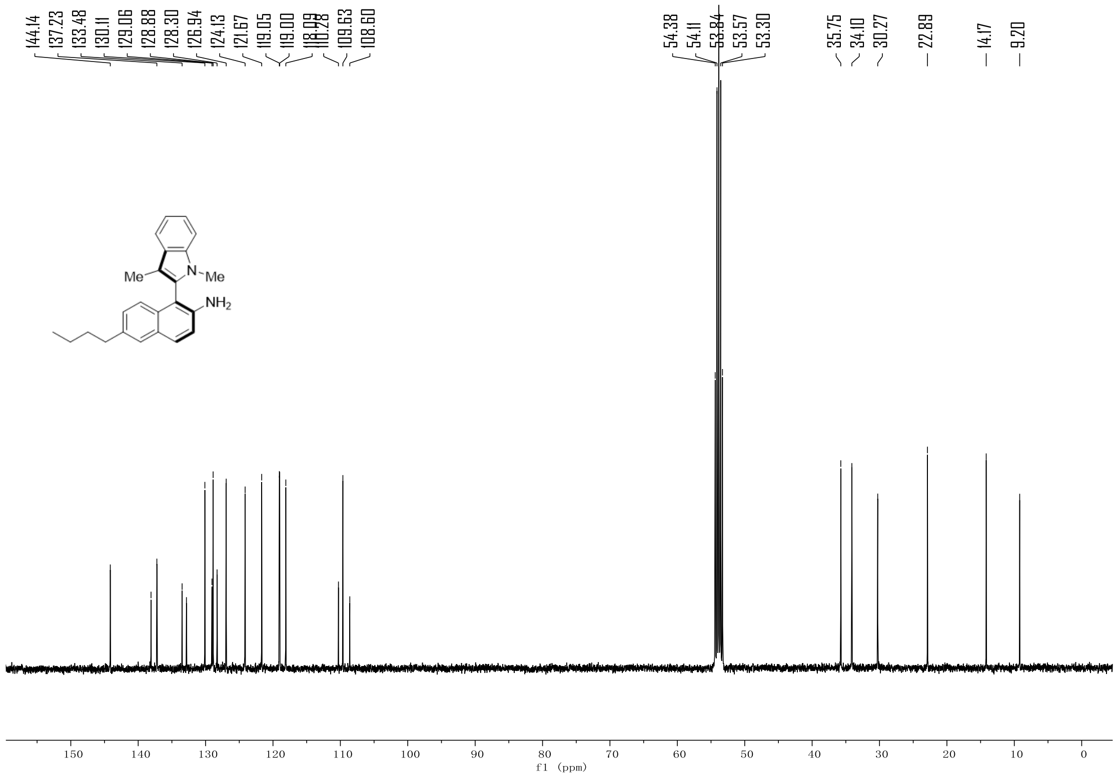

^{1}H NMR spectra of **13** (400 MHz, Methylene Chloride-d_2)

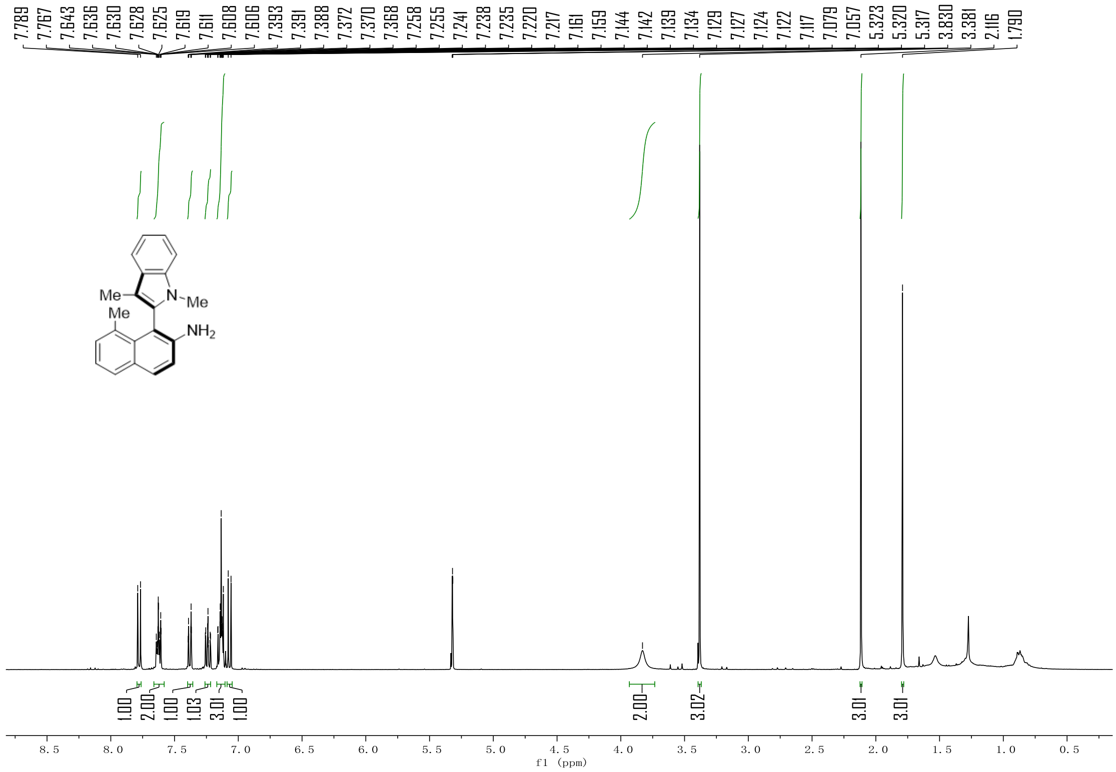

^{13}C NMR spectra of **13** (101 MHz, Methylene Chloride-d_2)

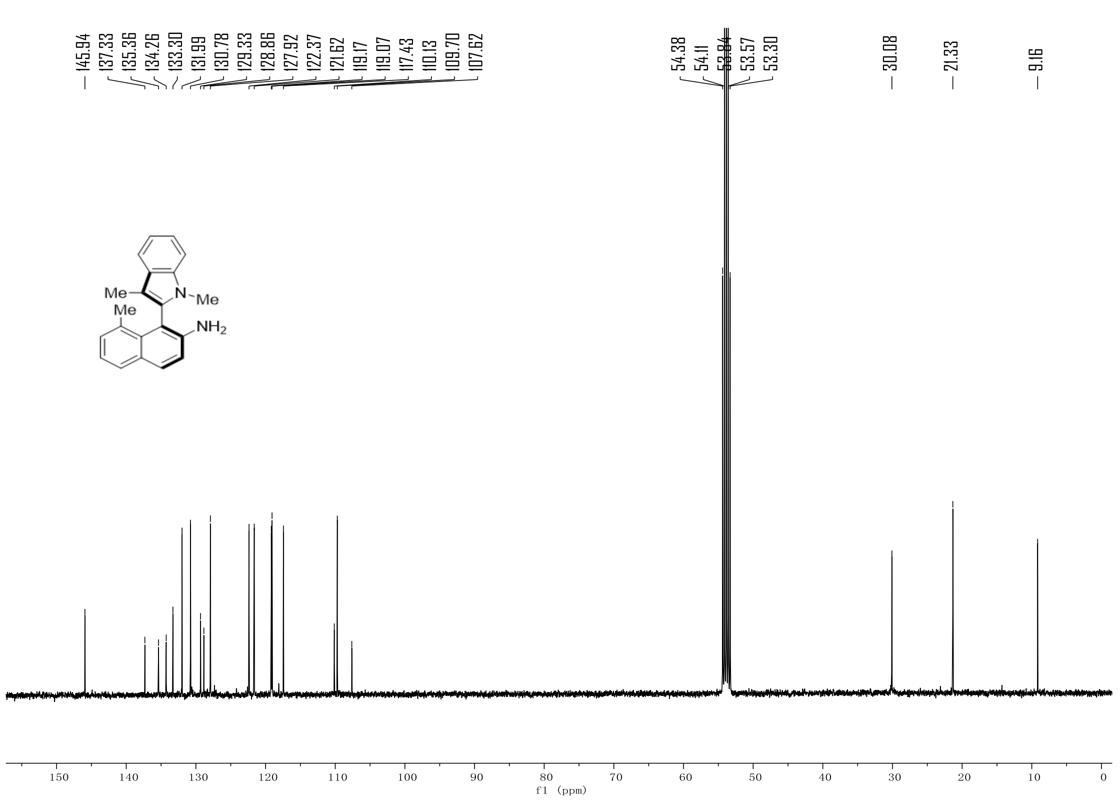

^{1}H NMR spectra of **15** (400 MHz, Methylene Chloride-d_2)

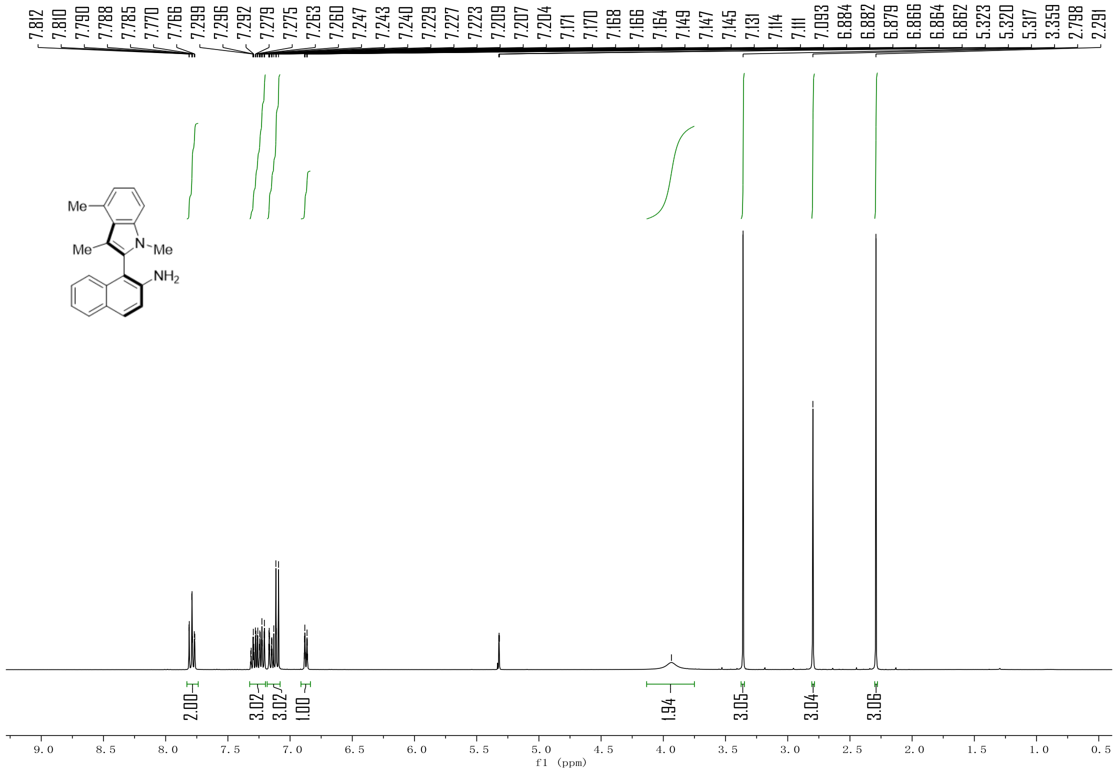

^{13}C NMR spectra of **15** (101 MHz, Methylene Chloride-d_2)

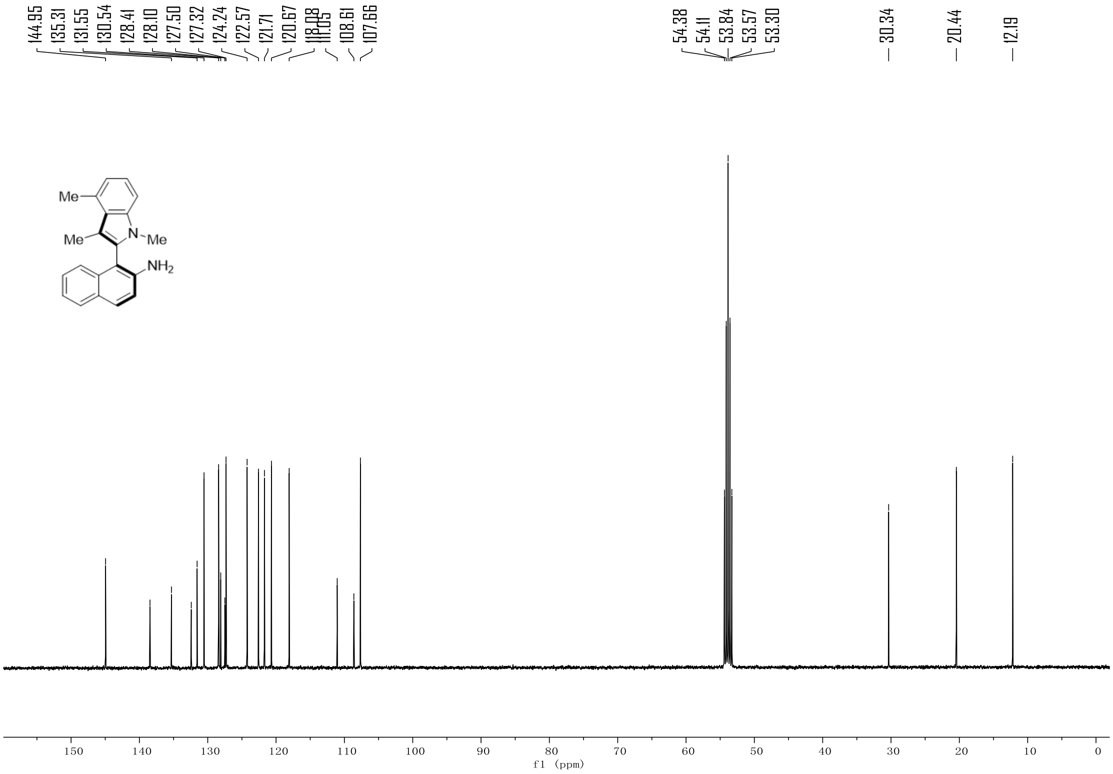

^{1}H NMR spectra of **16** (101 MHz, Methylene Chloride-d_2)

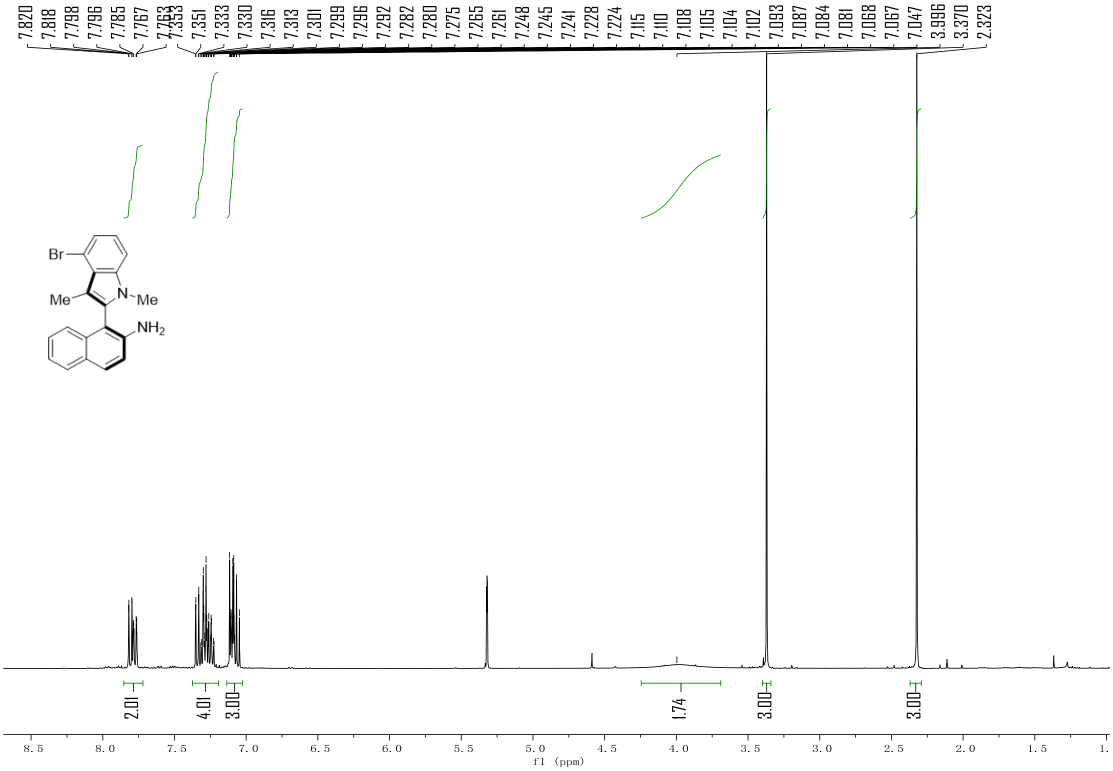

^{13}C NMR spectra of **16** (101 MHz, Methylene Chloride-d_2)

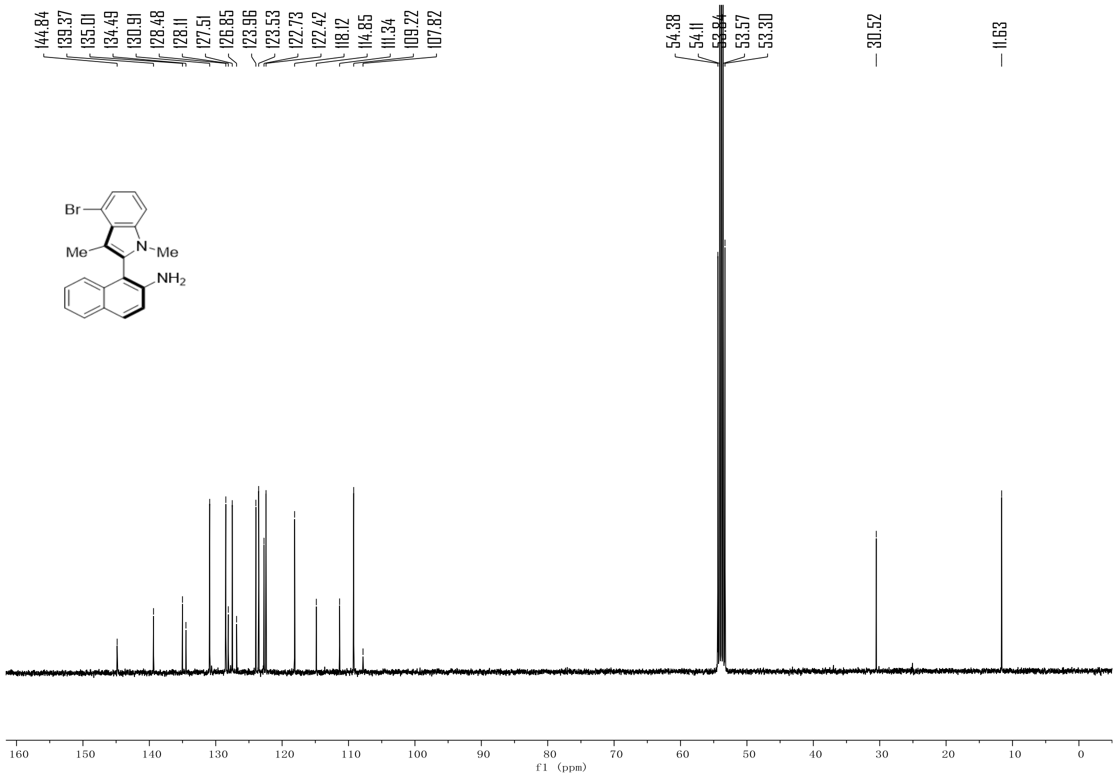

^{1}H NMR spectra of **17** (400 MHz, Methylene Chloride-d_2)

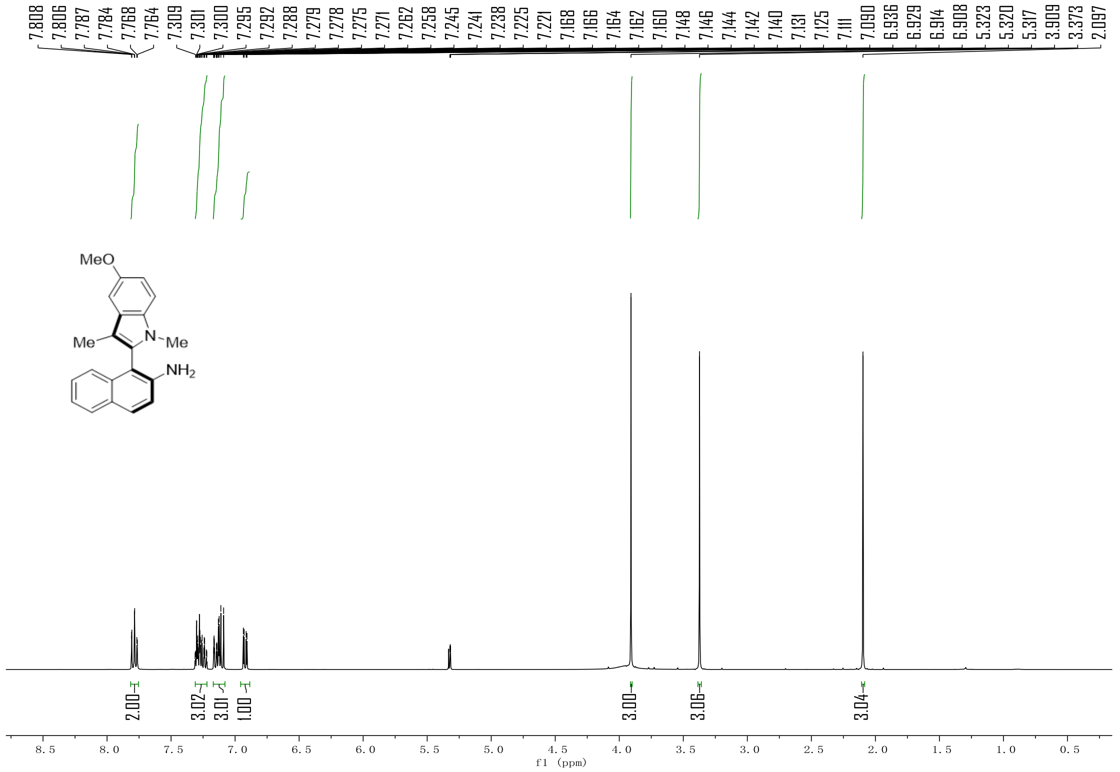

^{13}C NMR spectra of **17** (101 MHz, Methylene Chloride-d_2)

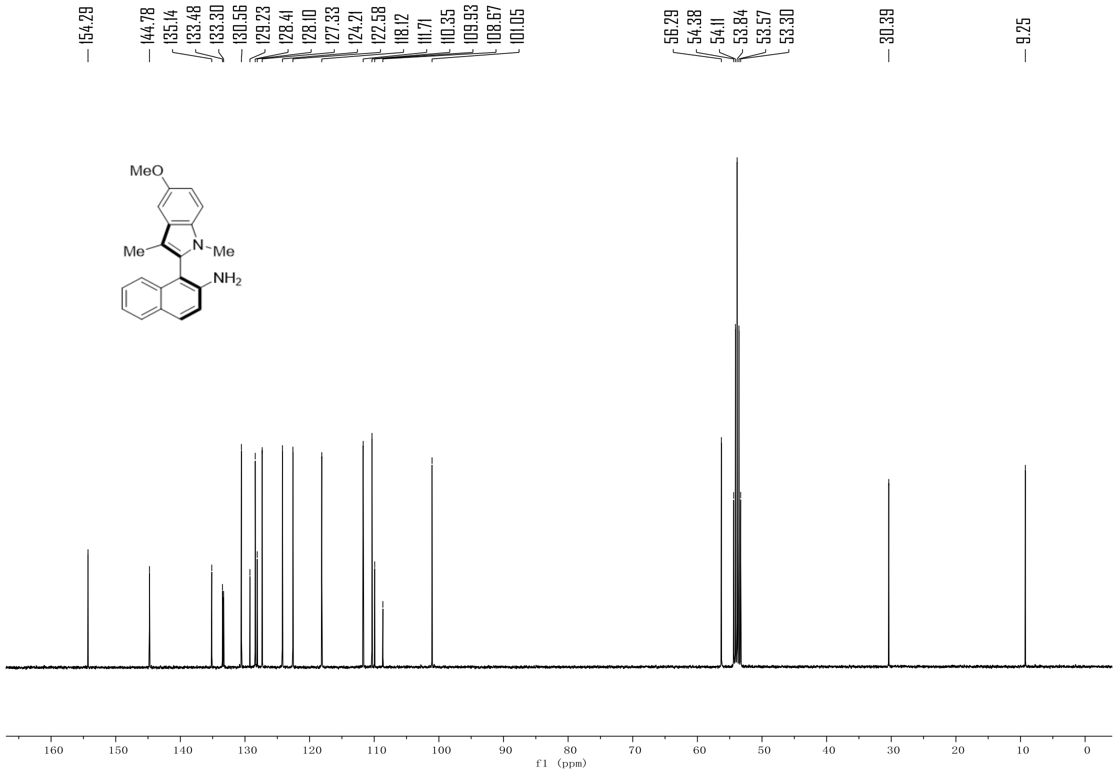

^{1}H NMR spectra of **18** (400 MHz, Methylene Chloride-d_2)

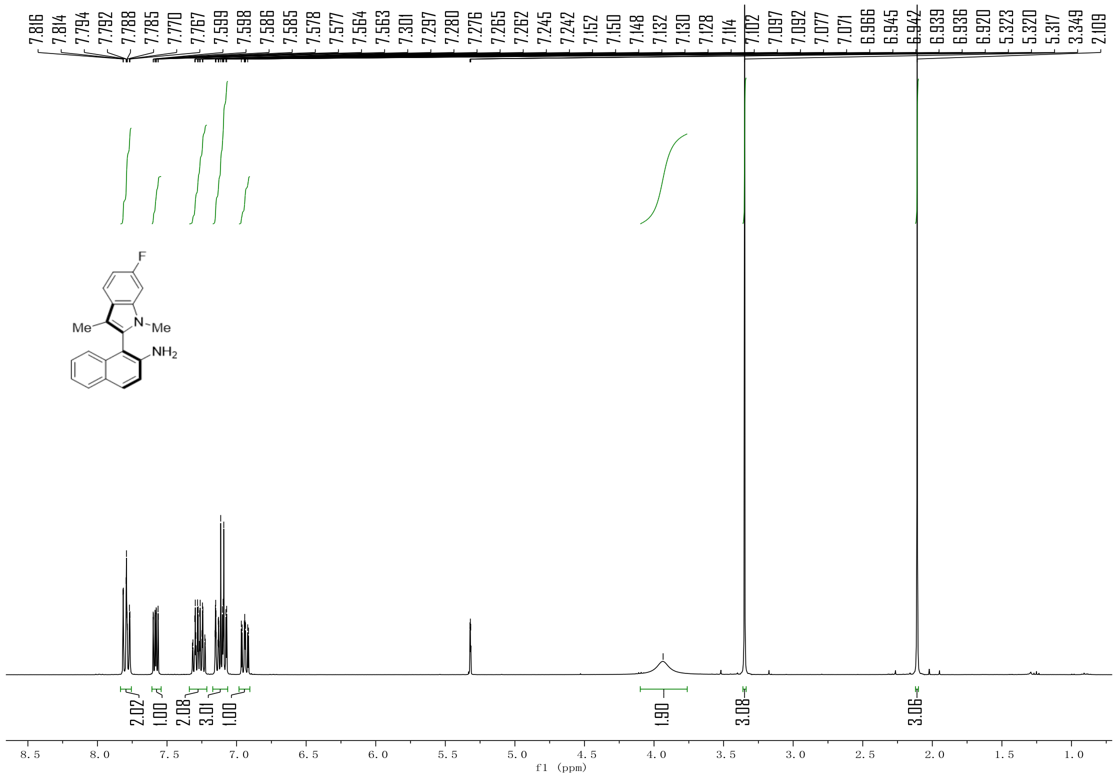

^{13}C NMR spectra of **18** (101 MHz, Methylene Chloride-d_2)

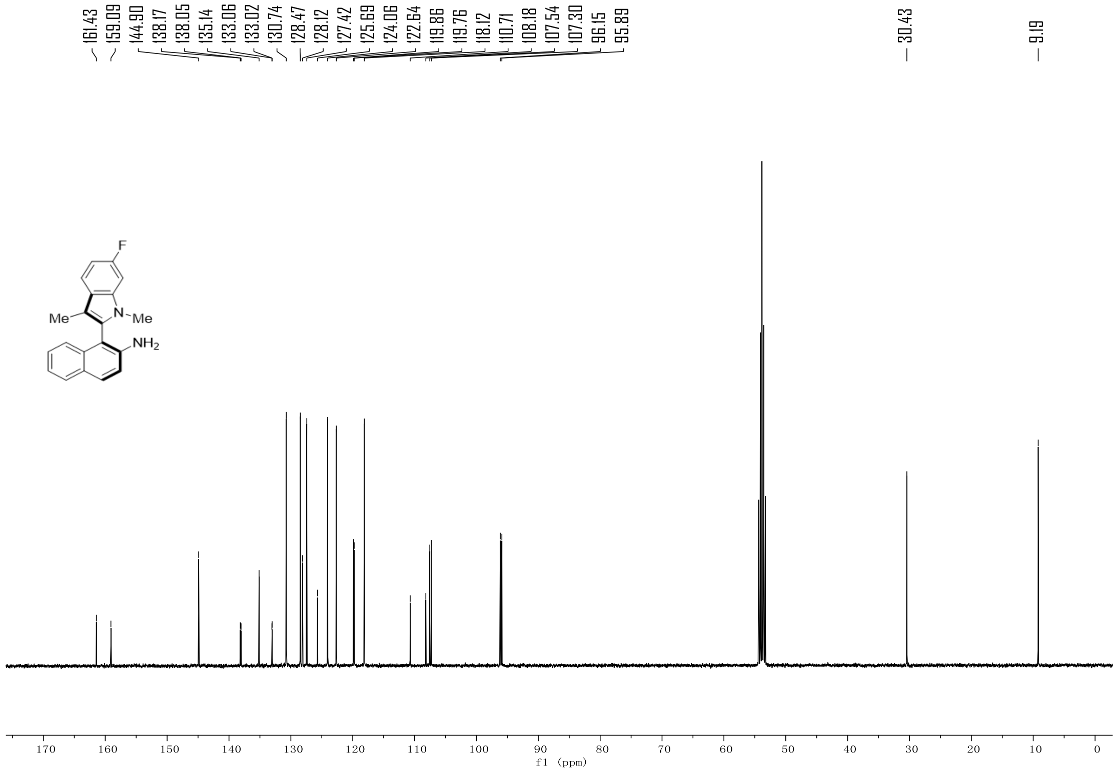

^{19}F NMR spectra of **18** (377 MHz, Methylene Chloride-d_2)

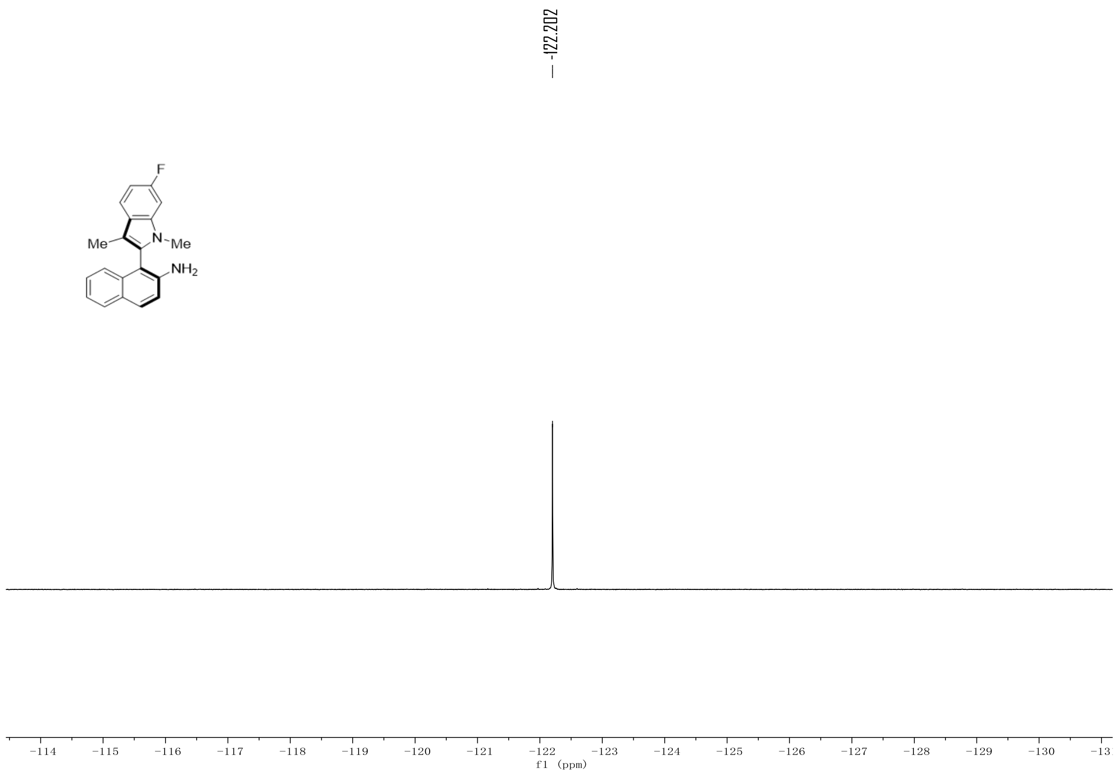

^{1}H NMR spectra of **19** (400 MHz, Methylene Chloride-d_2)

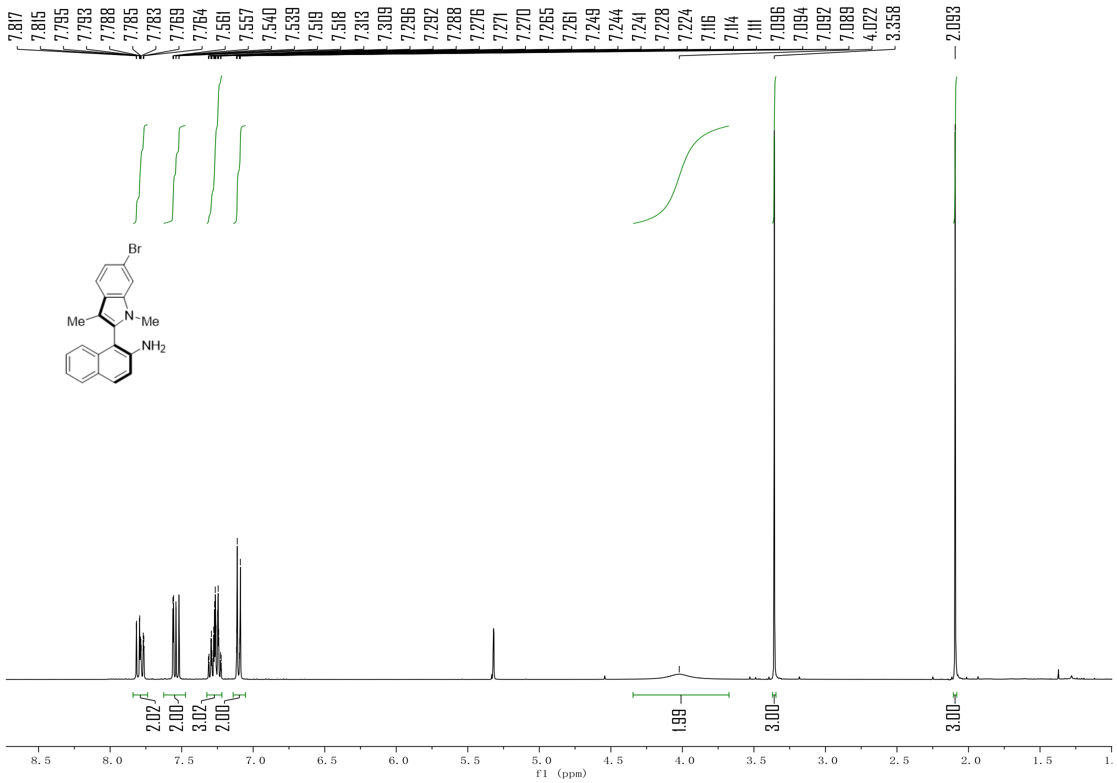

^{13}C NMR spectra of **19** (101 MHz, Methylene Chloride-d_2)

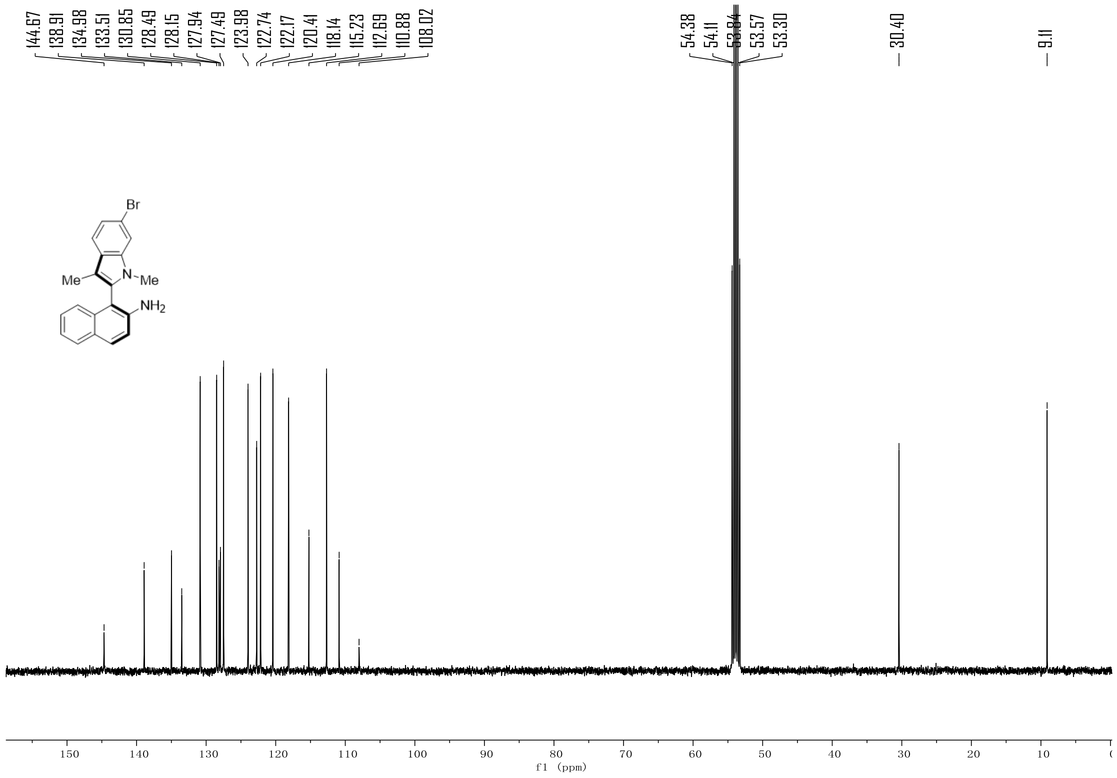

^{1}H NMR spectra of **20** (400 MHz, Methylene Chloride-d_2)

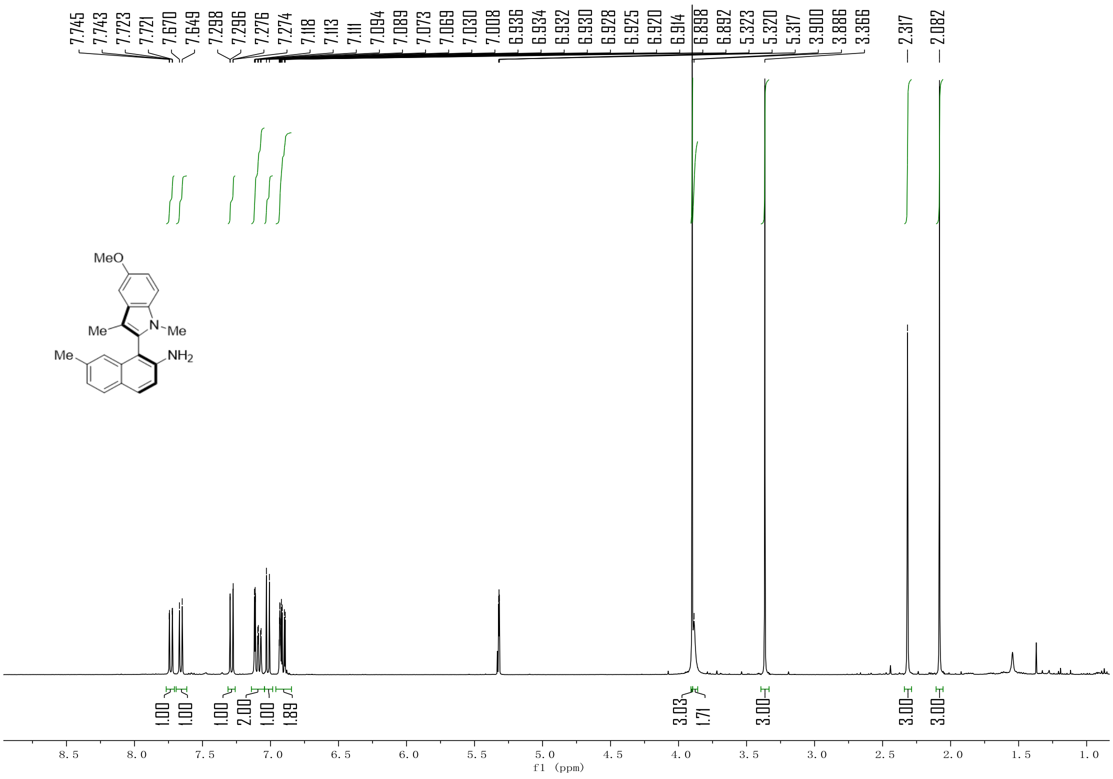

^{13}C NMR spectra of **20** (101 MHz, Methylene Chloride-d_2)

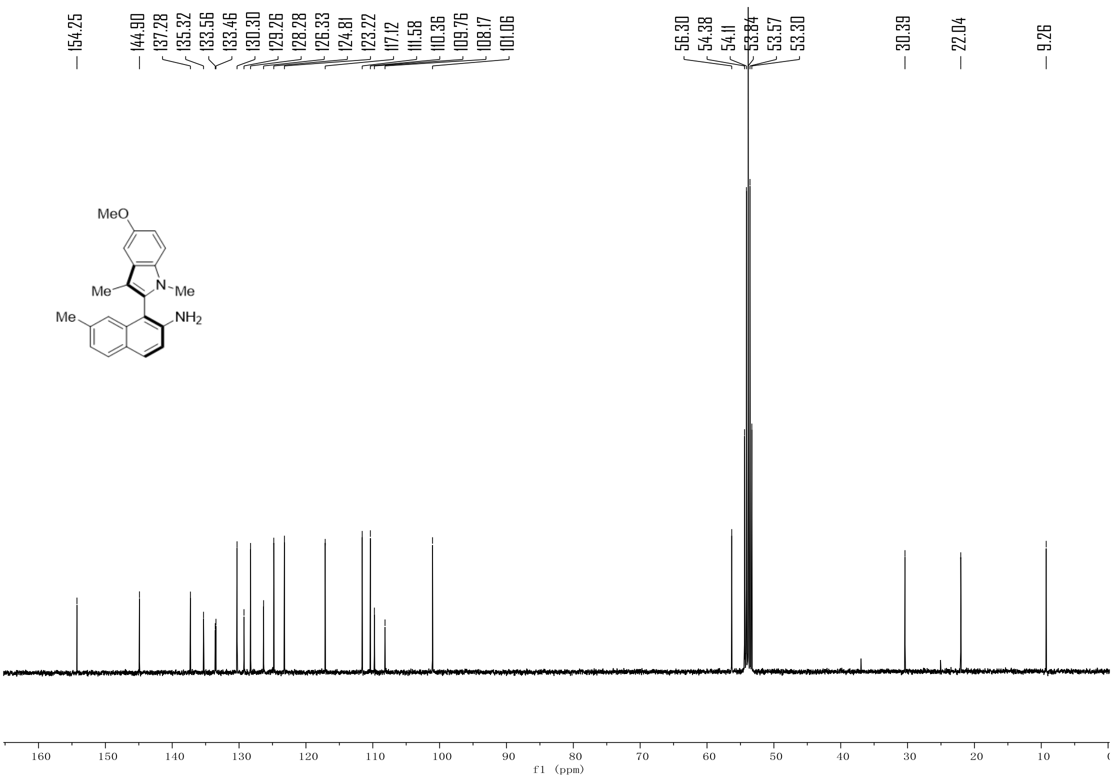

^{1}H NMR spectra of **21** (400 MHz, Methylene Chloride-d_2)

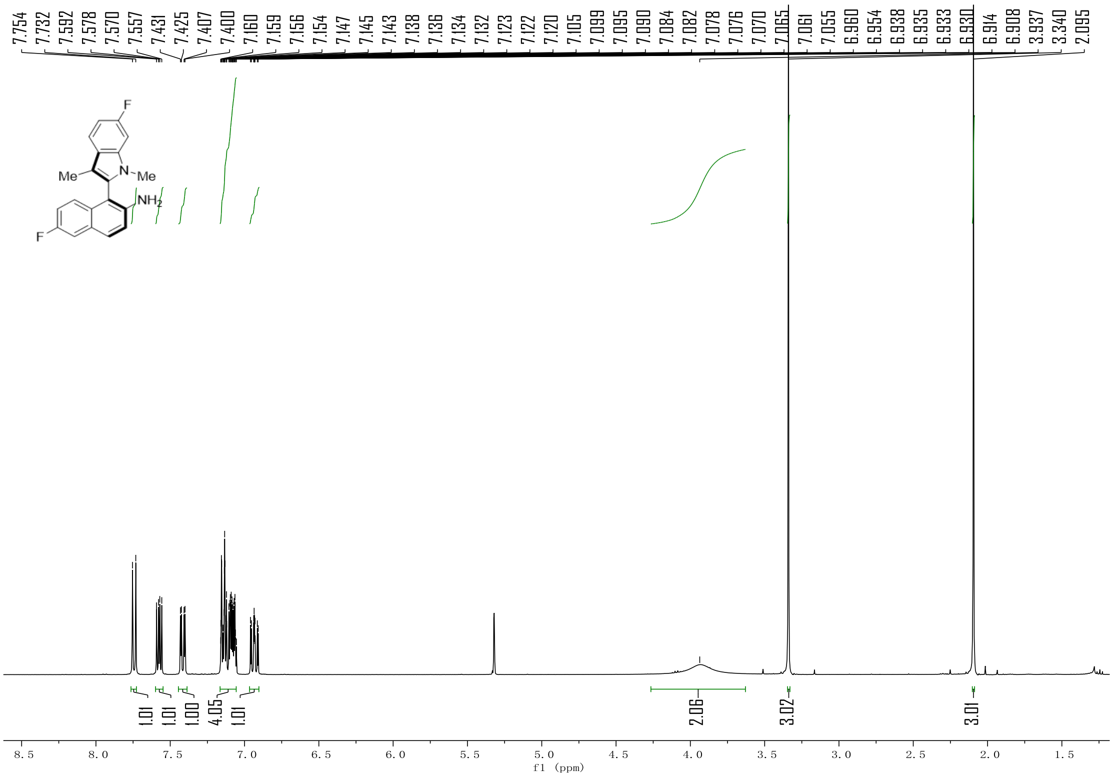

^{13}C NMR spectra of **21** (101 MHz, Methylene Chloride-d_2)

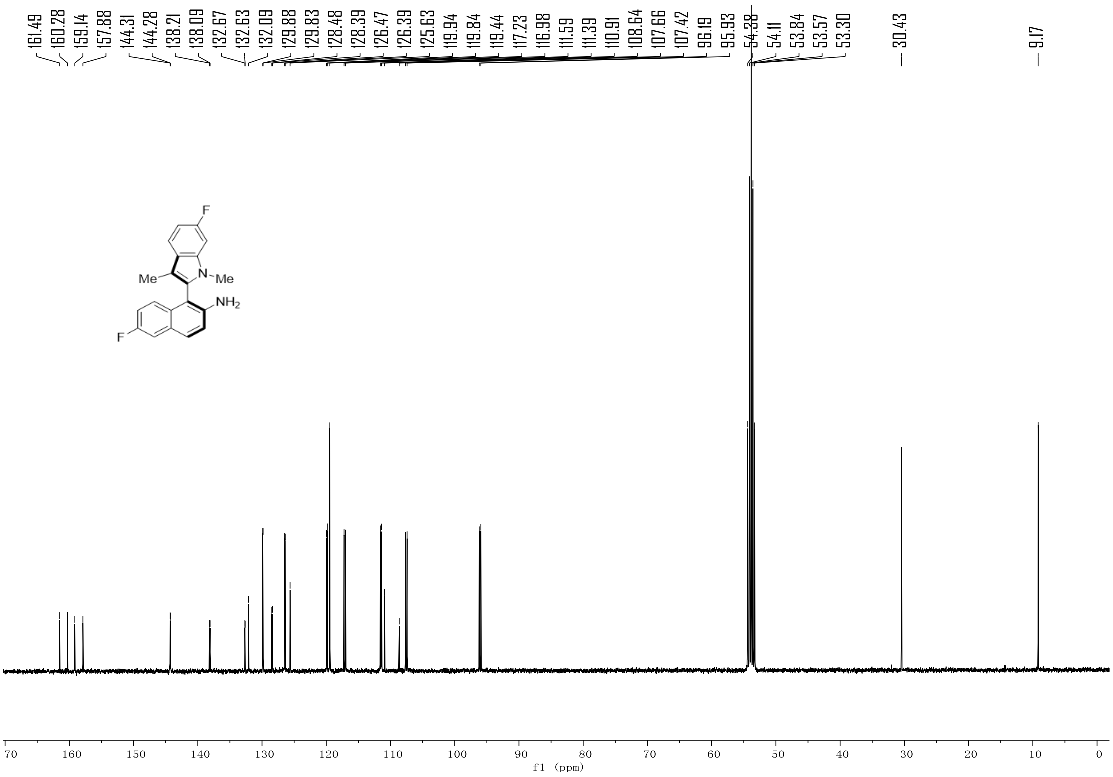

^{19}F NMR spectra of **21** (377 MHz, Methylene Chloride-d_2)

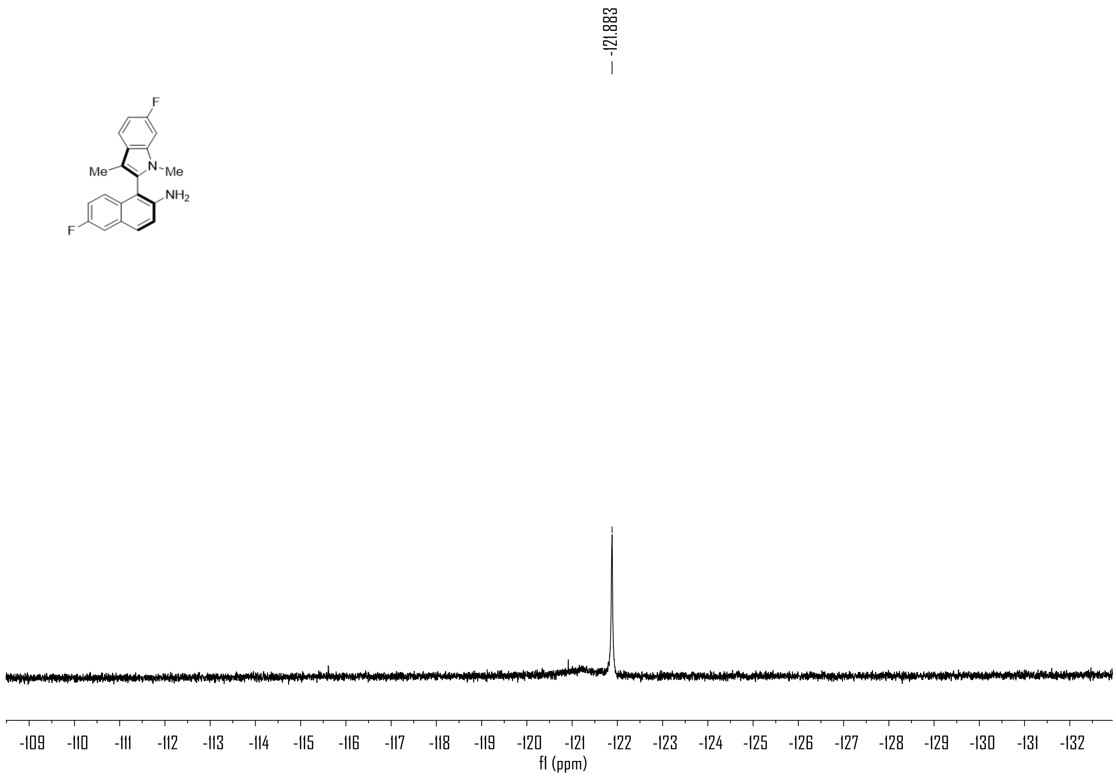

^{1}H NMR spectra of **22** (400 MHz, Methylene Chloride-d_2)

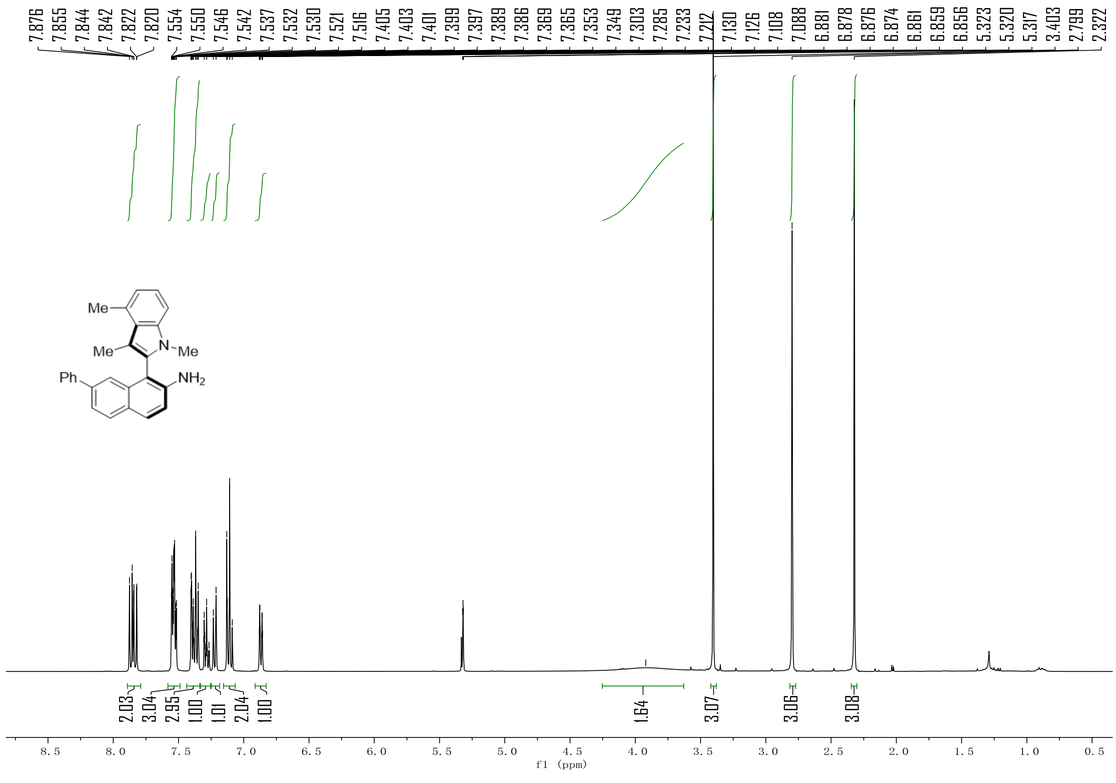

^{13}C NMR spectra of **22** (101 MHz, Methylene Chloride-d_2)

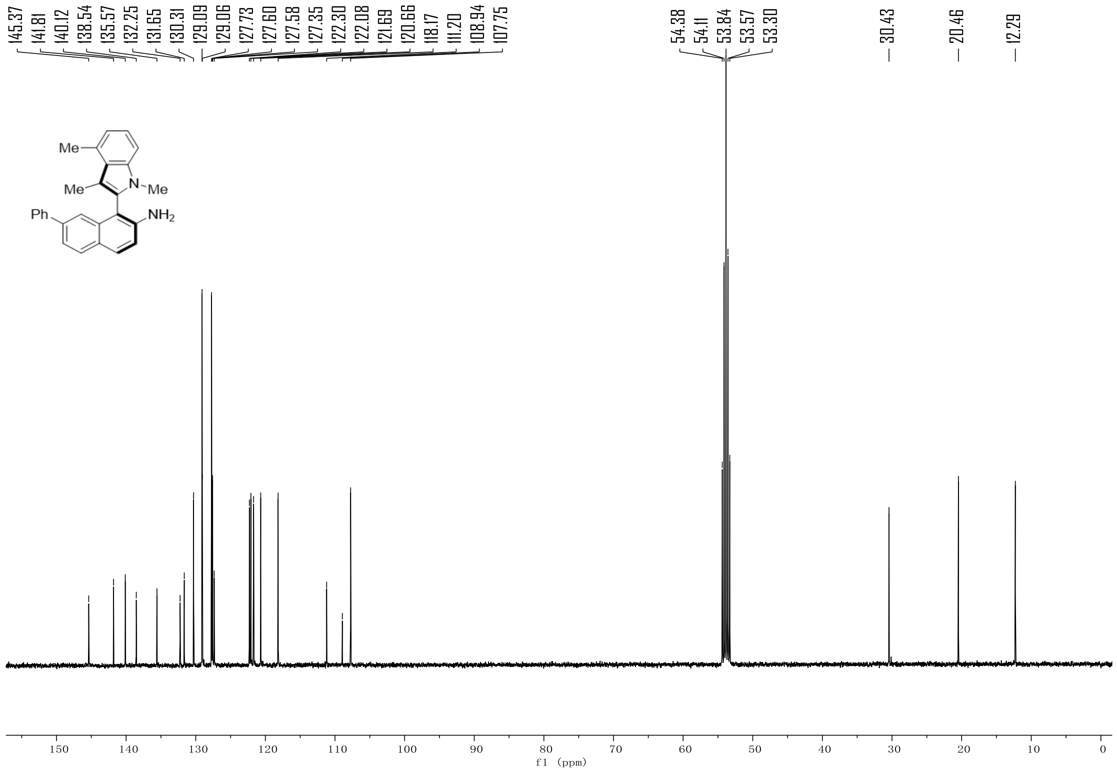

^{1}H NMR spectra of **23** (400 MHz, Methylene Chloride-d_2)

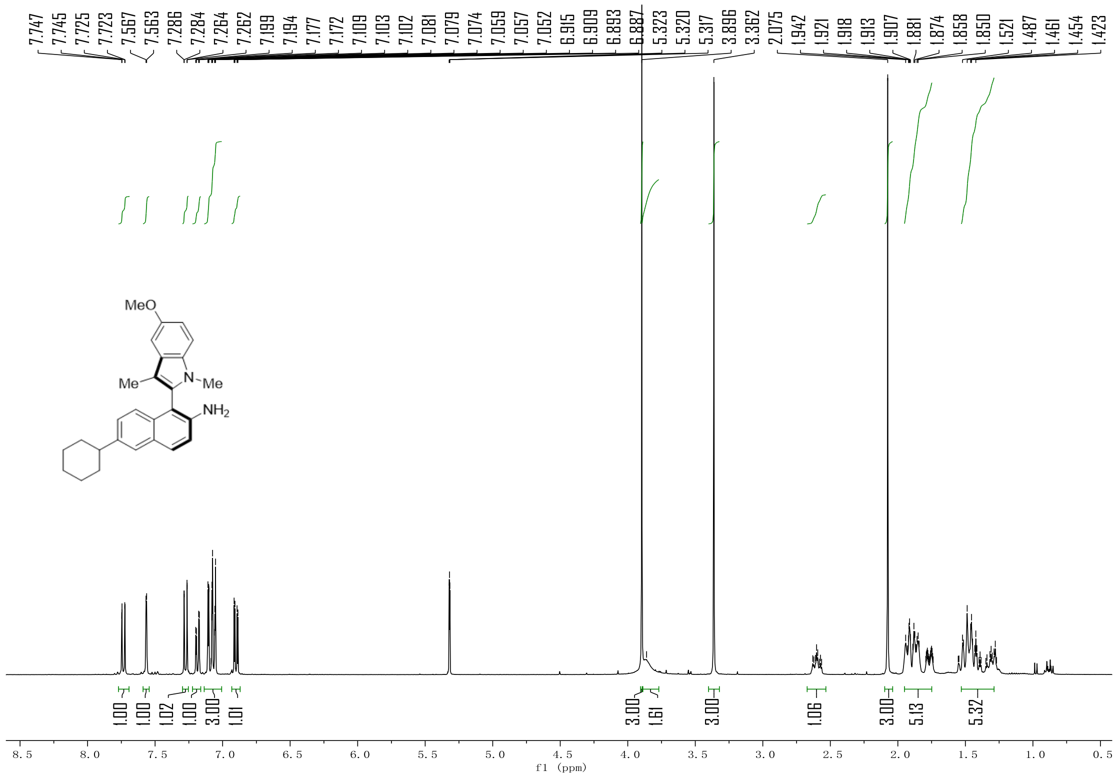

^{13}C NMR spectra of **23** (101 MHz, Methylene Chloride-d_2)

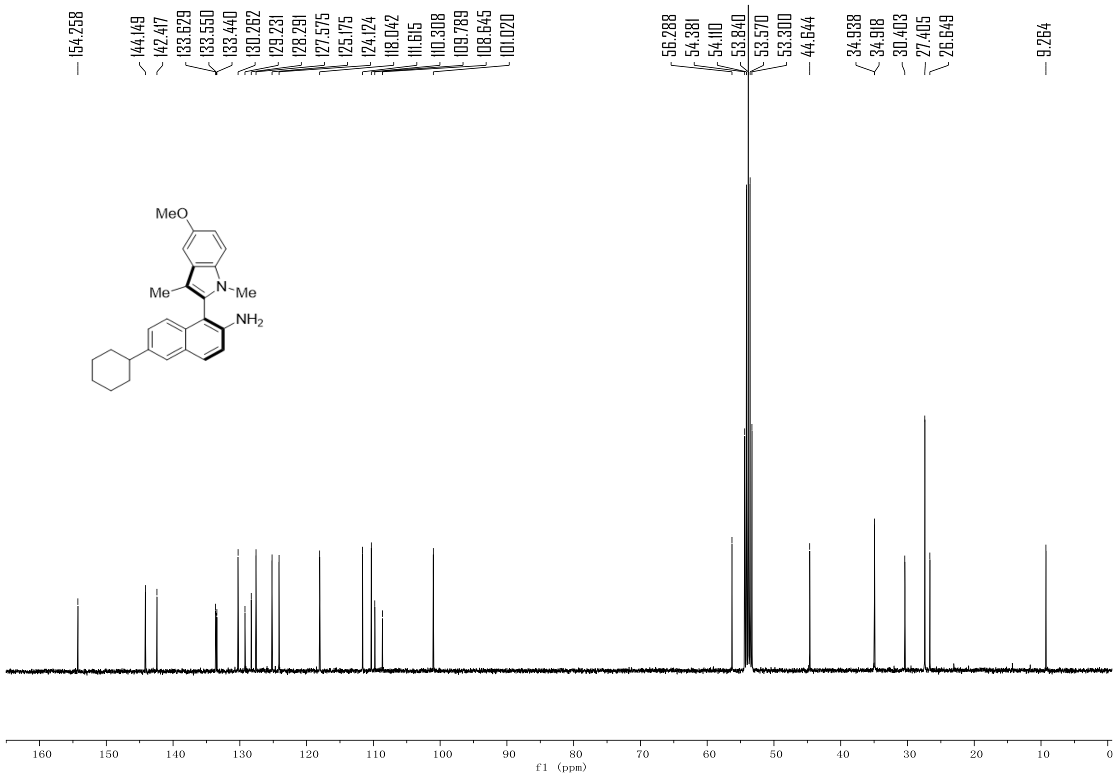

^{1}H NMR spectra of **24** (400 MHz, Methylene Chloride-d_2)

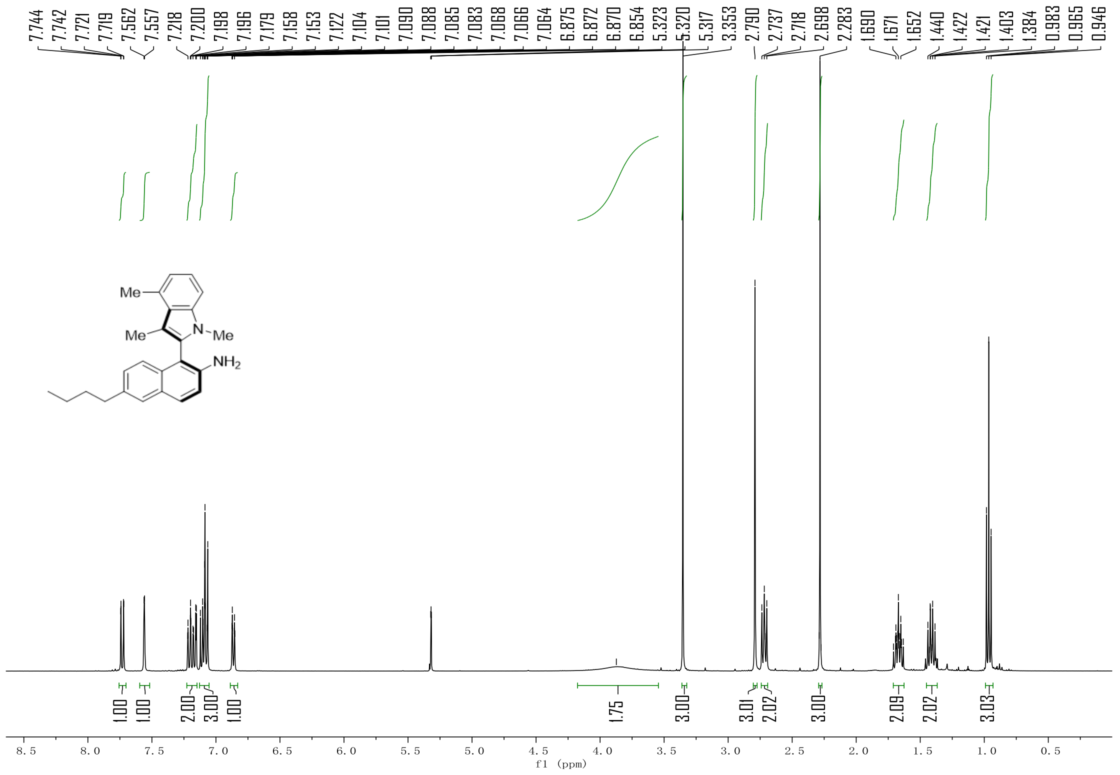

^{13}C NMR spectra of **24** (101 MHz, Methylene Chloride-d_2)

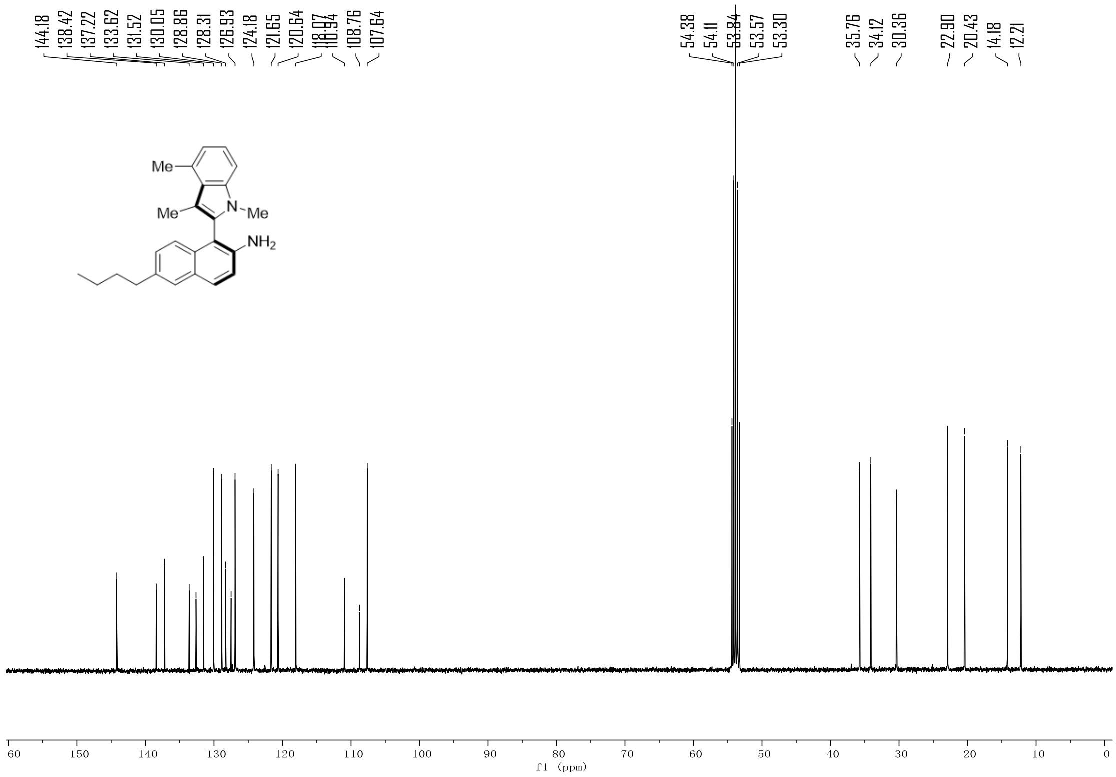

^{1}H NMR spectra of **25** (400 MHz, Methylene Chloride-d_2)

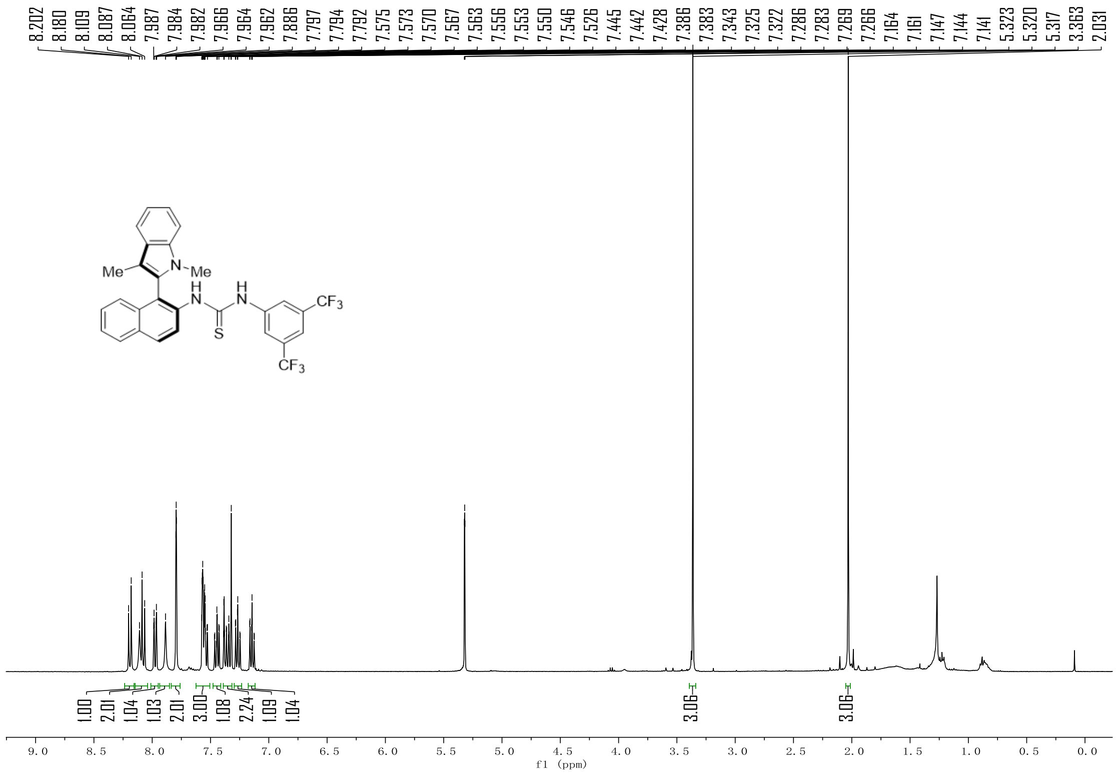

^{13}C NMR spectra of **25** (101 MHz, Methylene Chloride-d_2)

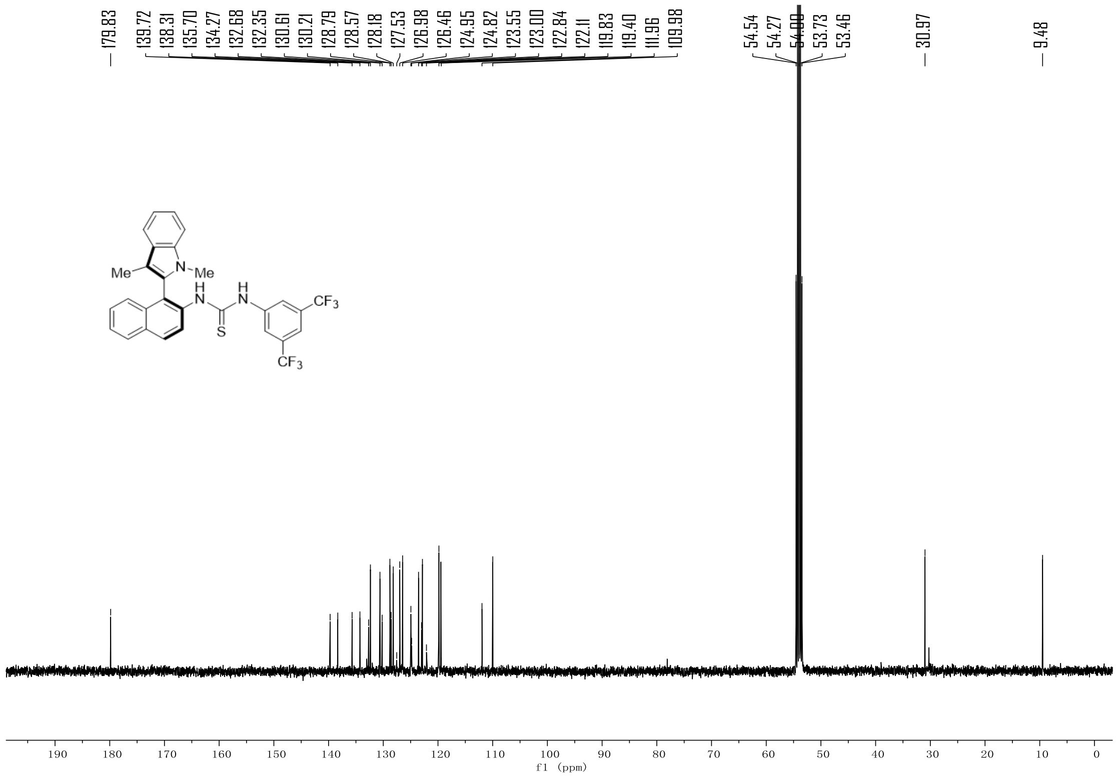

^{19}F NMR spectra of **25** (377 MHz, Methylene Chloride-d_2)

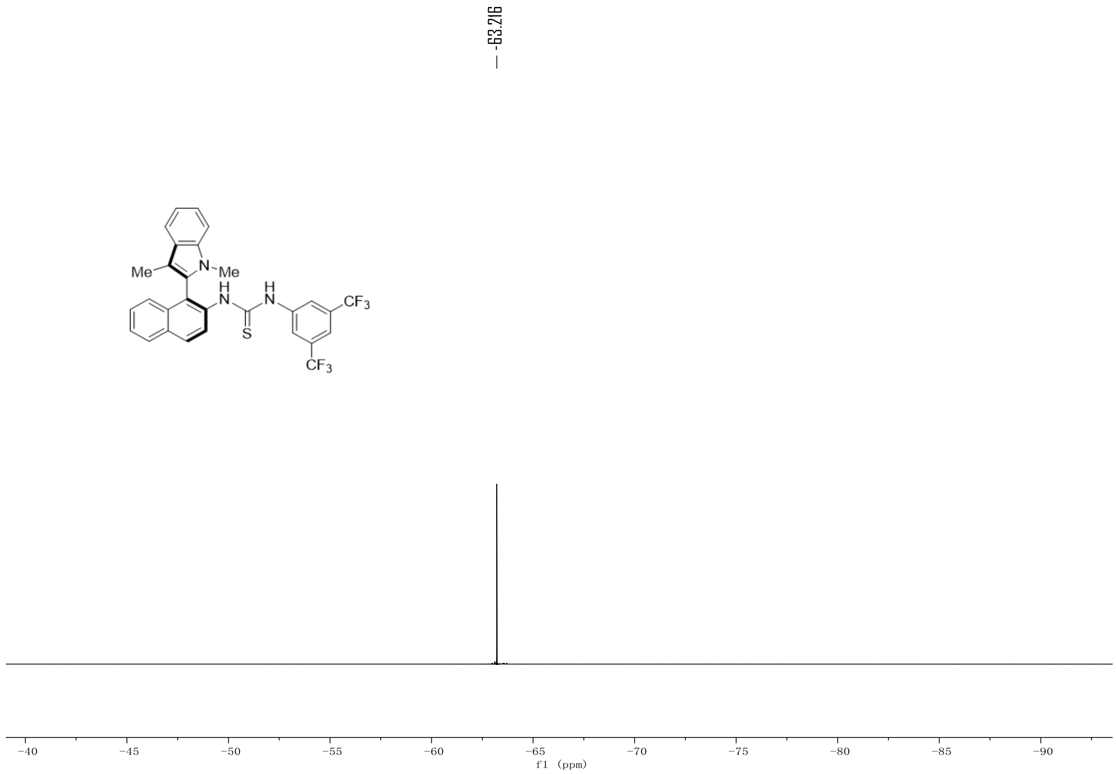

^{1}H NMR spectra of **26** (500 MHz, Methylene Chloride-d_2)

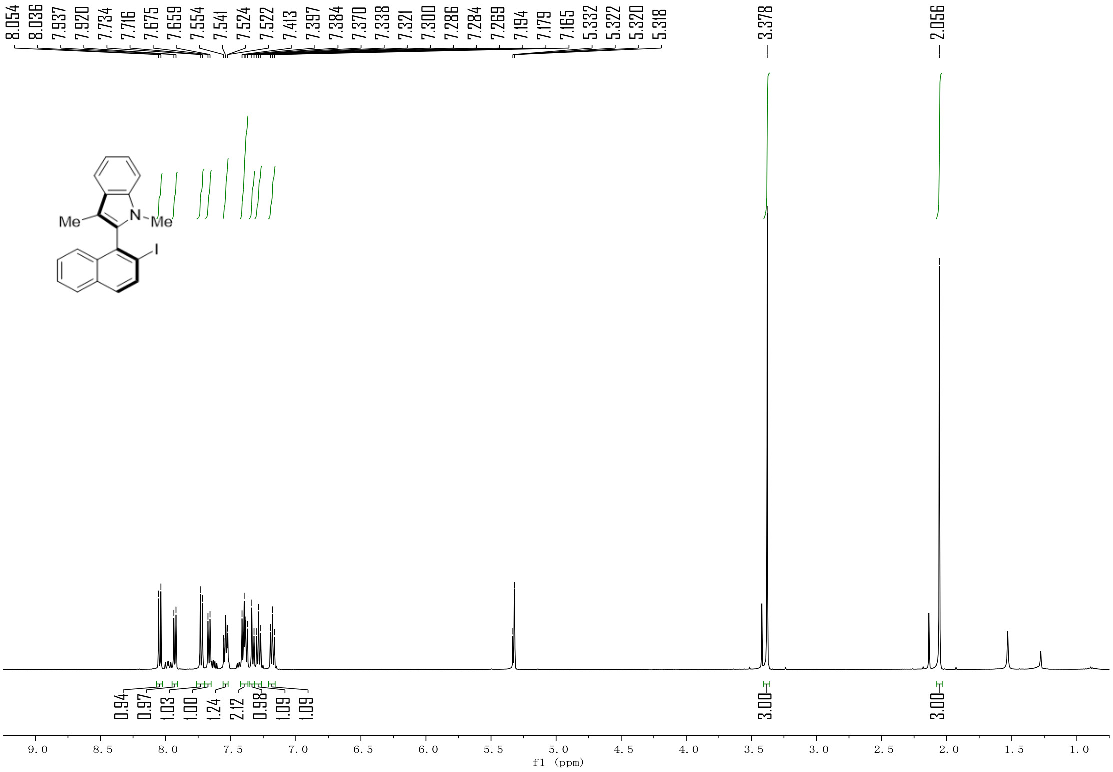

^{13}C NMR spectra of **26** (126 MHz, Methylene Chloride-d_2)

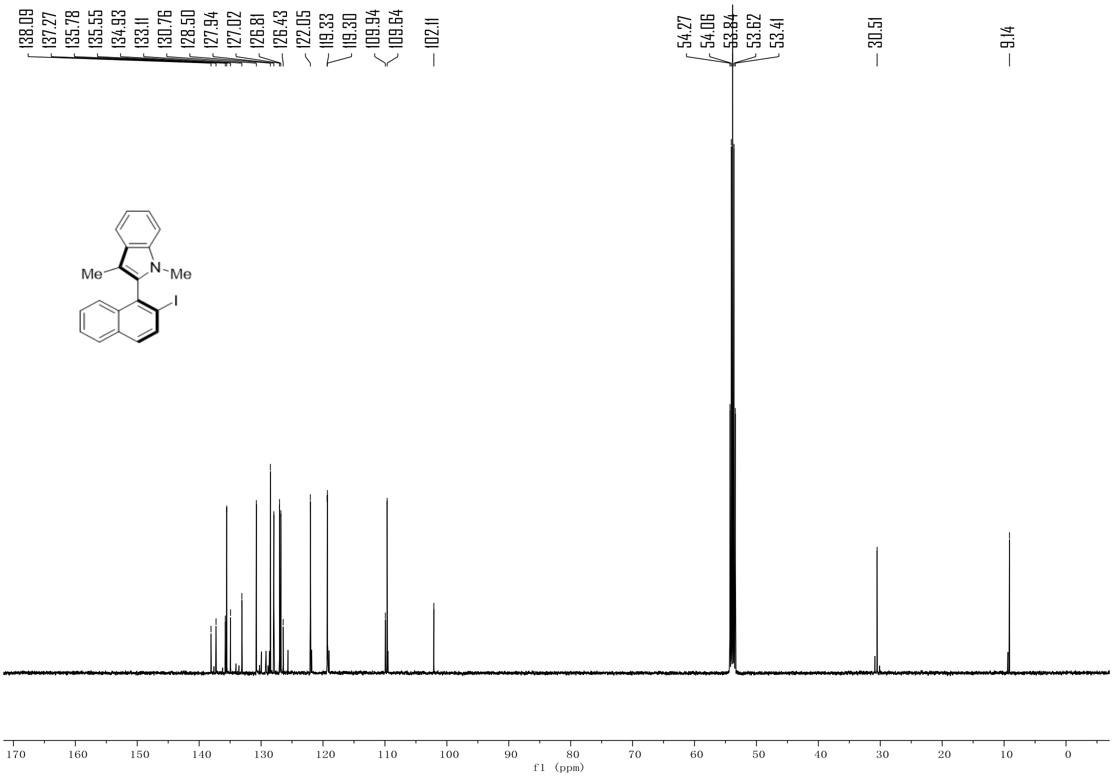

^{1}H NMR spectra of **27** (600 MHz, Methylene Chloride-d_2)

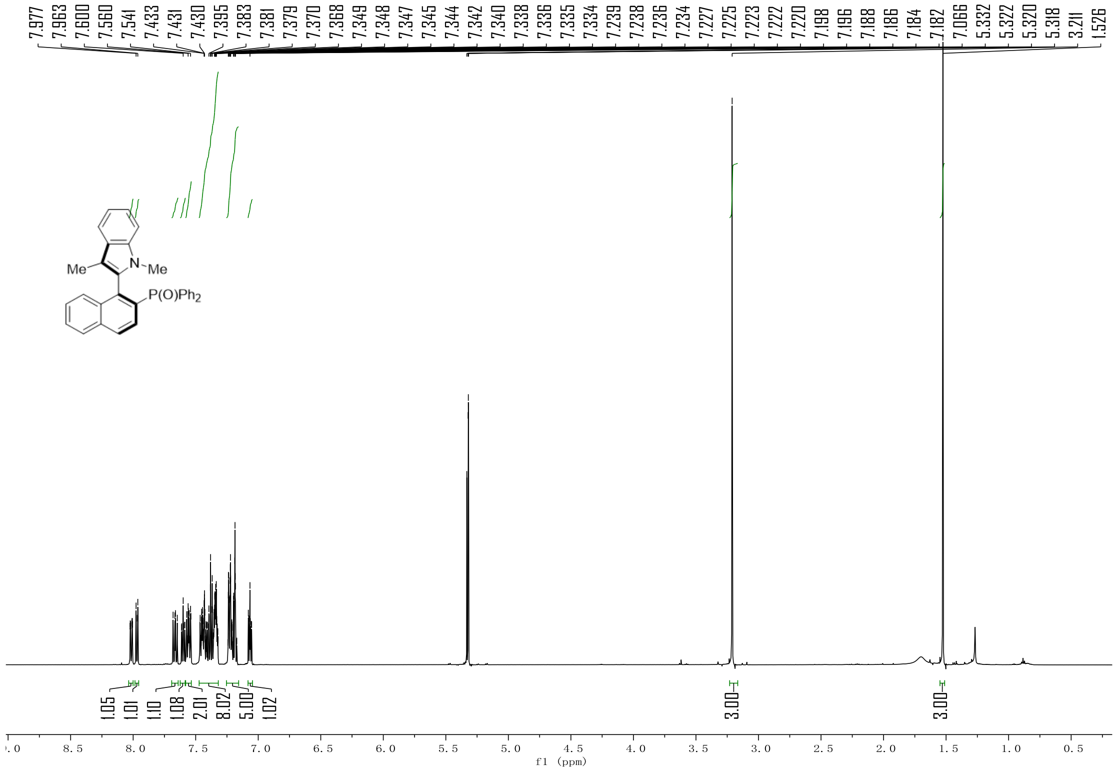

^{13}C NMR spectra of **27** (151 MHz, Methylene Chloride-d_2)

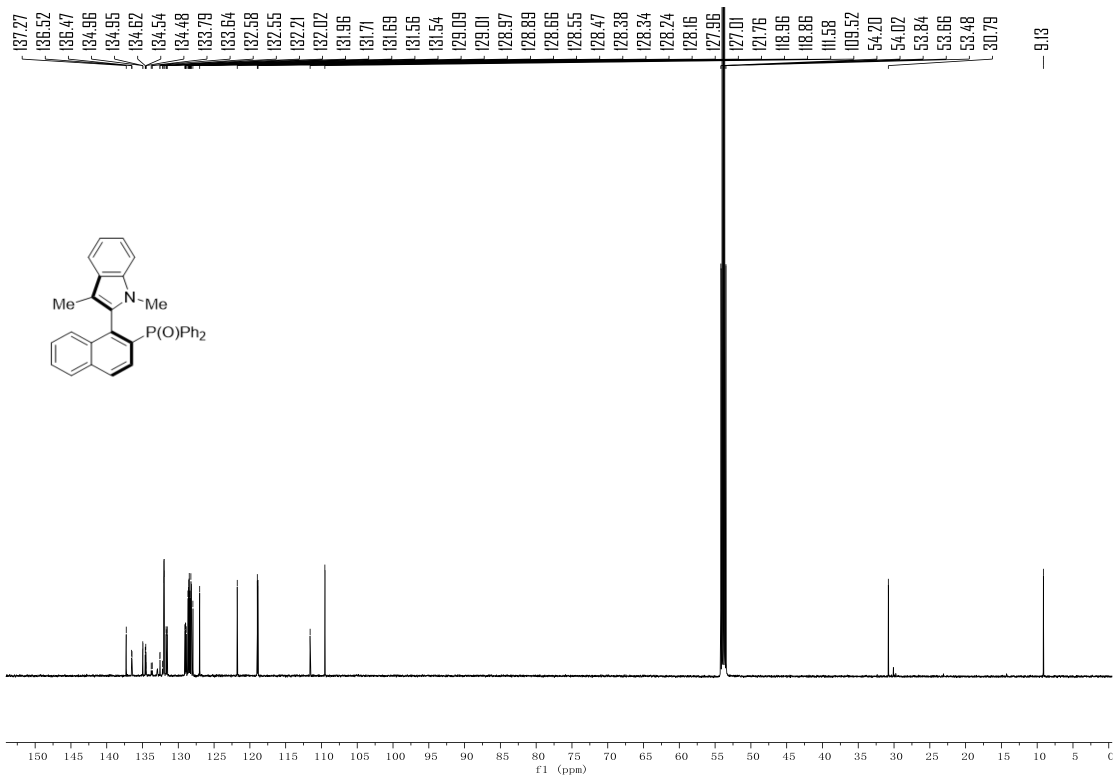

^{31}P NMR spectra of **27** (243 MHz, Methylene Chloride-d_2)

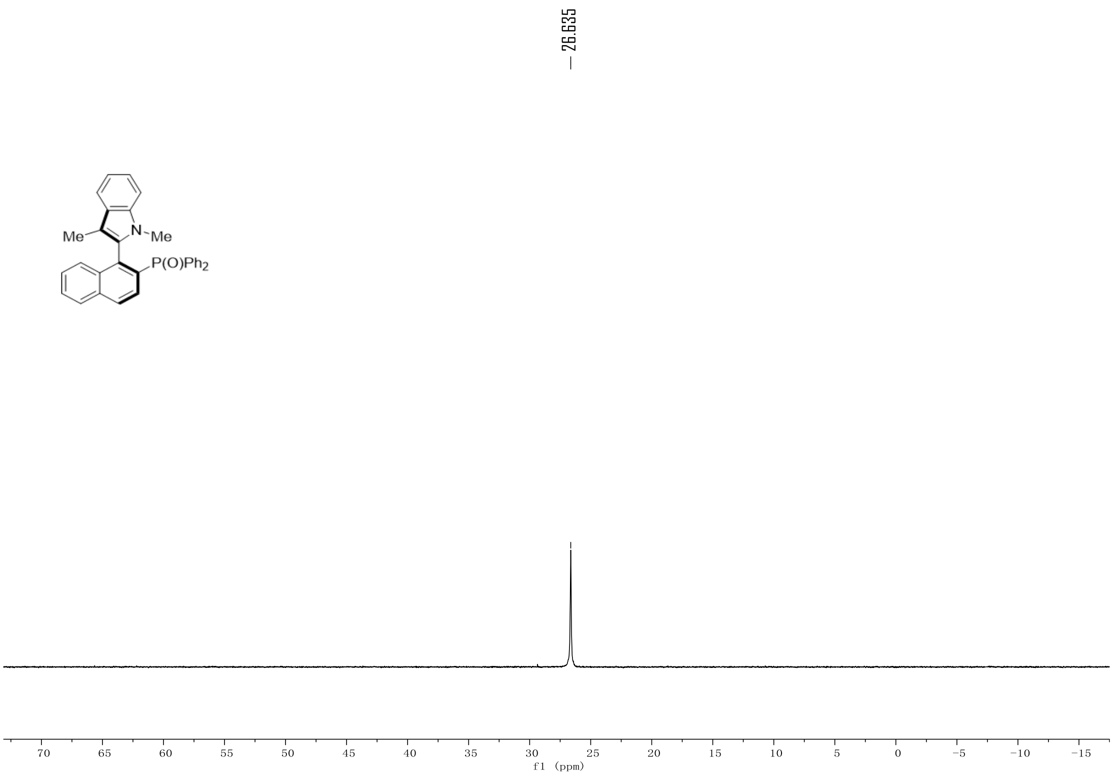

^{1}H NMR spectra of **28** (500 MHz, Methylene Chloride-d_2)

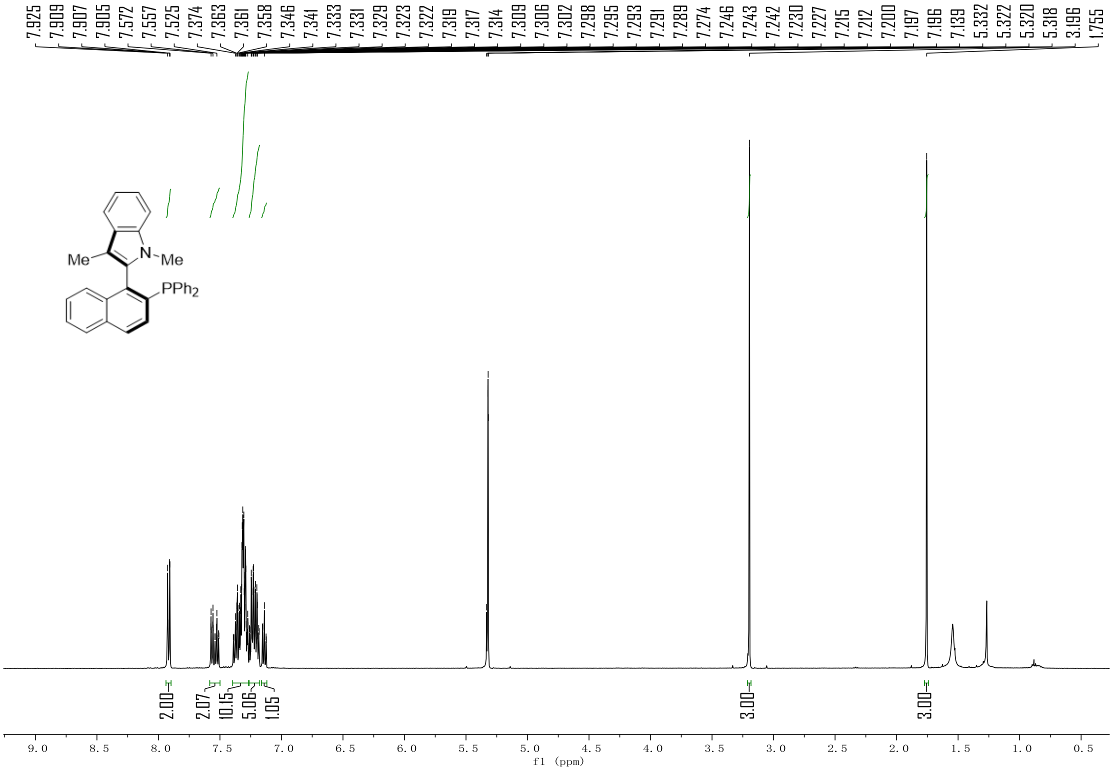

^{13}C NMR spectra of **28** (126 MHz, Methylene Chloride-d_2)

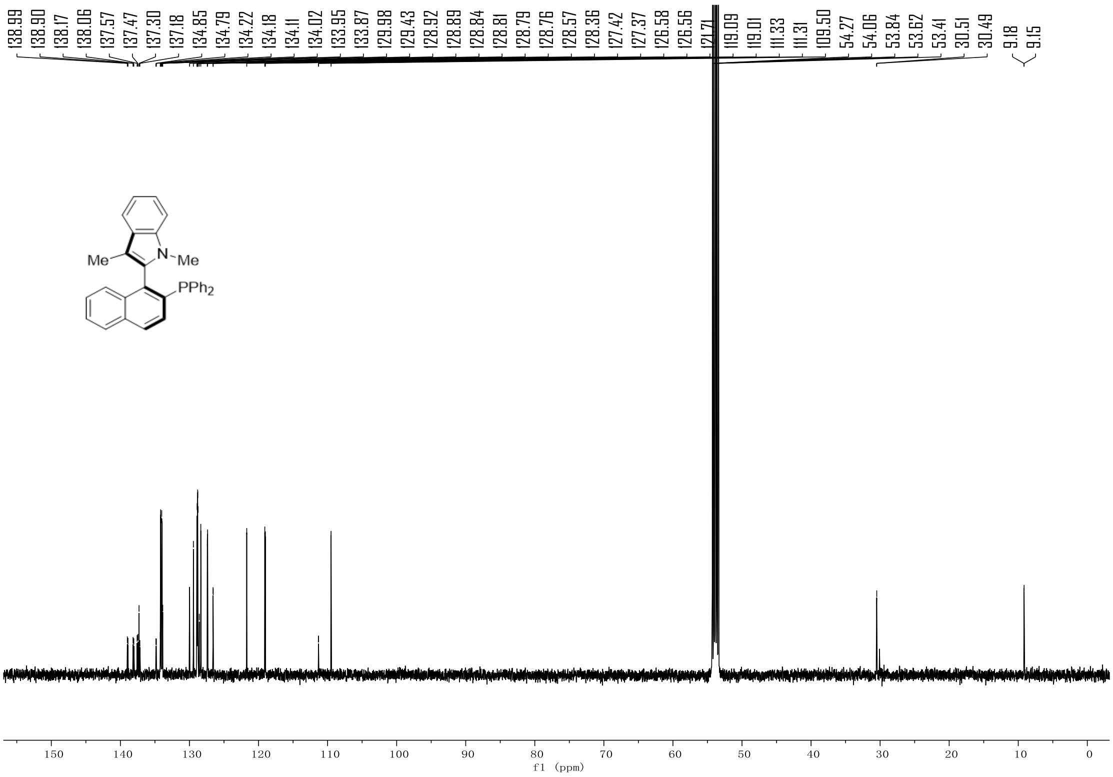

^{31}P NMR spectra of **28** (202 MHz, Methylene Chloride-d_2)

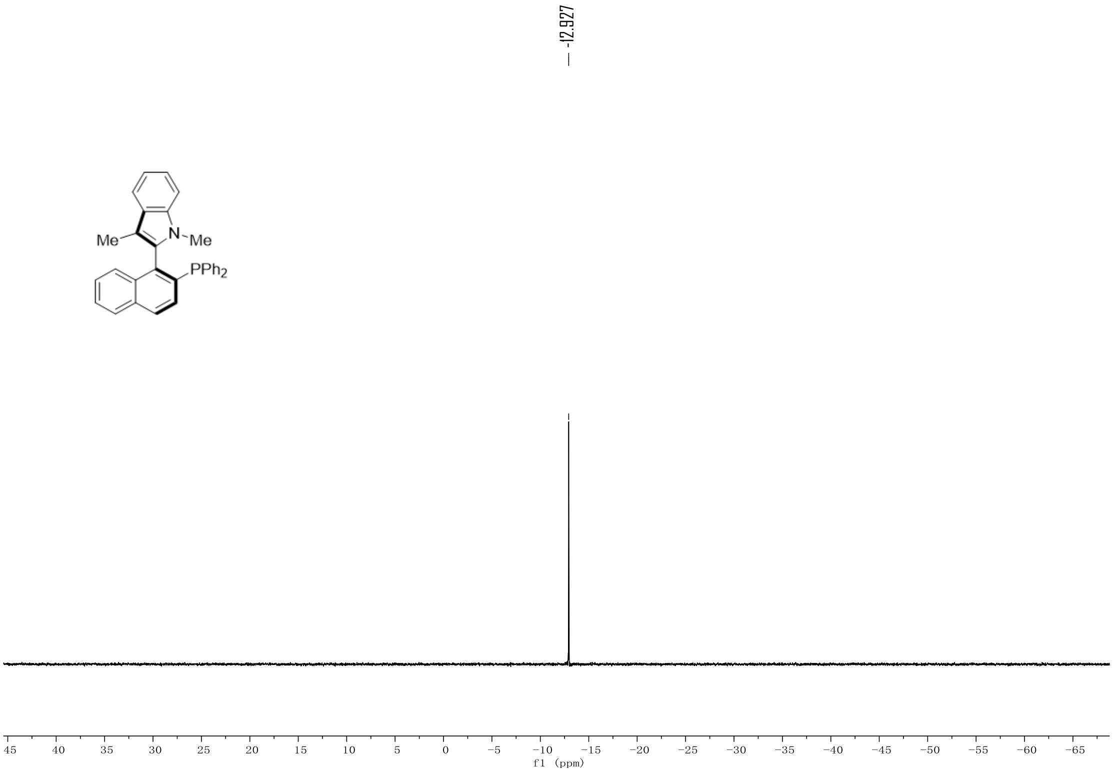

^{1}H NMR spectra of **29** (500 MHz, $CDCl_3$)

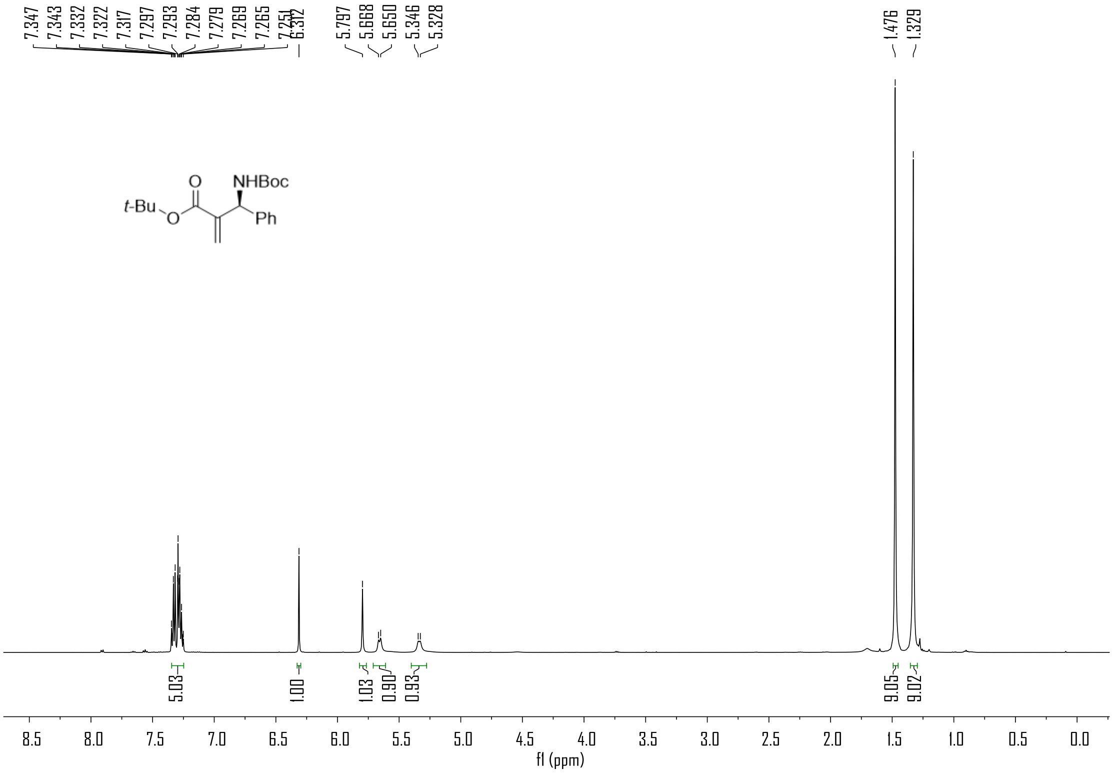

^{13}C NMR spectra of **29** (126 MHz, $CDCl_3$)

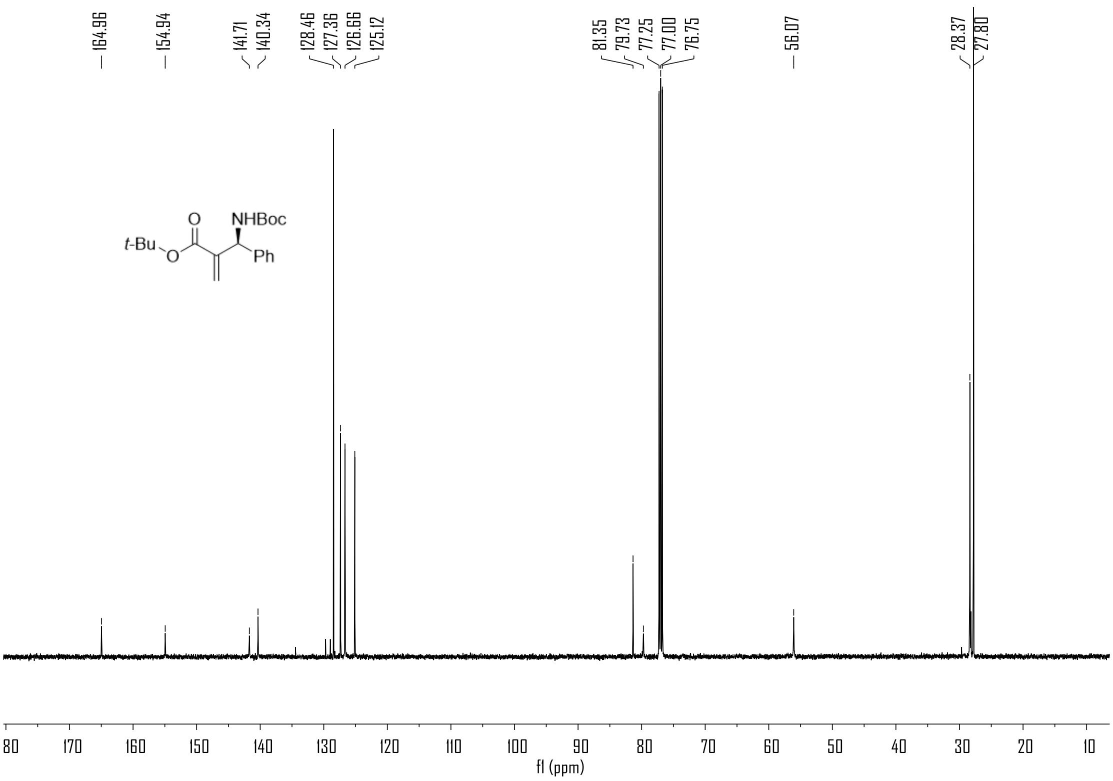

^{1}H NMR spectra of **30** (500 MHz, $CDCl_3$)

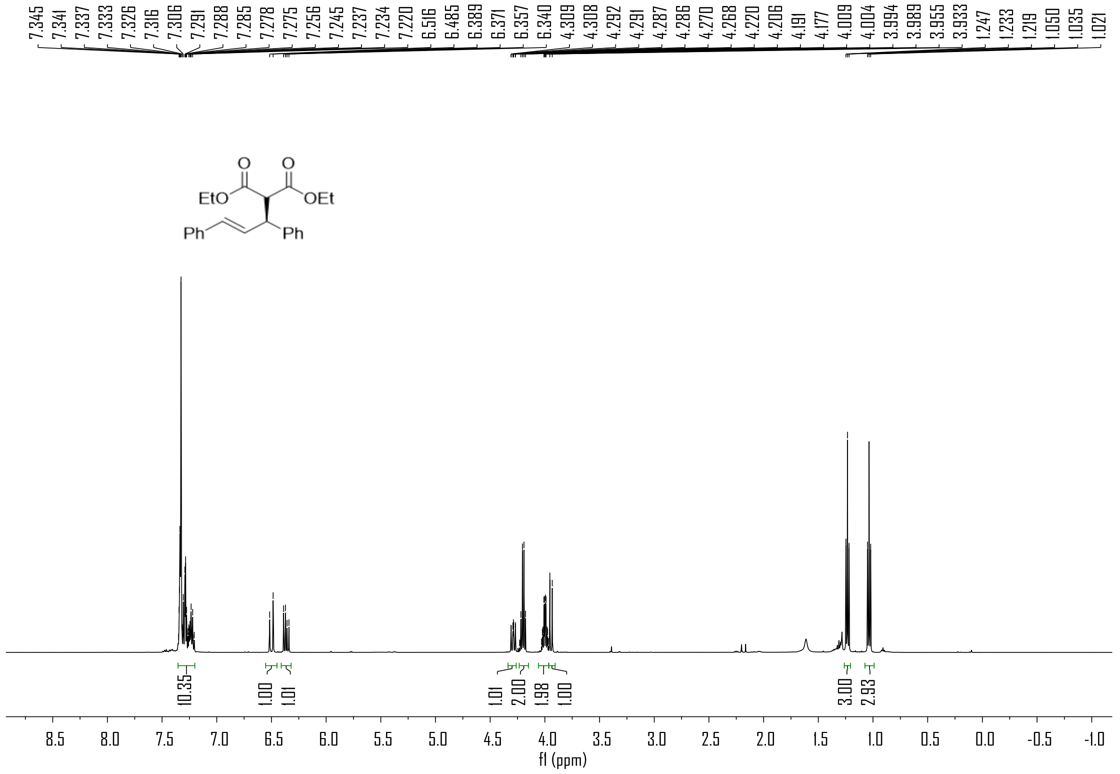

^{13}C NMR spectra of **30** (126 MHz, $CDCl_3$)

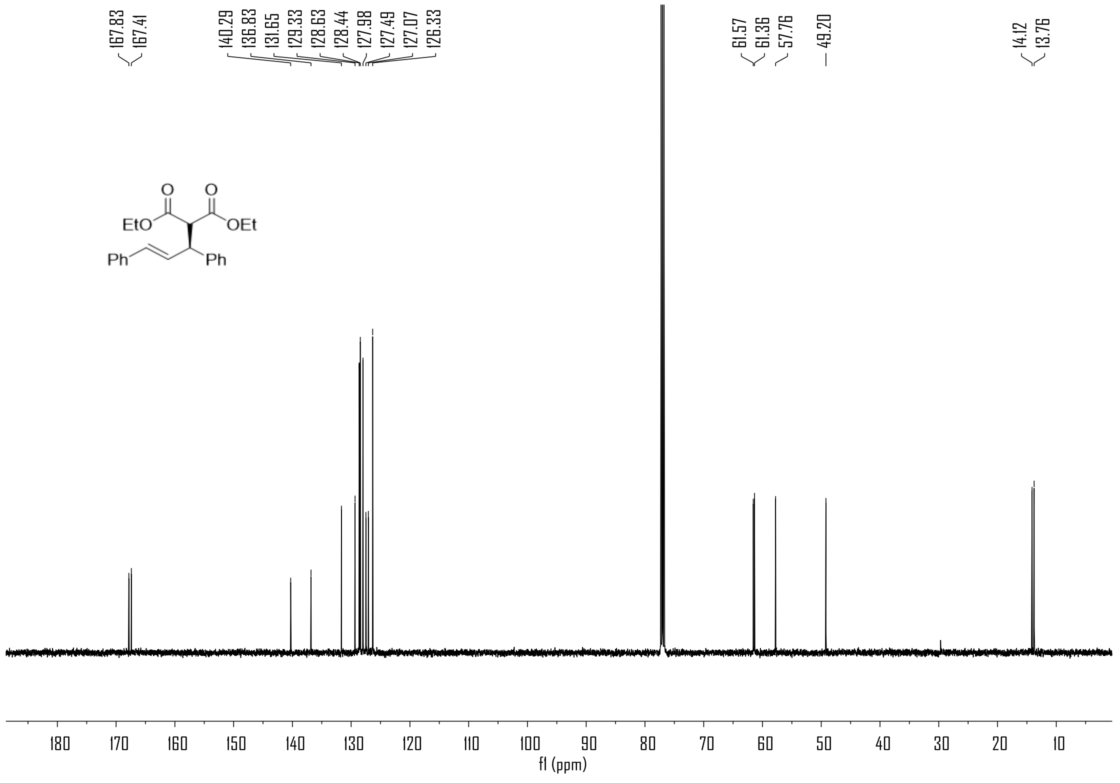

9. Supplementary references

1. Boutadla, Y., Davies, D. L., Jones, R. C., Singh, K. The Scope of Ambiphilic Acetate-Assisted Cyclometallation with Half-Sandwich Complexes of Iridium, Rhodium and Ruthenium. *Chem. Eur. J.* **2011**, *17*, 3438-3448.

2. Qi, L.-W., Rogge, T., Houk, K. N., Lu, Y. Iridium nitrenoid-enabled arene C−H functionalization. *Nat. Catal*. **2024**, *7*, 934–943.

3. Morris, D. M., McGeagh, M., De Peña, D., Merola, J. S. Extending the range of pentasubstituted cyclopentadienyl compounds: The synthesis of a series of tetramethyl(alkyl or aryl)cyclopentadienes (Cp*R), their iridium complexes and their catalytic activity for asymmetric transfer hydrogenation. *Polyhedron* **2014**, *84*, 120-135.

4. Chen, W., Xia, Y., Lin, L., Yuan, X., Guo, S., Liu, X., Feng, X. Asymmetric Synthesis of Furo[3,4-b]indoles by Catalytic [3+2] Cycloaddition of Indoles with Epoxides. *Chem. Eur. J.* **2015**, *21*, 15104-15107.

5. Wang, W., Zhang, F., Liu, Y., Feng, X. Diastereo- and Enantioselective Construction of Vicinal All-Carbon Quaternary Stereocenters via Iridium/Europium Bimetallic Catalysis. *Angew. Chem. Int. Ed.* **2022**, *61*, e202208837.

6. Li, Y., Wang, R., Wang, T., Cheng, X.-F., Zhou, X., Fei, F., Wang, X.-S. A Copper-Catalyzed Aerobic [1,3]-Nitrogen Shift through NitrogenRadical 4-exo-trig Cyclization. *Angew. Chem. Int. Ed.* **2017**, *56*, 15436 –15440.

7. Zhan, B.-B., Wang, L., Luo, J., Lin, X.-F., Shi, B.-F. Synthesis of Axially Chiral Biaryl-2-amines by PdII -CatalyzedFree-Amine-Directed Atroposelective CH Olefination. *Angew. Chem. Int. Ed*. **2020**, *59,* 3568 –3572.

8. Xia W., An, Q.-J., Xiang, S.-H., Li, S., Wang, Y.-B., Tan, B. Chiral Phosphoric Acid Catalyzed Atroposelective C H Amination of Arenes. *Angew. Chem. Int. Ed*. **2020**, *59,* 6775 –6779.

9. Dai, L., Zhou, X., Guo, J., Dai, X., Huang, Q., Lu, Y. Diastereo- and atroposelective synthesis of N-arylpyrroles enabled by light-induced phosphoric acid catalysis. *Nat. Commun*. **2023**, *14,* 4813.
